# Genome-wide identification of rubber tree (*Hevea brasiliensis* Muell. Arg.) aquaporin genes and their response to ethephon stimulation in the laticifer, a rubber-producing tissue

**DOI:** 10.1186/s12864-015-2152-6

**Published:** 2015-11-25

**Authors:** Zhi Zou, Jun Gong, Feng An, Guishui Xie, Jikun Wang, Yeyong Mo, Lifu Yang

**Affiliations:** Danzhou Investigation & Experiment Station of Tropical Crops, Ministry of Agriculture, Rubber Research Institute, Chinese Academy of Tropical Agricultural Sciences, Danzhou, 571737 P. R. China

**Keywords:** Rubber tree (*Hevea brasiliensis* Muell. Arg.), Laticifer, Transcriptome, Aquaporin, Plasma membrane intrinsic protein, Water balance

## Abstract

**Background:**

Natural rubber, an important industrial raw material, is specifically synthesized in laticifers located inside the rubber tree (*Hevea brasiliensis* Muell. Arg.) trunk. Due to the absence of plasmodesmata, the laticifer water balance is mediated by aquaporins (AQPs). However, to date, the characterization of *H. brasiliensis* AQPs (HbAQPs) is still in its infancy.

**Results:**

In this study, 51 full-length AQP genes were identified from the rubber tree genome. The phylogenetic analysis assigned these AQPs to five subfamilies, including 15 plasma membrane intrinsic proteins (PIPs), 17 tonoplast intrinsic proteins (TIPs), 9 NOD26-like intrinsic proteins (NIPs), 4 small basic intrinsic proteins (SIPs) and 6 X intrinsic proteins (XIPs). Functional prediction based on the analysis of the aromatic/arginine (ar/R) selectivity filter, Froger’s positions and specificity-determining positions (SDPs) showed a remarkable difference in substrate specificity among subfamilies. Homology analysis supported the expression of 44 HbAQP genes in at least one of the examined tissues. Furthermore, deep sequencing of the laticifer transcriptome in the form of latex revealed a key role of several PIP subfamily members in the laticifer water balance, and qRT-PCR analysis showed diverse expression patterns of laticifer-expressed HbAQP genes upon ethephon treatment, a widely-used practice for the stimulation of latex yield.

**Conclusions:**

This study provides an important genetic resource of HbAQP genes, which will be useful to improve the water use efficiency and latex yield of *Hevea*.

**Electronic supplementary material:**

The online version of this article (doi:10.1186/s12864-015-2152-6) contains supplementary material, which is available to authorized users.

## Background

Aquaporins (AQPs), also known as major intrinsic proteins (MIPs), are a class of integral membrane proteins that facilitate the passive transport of water and other small solutes across biological membranes [1]. Since their first discovery in 1990s, AQPs have been found in almost all living organisms [2]. Compared with animals and microbes, AQPs are particularly abundant and diverse in land plants and more than 20 homologs have been identified from *Arabidopsis thaliana*, *Zea mays*, *Oryza sativa*, *Vitis vinifera*, *Populus trichocarpa*, *Gossypium hirsutum*, *Solanum tuberosum*, *Solanum lycopersicum* and *Glycine max* [3–11]. According to the sequence similarity, the AQPs in model plant *Arabidopsis* are divided into four subfamilies, i.e. the plasma membrane intrinsic protein (PIP) subfamily that contains two subgroups, the tonoplast intrinsic protein (TIP) that contains five subgroups, the NOD26-like intrinsic protein (NIP) that contains seven subgroups and small basic intrinsic protein (SIP) that contains two subgroups. In moss and some dicots including *P. trichocarpa*, one more subfamily named X intrinsic protein (XIP) subfamily that contains three subgroups is also found [7, 12].

AQPs assemble in tetramers in the cell membrane, although each monomer can act as a water channel [13]. Even though the overall pairwise sequence similarity can be low, AQPs share some structural features such as harboring six transmembrane helices (TM1–TM6) connected by five loops (LA–LE). LB and LE from opposite sides dip into the membrane and form two half helices (HB and HE), at the N-termini of which, two highly conserved NPA (Asn-Pro-Ala) motifs form one selectivity region. And another region that determines the substrate specificity is known as the aromatic/arginine (ar/R) selectivity filter (H2 in TM2, H5 in TM5, LE1 and LE2 in LE) [13]. The NPA motifs create an electrostatic repulsion of protons and act as a size barrier, where the ar/R filter renders the pore constriction site diverse in both size and hydrophobicity [13]. In addition to water-conducting AQPs, certain AQPs called aquaglyceroporins (GLPs) transport glycerol instead. Statistical analysis indicated that GLPs feather five highly conserved amino acid residues (named Froger’s positions: P1–5): an aromatic residue at P1, an acidic residue at P2, a basic residue at P3, a proline followed by a nonaromatic residue at P4 and P5, as Y^108^-D^207^-K^211^-P^236^-L^237^ observed in the *Escherichia coli* glycerol facilitator GlpF in contrast to A^103^-S^190^-A^194^-F^208^-W^209^ in the pure water channel AqpZ [14]. Very recently, nine specificity-determining positions (SDPs) for non-aqua substrates, i.e. urea, boric acid, silicic acid, ammonia (NH_3_), carbon dioxide (CO_2_) and hydrogen peroxide (H_2_O_2_) were also proposed for each group via a comprehensive analysis of functionally characterized AQPs [15].

The para rubber tree (*Hevea brasiliensis* Muell. Arg.) is a perennial tropical plant species that belongs to the Euphorbiaceae family. Although it is native to the Amazon basin, the economic importance and increasing demand of natural rubber has prompted its wide-domestication to Southeast Asia. To date, the rubber tree is still the only commercial source of natural rubber for its high production and quality of rubber. Natural rubber (*cis*-1,4-polyisoprene) is specifically synthesized in the highly differentiated vessels termed laticifers, which are located in the secondary phloem of the tree trunk and are periodically differentiated from the vascular cambium [16]. The rubber-containing latex which represents the cytoplasmic content of laticifers is harvested by tapping the bark every 2–5 days, and the latex yield is determined by the initial flow rate, duration of latex flow and latex regeneration between two tappings [17].

Ethylene, a gas phytohormone, plays crucial roles in numerous aspects of growth, development, and response to biotic and abiotic stresses in plants [18]. In *Arabidopsis*, the ethylene signaling pathway has been well established and its involvement in certain agronomically important processes such as seed dormancy, fruit ripening, abscission and senescence has made ethylene a target for manipulation by chemical and biotechnological methodologies [19]. Ethephon (also known as Ethrel), an ethylene releaser, was initiatively tested for rubber yield promotion as early as the 1970s and now is widely used in rubber production all over the world. Although the molecular mechanism on ethephon stimulation of latex yield is still unclear, researches showed that the treatment of rubber tree barks with ethephon could significantly decrease latex dry rubber content (DRC) or total solid content (TSC), extend the bark drainage area, and prolong latex flow duration [20–23]. These effects are mainly benefited from water influx toward laticifers and the resultant latex dilution.

Since water accounts for 60–70 % of total latex upon each tapping, sufficient water supply is essential for both latex flow and latex regeneration [24]. Nevertheless, the mature laticiferous vessel rings are devoid of functional plasmodesmata connections [25], and thus the water inflow into laticifers is mediated largely by AQPs. However, the molecular characterization of rubber tree AQPs (HbAQPs) is still in its infancy. As of June 2015, only eight full-length HbAQP cDNAs have been reported [21–23, 26–29]. Among them, the water transport activity of HbPIP1, HbPIP2, HbPIP1;1, HbPIP2;1 HbPIP2;3 and HbTIP1;1 was characterized by Tungngoen et al. and our group using *Xenopus laevis* oocytes: HbTIP1;1, HbPIP2;1 and HbPIP2;3 were shown to be highly efficient, whereas HbPIP1;1; HbPIP1;4 and HbPIP2;7 were less efficient [21–23, 26].

Lately, the rubber tree genome was sequenced by Rahman et al. [30] and Rubber Research Institute, Chinese Academy of Tropical Agricultural Sciences. In addition, more than 50,000 ESTs (expressed sequence tags) and a high number of RNA sequencing reads derived from several tissues such as shoot apex, leaf, laticifer, bark, root and somatic embryogenesis are also available in NCBI SRA [30–35]. These datasets provide a good chance to analyze the rubber tree AQP gene family from a global view. In the present study, a genome-wide search was carried out to identify AQP genes encoded in the rubber tree genome. Further, functional prediction was performed based on the analysis of the ar/R filter, Froger’s positions and SPDs, and deep transcriptome sequencing and qRT-PCR expression analysis were also adopted to identify the most important AQP members expressed in the laticifer.

## Results

### Identification and classification of rubber tree aquaporin genes

Via a comprehensive homology analysis, 57 or 54 loci putatively encoding AQP-like genes were identified from the rubber tree genome of clone RY7-33-97 or RRIM600, respectively (data not shown). Since all AQP-encoding loci identified in the RRIM600 genome were found in the genome of RY7-33-97 and some genes from RRIM600 are incomplete and/or the sequences have a high number of “N”s, the AQP genes identified from the RY7-33-97 genome were selected for further analyses. After discarding loci encoding partial AQP-like sequences which are truncated and lacking any of the NPA motifs, 51 full-length AQP genes were retained and the gene models are available in Additional file 1.

**Fig. 1:**
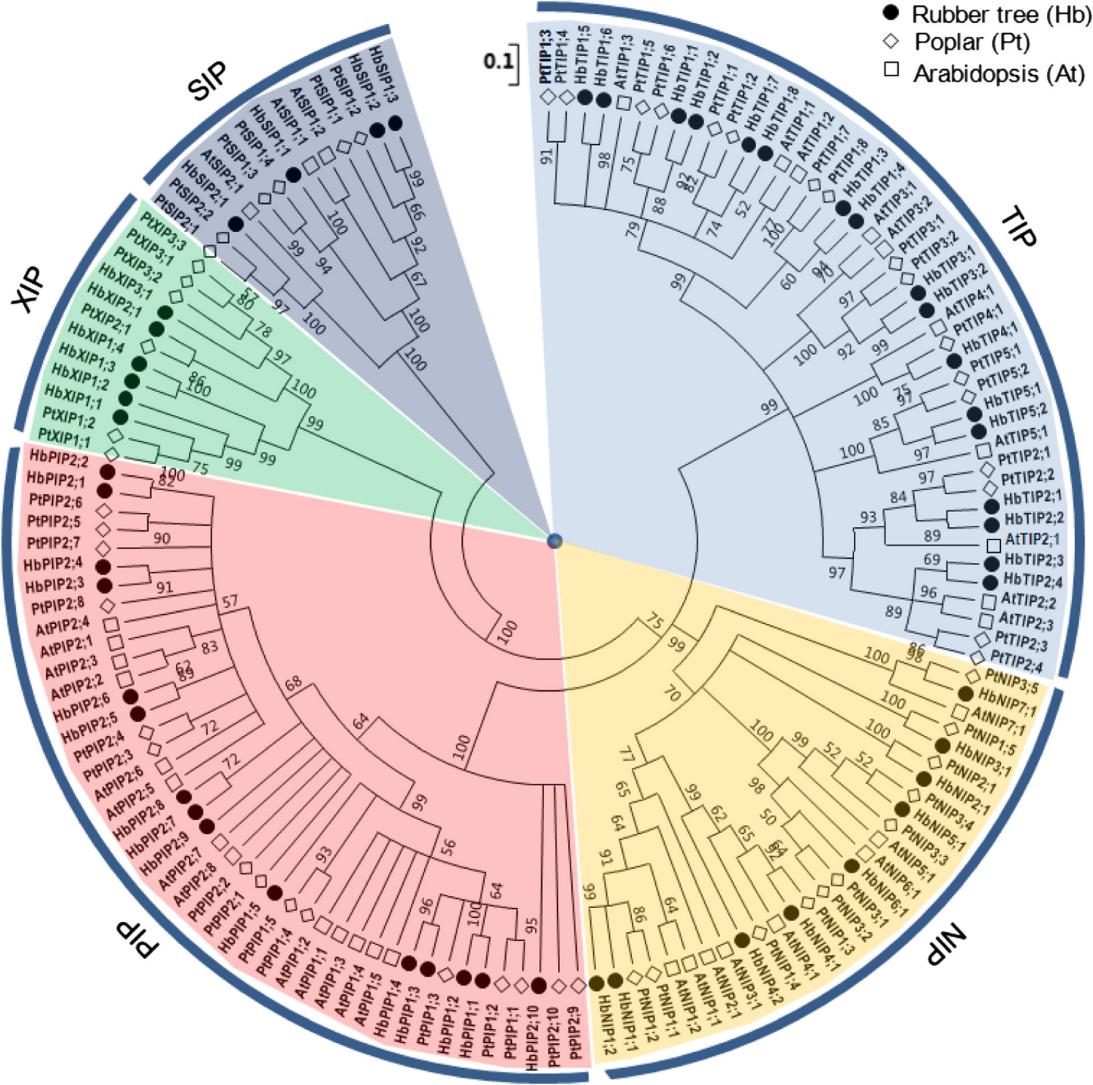
Phylogenetic analysis of deduced amino acid sequences of the 51 HbAQPs with *Arabidopsis* and poplar homologs. Amino acid sequences were aligned using ClustalX and the phylogenetic tree was constructed using bootstrap maximum likelihood tree (1000 replicates) method and MEGA6 software. The distance scale denotes the number of amino acid substitutions per site. The name of each subfamily is indicated next to the corresponding group. Species and accession numbers are listed in Table 1 and Additional file 2

To analyze the evolutionary relationship and their putative function, an unrooted phylogenetic tree was constructed from the deduced amino acid sequences of HbAQPs together with that from *Arabidopsis* (AtAQPs) and poplar (PtAQPs) (the Phytozome accession numbers are available in Additional file 2) . The reasons for choosing these two species are mainly as follows: the complete set of AQP genes in *Arabidopsis* was firstly identified and then well characterized; the well-studied wood plant poplar harbors one more subfamily (XIP) that is not found in *Arabidopsis*. According to the phylogenetic analysis, 51 HbAQPs were grouped into five subfamilies, i.e. PIP (15), TIP (17), NIP (9), SIP (4) and XIP (6) (Table 1; Fig. 1). Following the nomenclature of *Arabidopsis* and poplar [3, 36], the HbPIP subfamily was further divided into two phylogenetic subgroups (5 HbPIP1s and 10 HbPIP2s), the HbTIP subfamily into five subgroups (8 HbTIP1s, 4 HbTIP2s, 2 HbTIP3s, 1 HbTIP4 and 2 HbTIP5s), the HbNIP subfamily into seven subgroups (2 HbNIP1s, 1 HbNIP2, 1 HbNIP3, 2 HbNIP4s, 1 HbNIP5, 1 HbNIP6 and 1 HbNIP7), the HbSIP subfamily into two subgroups (3 HbSIP1s and 1 HbSIP2) and the HbXIP subfamily into three subgroups (4 HbXIP1s, 1 HbXIP2 and 1 HbXIP3) (Fig. 1). Although the closest homolog of HbNIP2;1 and HbNIP3;1 is not AtNIP2;1 or AtNIP3;1, their counterparts in poplar were identified and thus the names were assigned. As shown in Fig. 1, many HbAQPs were grouped in pairs, i.e. HbPIP1;1/HbPIP1;2, HbPIP1;3/HbPIP1;4, HbPIP2;1/HbPIP2;2, HbPIP2;3/HbPIP2;4, HbPIP2;5/HbPIP2;6, HbPIP2;7/HbPIP2;8, HbTIP1;1/HbTIP1;2, HbTIP1;3/HbTIP1;4, HbTIP1;5/HbTIP1;6, HbTIP1;7/HbTIP1;8, HbTIP2;1/HbTIP2;2, HbTIP2;3/HbTIP2;4, HbTIP3;1/HbTIP3;2, HbTIP5;1/HbTIP5;2, HbNIP1;1/HbNIP1;2, HbSIP1;2/HbSIP1;3, HbXIP1;3/HbXIP1;4, which exhibit sequence identities of 78.9–96.3 % and 71.9–98.3 % at the nucleotide or amino acid level, respectively (Additional file 3).

**Table 1:** List of 51 HbAQP genes identified in this study

	Name	Nucleotide length (bp, from start to stop codons)		Intron NO.	EST hits in GenBank	Shoot apex^a^	Leaf^b^	laticifer^c^	Bark^d^	Root^e^	Somatic embryogenesis^f^	Cloning strategy	Phytozome ID of AtAQP ortholog	Phytozome ID of PtAQP ortholog
		Gene	CDS											
PIP	*HbPIP1;1*	1161	864	3	10	Yes	Yes	Yes	Yes	Yes	Yes	RT-PCR	AT4G00430	Pt_0010s19930
	*HbPIP1;2*	1155	864	3	6	Yes	Yes	Yes	Yes	Yes	Yes	RT-PCR	AT4G00430	Pt_0010s19930
	*HbPIP1;3*	2952	864	3	0	Yes	Yes	Yes	Yes	Yes	Yes	RT-PCR	AT4G00430	Pt_0003s12870
	*HbPIP1;4*	2285	864	3	4	Yes	Yes	Yes	Yes	Yes	Yes	RACE	AT4G00430	Pt_0003s12870
	*HbPIP1;5*	1370	810	3	2	Yes		Yes	Yes	Yes		RT-PCR	AT4G00430	Pt_0016s12070
	*HbPIP2; 1*	1150	867	3	4	Yes	Yes	Yes	Yes	Yes	Yes	RT-PCR	AT3G53420	Pt_0006s12980
	*HbPIP2;2*	1174	867	3	2	Yes	Yes		Yes	Yes	Yes		AT3G53420	Pt_0006s12980
	*HbPIP2;3*	1801	861	3	2	Yes	Yes	Yes	Yes	Yes	Yes	RT-PCR	AT3G53420	Pt_0006s12980
	*HbPIP2;4*	1590	861	3	0	Yes	Yes	Yes	Yes	Yes	Yes	RT-PCR	AT3G53420	Pt_0006s12980
	*HbPIP2;5*	1673	858	3	0	Yes	Yes	Yes	Yes	Yes	Yes	RT-PCR	AT3G53420	Pt_0010s22950
	*HbPIP2;6*	2927	861	3	0	Yes	Yes		Yes	Yes	Yes		AT3G53420	Pt_0010s22950
	*HbPIP2; 7*	1263	837	3	15	Yes	Yes	Yes	Yes	Yes	Yes	RACE	AT3G53420	Pt_0004s18240
	*HbPIP2;8*	1263	843	3	1	Yes	Yes		Yes	Yes			AT3G53420	Pt_0004s18240
	*HbPP2;9*	1120	843	3	0	Yes	Yes		Yes	Yes	Yes		AT3G53420	Pt_0004s18240
	*HbPIP2; 1*	1401	858	3	0					Yes			AT3G53420	Pt_0005s11110
TIP	*HbTIP1;1*	853	759	1	1	Yes	Yes		Yes	Yes	Yes	RT-PCR	AT2G36830	Pt_0006s12350
	*HbTIP1;2*	851	759	1	0	Yes	Yes	Yes	Yes	Yes	Yes	RT-PCR	AT2G36830	Pt_0006s12350
	*HbTP1;3*	848	762	1	0	Yes	Yes			Yes			AT2G36830	Pt_0009s01070
	*HbTIP1;4*	820	684	1	0								AT2G36830	Pt_0009s01070
	*HbTP1;5*	931	759	2	2	Yes	Yes		Yes	Yes	Yes		AT4G01470	Pt_0008s05050
	*HbTIP1;6*	974	759	2	0	Yes	Yes	Yes	Yes	Yes	Yes	RT-PCR	AT4G01470	Pt_0008s05050
	*HbTIP1;7*	1189	759	2	0	Yes				Yes	Yes		AT4G01470	Pt_0001s24200
	*HbTIP1;8*	997	759	2	0	Yes	Yes		Yes	Yes	Yes		AT4G01470	Pt_0001s24200
	*HbTP2;1*	1574	747	2	0	Yes	Yes		Yes	Yes	Yes		AT3G16240	Pt_0001s18730
	*HbTIP2;2*	1165	747	2	0	Yes	Yes	Yes	Yes	Yes	Yes	RT-PCR	AT3G16240	Pt_0001s18730
	*HbTIP2;3*	1010	753	2	0	Yes				Yes	Yes		AT4G17340	Pt_0003s07550
	*HbTIP2;4*	1009	753	2	0					Yes			AT4G17340	Pt_0003s07550
	*HbTIP3;1*	967	774	2	0	Yes				Yes	Yes		AT1G17810	Pt_0017s03540
	*HbTIP3;2*	915	742	2	0					Yes			AT1G17810	Pt_0017s03540
	*HbTIP4;1*	1010	756	2	0	Yes	Yes		Yes	Yes	Yes		AT2G25810	Pt_0006s25620
	*HbTIP5;1*	1181	759	2	0					Yes	Yes		AT3G47440	Pt_0001s00690
	*HbTIP5;2*	1119	744	2	0								AT3G47440	Pt_0001s00690
NIP	*HbNIP1;1*	1582	861	4	0			Yes	Yes	Yes		RT-PCR	AT4G18910	Pt_0011s06770
	*HbNP1;2*	1939	864	4	0	Yes	Yes	Yes	Yes	Yes	Yes	RT-PCR	AT4G18910	Pt_0011s06770
	*HbNIP2;1*	2930	858	4	0		Yes		Yes	Yes	Yes			Pt_0017s11960
	*HbNP3;1*	1249	849	4	0			Yes		Yes		RT-PCR		Pt_0002s09740
	*HbNIP4;1*	1237	804	4	0				Yes				AT5G37810	Pt_0010s12330
	*HbNIP4;2*	1268	846	4	0				Yes				AT5G37820	Pt_0017s03060
	*HbNIP5;1*	1932	897	3	0	Yes	Yes		Yes	Yes	Yes		AT4G10380	Pt_0001s45920
	*HbNIP6;1*	3371	927	4	0	Yes	Yes	Yes	Yes	Yes	Yes	RT-PCR	AT1G80760	Pt_0001s14850
	*HbNIP7;1*	1359	897	4	0								AT3G06100	Pt_0008s20750
XIP	*HbXIP1;1*	973	870	1	0									Pt_0009s13090
	*HbXIP1;2*	1022	891	1	0					Yes				Pt_0009s13090
	*HbXIP1;3*	959	831	1	0									Pt_0009s13090
	*HbXIP1;4*	959	831	1	0									Pt_0009s13090
	*HbXIP2;1*	1163	918	2	0	Yes	Yes		Yes	Yes	Yes	RT-PCR		Pt_0009s13110
	*HbXIP3; 1*	771	771	0	0									Pt_0009s13070
SIP	*HbSIP1;1*	720	720	0	0	Yes	Yes		Yes	Yes			AT3G04090	Pt_0014s15250
	*HbSIP1;2*	4572	720	2	0	Yes	Yes	Yes	Yes	Yes	Yes	RT-PCR	AT3G04090	Pt_0019s04640
	*HbSP1;3*	5438	720	2	0	Yes	Yes		Yes	Yes	Yes		AT3G04090	Pt_0019s04640
	*HbSIP2;1*	13833	723	2	0	Yes	Yes	Yes	Yes	Yes	Yes	RT-PCR	AT3G56950	Pt_0016s02560

^a^Based on the 454 transcriptome data under the NCBI SRA accession number of DRX000223; ^b ^Based on the 454 transcriptome data of SRX451708 and Illumina transcriptome data of SRX206128, SRX206129, SRX206130, SRX203083, SRX203085, SRX203117, SRX203118 and SRX278515; ^c ^Based on the 454 transcriptome data of SRX451705 and Illumina transcriptome data of SRX037405, SRX206131, SRX206132 and SRX278514; ^d ^Based on the 454 transcriptome data of SRX451707 and Illumina transcriptome data of SRX278513; ^e ^Based on the 454 transcriptome data of SRX451710; ^f ^Based on the 454 transcriptome data of SRX451709. Read-mapping was carried out using Bowtie2 with default parameters, and mapped read number of more than one was counted as “Yes” representing detected genes

Homology search showed that 11 out of 51 HbAQP genes have EST hits in GenBank (as of June 2015), i.e. *HbPIP1;1*, *HbPIP1;2*, *HbPIP1;4*, *HbPIP1;5*, *HbPIP2;1*, *HbPIP2;2*, *HbPIP2;3*, *HbPIP2;7*, *HbPIP2;8*, *HbTIP1;1* and *HbTIP1;5*. Eight full-length HbAQP cDNAs, including *HbPIP1;1* (GenBank accession number GQ903902), *HbPIP1;4* (denoted as *HbPIP1* under the accession number of GQ479823), *HbPIP2;1* (FJ851079), *HbPIP2;2* (KP990544), *HbPIP2;5* (denoted as *HbPIP2;3* under the accession number of KF921089), *HbPIP2;7* (denoted as *HbPIP2* under the accession number of GQ479824), *HbTIP1;1* (FJ851080) and *HbTIP1;2* (denoted as *HbTIP1* under the accession number of KP990545) have been reported [21–23, 26–29]. Read alignments against RNA sequencing data of rubber tree shoot apex, leaf, laticifer, bark, root and somatic embryogenesis [30–35] indicated that the expression of 44 HbAQP genes was observed in at least one of the examined tissues (Table 1). Whereas, seven genes coding for two *TIP*s (*HbTIP1;4* and *HbTIP5;2*), one *NIP* (*HbNIP7;1*), four *XIP*s (*HbXIP1;1*, *HbXIP1;3*, *HbXIP1;4* and *HbXIP3;1*) might be expressed exclusively in response to a specific stimulus or in a very specific part of the plant and thus are excluded in the available datasets. In *Arabidopsis*, the orthologs of *HbTIP1;4* (*AtTIP1;3*) and *HbNIP7;1* (*AtNIP7;1*) were also shown to be pollen or anther-specific, respectively [37, 38]. Besides supported by ESTs and/or RNA sequencing reads, the exon-intron structures of laticifer-expressed HbAQP genes (see below) were also confirmed with cloned cDNAs (Table 1).

### Analysis of exon-intron structure

**Fig. 2:**
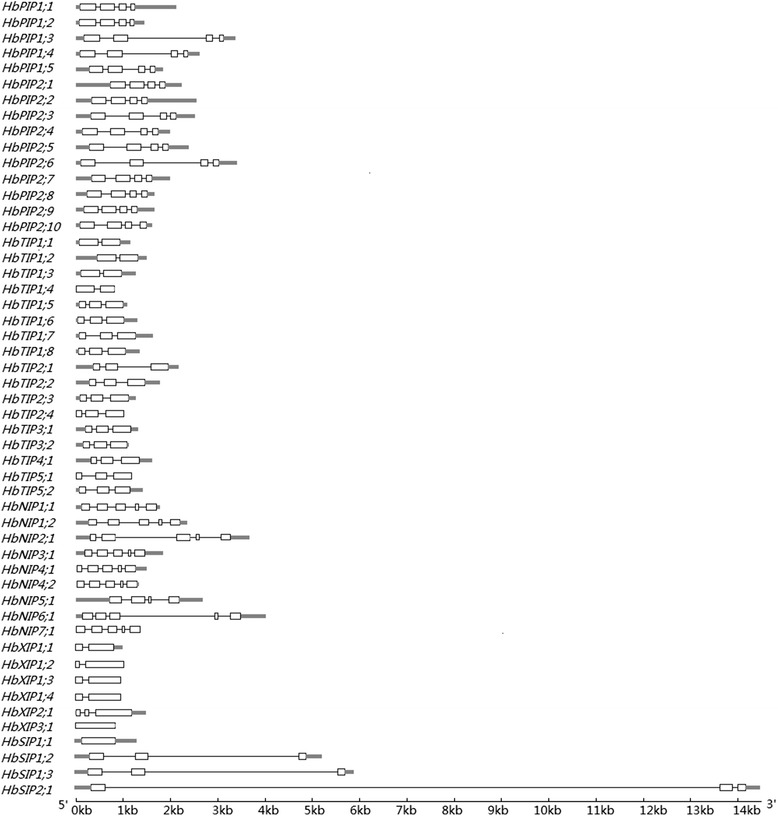
Exon-intron structures of the 51 HbAQP genes. Shown is a graphic representation of the gene models of all 51 HbAQPs identified in this study using GSDS. UTRs are shown as gray boxes, exons are shown as white boxes and introns are shown as black lines

The exon-intron structures of the 51 HbAQP genes were analyzed based on the gene models. Although the ORF (open reading frame) length of each gene is similar (684–927 bp), the gene size (from start to stop codons) is distinct (720–13833 bp, Table 1; Fig. 2). The introns of HbAQP genes harbor an average length of 404 bp, with the minimum of 71 bp in *HbNIP2;1* and the maximum of 13000 bp in *HbSIP2;1*. Genes in different subfamilies harbor distinct exon-intron structures. All members of the HbPIP subfamily feature three introns (92–736 bp, 78–1650 bp and 80–186 bp, respectively). Except for *HbTIP1;1*, *HbTIP1;2*, *HbTIP1;3* and *HbTIP1;4* that contain only one intron, other HbTIP genes contain two introns instead. HbNIP genes usually have four introns except for *HbNIP5;1* containing three introns. Most HbSIP genes contain two introns except for *HbSIP1;1* without any intron. Subgroups of HbXIP genes vary in the number of introns: one intron for subgroup one, two or zero for subgroups two and three, respectively (Fig. 2).

### Structural features of HbAQPs

Sequence analysis showed that the 51 deduced HbAQPs consist of 227–305 amino acids, with a theoretical molecular weight of 23.78–32.28 kDa and a *p*I value of 4.59–9.74. Homology analysis revealed a high sequence diversity existing within and between the five subfamilies. The sequence similarities of 66.4–99.3 % were found within HbPIPs, 49.8–98.4 % within HbTIPs, 45.3–93.1 % within HbXIPs, 44.6–90.6 % within HbNIPs, and 40.6–95.0 % within HbSIPs. HbPIPs share the highest sequence similarity of 35.2–49.0 % with HbTIPs, 34.0–41.8 % with HbXIPs, 30.2–36.8 % with HbNIPs, and the lowest of 22.9–32.5 % with HbSIPs. HbTIPs show 28.7–46.4 %, 28.1–39.9 % and 24.2–41.1 % sequence similarities with HbNIPs, HbXIPs and HbSIPs, respectively. HbNIPs share sequence similarities of 26.0–35.0 % and 23.0–32.2 % with HbXIPs and HbSIPs, whereas HbSIPs share the lowest similarity of 20.8–33.7 % with HbXIPs (Additional file 4).

Topological analysis showed that all HbAQPs were predicted to harbor six transmembrane helical domains (Table 2), which is consistent with the results from multiple alignments with structure proven AQPs (see Additional file 5). The subcellular localization of each HbAQP was also predicted (Table 2). HbPIPs with an average *p*I value of 8.47 are localized to plasma membranes. HbTIPs with an average *p*I value of 5.81 are mainly localized to vacuoles (known as lutoids in laticifers with a natural pH of about 6), though several members were predicted to be localized to endoplasmic reticulum (ER), chloroplast and cytosol. HbNIPs with an average *p*I value of 7.60 are mostly localized to plasma membranes, but HbNIP2;1 and HbNIP3;1 were predicted to be localized to the membrane of vacuole and chloroplast, respectively. Two members (HbSIP1;2 and HbSIP1;3) of the SIP subfamily (with an average *p*I value of 9.06) were predicted to be localized to plasma membranes, whereas HbSIP1;1 and HbSIP2;1 are localized to the membrane of vacuole and chloroplast, respectively. Although the XIP subfamily harbors only six members (with an average *p*I value of 7.95), the predicted localizations are diverse, including the vacuole, chloroplast, plasma membrane and cytosol. To learn more about the putative function of HbAQPs, the conserved residues typical of dual NPA motifs, the ar/R filter, five Froger’s positions and nine SDPs were also identified (Tables 2 and 3).

**Table 2:** Structural and subcellular localization analysis of the HbAQPs

Name	Len	Mw (KDa)	pI	TM^a^	TM^b^	TM^c^	Loc^d^	Ar/R selectivity filter				NPA motifs		Froger's positions				
								H2	H5	LE1	LE2	LB	LE	PI	P2	P3	P4	P5
HbPIP1;1	287	30.80	8.59	6	6	6	Plas	F	H	T	R	NPA	NPA	E	S	A	F	W
HbPIP1;2	287	30.80	8.59	6	6	6	Plas	F	H	T	R	NPA	NPA	E	S	A	F	W
HbPIP1;3	287	30.74	8.24	6	6	6	Plas	F	H	T	R	NPA	NPA	E	S	A	F	W
HbPIP1;4	287	30.78	8.62	6	6	6	Plas	F	H	T	R	NPA	NPA	E	S	A	F	W
HbPIP1;5	269	28.92	8.60	5	6	5	Plas	F	H	T	R	NPA	NPA	Q	S	A	F	W
HbPIP2;1	288	30.71	7.61	6	6	6	Plas	F	H	T	R	NPA	NPA	Q	S	A	F	W
HbPIP2;2	288	30.71	8.20	6	6	6	Plas	F	H	T	R	NPA	NPA	Q	S	A	F	W
HbPIP2;3	286	30.58	8.50	6	6	6	Plas	F	H	T	R	NPA	NPA	Q	S	A	F	W
HbPIP2;4	286	30.61	8.19	6	6	6	Plas	F	H	T	R	NPA	NPA	Q	S	A	F	W
HbPIP2;5	285	30.34	9.18	6	6	6	Plas	F	H	T	R	NPA	NPA	Q	S	A	F	W
HbPIP2;6	286	30.39	9.06	6	6	6	Plas	F	H	T	R	NPA	NPA	Q	S	A	F	W
HbPIP2;7	278	29.56	9.11	6	6	6	Plas	F	H	T	R	NPA	NPA	M	S	A	F	W
HbPIP2;8	280	29.76	8.97	6	6	6	Plas	F	H	T	R	NPA	NPA	M	S	A	F	W
HbPIP2;9	280	29.84	6.51	6	6	6	Plas	F	H	T	R	NPA	NPA	M	S	A	F	W
HbPIP2;10	285	30.45	9.05	6	6	6	Plas	F	H	T	R	NPA	NPA	M	S	A	F	W
HbTIP1;1	252	25.91	5.91	7	6	7	Vacu	H	I	A	V	NPA	NPA	T	S	A	Y	W
HbTIP1;2	252	26.07	5.70	7	6	7	Vacu	H	I	A	V	NPA	NPA	T	S	A	Y	W
HbTIP1;3	253	26.44	6.27	6	6	6	Cyto	H	I	A	V	NPA	NPA	T	S	A	Y	W
HbTIP1;4	227	23.78	6.18	3	6	4	Vacu	H	I	A	V	NPA	NPA	T	S	A	Y	W
HbTIP1;5	252	25.88	4.96	7	6	6	Vacu/Cyto	H	I	A	V	NPA	NPA	T	S	A	Y	W
HbTIP1;6	252	25.79	5.70	7	6	7	Cyto	H	I	A	V	NPA	NPA	T	S	A	Y	W
HbTIP1;7	252	25.90	4.97	6	6	6	Vacu/Cyto	H	I	A	V	NPA	NPA	T	S	A	Y	W
HbTIP1;8	252	25.72	4.79	6	7	6	Vacu/Cyto	H	I	A	V	NPA	NPA	T	S	A	Y	W
HbTIP2;1	248	25.40	5.33	7	6	6	Vacu	H	I	G	R	NPA	NPA	T	S	A	Y	W
HbTIP2;2	248	25.34	5.59	7	6	7	Vacu	H	I	G	R	NPA	NPA	T	S	A	Y	W
HbTIP2;3	250	25.31	4.87	6	6	6	Vacu	H	i	G	R	NPA	NPA	T	S	A	Y	W
HbTIP2;4	250	25.31	4.59	4	6	5	Vacu/Plas	H	I	G	S	NPA	NPA	A	S	A	Y	W
HbTIP3;1	257	27.38	6.43	6	6	6	Cyto	H	I	A	R	NPA	NPA	T	A	A	Y	W
HbTIP3;2	243	25.66	9.74	6	6	6	Cyto	H	I	A	R	NPA	NPA	T	A	A	Y	W
HbTIP4;1	251	26.24	5.91	6	6	7	Vacu	H	I	A	R	NPA	NPA	T	S	A	Y	W
HbTIP5;1	252	25.95	6.71	5	6	6	ER	N	V	G	C	NPA	NPA	I	A	A	Y	W
HbTIP5;2	247	25.32	5.13	6	6	6	Chlo	N	V	G	C	NPA	NPA	I	A	A	Y	W
HbNIP1;1	286	30.41	7.57	6	6	6	Plas	W	V	A	R	NPA	NPA	F	S	A	Y	L
HbNIP1;2	287	30.39	8.94	6	6	6	Plas	W	V	A	R	NPA	NPA	F	S	A	Y	I
HbNIP2;1	285	29.88	8.72	6	6	6	Vacu	G	S	G	R	NPA	NPA	L	S	A	Y	I
HbNIP3;1	282	30.20	8.34	6	6	5	Chlo	W	V	A	R	NPA	NPA	F	S	A	F	I
HbNIP4;1	267	28.80	5.28	6	6	6	Plas	W	V	G	R	NPA	NPV	L	S	A	Y	I
HbNIP4;2	281	29.60	5.71	6	6	6	Plas	W	V	A	R	NPA	NPA	F	S	A	Y	I
HbNIP5;1	298	30.98	8.65	6	6	5	Plas	A	I	G	R	NPS	NPV	F	T	A	Y	L
HbNIP6;1	305	31.58	8.68	6	6	5	Plas	T	I	A	R	NPS	NPV	F	T	A	Y	L
HbNIP7;1	298	31.85	6.50	6	6	6	Plas	A	V	G	R	NPA	NPA	Y	S	A	Y	I
HbSIP1;1	239	25.35	9.54	5	6	5	Vacu	A	V	P	N	NPT	NPA	M	A	A	Y	W
HbSIP1;2	239	25.97	7.74	6	6	6	Plas	F	V	P	N	NPT	NPA	M	A	A	Y	W
HbSIP1;3	239	25.83	9.36	6	6	5	Plas	V	V	P	N	NPT	NPA	M	A	A	Y	W
HbSIP2;1	240	26.40	9.60	4	6	5	Chlo	S	H	G	S	I NPl	NPA	F	V	A	Y	W
HbXIP1;1	289	31.23	6.36	6	6	6	Cyto	V	V	V	R	SPV	NPA	M	C	A	F	W
HbXIP1;2	296	32.13	8.06	6	7	6	Chlo	V	V	V	R	SPI	NPA	M	F	A	F	W
HbXIP1;3	276	29.63	7.50	5	6	5	Cyto	V	I	P	R	NPT	NPA	M	C	A	F	W
HbXIP1;4	276	29.50	8.31	5	6	5	Vacu	V	I	A	R	NPT	NPA	M	C	A	F	W
HbXIP2;1	305	32.28	8.61	6	6	6	Plas		V	V	R	NPV	NPA	V	C	A	F	W
HbXIP3;1	256	27.16	8.87	6	7	5	Vacu	V	V	A	R	NPL	NPA	V	C	A	F	W

^a,b,c^Representing the numbers of transmembrane helices predicted by TOPCONS, TMPRED and TMHMM, respectively; ^d^Best possible subcellular localization prediction by the WoLF PSORT. (*Ar/R* aromatic/arginine, *Chlo* chloroplast, *Cyto* cytosol, *ER* endoplasmic reticulum, *H2* transmembrane helix 2, *H5* transmembrane helix 5, *LE* loop E, *Loc* subcellular localization, *NPA* Asn-Pro-Ala, *Plas* plasma membrane, *TM* transmembrane helix, *Vacu* vacuolar membrane)

**Table 3:**
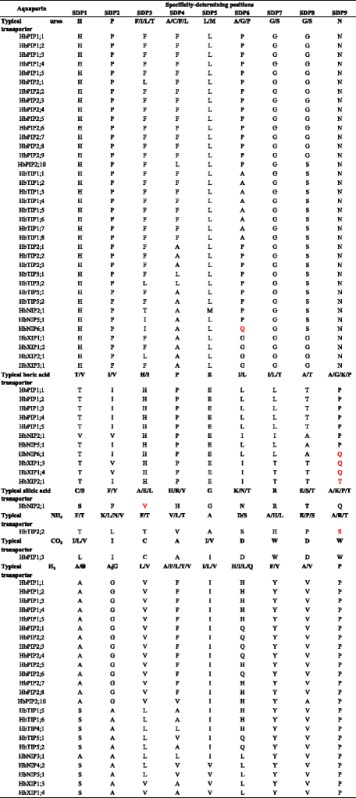
Summary of typical SDPs and those identified in the HbAQPs^a^

^a^The SDPs (specificity-determining positions) in rubber tree aquaporins differing from typical SDPs determined in this study are highlighted in red

### HbPIP subfamily

All HbPIPs were identified to have similar sequence length, however, HbPIP2s (278–288 residues) can be distinguished from HbPIP1s (269–287 residues) by harboring relatively shorter N-terminal and longer C-terminal sequences (Additional file 5). The five HbPIP1s have sequence similarities of 79.2–99.3 %, whereas the similarity percents of ten HbPIP2s are 77.4–98.3 %. Between HbPIP1 and HbPIP2 members, sequence similarities of 59.1–65.9 % are observed (Additional file 4). The dual NPA motifs, ar/R filter (F-H-T-R), and four out of five Froger’s positions are highly conserved in HbPIPs (Table 2). In contrast, the P1 position is more variable with the appearance of an E, Q or M residue (Table 2). In addition, two phosphorylation sites corresponding to S115 and S274 in SoPIP2;1 [13] are invariable in HbPIP2s, and the former one is even highly conserved in all HbPIPs, HbTIPs and HbXIPs except for the S → T substitution in several members (Additional file 5), implying their regulation by phosphorylation.

### HbTIP subfamily

HbTIPs consist of 227–257 residues. Those belonging to HbTIP1s (227–253 residues) share 73.1–98.0 % sequence similarities, whereas HbTIP2s (248–250 residues) have sequence similarities of 83.2–98.4 % (Table 2). Members of the HbTIP1 subgroup exhibit sequence similarities of 56.1–76.6 %, 59.1–70.6 %, 52.7–67.3 % and 49.8–64.3 % with subgroups HbTIP2, HbTIP3, HbTIP4 and HbTIP5, respectively. HbTIP2s share sequence similarities of 61.1–68.9 %, 59.6–65.1 % and 63.1–68.4 % with HbTIP3s, HbTIP4 and HbTIP5s, respectively. HbTIP3s share sequence similarities of 61.1–63.8 % and 58.0–60.5 % with HbTIP4 and HbTIP5s, respectively (Additional file 4). And HbTIP4 shares 59.0 % and 60.2 % sequence similarities with HbTIP5;1 and HbTIP5;2, respectively. Dual NPA motifs and P3, P4 and P5 positions are highly conserved in HbTIPs (Table 2). Residue substitution is observed at the P1 and P2 positions: T is replaced by A in HbTIP2;4 or I in HbTIP5s at the P1 position, and S is replaced by A in HbTIP3s and HbTIP5s (Table 2). Of the ar/R filter, H at H2 and I at H5 positions are replaced by N and V in HbTIP5s, respectively; A is found to be conserved in HbTIP1s, HbTIP3s, and HbTIP4 and G in HbTIP2s and HbTIP5s at the LE1 position, respectively; and residues at the LE2 position are more variable, mainly V, R, S or C (Table 2).

### HbNIP subfamily

HbNIPs consist of 267–305 residues (Table 2). With the exception of HbNIP1 and HbNIP4 subgroups that contain two members, each of the other five subgroups harbors a single member. HbNIP1;1 shares the highest sequence similarity of 90.6 % with HbNIP1;2, whereas HbNIP4;1 shares a similarity of 69.4 % with HbNIP4;2. HbNIP1s show sequence similarities of 54.5–55.7 %, 56.5–57.0 %, 59.9–63.8 %, 51.8 %, 50.0–50.6 % and 47.0 % within the subgroups of HbNIP2, HbNIP3, HbNIP4, HbNIP5, HbNIP6 and HbNIP7, respectively. HbNIP2 shows 54.8 %, 52.2–53.7 %, 49.1 %, 48.0 % and 45.4 % sequence similarities with HbNIP3, HbNIP4s, HbNIP5, HbNIP6 and HbNIP7, respectively. The HbNIP3 shows 52.1–55.0 %, 48.4 %, 47.3 % and 45.2 % sequence similarities with HbNIP4s, HbNIP5, HbNIP6 and HbNIP7, respectively. HbNIP4s show 45.1–47.2 %, 44.6–47.7 % and 44.7–46.3 % sequence similarities with HbNIP5, HbNIP6 and HbNIP7, respectively. HbNIP5 shows 75.1 % and 46.4 % sequence similarities with HbNIP6 and HbNIP7, respectively. HbNIP6 shows 46.3 % sequence similarity with HbNIP7. The HbNIPs have typical dual NPA motifs except for HbNIP4;1, HbNIP5;1 and HbNIP6;1 (Table 2). A is replaced by V in the second NPA motif of HbNIP4;1 and by S or V in the first or second NPA motif of HbNIP5;1 and HbNIP6;1. Compared with other subfamilies, HbNIPs are highly variable in the ar/R filter and Froger’s positions: W/G/A/T at the H2 position, F/L/V/I at the H5 position, A/G at the LE1 position, F/L/Y at the P1 position, S/T at the P2 position, F/Y at the P4 position and L/I at the P5 position (Table 2). In addition, one CDPK phosphorylation site corresponding to S262 in GmNOD26 [39] was also found in the C-terminus of most HbNIPs (Additional file 5).

### HbSIP subfamily

There are only four members in the HbSIP subfamily. Three HbSIP1s consist of 239 residues, while HbSIP2;1 has 240 residues (Table 2). HbSIP2;1 shares sequence similarities of 40.6–43.0 % with HbSIP1s. Within the HbSIP1 subgroup, HbSIP1;2 shares the highest sequence similarity of 95.0 % with HbSIP1;3, whereas HbSIP1;1 shows the lowest similarity of 70.0 % with HbSIP1;2 (Additional file 4). The three HbSIP1s harbor the same NPT/NPA motifs, ar/R filter (A/FV-V-P-N) and Froger’s positions (M-A-A-Y-W), whereas HbSIP2;1 exhibits NPL/NPA motifs, S-H-G-S ar/R filter and F-V-A-Y-W Froger’s positions (Table 2).

### HbXIP subfamily

HbXIPs vary from 256 to 305 residues in length (Table 2). HbXIP1s share sequence similarities of 45.3–50.3 % and 45.5–47.4 % with HbXIP2;1 and HbXIP3;1, respectively, whereas HbXIP2;1 shows 66.6 % sequence similarity with HbXIP3;1. Within the HbXIP1 subgroup, HbXIP1;4 shares the highest sequence similarity of 93.1 % with HbXIP1;3, whereas HbXIP1;2 shows the lowest similarity of 55.3 % with HbXIP1;3 (Additional file 4). In HbXIP1s, the second NPA motif and the LE2, P3, P4 and P5 positions are highly conserved. SPV/SPI/NPT/NPV/NPL in the first NPA motif, V/I at the H2 position, I/V at the H5 position, V/P/A at the LE1 position, M/V at the P1 position and F/C at the P2 position were observed (Table 2). Similar to most XIPs [7], two highly conserved C residues in the LGGC motif of LC and the NPARC motif of LE were also found in HbXIPs except for HbXIP1;2, in which the F residue is located at the corresponding position of LE (Additional file 5).

### Transcriptional profiles of HbAQP genes in the laticifer and their response to ethephon stimulation

**Fig. 3:**
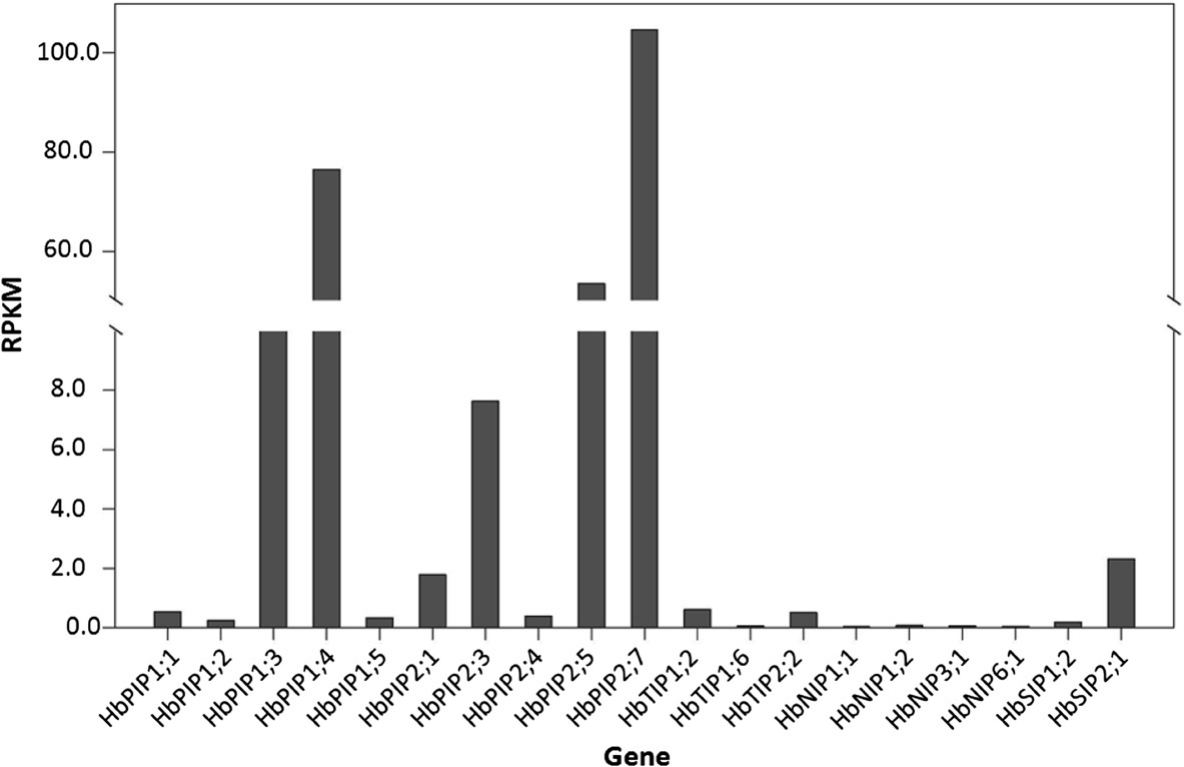
Expression profiles of 19 HbAQP genes detected in the laticifer based on Illumina sequencing

The rubber tree laticifer is a single-cell-type tissue specifically for natural rubber biosynthesis. To identify the AQP genes expressed in the laticifer and determine the most important members in the laticifer water balance, the latex representing the laticifer cytoplasm was collected and high-quality total RNAs (260/280 value between 1.95 and 2.00, 28S/18S value between 3.0 and 3.2 and RIN value between 8.9 and 9.1) were isolated from three biological replicates, respectively. Then, RNAs were pooled and subjected to Illumina RNA sequencing. Approximate 5.49 gigabase pairs of raw data (100 nt paired-end reads) were generated. After cleaning and quality checks, about 44.5 million high-quality clean reads with an average length of 95 nt were retained and assembled into 74,102 Unigenes longer than 200 bp, with an average length of 775 bp and an N50 of 1260 bp (i.e. 50 % of the assembled bases were incorporated into Unigenes of more 1260 bp). Expression profiling showed that 19 out of the 51 identified HbAQP genes were detected in the laticifer transcriptome, including genes coding for 10 PIPs (sort by abundance, *HbPIP2;7*, *HbPIP1;4*, *HbPIP2;5*, *HbPIP1;3*, *HbPIP2;3*, *HbPIP2;1*, *HbPIP1;1*, *HbPIP2;4*, *HbPIP1;5* and *HbPIP1;2*, the same as follows), 3 TIPs (*HbTIP1;2*, *HbTIP2;2* and *HbTIP1;6*), 4 NIPs (*HbNIP1;2*, *HbNIP3;1*, *HbNIP6;1* and *HbNIP1;1*) and 2 SIPs (*HbSIP2;1* and *HbSIP1;2*) (Fig. 3). Based on the RPKM value, the total expression level of PIP members was 1306, 225, 104 folds more than the NIP, TIP or SIP members, respectively, indicating a crucial role of the PIP subfamily in the laticifer water balance. Among ten laticifer-expressed PIP genes, *HbPIP2;7*, *HbPIP1;4*, *HbPIP2;5*, *HbPIP1;3* and *HbPIP2;3* were considerably more abundant, counting about 418-, 306-, 204-, 30- and 15-folds higher than the well-studied *HbPIP1;1*. Whereas, *HbSIP2;1*, the sixth laticifer-abundant AQP gene, expressed relatively more than any other non-PIP members (Fig. 3).

**Fig. 4:**
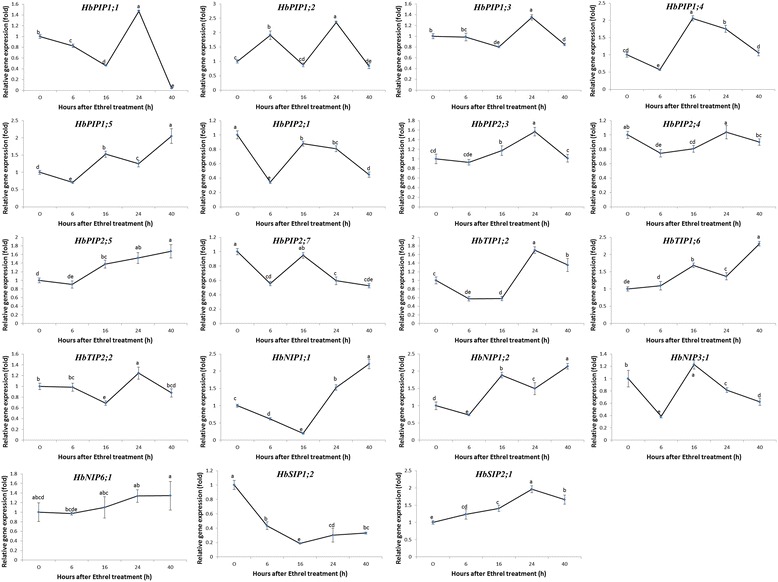
qRT-PCR analysis of 19 laticifer-expressed HbAQP genes upon ethephon stimulation. Data are mean ± SD (*n* = 9). Different letters mean significant difference over the time course

Given the important role and wide application of ethephon stimulation on rubber yield promotion, the response of the above 19 laticifer-expressed HbAQP genes subjected to ethephon treatment was analyzed using qRT-PCR over a time course (6–40 h). As described before, treating the rubber tree bark with ethephon was shown to induce a huge increase in latex yield, starting as early as 6 h after the treatment, and the yield increase was about 2.2-, 3.1-, 2.9- and 4.4-folds higher than the control at 6, 16, 24, and 40 h after the treatment, respectively; the TSC was significantly decreased at the time points of 24 and 40 h; and the latex flow duration was significantly prolonged from the time point of 16 h [22]. As shown in Fig. 4, except for *HbNIP6;1*, ethephon treatment had significant effects on all other tested HbAQP genes at one or more time points, implying their regulation by ethylene. At the early stage of ethephon treatment, i.e. 6 h, the transcriptional levels of 13 HbAQP genes were significantly affected, including two up-regulated (*HbPIP1;2* and *HbSIP2;1*) and eleven down-regulated (*HbPIP1;1*, *HbPIP1;4*, *HbPIP1;5*, *HbPIP2;1*, *HbPIP2;4*, *HbPIP2;7*, *HbTIP1;2*, *HbNIP1;1*, *HbNIP1;2*, *HbNIP3;1* and *HbSIP1;2*) genes. At 16 h post treatment, 16 HbAQP genes were significantly regulated, including eight up-regulated (*HbPIP1;4*, *HbPIP1;5*, *HbPIP2;3*, *HbPIP2;5*, *HbTIP1;6*, *HbNIP1;2*, *HbNIP3;1* and *HbSIP2;1*) and eight down-regulated (*HbPIP1;1*, *HbPIP1;3, HbPIP2;1*, *HbPIP2;4*, *HbTIP1;2*, *HbTIP2;2*, *HbNIP1;1* and *HbSIP1;2*) genes. At 24 h post treatment, 17 HbAQP genes were significantly regulated, including thirteen up-regulated (*HbPIP1;1*, *HbPIP1;2*, *HbPIP1;3*, *HbPIP1;4*, *HbPIP1;5*, *HbPIP2;3*, *HbPIP2;5*, *HbTIP1;2*, *HbTIP1;6*, *HbTIP2;2*, *HbNIP1;1*, *HbNIP1;2* and *HbSIP2;1*) and four down-regulated (*HbPIP2;1*, *HbPIP2;7*, *HbNIP3;1* and *HbSIP1;2*) genes. At 40 h post treatment, 14 HbAQP genes were significantly regulated, including seven up-regulated (*HbPIP1;5*, *HbPIP2;5*, *HbTIP1;2*, *HbTIP1;6*, *HbNIP1;1*, *HbNIP1;2* and *HbSIP2;1*) and seven down-regulated (*HbPIP1;1*, *HbPIP1;2*, *HbPIP1;3*, *HbPIP2;1*, *HbPIP2;7*, *HbNIP3;1* and *HbSIP1;2*) genes. Although the time points of 16 and 24 h post treatment harbored similar significantly regulated genes, the later had relatively more genes (especially PIP subfamily members) that were up-regulated. Although the expression patterns of the regulated genes were diverse, they could be classified into seven groups: the cluster 1 that includes *HbPIP2;5* was gradually increased upon ethephon stimulation; the cluster 2 including *HbPIP2;3* and *HbSIP2;1* were firstly increased and then decreased, which is like a clock; the cluster 3 including *HbTIP1;6* was firstly increased, subsequently decreased and increased at the last time point tested; the cluster 4 including *HbPIP1;2* was firstly increased, subsequently decreased, then increased and finally decreased; the cluster 5 including *HbNIP1;1* and *HbSIP1;2* were firstly decreased and then increased; the cluster 6 that includes 9 genes (i.e. *HbPIP1;1*, *HbPIP1;3*, *HbPIP1;4*, *HbPIP2;1*, *HbPIP2;4*, *HbPIP2;7*, *HbTIP1;2*, *HbTIP2;2* and *HbNIP3;1*) were firstly decreased, subsequently increased and finally decreased; the cluster 7 that includes *HbPIP1;5* and *HbNIP1;2* were firstly decreased, subsequently increased, then decreased and finally increased. At 24 h post ethephon stimulation, eight genes (i.e. *HbPIP1;1*, *HbPIP1;2*, *HbPIP1;3*, *HbPIP2;3*, *HbPIP2;4*, *HbTIP1;2*, *HbTIP2;2* and *HbSIP2;1*) exhibited the highest expression levels, whereas the highest expression of six genes (i.e. *HbPIP1;5*, *HbPIP2;5*, *HbTIP1;6*, *HbNIP1;1*, *HbNIP1;2* and *HbNIP6;1*) occurred at 40 h. Moreover, the transcript abundance of *HbPIP2;5* and *HbNIP1;1* were similar at the time points of 24 h and 40 h (Fig. 4). As describe above, *HbPIP2;5*, *HbPIP2;3* and *HbPIP1;3* were among the top 5 highly abundant AQP genes expressed in laticifers (Fig. 3). In addition, another highly abundant AQP genes (i.e. *HbPIP1;4*) was expressed most at 16 h post ethephon stimulation (Fig. 4).

## Discussion

### High abundance and diversity of HbAQPs

**Fig. 5:**
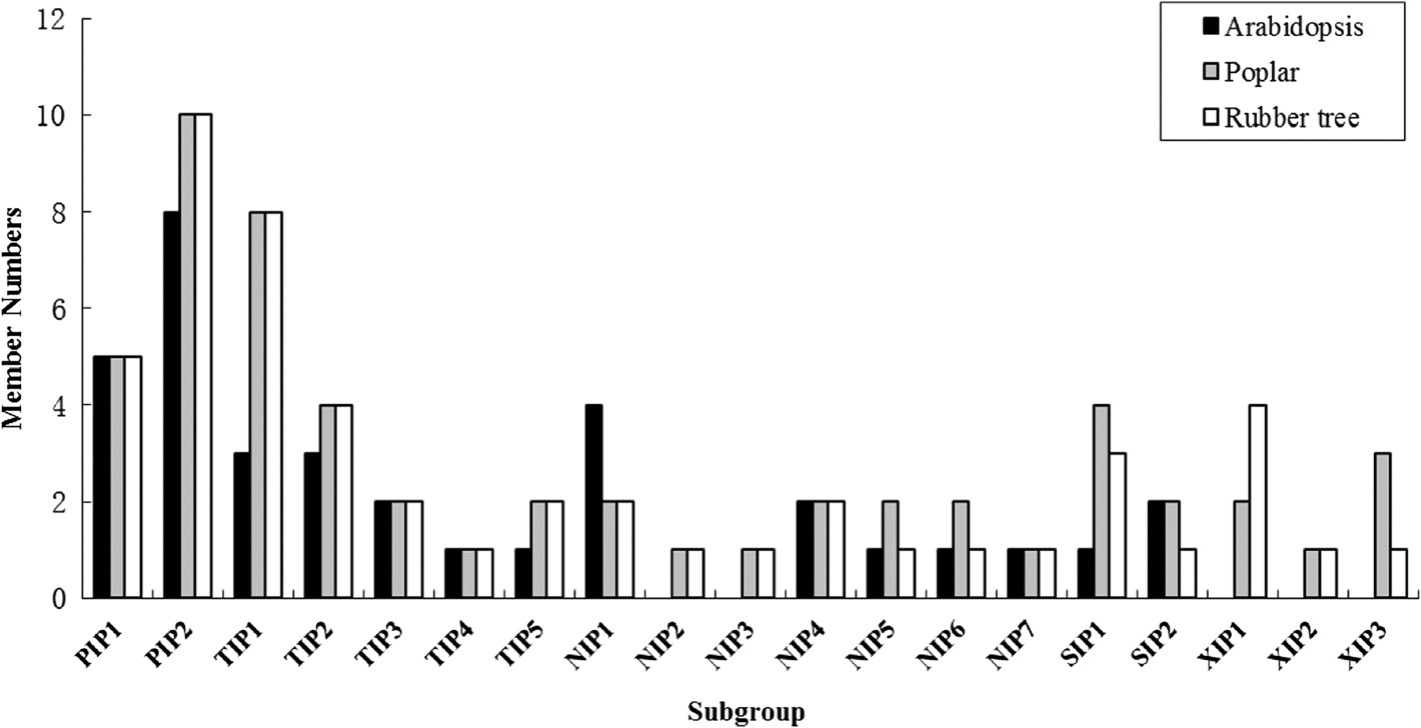
Distribution of the 51 HbAQP genes and their *Arabidopsis* and poplar homologs in subgroups

A total of 51 full-length AQP genes were identified from the rubber tree genome, which is comparable to 55 members reported in poplar (a tree species also belongs to Malpighiales) [7, 36]; more than 23 in grapevine [6], 33 in rice [5], 35 in *Arabidopsis* [3], 36 in maize [4], 41 in potato [10] and 47 in tomato [9]; less than 66 in soybean [11] and 71 in cotton [8]. Since the AQP genes in *Arabidopsis* and poplar were well characterized, their deduced proteins were added in the phylogenetic analysis of HbAQPs, which assigned them to five subfamilies. With the exception of the XIP subfamily, the further classification of HbAQP subfamilies into subgroups is consistent with *Arabidopsis*, i.e. two PIP subgroups, five TIP subgroups, seven NIP subgroups and two SIP subgroups. Nevertheless, classing AtNIP2;1 and AtNIP3;1 into the NIP1 subgroup was proposed. As shown in Fig. 1, AtNIP2;1 and AtNIP3;1 were clustered with the NIP1 subgroup, sharing the highest similarity with AtNIP1;2 in *Arabidopsis*, HbNIP1;2 or HbNIP1;1 in rubber tree, PtNIP1;2 or PtNIP1;1 in poplar, respectively. Thereby, no NIP2s and NIP3s were retained in *Arabidopsis* as seen in rubber tree and poplar (Fig. 5). Since no XIP homologs were found in the *Arabidopsis* genome, the nomenclature for poplar proposed by Lopez et al. [36] was adopted to divide HbXIPs into three subgroups. Besides supported by high bootstrap values, XIP1s are characterized by the ar/R filter of V-M-V/P/A-R, XIP2s by I-I-V-R and XIP3s by V-K-A-R.

Gene pairs were identified not only in rubber tree, but also in poplar and *Arabidopsis* (Fig. 1). For example, five AtPIP1s were clustered together apart from PIP1s of rubber tree and poplar; HbPIP1;1 and HbPIP1;2 were clustered with PtPIP1;1 and PtPIP1;2. These results suggest the occurrence of more than one gene duplication events. Previous studies indicated that poplar underwent one whole-genome triplication event (designated ‘γ’) and one doubling event, whereas *Arabidopsis* underwent the same γ event and two independent doubling events, though the *Arabidopsis* genome encodes relatively less AQP genes due to massive gene loss and chromosomal rearrangement after genome duplications [40–42]. The γ duplication occurred at approximate 117 million years ago, shortly before the origin of core eudicots [43]. As a core eudicot plant, the rubber tree appears to share the γ duplication. However, another one as the data suggested is likely to be a doubling event independent from both *Arabidopsis* and poplar, probably occurred after the divergence of Euphorbiaceae and Salicaceae. A genome-wide comparative analysis may provide more information.

### Functional inference of HbAQPs

Although plant AQPs firstly raised considerable interest for their high water permeability, when heterologously expressed in *Xenopus* oocytes or yeast cells, increasing evidence has shown that some of them are also participated in the transport of other small molecules such as glycerol, urea, boric acid, silicic acid, NH_3_, CO_2_ and H_2_O_2_ [44]. Based on atomic resolution structures and molecular dynamics stimulations of GlpF, AqpZ, AQP1 and other MIPs, several structural features determining their transport selectivity were identified, e.g. the two opposite NPA motifs, the ar/R filter and the amino acid residues at Froger’s positions for discriminating between AQPs and GLPs [14, 45]. As shown in Table 2, most HbAQPs exhibit an AqpZ-like Froger’s positions to favor the permeability of water. In contrast, HbSIP2;1 and NIP subfamily members possess mixed key residues of GlpF for P1 and P5, and AqpZ for P2–P4. The glycerol permeability of GmNOD26 and *Arabidopsis* NIPs was reported [46, 47], however, the potential glycerol transport ability of HbSIP2;1-like SIPs have not be confirmed by experimental means yet.

In addition to high permeability to water, plant PIPs were reported to transport urea, boric acid, CO_2_ and H_2_O_2_ [48]. As shown in Table 2, all HbPIPs represent the F-H-T-R ar/R filter as observed in AqpZ which harbors an extremely narrow and hydrophilic pore (diameter 2.8 Å) [45], suggesting their high water permeability. However, when expressed in *Xenopus*, extremely low water permeability of HbPIP1 members such as HbPIP1;1 and HbPIP1;4 was observed [22, 26] as seen in many other plant species [49]. Based on the SDP analysis proposed by Hove and Bhave [15], all HbPIPs represent urea-type SDPs (H-P-F/L-F/L-L-P-G-G/S-N); HbPIP1s represent boric acid-type SDPs (T-I-H-P-E-L-L-T-P); HbPIP1;3 represents CO_2_-type SDPs (I-I-C-A-I-D-W-D-W); HbPIPs except for HbPIP2;9 represent H_2_O_2_-type SDPs (A-G-V-F/V-I-H/Q-Y-V/A-P) (Table 3), supporting their similar functionality.

Although highly variable in the ar/R filter, plant TIPs were shown to transport water as efficiently as PIPs [21]. Additionally, they also allow urea, NH_3_ and H_2_O_2_ through [50]. As shown in Table 3, all HbTIPs except for HbTIP2;4 and HbTIP4;1 represent urea-type SDPs (H-P-F/L-F/L-L-A/P-G-S-N), whereas HbTIP1;5, HbTIP1;6, HbTIP4;1, HbTIP5;1 and HbTIP5;2 represent H_2_O_2_-type SDPs (S-A-L-A/L/V-I-H/Q-Y-V-P), indicating similar functionality. Compared with typical NH_3_-SDPs (F/T-K/L/N/V-F/T-V/L/T-A-D/S-A/H/L-E/P/S-A/R/T), HbTIP2;2 seems to represent novel SDPs with the substitution of S for A/R/T at SDP9.

Besides glycerol and water, plant NIPs have been found to transport urea, boric acid, silicic acid, NH_3_ and H_2_O_2_ [50–52]. As shown in Table 3 and Additional file 6, HbNIP5;1 is promised to be a urea and boric acid transporter with nine SDPs of H-P-I-A-L-P-G-S-N or T-I-H-P-E-L-L-A-P. HbNIP2;1 represent typical urea SDPs (H-P-T-A-M-P-G-S-N), and SDPs of V-V-H-P-E-I-I-A-P with the substitution of V for I at SDP2 in comparison to typical boric acid SDPs (T/V-I-H-P-E-I/L-I/L-A/T-A/G/P). Compared with typical urea and boric acid SDPs, HbNIP6;1 seems to represent novel SDPs-types with the substitution of Q for A/P at SDP6 or Q for A/P/G at SDP9. Although characterized as an NIP III member, the silicic acid transport ability of HbNIP2;1 needs to be experimentally validated since it seems to represent novel SDPs (S-F-V-H-G-N-R-T-Q in contrast to typical C/S-F/Y-A/E/L-H/R/Y-G-K/N/T-R-E/S/T-A/K/P/T) similar to that of GmNIP2;1 and GmNIP2;2 (S-Y-E-R-G-N-R-T-P) [53]. Although GmNOD26 was reported to transport NH_3_ [54], whether its close rubber tree homologs (i.e. HbNIP1;1, HbNIP1;2, HbNIP3;1, HbNIP4;1 and HbNIP4;2) represent novel SDPs-types still needs to be tested. HbNIP3;1, HbNIP4;2 and HbNIP5;1 represent H_2_O_2_-type SDPs (A/S-A-L-L/V-I/V-L-Y-V-P) slightly different from AtNIP1;2 (S-A-L-L-V-L-Y-V-P) [50].

As a recently identified AQP subfamily, plant XIPs were shown to transport water, glycerol, urea, boric acid and H_2_O_2_ [36, 55]. According to phylogenetic relationships, XIPs are split into two independent clusters termed XIP-A and XIP-B, where XIP-A includes only XIP1 subgroup and XIP-B contains at least four subgroups, i.e. XIP2, XIP3, XIP4, and XIP5 [36]. Consistent with poplar XIPs (two XIP1, one XIP2 and three XIP3), six HbXIPs can be assigned to subgroups XIP1 (4), XIP2 (1) and XIP3 (1) (Fig. 4). When expressed in *Xenopus* oocytes, PtXIP2;1 and PtXIP3;3 transported water while other PtXIPs did not. Although the mechanism why PtXIP1s, PtXIP3;1 and PtXIP3;2 do not transport water is still unclear, the close homologs of PtXIP1s in *Nicotiana tabacum* and potato were also reported to have undetectable water permeability. In contrast, Solanaceae XIPs showed high permeability to glycerol [55]. Therefore, although exhibiting an AqpZ-like Froger’s positions, all HbXIPs maybe transport glycerol. Meanwhile, HbXIP2;1 and HbXIP3;3 are probably capable of transporting water. As shown in Table 3, HbXIP1;1, HbXIP1;2, HbXIP2;1 and HbXIP3;1 are promised to be urea transporters with nine SDPs of H-P-F/L-A-L-G-G-G-N; HbXIP1;3, HbXIP1;4 and HbXIP2;1 may represent novel boric acid SDPs-types with the substitution of Q or T for A/G/K/P at SDP9; HbXIP1;3 and HbXIP1;4 harbor H_2_O_2_-type SDPs (A-G-L-V-L-H-Y-V-P) with a slight difference from some Solanaceae XIPs (S-A-V-A-V-L-Y-V-P) [55].

### A crucial role of HbPIPs in the water balance of laticifers

As a unique site for rubber biosynthesis, the laticifers are present in a wide variety of rubber tree tissues, including shoots, roots, stems, leaves, flowers, fruits, cotyledons, inner seed coats, etc., and can be divided into primary and secondary laticifers according to their origin [16]. Compared with the procambium-derivation of primary laticifers, the secondary laticifers, mainly located in the soft inner bark of the rubber tree trunk, are periodically differentiated from the vascular cambium and serve as a sole source for the commercial latex [56]. During the differentiation and maturation process, laticifer mother cells articulate with each other and further anastomose together into a successive vertical network (called rings or mantles) arranged as concentric sheaths in the secondary phloem [56]. Unlike other cells such as neighboring parenchyma cells, the mature laticiferous cells are totally devoid of plasmodesmata [25], and thus its water exchanges with surrounding cells are mainly governed by AQPs. Upon bark tapping, the laticifer cytoplasm is expelled in the form of latex due to the high turgor pressure inside [57]. Generally, latex flow can continue for several hours until coagulation processes lead to the plugging of severed laticifers [58]. During the latex flow, a progressive decrease in DRC was observed [21–23], indicating rapid water influx and latex dilution inside laticifers caused by the activity of HbAQPs. Given that of HbPIPs and HbTIPs account for more than 62.7 % of the total HbAQPs and their AqpZ-like Froger’s positions favoring the high water permeability, we initially prospect that these two subfamilies may play important roles in the laticifer water balance: the plasma membrane-targeted HbPIPs facilitate the water transport from the extracellular space to the laticifer cytoplasm, whereas the lutoid-targeted HbTIPs play an essential role in maintaining the cell osmotic balance as observed in most plant cells [59]. However, in contrast to the mature plant cells characterized by a large central vacuole which occupies 80 % or more of the intracellular space, the lutoids in laticifers are polydispersed microvacuoles occupying only 12 % of the total latex [60], arguing the central role of HbTIPs in the laticifer water balance, though their potential role in the lutoid stability and latex vessel plugging should be noted. To address this issue, the transcriptome of such a single-cell-type tissue was deeply sequenced. Results showed that PIP members were the main AQP genes expressed in the laticifer (similar results were also observed when the recently available laticifer transcriptome of clone RRIM928 was analyzed, see Additional file 7), suggesting their crucial role, especially the highly abundant *HbPIP2;7*, *HbPIP1;4* and *HbPIP2;5*, in the laticifer water balance. When expressed in *Xenopus* oocytes, our previous study showed that HbPIP2;5 could transport water as efficiently as HbPIP2;1 [21, 23]; in contrast, HbPIP1;4 and HbPIP2;7 were shown to be less efficient [22]. In addition, as a PIP1 member, the poor efficiency of HbPIP1;1 was also observed [26]. Therefore, the exact role of *HbPIP2;7*, *HbPIP1;4* and *HbPIP2;5* in the water balance of rubber tree laticifers needs further investigations.

To profile the AQP genes in response to ethylene stimulation in laticifers, the latex at different time points after ethephon treatment was collected from rubber tree clone PR107. Similar to PB217, PR107 clone is characterized as a relatively late mature variety which has a high TSC, short latex flow duration and low latex metabolism, however, ethephon stimulation could significantly prolong its latex flow duration and enhance latex yield [22, 23]. Our qRT-PCR analysis showed that the expression levels of most laticifer-expressed genes significantly changed at least one tested time point after ethephon application (Fig. 4), indicating their involvements in the ethephon enhanced water influx into laticifers. Among these time points, the latex collected at 24 and 40 h (especially 24 h) after ethephon treatment was shown to harbor the most abundant transcripts, which include four of the five highly abundant *HbPIP1;3*, *HbPIP2;3*, *HbPIP1;4* and *HbPIP2;5*, corresponding to the significantly decreased TSC, the longest latex flow duration and the highest latex yield as reported by Wang et al. who utilized the same materials [22]. Besides, similar effects of ethephon on latex yield and latex TSC of the PB217 clone were also observed by Tungngoen et al., although they used mature virgin trees as materials [21].

## Conclusions

To our knowledge, this is the first genome-wide study of the rubber tree AQP gene family and using systematic nomenclature assigned 51 HbAQPs into five subfamilies based on the sequence similarity and phylogenetic relationship with their *Arabidopsis* and poplar counterparts. Furthermore, their structural and functional properties were investigated based on the analysis of the ar/R filter, Froger’s positions and SPDs, which suggested the potentially key role of HbPIPs and HbTIPs in the laticifer water balance. Most importantly, the laticifer transcriptome was deeply sequenced to identify the most important AQPs in such a single-cell-type tissue, and qRT-PCR analysis was also performed to investigate the expression profiles of laticifer-expressed HbAQP genes upon ethephon stimulation. Our results revealed that *HbPIP*s were the mainly AQP genes expressed in the laticifer. Among 19 HbAQP genes detected in the laticifer, most of them were significantly regulated by ethylene. Consistent with the significantly decreased TSC and increased latex yield, most laticifer-expressed PIP genes were considerably induced at the time point of 24 h after ethephon application, supporting their crucial roles in the water balance of laticifers in the case of ethephon stimulation. This study provides an important genetic resource of HbAQP genes, which will be useful to improve the water use efficiency and latex yield of *Hevea*.

## Methods

### Identification of rubber tree aquaporin genes

The deduced amino acids of HbAQPs available in the NCBI GenBank were used as queries to search the available RRIM600 genome and our in-house RY7-33-97 genome for rubber tree homologs. Sequences with an E-value of less than 1e^−5^ in the tBlastn search [61] were selected for further analyses. The gene structures were firstly predicted using GeneMark.hmm [62], and the gene models were further validated with ESTs and raw RNA sequencing reads available at GenBank. The exon-intron structures of AQP genes detected in the laticifer transcriptome were also confirmed by aligning the cloned cDNAs to the corresponding gene sequences. Gene structures were displayed using GSDS [63]. Homology search for nucleotides or Sanger ESTs was performed using Blastn, and sequences with an identity of more than 98 % were taken into account. RNA sequencing reads were mapped using Bowtie 2 [64] with default parameters, and mapped read number of more than one was counted as expressed. Unless specific statements, the tools used in this study were performed with default parameters.

### Sequence alignments and phylogenetic analysis

Multiple sequence alignment using deduced proteins was performed with ClustalX [65], and the unrooted phylogenetic tree was constructed by the maximum likelihood method using MEGA6 [66]. The reliability of branches in the resulting tree was supported with 1,000 bootstrap resamplings. Classification of AQPs into subfamilies and subgroups was done as described before [3, 36].

### Structural features of rubber tree aquaporins

Biochemical features of HbAQPs were determined using ProtParam (http://web.expasy.org/protparam/). The subcellular localization was predicted using WoLF PSORT [67]. The transmembrane regions were detected using TOPCONS [68], TMPRED [69] and TMHMM [70]. Functional prediction was carried out based on dual NPA motifs, ar/R filters (H2, H5, LE1, LE2), Froger’s positions (P1–P5) and specificity-determining positions (SDP1–SDP9) from alignments with the structure resolved *Spinacia oleracea* PIP2;1 and functionally characterized AQPs as collected by Hove and Bhave [15].

### Plant materials and field experiments

PR107, the male parent of rubber tree clone RY7-33-97, was planted at the experimental farm of Chinese Academy of Tropical Agricultural Sciences (Danzhou, China) in 2002. Six batches of three trees with similar growth performance and latex yield were selected for this study. The trees had been tapped for 3 years on the s/2 d 3 system (tapping every 3 days with half spiral) without ethephon stimulation. For ethephon stimulation, five batches of trees were treated with 1 g of 2.5 % (*w*/*w*) ethephon in carboxyl methyl cellulose (CMC, 1 %) for 6, 16, 24, and 40 h before the sampling. The sixth batch was treated with 1 % CMC as a control.

### Latex collection and total RNA extraction

The latex was collected through tapping the bark at around 6:00 am, and samples representing three biological replicates were subjected for total RNA isolation as described by Tang et al. [71]. Briefly, the latex within the first 45 min was dropped into liquid nitrogen after discarding the first 5 drops. The frozen latex was suspended with extraction buffer (0.3 M LiCl, 0.01 M disodium salt EDTA, 10 % (W/V) SDS, 0.1 M Tris–HCl), and equal volume of water-saturated phenol/chloroform/isoamyl alcohol (PCI) (25:24:1) was added and vigorously shaken. Then, the mixture was centrifuged at 12,000 × g for 10 min at 4 °C, and the aqueous phase was collected and subjected to one more PCI and one chloroform/isoamyl alcohol (24:1) extraction. The supernatant was precipitated with 8 M LiCl solution for twice. The pellet was dissolved with H_2_O, and 3 M NaAc (pH 5.2) and absolute alcohol were added to precipitate the RNA. After washed with 75 % ethanol, the RNA was dissolved with H_2_O. The concentration and integrity of total RNA was confirmed using a 2100 Bioanalyzer (Agilent, Palo Alto, CA, USA).

For the expression analysis, the first-strand cDNA was synthesized from 2 μg of total RNA to a final 20 μL reaction mixture using PrimeScript® RT reagent kit with gDNA Eraser (Takara, Dalian, China) according to the manufacture’s instruction, and then stored at −20 °C.

For Illumina sequencing, magnetic beads with biotin-Oligo (dT) were used to isolate poly(A) mRNA according to the manufacturer’s protocol of Illumina TruSeqTM RNA sample preparation kit (Qiagen GmbH, Hilden, Germany).

### Expression analysis based on Illumina sequencing

RNA sequencing was performed as described previously [72] using Illumina HiSeq™ 2000 (Illumina Inc., San Diego, CA, USA) at Beijing Genomics Institute (Shenzhen, China). The raw data were filtered by the Illumina pipeline to remove adaptor sequences, adaptor-only reads, reads with “N” rate larger than 10 % (“N” representing ambiguous bases) and low quality reads containing more than 50 % bases with Q-value ≤ 5. Assembly of clean reads was carried out using SOAP *de novo* [73] (Luo et al. 2012). The trimmed reads were mapped to Unigenes using Bowtie 2 [64], and the RPKM (reads per kilo bases per million reads) method [74] was used for the expression annotation.

### qRT-PCR analysis

*HbYLS8*, the most stably expressed genes in response to ethephon stimulation [75], was selected as the reference gene in this study. The gene-specific primers are listed in Additional file 8, and the PCR reaction was performed using the SYBR-green Mix (Takara, Dalian, China) and the Real-time Thermal Cycler (Type 5100, Thermal Fisher Scientific Oy, Finland). All qRT-PCR assays were performed in triplicate for each biological sample. The amplification efficiency of each primer pair was estimated via melting curve analysis, and PCR products were confirmed by Sanger sequencing. The relative abundance of each transcript was estimated with the 2^∆∆Ct^ method after normalization against *HbYLS8* using PikoReal2.0 software unless otherwise specified. Statistical analyses were executed using the Data Processing System software v11.0. The differences among means were tested following Duncan’s one-way multiple-range post hoc ANOVA (*P* < 0.05).

## Additional files

Additional file 1:
**The gene models for the 51 HbAQP genes identified in this study.** (PDF 447 kb) Additional file 2:
**List of the Phytozome accession numbers of the AQP genes identified in** ***Arabidopsis*** **(35) and poplar (55).** (XLS 62 kb) Additional file 3:
**Percent identity of the identified HbAQP gene pairs at the nucleotide and amino acid levels.** (XLS 28 kb) Additional file 4:
**Percent similarity within and between five subfamilies of the HbAQPs.** (XLS 37 kb) Additional file 5:
**Alignment of predicted amino acid sequences of rubber tree aquaporins with structure determined Spinach SoPIP2;1.** (PDF 275 kb) Additional file 6:
**SDP analysis of the HbAQPs based on the sequence alignment with AQPs transporting non-aqua substrates.** (PDF 165 kb) Additional file 7:
**Expression profiles of the 51 HbAQP genes in the laticifer of rubber tree clone RRIM928.** (PDF 36 kb) Additional file 8:
**qRT-PCR primers of the 19 HbAQP genes expressed in laticifers.** (XLS 11 kb)

## Abbreviations

AQP: Aquaporin
Ar/R: Aromatic/arginine
DRC: Dry rubber content
EST: Expressed sequence tag
GLP: Aquaglyceroporin
MIP: Major intrinsic protein
NIP: NOD26-like intrinsic protein
NPA: Asparagine-proline-alanine
ORF: Open reading frame
PIP: Plasma membrane intrinsic protein
P1-P5: Residues at P1 to P5 positions
RPKM: Reads per kilo bases per million reads
SDP: Specificity-determining position
SIP: Small basic intrinsic protein
TIP: Tonoplast intrinsic protein
TM: Transmembrane helix
TSC: Total solid content
XIP: X intrinsic protein
