# Supplementary material for: Genome-wide identification of rubber tree (Hevea brasiliensis Muell. Arg.) aquaporin genes and their response to ethephon stimulation in the laticifer, a rubber-producing tissue
Source: BMC Genomics. 2015 Nov 25;16:1001. doi: 10.1186/s12864-015-2152-6 (PMC4658816; doi:10.1186/s12864-015-2152-6)
Supplement: Additional file 1: — The gene models for the 51 HbAQP genes identified in this study. (PDF 447 kb) [file 12864_2015_2152_MOESM1_ESM.pdf]

Additional file 1: The gene models for the 51 HbAQP genes identified in this study. The coding region is marked with uppercase letters, under which is its deduced amino acids. The transcribed untranslated regions, including 5' UTR, intron and 3' UTR sequences, are marked with lowercase letters. The start and stop codons are blacked.

1. ***HbPIP1;1***

1 cacttatcaaaatcaaacggccattaacaagcgtctgatcatcaatttcagtgtattggc

1 M E G K E E D V R L G A

61 tttggtctttttggagctaaacagct**ATG**GAGGGCAAGGAAGAAGATGTTAGATTGGGAG

13 N K Y R E T Q P I G T S A Q S Q D D K D

121 CTAACAAATATAGGGAGACGCAGCCCATTGGTACGTCAGCTCAGAGCCAAGATGACAAGG

33 Y T E P P A A P L F E P T E L T S W S F

181 ACTACACTGAACCACCAGCAGCGCCGCTGTTCGAGCCAACGGAGCTCACTTCTTGGTCCT

53 Y R A G I A E F I A T F L F L Y I S V L

241 TTTACAGGGCTGGTATTGCAGAGTTCATAGCCACTTTCTTGTTCTTATACATCTCTGTTT

73 T V M G V V K A P T K C S T V G I Q G I

301 TGACTGTCATGGGTGTAGTTAAGGCACCCACCAAGTGTTCGACTGTTGGGATTCAGGGGA

93 A W S F G G M I F A L V Y C T A G I S G

361 TAGCTTGGTCCTTTGGTGGCATGATCTTCGCTCTTGTCTATTGTACTGCTGGCATTTCAG

421 gttcggaaattgtatttttatctttaaacctttttttcctttctttttttcattcttga

113 G H I N P A V T

481 aactaatagcaagatactatgttgttggtggtgcagGAGGTCACATAAACCCGGCGGTGA

121 F G L F L A R K L S L T R A L Y Y M V M

541 CGTTTGGGCTGTTTTTGGCAAGGAAACTGTCCTTGACAAGGGCTTTGTACTACATGGTGA

141 Q C L G A I C G A G V V K G F E G R H Q

601 TGCAGTGCCTTGGAGCCATATGTGGTGCTGGTGTAGTGAAAGGATTTGAAGGGCGTCACC

161 Y T L L G G G A N S V N P G Y T K G D G

661 AGTATACTTTGTTGGGTGGTGGTGCCAATAGTGTGAACCCAGGTTACACCAAAGGTGATG

181 L G A E I V G T F V L V Y T V F S A T D

721 GGCTTGGTGCTGAAATTGTTGGCACCTTTGTTCTTGTCTACACTGTCTTCTCTGCTACTG

201 A K R N A R D S H V P

781 ATGCCAAACGTAATGCCAGAGACTCCCATGTTCCTgtaagctatagtctatagcccttcc

841 tcttgaattaatctctctatcaaaataaatggtaaaacaataatttaatgttcatgttgg

212 I L A P L P I G F A V F L V H L

901 aaatttatggtcagATTTTGGCACCTTTGCCAATTGGGTTCGCTGTGTTCTTGGTGCACT

228 A T I P I T G T G I N P A R S L G A A I

961 TGGCTACCATCCCAATTACAGGAACTGGTATCAACCCAGCTCGTAGTCTTGGTGCAGCAA

248 I F N K D K G W D D H

1021 TCATCTTCAACAAGGACAAGGGCTGGGATGATCACgtaagtcattaaacttctcaatact

1081 ttctttctcaaattctttaccagtgcacggcccattatttacttctacattgggtttgcc

259 W I F W V G P F I G A A L A A

1141 ttctggtgcatgttcagTGGATTTTCTGGGTGGGTCCATTCATTGGAGCAGCACTTGCAG

274 L Y H Q V V I R A I P F K K *
1201 CTCTATACCACCAAGTTGTGATCAGAGCCATCCCTTTCAAGAAG**TGA**tcatgatgcaaat
1261 gtttatggccatttcttgttttcaaaatcaagaccaagatcgagctcagtttgttagctt
1321 gtttatctgtttcattactgtgtacttttaaccatatgagcaccctgtaataatagcact
1381 atgttttccttgtcaaagagccttcttctaccttcctttttatttgtggaagggaaggaa
1441 agtgggccatgtctgatgagcccatcttaagttaggtgggctgtatgtattcctatgatg
1501 gtatttatgtgaatggttgtgaatttctggaatgtcattttctttggctccgaccaaaaa
1561 cctacttcttagctcatgctctaaaagagtacaataattgatagtgccaagttgtccttt
1621 ttccccgtttatcttgtaattattaatgttgtgtgaggtgggataaggcagcttttgatg
1681 atttggctttgcttccttccatttttttcccttcccctctctagaatattgaatttcttt
1741 agtttcttactctagcttggagataagctttattattgcaactaatctacgagataatat
1801 acaaaaattcct

2. *HbPIP1;2*

1 actgataaaaaatgaacggtcactaacaagcctttgatcaagtttcagtatcttggtgtt
1 M E G K E E D V R L G A N K
61 tcttgagcttaaacagct**ATG**GAGGGCAAGGAAGAAGATGTTAGATTGGGAGCTAACAAA
15 Y R E T Q P I G T A A Q S L D D K D Y T
121 TATAGGGAGACGCAGCCCATTGGTACGGCGGCTCAGAGCCTAGATGACAAGGATTATACT
35 E P P P A P L F E P T E L T S W S F Y R
181 GAGCCGCCACCGGCGCCGCTATTTGAGCCGACTGAGCTCACTTCTTGGTCCTTTTACAGG
55 A G I A E F I A T F L F L Y I S I L T V
241 GCTGGTATTGCAGAGTTCATAGCCACTTTCTTGTTCTTGTACATCTCTATTTTAACTGTG
75 M G V V K A P T K C S T V G I Q G I A W
301 ATGGGTGTGGTTAAGGCACCCACGAAGTGTTCAACTGTTGGGATTCAAGGGATCGCTTGG
95 A F G G M I F A L V Y C T A G I S G
361 GCCTTTGGTGGCATGATCTTCGCTCTTGTATACTGTACTGCTGGCATTTCAGgttcggat
421 attttacttaaacatctctaaagtcgaatatatataattttttttttctttcattcctga
113 G H I N P A V
481 tgctaatagttagatactacggtggctgtggtgatgcagGAGGTCACATAAACCCGGCGG
120 T F G L F L A R K L S L T R A L Y Y M V
541 TGACGTTTGGGCTGTTTTTGGCAAGGAAACTGTCCTTGACGAGGGCTTTGTACTACATGG
140 M Q C L G A I C G A G V V K G F E G R H
601 TGATGCAGTGCCTTGGAGCCATATGTGGTGCTGGTGTGGTGAAAGGATTTGAAGGACGTC
160 Q Y T L L G G G A N S V N P G Y T K G D
661 ACCAGTATACTTTGTTGGGTGGTGGTGCCAATAGTGTGAACCCAGGTTACACCAAAGGTG
180 G L G A E I V G T F V L V Y T V F S A T
721 ATGGGCTTGGTGCTGAAATTGTTGGCACCTTTGTTCTTGTCTACACTGTCTTCTCCGCCA
200 D A K R N A R D S H V P
781 CTGATGCCAAACGTAATGCCAGAGACTCCCATGTTCCTgtaagctatagcctatagccct
841 tcttctttaattaatggctctctcaactaaattgttaaacaagaaattatttaatgatca
212 I L A P L P I G F A V F L V
901 tggtggaaatttatggtcagATTTTGGCACCTTTGCCAATTGGGTTCGCTGTGTTCTTGG
226 H L A T I P I T G T G I N P A R S L G A
961 TGCACTTGGCTACTATCCCAATTACAGGAACTGGTATCAACCCAGCTCGCAGTCTTGGCG

246 A I I F N K D K G W D D H
1021 CAGCAATCATCTTCAACAAGGACAAGGGCTGGGATGATCATgtaagtctttaaactccca
1081 actttctttctcaaagtcctactagaattgacttattttctatattcggtgtgctgtgtg
259 W I F W V G P F I G A A L A A L Y H Q
1141 cagTGGATTTTCTGGGTGGGTCCATTCATTGGAGCAGCACTTGCAGCTCTGTACCACCAA
278 V V I R A I P F K K *
1201 GTTGTGATCAGAGCCATTCCTTTCAAGAAG**TGA**tcatatgatgcatatatgtggccgttt
1261 gattatgttttcccgatcaagatcaagatcaagatgaagctcagtttgtttgcattttct
1321 atgttatattactttgtatttgtacctatatgagcacatataataatagtattatgtttc
1381 ctaccaagggccttcttctaccttcttcctttttttttatttgtggaagggaagaggtgg
1441 gtgacgagtgctgggcccatgttaagtaggtgggctgtatgtattcatatgataatgttt
1501 atgtgaatagttctgaattgtgaaatgtcattttcttgtcagccgaccgaaaacctactc
1561 tcttac

3. ***HbPIP1;3***

1 attgggttaagggtcaacaattaatgacacttagctatctataaaatgcccttatcttct
61 actcccttacaggctctgaagcttttacttgccttttcagtgtgctttaactctacttag
1 M E G
121 agagggtcaagaatccatctttttttctgtgggagagagagacggatagagca**ATG**GAGGG
4 K E E D V R L G A N K F T E R Q P I G T
181 CAAGGAAGAGGATGTTAGACTAGGAGCAAACAAATTCACAGAGAGGCAGCCCATAGGGAC
24 S A Q S D K D Y K E P P P A P L F E P G
241 ATCAGCCCAGTCTGATAAGGACTACAAGGAGCCACCACCAGCTCCCCTGTTTGAGCCAGG
44 E L C S W S F Y R A G I A E F I A T F L
301 TGAGCTATGCTCATGGTCCTTCTACAGGGCTGGGATTGCCGAGTTCATCGCTACTTTCTT
64 F L Y I T V L T V M G F S K S T N K C A
361 GTTCCTCTACATCACCGTATTGACTGTCATGGGTTTCTCTAAGTCCACCAACAAGTGTGC
84 T V G T Q G I A W A F G G M I F A L V Y
421 CACTGTAGGTACCCAGGGTATTGCTTGGGCCTTTGGTGGTATGATCTTTGCCCTTGTCTA
104 C T A G I S G
481 CTGCACTGCTGGTATCTCAGgtaacaaaatgccgccttcattctttttgtcctggggttt
541 agctttgtttcgtttcacatctctctctctctctctctctctctctctctctctcagt
601 tttgaggtataacttttgtgggtatttcttggatttgcatctttatgcaaaaattagcct
661 tctttttatgtattggatagggggtttgagttttgtgcttctttctcgaaccagttact
721 ttttatcatagtttacactgcttaataacactctgatcaggaacattactaaccagatct
111 G H I N P A V T F G L F L
781 acatgtttgctgcaacaacagGTGGACACATCAACCCAGCAGTGACCTTTGGTCTGTTTC
124 A R K L S L T R A L F Y I I M Q C L G A
841 TGGCAAGAAAGCTCTCCTTGACAAGGGCTCTGTTCTACATCATCATGCAATGCCTTGGTG
144 I C G A G V V K G F E G D R V Y E T L G
901 CCATCTGTGGCGCTGGGGTGGTGAAAGGTTTTGAGGGAGATCGTGTATATGAGACTTTGG
164 G G A N V V A H G Y T K G D G L G A E I
961 GTGGTGGAGCCAACGTTGTGGCTCATGGCTATACTAAGGGTGATGGTCTTGGTGCTGAGA
184 V G T F V L V Y T V F S A T D A K R N A

1021 TTGTCGGCACCTTTGTTCTTGTCTACACTGTCTTCTCTGCCACCGATGCAAAGAGGAACG

204 R D S H V P

1081 CCAGAGATTCTCATGTCCCTgtatgtctttttattctcctcttatatttggcttttata

1141 attatattctgtggcggcttgattctttgtggtatacgtgtctgataattttagtataaa

1201 ttatgtggttaaatgcattagtagcagaaattattcttagttatctgattgcttgattct

1261 ccaaattctaggctctgcaacatttgtgtgattcttaattgctgaagtaaactcagacaa

1321 ttggtttttcactcatgggttagtgaactcttgtaaactttgtatgggaagtggctgaca

1381 gccagtaatttgccattttctgctcatgaagttgatgatttcagcagcaaccttataatt

1441 ttgcatttgcattggtgatggtcgagtagtgattttggtgtagtgacctatgcttgccag

1501 gctaggatgtttgcattatctgtggcatatatatatattgaatttttttatacaagaaaat

1561 atcctacatgagttggaagtgaggcctcgtcattaattaggtggtgttgcctttgcttct

1621 tgcttgtgctcagctagaaattcttacgtgggttttggttaatcataaaacatagcagac

1681 caggtgcatgtgtaccttggctatctctattagcttcccgtgcatgacttaatggaaaat

1741 atcttgcccaaatttggtgtatgtataccaacactcgtatggagtgacatctatttgtac

1801 actgggactcaccccatgatggttttggcatacttacatcatttttggacaagaatggca

1861 caaagtcatcaatctatttaatttccatttataatttgaacaaaattaatgttatttact

1921 gtgtgctaccgcataatgaatttttttcgatgatatgatgatggtaaatggttgaatggc

1981 tgcatgtctctcattatcaaatgtgtataaatcagtttacacttttcatacatgttgtat

2041 ttggaaaactagagggcaatgcatgttctgcttttgttctgagagtggagtgttaatatt

2101 tttggttaattatgaccgtgtgagatggaagttgtgagacaatgtgcttaaatcctttct

2161 catactgaggtagctagaattcattgaaatttcaaattcttcttaaggccatctaaaatt

2221 caaacacaacatcaatgtgaaaaaaggttatgaactcagtccaatggtttcttgtacgat

2281 gatgtgtggtgttatgagtttaatttggttcagttttgaccttttgcttttcacttaagc

2341 agtatatatagagtttttaaagattctgaaaacaagagatacacaattgccaaactggtc

2401 aaaatcaaatggagggagcagctttctacgtttaaaatttttctttatgcaatgttgccc

2461 tcataagttgtgtgaacatttgatgccctttccttgagcatgccatagcttgccaaatgt

2521 ctagtccagtcatggaccatataagtgataactaatcttgaggtcacctgatatgggata

2581 gtttatgattgaactaggttggcaatttggtcttgctcattgctagaaaaggcattttgt

2641 tgattgattaatctccggtgttccttaaatctgaattgtgaagcaagtgcaataatgtgg

210 I L A P

2701 aagccatgatcggacttctctcactagaaaattctctgatcattttgcagATTTTGGCTC

214 L P I G F A V F L V H L A T I P I T G T

2761 CTCTTCCCATTGGGTTTGCTGTGTTCTTGGTTCATTTGGCCACCATCCCCATCACTGGAA

234 G I N P A R S L G A A I I F N K D H A W

2821 CTGGCATTAACCCAGCCAGGAGCCTTGGAGCTGCTATCATCTTCAACAAAGACCACGCAT

254 D D H

2881 GGGATGACCATgtaagtattttgacactagggtggattttagagcaactttctctgaatg

2941 agtagtagatgcttcactacaaatataataatttctcgtatcttgataagcagtcttta

257 W V F W V G P F I G A

3001 ttctttgtgttctacctatgttgtacagTGGGTTTTCTGGGTTGGGCCCTTCATTGGAGC

268 A L A A V Y H Q I V I R A I P F K A R A

3061 TGCCCTTGCTGCTGTGTACCACCAGATAGTCATCAGAGCTATCCCTTTCAAGGCCAGAGC

288 *

3121 T**TAA**tgtttttaccatcttattcatcagaggccgcctttgagtcttatcaaacacttctc

3181 ccttggtgttctcctcttcatttcttttgttgtgtatttttgtttctatccctccatgtg
3241 aatctggtatttggagtgttatcacgagtgtaaattatctagtgttgctgtattattgtg
3301 atacccactgaatgcagaaagcccttttatctttaatctttgctttttcctaagcaaatc
3361 ttgtc

4. *HbPIP1;4*

1 gagaggatcaacaacctttttctttctcgtgtgaggtgaggagagagagagagagagata
1 M E G K E E D V R L G A N
61 gagagagagagtgagagagagca**ATG**GAGGGCAAGGAAGAGGATGTTAGACTAGGAGCAA
14 K F T E R Q P I G T S A Q T D K D Y K E
121 ACAAATTCACAGAGAGACAACCCATAGGGACATCAGCCCAGACTGATAAGGACTACAAGG
34 P P P A P L F E P G E L S S W S F Y R A
181 AGCCACCACCTGCTCCCTTGTTTGAGCCAGGTGAGCTCAGCTCATGGTCCTTCTACAGGG
54 G I A E F I A T F L F L Y I T V L T V M
241 CTGGGATTGCCGAGTTCATCGCTACTTTCTTGTTCCTCTACATCACTGTCTTGACTGTCA
74 G F S K P N N K C T T V G T Q G I A W A
301 TGGGTTTCTCCAAGCCAAATAACAAGTGCACCACAGTGGGTACCCAAGGTATTGCTTGGG
94 F G G M I F A L V Y C T A G I S G
361 CCTTTGGTGGTATGATCTTTGCCCTTGTCTACTGCACTGCTGGTATCTCAGgtaaatctt
421 tagttttgtttcacatctgctctggttcctcctccccccatcagttttgctgtttaattt
481 tgagctaaaacttttgtgggtaaaaggatttgcatctttgtgtgaaaattagccatcttt
541 tttcttttactggttattttcttacaagagctttctttataaaaccaattacttcttata
601 atagtttacaccactccatctactatgatcaggaaccttactaaccagatctacatgttt
111 G H I N P A V T F G L F L A R
661 gctgcaacaacagGTGGACATATCAACCCAGCAGTGACCTTTGGTCTGTTTCTGGCAAGG
126 K L S L T R A L F Y M I M Q C L G A I C
721 AAGCTCTCCTTGACAAGGGCTTTGTTCTACATGATCATGCAATGCCTTGGTGCCATCTGT
146 G A G V V K G F E G N R A Y E T L G G G
781 GGTGCTGGGGTGGTAAAAGGTTTTGAGGGAAACCGTGCATATGAGACTTTGGGTGGTGGA
166 A N V V A H G Y T K G D G L G A E I V G
841 GCCAACGTTGTGGCCCATGGCTACACTAAGGGTGATGGTCTTGGTGCTGAGATTGTTGGC
186 T F V L V Y T V F S A T D A K R N A R D
901 ACCTTTGTTCTTGTCTACACTGTCTTCTCTGCCACTGATGCAAAGAGGAACGCCAGAGAC
206 S H V P
961 TCTCATGTCCCTgtatggtctcttttctccttctttgtgttatttggcttgatcatgtgg
1021 tatgtgtgtcttgtaacttggattttgcataaattacatgtggttgagctcattaatagt
1081 tgcataaattcttcttagttagctgattggttgattctccaaattcaaggctctggaaaa
1141 ttttgcgtgattcttcttaattttagaagtgaattagataagtgatttttcattcatggc
1201 ttagtgaactcctctaaattctgtactgacaatggcagacagcctgcaatttgccattaa
1261 tggtttagtagcgattttggtgtagtgacccctacatgctatgatgtgagcattttcttt
1321 ggcatgaatgatttgtttctgttctgctgcttcaattttagttataagagaatatctcgg
1381 atgatatagccaagattatatattatcaaggatgtgatcctttatagcagaaccattata
1441 gccaagattatatattatcaaggatgtgatcctttatagcagaatcattatccttgatag
1501 caaaacaagaatttattgaaatttaaagtgcttatttctcaattgcatctaagttcttgc
1561 cagatcaatgtgaaacaaggttttggactcaatagtcttgttttctagtatcatgatgtg

1621 tggtgtatgagagtttaatttggttcagttttgaccgcctactttaaccatatcactaac

1681 catatcacatctgggatatgaagaaataaatgatgaattgagatggtattttgtaatcag

1741 tgattatcttgaatatattgactatttctttatttcctgaagaatttttagattttga

1801 gatggtattttcctgactatctctttcactgctggctcttgagctcaactgatatgagtg

1861 atttatgtggtcttgctcaatactagaaaaggcatttgttggttgattaaacactacgg

1921 ttttcctttaaccagaattgttaaacaagtaaaataatattggggcactggcactgctct

210 I L A P L P I G F

1981 cactggataaactaactctctgatcttttgcagATTTTGGCTCCTCTTCCTATTGGGTTC

219 A V F L V H L A T I P I T G T G I N P A

2041 GCAGTGTTCTTGGTTCATTTGGCAACCATCCCCATCACTGGAACTGGCATTAACCCAGCC

239 R S L G A A I I F N K D H A W D D H

2101 AGGAGTCTTGGGGCTGCTATCATCTTCAACAAAGACCACGCATGGGATGACCATgtaagt

2161 attccgactaataggtggactgtagcactttcactgcagtaaattacaaatataataatg

257 W I F

2221 ttgtcgcttttacggggattttgattctctgttatctgactttgttgtgcagTGGATTTT

260 W V G P F I G A A L A A V Y H Q I V I R

2281 CTGGGTTGGACCCTTCATTGGAGCTGCCCTTGCTGCTGTGTACCACCAGATAGTCATTAG

280 A I P F K A R A *

2341 AGCTATCCCTTTCAAGGCCAGGGCC**TAA**tgttttagcatcacttttctcccggtgttctc

2401 ctcatcatttctttttattatgttactttggtttttgtccctccatgtgaatctgggtat

2461 tggagtgttaattatgtgtgtaaattatctagtggtgctgtattaagcattgtgatgcct

2521 attaagcattgtgatgcctattgaatgcagagattctttttacttataaccttgctctc

2581 tctcactctctctctctct

5. *HbPIP1;5*

1 gatgatgttgagaacatagtataccagggcagcccgtagaaaagagggaactgttgcgtt

61 gtgggtgcccactttgcgtgatacacattagccatgtgttgggatccacggtactgctcg

121 aagcttaatcatgcaacgcattactcatgggcggctaatccagccagcagacccaagtca

181 ccttcttccgccgaaaccacatacgcagcaccatatcctgctgaaagcaaattgaagcga

1 M Q

241 gaaaaaatggaggagagaaaaggatgtgaggttaggagcaatcaagttccaagag**ATG**CA

3 P L G T S A Q T D K D Y R D P L P A P L

301 ACCCCTAGGTACCTCAGCCCAGACAGACAAGGACTACAGGGATCCACTACCAGCACCTCT

23 F E P V E L K S W S F W R A G I P E F F

361 CTTCGAGCCTGTTGAGCTCAAGTCCTGGTCTTTCTGGAGAGCTGGAATTCCTGAGTTTTT

43 A T F F F L Y V T V L T V M C F S R S P

421 CGCCACCTTTTTTTTCCTCTATGTCACTGTATTGACCGTCATGTGCTTCAGCAGGTCACC

63 N K C A S V G V Q G I A W A F G G M I F

481 TAATAAGTGTGCCTCTGTGGGTGTCCAGGGCATAGCTTGGGCCTTTGGGGGCATGATATT

83 V L V Y C T A G I S G

541 TGTTCTTGTTTATTGCACTGCTGGTATCTCAGgtcagatctttctccctccactttgatt

601 cttgttatatctctacaatccttctcctacgtgcttgactaattaacagtatcttgcttg

94 G H I N P A V T F G L T L A

661 ctgatggattcggcagGTGGGCACATAAACCCTGCAGTAACTTTTGGTCTTACCTTGGCA

108 R K V S L T R A I F Y M V M Q C L G A I

721 AGGAAGGTGTCCCTCACCAGAGCCATATTCTACATGGTGATGCAGTGCCTTGGAGCGATC
128 F G A G I V K G F Q P T P F E T L G G G
781 TTCGGAGCAGGGATTGTGAAGGGGTTTCAGCCAACACCGTTTGAAACGCTGGGTGGTGGA
148 A N V V N P G Y S K G D G L G A E I V E
841 GCTAACGTGGTGAACCCAGGGTATTCCAAGGGCGATGGCCTTGGTGCTGAGATTGTTGAA
168 T F A L V Y T V L S A T D A K R S A R D
901 ACCTTTGCGCTTGTCTACACTGTTCTCTCTGCCACTGATGCCAAGAGAAGCGCTAGAGAC
188 S H V P
961 TCCCATGTCCCTgtaagtacctctcaattctggtagatttgcctccgcatgtcgccagag
1021 acgacatccatggtcacttctgagaatttccccaaaaagcaaatgtagagtatggctaga
1081 ttttttttttccaaattaccgaagcattttgaacatttccttgctgaaagaaaaagaaaa
1141 tggagacagaagggtgggtaaaggtggaatgaaaagctacaacaatcatctcatccttcc
1201 tatccctccccttctttttcctctcctttcccttaaaattctcttcctctgcaggaagc
1261 tagcgaggtttaattaaaagttgcaagcatctttaatttctgataagactttttggttag
192 I L A S L P I G F A V F L V H L A T I
1321 cagATACTGGCATCACTCCCTATTGGGTTCGCAGTGTTTTTGGTTCACTTGGCCACCATA
211 P I T G T G I N P A R S L G A A I V Y N
1381 CCCATTACAGGAACTGGAATCAACCCTGCTCGCAGTCTTGGAGCAGCAATCGTATACAAC
231 E D C A W Y D H
1441 GAGGACTGTGCATGGTATGATCATgttagtacgaatattgcaatcattaattgttatgtt
1501 ttttcttcaattactaatcaaatttttgcttttatttatcatgattttaaaattaagtct
239 W I F W V R P F I G A A L A A L S
1561 ttgcttcagTGGATCTTCTGGGTACGACCTTTCATTGGCGCTGCGCTTGCTGCACTATCC
256 N Q I V I R A I P F M S K A *
1621 AACCAGATAGTCATCAGAGCCATCCCATTTATGTCCAAAGCC**TGA**gaaccaactttcaca
1681 atctttttatgttctgtttattgtatagttatgttttatgtcttcttcctccctctattt
1741 tcaagttaacatctttaagagtttaaatgttaattatgtgtatgagcaactgttctaaat
1801 gcatgttcgaaacgaatccagtaccattaattaaaac

6. *HbPIP2;1*

1 ggaagtggttgggggaatcaacggtggtgttatagttggacttgagttgaatggacggtt
61 attggtggccatatgaatggtgatattgtttatggacggttttgtcgccattacccacca
121 cgtcattcccttacagtagtcaataacatggtgggtccacccgcataccatcttggagat
181 acgtgctatctcataagctactatacttgttccgcccagagtaaaacaacgtcgtattaa
241 taccagcccatggcacttcatttcttggaaaattgcatgtgtaagttggattatgcaacc
301 attcatgcggaatgttaggttaaaagttctccaatgttcgcaagaaaatgtggccataat
361 ttcttggcgattggagaatgcaagtaaaaccaatcacatggaaaggccacagcagttgcc
421 agaattgagagactattagaacttacccgaacaatctctactctttagccgaatcagaag
481 aggccaaagaagcaatcacatgaagtgtgagggtggggccacagaaagcgcaagaaacca
541 tttgttaagttagatggggcggcaaagctaagttaaattaacactcactcctctcaaaac
601 tcttcaatactcaatactcacatatcccataaatacccccctccacttccacacttccca
661 ctacaataatcaacatctcttagctcagtcgcctctttcctttctcttgccaaattctgt
1 M A K D V E V G G Q G G E F Q A K
721 cacggtacct**ATG**GCCAAGGACGTTGAAGTTGGAGGACAAGGCGGTGAGTTCCAAGCCAA
18 D Y N D P P P A P L I D A E E F T Q W S

```
 781 GGACTACAATGACCCGCCACCTGCACCACTGATCGATGCCGAGGAGTTTACTCAGTGGTC
  38  F  Y  R  A  I  I  A  E  F  I  A  T  L  L  F  L  Y  I  T  V
 841 GTTTTATAGGGCTATCATTGCTGAGTTTATAGCCACGCTTTTGTTTTTGTACATCACTGT
  58  L  T  V  I  G  Y  K  S  Q  T  D  P  A  K  N  A  D  P  C  G
 901 TTTGACTGTGATTGGTTACAAGAGCCAAACCGACCCTGCCAAGAACGCTGATCCATGTGG
  78  G  V  G  I  L  G  I  A  W  A  F  G  G  M  I  F  I  L  V  Y
 961 TGGTGTTGGGATTCTTGGTATCGCTTGGGCCTTCGGTGGAATGATCTTTATTCTTGTTTA
  98  C  T  A  G  I  S  G
1021 CTGCACTGCTGGTATCTCAGgtaaatatcttcctgtgactcctgtcttgtaaaagcttgt
1081 gttctagcgactacaaataaaaataatttttgggttctaattaaatttgttaaatttaca
105    G  H  I  N  P  A  V  T  L  G  L  F  L  A  R  K  V  S  L
1141 gGTGGGCATATAAACCCTGCTGTGACTTTGGGTCTGTTTTTGGCGAGGAAAGTGTCTCTG
124 V  R  A  I  L  Y  M  A  A  Q  C  L  G  A  I  C  G  C  G  L
1201 GTGCGAGCTATATTGTACATGGCAGCTCAGTGCTTGGGAGCCATATGTGGATGTGGGCTC
144 V  K  A  F  Q  K  A  Y  Y  N  R  Y  G  G  G  A  N  E  L  A
1261 GTGAAAGCATTCCAAAAAGCTTATTACAATAGGTATGGTGGTGGAGCCAATGAGCTTGCT
164 D  G  Y  S  K  G  T  G  L  G  A  E  I  I  G  T  F  V  L  V
1321 GATGGGTACAGCAAGGGCACTGGATTGGGTGCTGAGATCATTGGTACCTTCGTTCTTGTC
184 Y  T  V  F  S  A  T  D  P  K  R  N  A  R  D  S  H  V  P
1381 TACACTGTCTTTTCTGCTACTGATCCCAAAAGGAATGCTAGAGACTCCCATGTTCCTgta
1441 tgtgctctgtgttacttataatccctctttttaactggtaaaacatcagttgagttgaac
203                            V  L  A  P  L  P  I  G  F  A  V
1501 taattttgtgtatttttggatttgtagGTCTTGGCTCCACTTCCAATTGGATTTGCTGTG
214 F  M  V  H  L  A  T  I  P  V  T  G  T  G  I  N  P  A  R  S
1561 TTCATGGTTCATCTGGCCACAATTCCAGTCACTGGCACTGGTATTAACCCAGCTAGGAGC
234 F  G  A  A  V  I  Y  N  Q  D  K  A  W  D  D  Q
1621 TTTGGAGCTGCTGTGATCTACAACCAGGACAAGGCATGGGATGACCAAgtatgttgtttt
1681 cctctataattagttcttattacctaccatgctaggcttagccccatgaaattattagct
250                         W  I  F  W  V  G  P  F  I  G  A  A  I  A
1741 aacttaaattttttttccagTGGATTTTCTGGGTTGGACCTTTCATTGGTGCCGCAATTG
264   A  F  Y  H  Q  Y  I  L  R  A  A  A  V  K  A  L  G  S  F  R
1801 CTGCATTCTACCACCAATATATCTTGAGAGCAGCTGCTGTTAAAGCTTTAGGATCTTTCA
284   S  T  S  N  I  *
1861 GGAGCACGTCCAACATATAAttgagaaaagaaattcctttatgctactaaatttgagagt
1921 ttcatctggtccttgaggctttgggaccttggaatatgtacattaatcatgtatctttgt
1981 gtgcttgggttcatggttttatttgtgcccaaactgttgcttccactctatttcttttt
2041 cccctttgtggccttgcactcatgatgatgtagacaaatttatgagcattgtgtcaatta
2101 tatgatttcaattggaaaataaaattcttatatattaatatatgccattgatgaagagat
2161 gttgatcatattcaatagattgagagaata
```

7. *HbPIP2;2*

```
   1 caagtgcaagaaaccatttgttaagctaaatggggcggcgaagtcaagttaaattagcac
  61 tcactctcctctcaaactcattatcaattcatgggaaaattagtgaatggtactaaaaat
 121 gatctttcaaaaatcttaaatttgggttataatcgaagtcggataaaaaaaaagaaaact
 181 tccaatattcaatactcataccccataaatacccccctgccacttccacacctccaccaca
```

241 ataatttacatctcttagcaatctagctactaccagaactcctcagtcttttcttccctt
1 M A K D I E V G G D
301 tctcctgccaaattctttcagcgtatagct**ATG**GCCAAGGACATTGAAGTTGGAGGCGAT
11 G S E F R A K D Y H D P A P S P L I D A
361 GGAAGTGAGTTCCGTGCCAAGGACTACCATGACCCTGCACCGTCACCATTGATAGATGCT
31 E E L T K W S F Y R A I I A E F I A T L
421 GAGGAGTTGACCAAGTGGTCGTTTTATAGGGCTATAATTGCTGAGTTTATAGCTACCCTC
51 L F L Y I T V L T V I G Y K S Q T D P A
481 TTGTTTTTATACATCACTGTTTTGACTGTAATTGGTTACAAGAGCCAAACTGACCCTGCC
71 K N A D A C G G V G I L G I A W A F G G
541 AAGAACGCTGATGCCTGTGGTGGTGTTGGGATTCTTGGCATTGCTTGGGCCTTTGGTGGC
91 M I F I L V Y C T A G I S G
601 ATGATCTTCATTCTTGTTTACTGCACTGCTGGCATCTCAGgtaattatattcttctccta
661 aaatgcaactgtttttgctacgtttccaaagtaactgaaactaaaaatgataagggttct
105 G H I N P A V T F G L
721 aattaaatttgtgaattttttgtagGTGGGCATATAAACCCAGCTGTGACTTTTGGGTTG
116 F L A R K V S L V R A V L Y M A A Q C L
781 TTTTTGGCGAGGAAAGTGTCTCTGGTGCGAGCTGTATTGTACATGGCAGCACAGTGCCTG
136 G A I C G C G L V K A F Q K A Y Y N R Y
841 GGGGCTATATGTGGATGTGGGCTGGTCAAGGCATTCCAAAAGGCTTATTATAATAGGTAT
156 G G G A N E L A D G Y S K G T G L G A E
901 GGTGGTGGAGCCAATGAGCTCGCTGATGGTTACAGTAAAGGCACTGGATTGGGAGCTGAG
176 I I G T F V L V Y T V F S A T D P K R N
961 ATCATCGGTACTTTTGTTCTTGTCTACACTGTTTTCTCTGCTACTGATCCCAAAAGGAAT
196 A R D S H V P
1021 GCCAGAGACTCCCATGTTCCTgtatgttcttgctctatcttaaatcataatccctcttgt
1081 ttattaactggtaaaatatcagtttagtttaaacaatttttgtgtatttttggatttata
203 V L A P L P I G F A V F M V H L A T I
1141 tgtagGTCTTGGCTCCACTTCCAATTGGATTTGCTGTGTTCATGGTTCACTTGGCCACAA
222 P V T G T G I N P A R S F G A A V I Y N
1201 TTCCAGTCACTGGCACTGGCATCAACCCAGCTAGGAGCTTTGGAGCTGCTGTGATCTACA
242 Q D K A W D D Q
1261 ACCAGGACAAGGCATGGGATGACCAAgtatgttgcttttcctattatttttacttatttc
1321 ttattacctatcatgccacttaaccccaagaaatcatcttaattagaaattttttttttt
250 W I F W V G P L I G A A I A A F Y H Q
1381 ccagTGGATTTTCTGGGTTGGACCTTTGATTGGTGCTGCAATTGCTGCATTTTACCACCA
269 Y I L R A S A A K A L G S F R S N S N I
1441 GTATATCTTGAGAGCATCTGCTGCTAAAGCTCTTGGGTCATTCAGGAGCAATTCCAACAT
289 *
1501 A**TAA**ttcaggagaaaaacaagcactctatgcttctaagattgagagttttatctggtcct
1561 tgagggtttaggaccttggaatatgtacattaattttgtatctttgtgtgcttgatgctt
1621 gggcacttgcttttatctgtgcccaaaatgttgcttcccactctgttttctattgtcctc
1681 ggctaatttcctttccctttttgtggcccttgtagtcatgatgatgtttacaaatttatg
1741 agcaagtcaagtgctttgtttcatttcatgataatccctaattataaataaatcattatt

1801 cagcaagttgagagagcaaactcagttaaaaattggattagcctatggtggtggattatg
1861 aaaatgaattgatggcaatttttattcataatttgacttaaaattagtaagatgagattc
1921 acaatagtatgaaaattaaatagtatcataacacttttaccgctatctattggaaagtga
1981 attgtagaaagtattttaacataggattgagttattggctcaagataagatgataagtca
2041 ttgctgccatagacacttcacaacgatcaaagccaacacttgcatcacagggagcttaaa
2101 cgcccatcaccacacatcttatctcgacaagtaaacttccccattttctcaacaaaaacg
2161 aagaactcagctcaacaacaatgaagggggcagagctttctggtataaaaagccaatacg
2221 atgatcctcctagacatagtatagaagcagattccaaaaatgtgagtaaattagagtaat
2281 ataaactctttcaacgcatagagctaacctaatgaacaagaaaatgacaaagaccctccc
2341 ttccctttatcctaaatcatgaaaatcaaatcaagaacaactaaaggtaccctctcgttg
2401 aagatctatctacatgcaaaccaatcaaatacaaaacagtaatcatgtaggggcgtttaa
2461 agaaaccaacaaacaatcaactcgaccaacctgaataaattcaaactgaaattagtaatt
2521 ttatataattaaatatt

8. ***HbPIP2;3***

1 gccttgtactgaaaaaaattacattagatgcatttagaagagccgttgcagctaatggt
61 ggggtcaagcaagcgaaggagggaaaaaaatgaagaaaaagatggtatttggtcggctaa
121 ttaaaatttaagttaattatgtcatcaaacgatgaatattatactctcttaatcatatac
181 cgtccatcctctcttccataaataccccctctttgctcgcctctctaaccacatattctc
241 cacccccctctcagagctcactagccaccagagaagcacaaaatcaagaaaatatatagat
1 M A K D V E V A E N P G E F S A
301 caataattctctc**ATG**GCTAAGGACGTGGAAGTTGCAGAGAATCCTGGAGAATTCTCGGC
17 K D Y H D P P P A P L I D V E E L G K W
361 CAAAGACTACCATGACCCTCCACCTGCACCTTTGATCGATGTAGAGGAACTTGGAAAATG
37 S F Y R A L I A E F I A T L L F L Y I T
421 GTCCTTCTACAGGGCTCTTATTGCCGAGTTCATTGCTACCCTTCTTTTCCTTTATATCAC
57 V L T V I G Y K S Q T D P A K N S D A C
481 TGTGTTGACAGTAATTGGATATAAGAGTCAGACTGACCCTGCCAAGAATTCTGATGCTTG
77 G G V G I L G I A W A F G G M I F I L V
541 TGGTGGTGTTGGTATTCTTGGCATTGCATGGGCCTTTGGTGGCATGATCTTTATTCTTGT
97 Y C T A G I S G
601 TTACTGCACTGCTGGTATCTCTGgtcagtttcttcttcttctttttcttttcctttttt
661 tctttctctgtttggttccaaagaaaattctcttttttttttccaaagatcttgcttttt
721 gtttgtgcttaatttctttctttttctatctcacagtggaaaaaatttaaccacgtatt
781 aagaacatgctttagaaaatttaacgtgaaacacatgcaatgttttcttcatggagttgg
841 ggggaaaactgaaacaagaagaagagaaaacactttaaagactaatttaatctttatttt
901 ctttatttttttttctacaaactgtttcctaaaacatatataaccaagaataatgtctgtt
961 ttgaagaaaatattggattttactcaagcttaatttataaataacaaaatttataaccaa
1021 tttaacaaattaatggtatattattcttaggtatttatatttttttcccatccagttact
105 G H I N
1081 cgtaaagaccagtactgatgataacccttgtttgtattctacacagGAGGACATATTAAC
109 P A V T F G L F L G R K V S L I R A L L
1141 CCAGCTGTGACCTTCGGGCTGTTCCTGGGACGCAAGGTGTCACTAATCAGGGCCCTTTTG
129 Y M V A Q C L G A I C G C G L V K A F Q
1201 TACATGGTAGCACAGTGCCTGGGTGCAATATGTGGGTGTGGTTTGGTCAAGGCCTTCCAA

149 K A Y Y T R Y G G G A N E L S S G Y S K
1261 AAGGCTTATTACACCAGGTATGGAGGTGGGGCCAACGAACTCTCTTCTGGGTACAGCAAG
169 G T G L G A E I I G T F V L V Y T V F S
1321 GGCACTGGTTTGGGTGCTGAGATCATTGGTACCTTTGTTCTTGTCTATACTGTCTTCTCT
189 A T D P K R N A R D S H V P
1381 GCCACTGATCCTAAGAGGAATGCAAGAGATTCCCATGTTCCTgtaaggacctgattctgt
1441 tttctctgaattgtttgataaaatatttgtgctatttaatgtttaggttcttgggatatg
1501 acatgagtcattttcttgctgactttgctggtttttctcatctcaagatcttgatttctg
1561 gggtctttactttctccacaccacagttttgatctgaagttggttaggtttttgatttgg
1621 aaagcaagcgcaagataatataagaaattatttaaaaaaaagaaaaaaaaactcatcatt
1681 tgatttctttttataatttatgaagtgcctgttaacattaaaatgatttaattaatattt
203 V L A P L P I
1741 aattaaatttctattaatttaatttctctcccaattaagGTATTGGCACCTCTTCCCATT
210 G F A V F M V H L A T I P I T G T G I N
1801 GGATTTGCTGTGTTCATGGTTCACCTTGCCACTATTCCAATTACTGGCACTGGCATCAAC
230 P A R S F G A A V I Y N K D K A W D D Q
1861 CCTGCTAGGAGTTTTGGAGCTGCTGTTATCTACAACAAAGACAAGGCCTGGGATGATCAG
1921 gtacaaaattaacctatttaaataaaaattaatacattcttctctcatatgcttgaactt
250 W I F W V G P F I G A A I A
1981 atttctgtttgttttgcagTGGATCTTCTGGGTTGGACCTTTTATTGGAGCCGCTATTG
264 A F Y H Q Y I L R A A A I K A L G S F R
2041 CTGCTTTCTACCACCAATACATTCTGAGAGCAGCAGCCATTAAGGCTCTAGGATCCTTCA
284 S N A *
2101 GGAGCAATGCT**TAA**tttagtttttaatttatttgataatttgaagaaagatggtgatgat
2161 gcttgagaagaataacaatggatgatgatgaatgagcaagttgtcttttaagaaggggtc
2221 ccttccccatttctctcttttccctttcaagggttttgagggagaaaattggaaaagcc
2281 catggaattgtaaataataaaaatttatggtcaaggtggggtgtcttttctatccctgtt
2341 gcttttgttatgatttgtatttttaagttgtcactttttgtttatcctttgatccttatct
2401 tatgatcttcagctcctagtttttcccaatccattatcttttgggtgtatttcatcttga
2461 ttgtgcttaattaatctagtaaaatgggttttgtagtctctctttttctttatcat

9. *HbPIP2;4*

1 ataaatacccctctgtgctcgcctctccaaccgcacattctccactctctctctctctct
61 ctctctctctctctctctgagctcactagctaccagagaagcagtaaatcaagaaaagct
1 M A K D V E V A E N P G E F
121 gtagagcaataattgtctc**ATG**GCTAAGGACGTGGAAGTTGCAGAGAATCCTGGAGAGTT
15 S A K D Y H D P P P A P L I D V E E L G
181 CTCAGCCAAGGACTACCATGACCCTCCACCTGCACCTTTGATCGATGTTGAGGAACTTGG
35 K W S L Y R A L I A E F I A T L L F L Y
241 AAAATGGTCCCTCTACAGAGCTCTTATTGCAGAGTTCATAGCCACCCTTCTCTTTCTTTA
55 I T V L T V I G Y K S Q T D P L K N A D
301 TATCACTGTTTTGACAGTTATTGGATACAAGAGCCAAACTGATCCTCTCAAGAATGCTGA
75 A C G G V G I L G I A W A F G G M I F I
361 TGCTTGTGGTGGTGTTGGTATTCTTGGTATTGCTTGGGCCTTTGGTGGCATGATCTTTAT
95 L V Y C T A G I S G

```
 421 TCTCGTTTACTGCACTGCTGGTATCTCTGgtaggtttcttcttttctttctttgtttggt
 481 tccaaagaaaatttttccttttgtttttccttccaaagaacatgttttggttttgtgctt
 541 aatttcttgattttctagcacctcactacagaagaattaactttaaccacacatatatac
 601 aaagaaaatttatgaaacaagaagaggaagaacactttaaaactcttttccctatgttag
 661 tagctagtttgttatccagttatcagtaaagagcagtactgatgatgatgaacacttgtt
 105                G  H  I  N  P  A  V  T  F  G  L  F  L  G  R
 721 tgtactctacacagGAGGACACATTAACCCAGCTGTGACCTTTGGGCTTTTCTTGGGACG
 120  K  V  S  L  I  R  A  L  L  Y  M  V  A  Q  C  L  G  A  I  C
 781 CAAGGTGTCACTAATCAGGGCCCTTTTATACATGGTAGCACAGTGCTTGGGTGCAATCTG
 140  G  C  G  L  V  K  A  F  Q  K  A  Y  Y  N  R  Y  G  G  G  A
 841 TGGGTGTGGTTTAGTGAAGGCCTTCCAAAAGGCTTATTATAACAGGTATGGAGGTGGGGC
 160  N  E  L  S  D  G  Y  N  K  G  T  G  L  G  A  E  I  I  G  T
 901 CAATGAATTATCGGATGGGTACAACAAGGGAACTGGATTGGGTGCTGAGATCATTGGCAC
 180  F  V  L  V  Y  T  V  F  S  A  T  D  P  K  R  N  A  R  D  S
 961 CTTTGTTCTTGTCTACACTGTCTTCTCTGCCACTGATCCTAAGAGGAATGCAAGAGACTC
 200  H  V  P
1021 CCATGTTCCTgtaagtaactgattcttttctctcttaatttttttgatctattttgtatt
1081 attaatgttctctgtaaaggatcatttaatttccttcagttgggatatgggtcattttct
1141 tgctgacttaatttgctggtttttccatctcaagatcttgatttctgaggcctttactt
1201 tctccacacgactactactacattttcaaaatgaagtttgttgggtttttatttggaca
1261 gcaagcacaagctaactaaaaaattggaaaatacccatcatttgataccttctttataat
 203                                                    V  L  A  P
1321 ttatgaagagcatgttaaagcttaaatgattttatttcttccaatgaagGTATTGGCACC
 207  L  P  I  G  F  A  V  F  M  V  H  L  A  T  I  P  I  T  G  T
1381 TCTCCCCATTGGATTTGCTGTGTTCATGGTTCACCTTGCCACTATTCCAATTACTGGCAC
 227  G  I  N  P  A  R  S  F  G  A  A  V  I  Y  N  K  D  K  A  W
1441 TGGTATCAACCCTGCTAGGAGTTTTGGAGCTGCTGTTATTTACAACAAAGATAAGGCCTG
 247  D  D  Q
1501 GGATGATCAGgtacaaaataaaacctatttccacattcatttttttaataaaaattatta
 250                                                          W  I
1561 cattatgcttctcgtcatatactaaagaaactttttttattattattattttacagTGGAT
 252  F  W  V  G  P  F  I  G  A  A  I  A  A  L  Y  H  Q  Y  I  L
1621 CTTCTGGGTTGGACCTTTTATTGGAGCTGCCATTGCTGCTTTGTACCACCAATACATTCT
 272  R  A  A  A  I  K  A  L  G  S  F  R  S  N  A  *
1681 TAGAGCAGCAGCCATTAAGGCTCTAGGATCCTTCAGGAGCAATGCTTAAataggccttttt
1741 aattttatttgataatttgaagaaagagggtggtgatgatgatgatgatgatgcttaaga
1801 agaataacaatggatgaagatgagtggtcaagttctgtcttttaaggggttccttcacta
1861 atttctttctcaatccctttcaaggatttatagggagaaatttggagtggaattgtaaat
1921 actaagcaatatttatggtcaagggtgtcttttcaatccctgttgtttttgttatgattg
1981 tattttaagttataattatgtcacttatgtctatcctttgatccttatcttatgatctcc
2041 tctttttttttttttaatccattatcttctgggtgtatttcttcttgtttaagcataatc
2101 aatctagtaaaatgggttgtcttcg
```

10. *HbPIP2;5*

```
   1 aaaaattgtttctcaatattaaatggaaaaatgactaaataagtactttatgaaataaaa
```

61 aataaaatccttggaaacaaagaaaatgaaaaaaaaaagaaaaaaaaaatgaaaaaaaaa
121 cagcctttatttacccgctctccttcaccatttatacactcgctcagctcagcttctct
181 gtatccaaagtctcaaaaccaaaacgccaacccccctctctctccctctctatccccttat
1 M V K D V T E
241 atacaaaatcttgctgtttctctttgctgagggtcgacc**ATG**GTGAAGGACGTGACAGAA
8 Q G S F P A K D Y H D P P P A P L I D A
301 CAAGGATCATTCCCAGCGAAGGACTACCATGACCCACCACCAGCACCATTGATTGATGCG
28 V E L T K W S F Y R A L I A E F I A T L
361 GTGGAGCTAACCAAGTGGTCATTTTACAGGGCCTTGATTGCTGAATTTATAGCAACTTTG
48 L F L Y I T V L T V I G Y K S Q T D P A
421 CTCTTTCTTTACATCACTGTTTTGACTGTGATTGGATACAAAAGCCAGACTGATCCTGCA
68 K N A D S C G G V G I L G I A W A F G G
481 AAGAATGCTGACTCTTGTGGTGGTGTTGGAATTCTTGGCATCGCTTGGGCCTTTGGTGGC
88 M I F I L V Y C T A G I S G
541 ATGATCTTTATTCTTGTTTACTGCACTGCTGGTATTTCAGgtgagatatatagctttaat
601 tttcctcctctttccttctttcttccttccctagaacagttttcagagcttcaagatttc
661 tttttgtagcttcttcgaatggtaaatgtttttttacagtagtttttccattattttccca
721 cttcttcaccaaggagtaagtattctttgctcatcaagttttcttgggaagcaaacagat
781 tattagtttctttgttaattgcgtactatctttaatgaatattatgttcctttttcttaa
841 gctagatacgaaaaaggatttgccttttatacttgaaaaaagaaaaaaaaaagaaaagaaa
901 agcattttccttttaaagatttctcaagaaaagtttttgtttttagaaatcagattttc
961 ttaagaaaagttgatgagcttgtacccagacattaaacacaggcacagccttggcttttt
102 G
1021 cttcaattgttagcgtattttagcagatagttacgtgatttttcaaatctgcgcagGAGG
103 H I N P A V T F G L F L A R K V S L V R
1081 GCACATTAACCCAGCAGTGACATTCGGGTTATTCCTGGCCCGAAAGGTCTCACTGGTACG
123 A V M Y M V A Q C L G A I A G V G L V K
1141 GGCCGTCATGTACATGGTGGCCCAGTGCTTGGGAGCCATAGCCGGTGTCGGATTGGTGAA
143 A F Q S S F Y K R Y G G G A N S L A A G
1201 GGCCTTCCAGAGTTCCTTCTATAAGAGGTATGGTGGTGGGGCCAACAGTCTGGCTGCTGG
163 Y S K G V G L G A E I I G T F V L V Y T
1261 GTACAGCAAAGGCGTTGGATTGGGTGCCGAGATCATCGGGACTTTTGTTTTGGTCTACAC
183 V F S A T D P K R N A R D S H V P
1321 AGTGTTTTCCGCCACAGATCCGAAGAGGAATGCCAGAGACTCCCATGTGCCGgtatgtat
1381 cataacatttctcatagaaattaattctctgagacttttgggctataattagtaatcact
1441 aattaattgttctcataaagatttgcatgcttgtttcacatgtgctatggtatgatatca
1501 tgaaccatgtgttttgctgactttagcaaatcttaatgcctgtttatatattaatgatta
200 V L A P L P I G F A
1561 acaatttttttttatttaattgtaaaattagGTTTTGGCTCCACTCCCAATTGGATTTG
210 V F M V H L A T I P I T G T G I N P A R
1621 CTGTATTCATGGTTCACTTGGCCACCATTCCAATCACTGGAACCGGCATCAACCCAGCCA
230 S L G A A V I Y N Q D K P W D D H
1681 GGAGTCTAGGAGCTGCTGTTATCTACAATCAGGACAAGCCCTGGGATGATCATgtcagta
1741 tatataaacatattcatcaacttaattatggttattaattaaacatggtaatgggtactt

247 W I F W V G P F I G
1801 aattttcttgttaaattatttttttcttgcagTGGATCTTTTGGGTTGGACCCTTCATTG
257 A A I A A F Y H Q F I L R A G A V K A L
1861 GTGCAGCCATTGCAGCCTTCTATCACCAATTCATCTTGAGGGCAGGAGCTGTGAAGGCTC
277 G S F R S N P T V *
1921 TTGGATCATTCAGGAGCAACCCAACTGTT**TAA**gggaagaaataatttttctaaactaata
1981 atgaaggaaaagcatgtgctggttggtttcattattagcccttctggtgtgttctttgtt
2041 gttgggggggttgaaaatgtgagaagagaggtttgaaagaattatggaatttgtagatat
2101 aaaagcctctttgaaaatgggaggcttttggttatccctttttttatttgttttgtttgat
2161 ttcacttagcaatgttatgatggttttcttggttggttcatcatcatcatctattgtcta
2221 ttttttcttctttgcacttattatgcttgtaagaaaagttaggctcttttgcttttatta
2281 taagtgtaggctatgcttgttttattatgagctttaagtttcattttcacatattatgtt
2341 tccatatttttttccaaatcaagtattcatttcataa

11. *HbPIP2;6*

1 gccaatacgccaaactctctctctctctctctctgtgtattaagggaacttcttgttgtt
1 M V K D I T E Q
61 tgtcttctgagaagctgatttaagcgcaattgtcgatc**ATG**GTAAAGGACATTACAGAAC
9 G S F S A K D Y H D P P P A P L I D A V
121 AAGGATCATTCTCTGCCAAGGACTACCACGACCCACCACCAGCTCCATTGATTGATGCGG
29 E L T K W S F Y R A L I A E F I A T L L
181 TGGAGCTAACCAAGTGGTCATTTTACAGGGCTCTGATTGCTGAATTTATAGCAACTTTGC
49 F L Y I T V L T V I G Y K S Q T D P A K
241 TCTTCCTTTACATCACTGTTTTGACTGTAATTGGCTATAAGAGCCAGACTGATCCTGCCA
69 T H D A C G G V G I L G I A W A F G G M
301 AGACTCATGACGCTTGTGGTGGTGTTGGAATTCTTGGCATCGCTTGGGCTTTCGGTGGCA
89 I F I L V Y C T A G I S G
361 TGATCTTTATTCTTGTTTACTGCACTGCTGGTATTTCAGgtgagctagctagctagcttt
421 cctcctcttttccttctttttcttcttccttccctatcaaaatttttacaacttcaagaa
481 tttcttttagcttcttcacaatgtttatttttcttggaagtttttgcatcttatcaaatt
541 ttcttgggaaacaaacagattatctctatcattaacgaatatcatgaccgtttttcttaa
601 gctaaatacaaaaaaggatttgccttttatacttgaaaaaagaaaagaaaagaaaagaaa
661 agaaaaaaaaaagcatttccttttaaagattttctcaagaaaagctgataagcttatat
721 ctagagttttccatccactcgcaaaagggctagttagtagttaaatttcatccttgaaaa
781 ttgagtttatttttaatttagtttaattgttttagaagtattaatttaaatttaatttaa
841 tttaatttaaaaattaaaaattttaaattaaagatttcttaatttaagttaaaaattaaa
901 gtttaatttatttttttaatttttgatttaattcaaaaataaaaattaaattaaattta
961 aattaaaaattttgagttaattaacataaaaaattaataagttaaattaaatatttttaa
1021 taaatataaaagtgaaattaaatattaagcgtaaaacaaacacggccgtagcttttgttg
102 G
1081 tcctattattagcgtattttagcagatggtgacgtgattttcctaatatgaccagGAGGG
103 H I N P A V T F G L F L A R K I S L V R
1141 CACATTAACCCAGCAGTGACATTCGGGCTATTCCTGGCCCGAAAGATCTCATTGGTGCGG
123 A V M Y M V A Q C L G A I A G V G L V K
1201 GCCGTGATGTACATGGTGGCTCAGTGCTTAGGAGCCATAGCCGGTGTCGGATTGGTGAAG

143 A F Q S S H Y K R Y G G G A N S L A N G
1261 GCGTTCCAGAGTTCCCACTATAAGAGGTATGGTGGTGGGGCCAACAGTCTGGCTAATGGG
163 Y S T G V G L G A E I I G T F V L V Y T
1321 TACAGCACAGGCGTTGGATTGGGTGCCGAGATCATCGGTACTTTTGTTTTGGTCTACACA
183 V F S A T D P K R S A R D S H V P
1381 GTGTTCTCCGCCACAGACCCAAAGAGGAGTGCCAGAGACTCCCATGTGCCCgtatgtacc
1441 ataacatctctcattccctcacttttctctattcttttactatttaaaattaattaatat
1501 ggtgtgtagctttttaagaaaatatattattttaaatatatttattaaaatttaaaaatt
1561 aattttaaattaattctaatatattttaattataaaatttttaaaactaattttcaaaaa
1621 atagaattataattttttttattactaataaacaagactacctacttaatttgactttatt
1681 tgatagcaaaaggtatattattcatatgacaaactcaatttaagtcccattgaccccacc
1741 taatttgtggttatagtaacctaatttattgcctttgcctcaaggtaaatgcaaaggtgg
1801 cttagaaattaatgctaggatctaatttcaatgaaaaaacagttataattaagaaagaat
1861 gatagtgtggacttataaaccaagcacaggtggaatattaatagctttcataagaaatta
1921 atggattaaactagaagaactctctagttaggatttcaggaccatttatgcatatttgaa
1981 atacgaattgagcttaattggtgttctaatttaattttaatcaatatgatattagtgtta
2041 tttttttaactattaaaagaaagaattctattctttggtaagatttagaaaatataatt
2101 tcatggtatgtaatattaatttaggcttattttcactagtttcaagaataaaaaaaagat
2161 agatggattcctttcgttcacatcatctttaatcagaagtttttaattatttatatttat
2221 atttttttatcttatgaaaatattatgctgtatttcattataatacttattaatgaattg
2281 ccaaagggtaagactataaaaaaaaaattgtgaaggagaaaagaaaagtgataattggga
2341 tgatatataataaaaattattagatgggtatgggaccttggcgcttgtggttgccacaaa
2401 aaggggtttcaatgccatttcttgtgttttttctttccttcaatgagtgaattctact
2461 gtttttgaccctttccaattcttgttcttaataaatgggtggttttgcttttgtgttac
2521 tattcattgtcattttctcctactttttagttgcaaagatcttacatagatttgtatgct
2581 tgcttcacatgtgctatagtttttttcctagtaattaacaaattttatttatttgattat
200 V L A P L P I G F A V F M V H L A T
2641 aaaattagGTTTTGGCTCCACTTCCAATTGGATTTGCTGTATTCATGGTTCACTTAGCCA
218 I P I T G T G I N P A R S L G A A V I Y
2701 CCATTCCAATCACTGGCACTGGCATCAACCCAGCTAGGAGTCTAGGAGCTGCTGTTATCT
238 N Q D K A W D D Q
2761 ACAACCAGGACAAGGCCTGGGATGACCAAgtcagtacacacacacacatatcattaaata
2821 aattaattatacttaattaattttgcaaagggtacttaattttcttgttaaattattatt
247 W I F W V G P F A G A A I
2881 aatttatttttttttattgcagTGGATCTTTTGGGTTGGACCTTTCGCTGGTGCAGCCAT
260 A A F Y H Q F I L R A G A V K A L G S F
2941 TGCAGCATTCTATCATCAGTTCATCTTGAGGGCTGGTGCTGTGAAGGCTCTTGGGTCATT
280 R S N P S T V *
3001 CAGGAGCAACCCCAGTACTGTT**TAA**gaaaaatccatcatcaaaaaaagaaataataatga
3061 aggaagagagagcatatgcatgcagctggttggctggtttctttgtagtggtgggttaaa
3121 aatcgatgaatgtgaaagagagagaatgaaaggattatgtaatttgtagatataaagtct
3181 ctttcaaaagtgaggcttttattatttttactgttatgtggcaatgatgtgatggttttc
3241 tagttagtttcaccctcttttgtctacttttcttctatgcacttatcatgcttgtgagca
3301 aagttaagctatttggtcaagtcttttgttgcttctgtcaaaagtttaggttgtgagtgt

3361 attattatgagcttttttttgtttttttttcag

12. *HbPIP2;7*

1 ataatttatcagctttccaaaagcaaagagaaaaaagccagaaataaccgttgtagcttg

61 tatgcattttggcttggatgcgtggctgacgcatccctgctctcttacattaatttgatg

121 attacaatcacatggttatcttttcatttcttcccaatatacccttatcttatccattaa

181 acccttccttctctcgttcttctttaaaagcgacccattctcctcactcctccattactc

241 acttacaagacaaccttcaatctcacaagacactgcttttggttttgggtttgacagaga

1 M A K E V S

301 tcaaaagaggagagagagagagagagagagagagagagagaag**ATG**GCTAAGGAAGTGAG

7 E E R Q P G K D Y V D P P P A P L I D M

361 TGAAGAAAGGCAGCCAGGTAAGGATTATGTTGATCCACCACCAGCTCCTCTAATCGACAT

27 A E I K L W S F Y R A L I A E F I A T L

421 GGCTGAGATCAAGCTCTGGTCTTTCTACCGTGCTCTTATAGCTGAGTTCATAGCCACTCT

47 L F L Y I T V A T V I G Y K K Q A D P C

481 TCTTTTCCTCTATATCACTGTAGCTACTGTAATTGGCTACAAGAAGCAAGCTGACCCTTG

67 A G V G L L G I A W A F G G M I F I L V

541 TGCTGGAGTTGGCCTTCTGGGTATTGCATGGGCCTTTGGTGGCATGATTTTCATTCTGGT

87 Y C T A G I S G

601 TTACTGCACTGCTGGTATCTCCGgtaagaaacctttttttctacgccttaactaccttcta

661 aattatgcatgcgcaaatcgtattatttgatgaaatttagttatttttgtaatccattta

721 acgaaacgtatgcgaattagtgtttttctgttttgtatactcaaagccatctctctactt

781 gaaaaatactctgcatctagttggttttgagaggtgtttgtttatgggttttggttgttt

95 G H I N P A V S F G L F L A R K V

841 tgggtgcagGTGGTCATATTAACCCGGCGGTCAGTTTTGGACTGTTCTTGGCGAGGAAGG

112 S L I R A V A Y M V A Q C L G A I C G V

901 TTTCACTAATCAGGGCTGTGGCTTACATGGTGGCTCAGTGCTTGGGTGCAATTTGTGGTG

132 G L V K A F M K H P Y N G L G G G A N T

961 TTGGGTTGGTGAAAGCATTTATGAAGCATCCATATAATGGCCTCGGAGGTGGTGCAAACA

152 V A P G Y N K G T A L G A E I I G T F V

1021 CAGTGGCACCTGGCTACAACAAAGGCACTGCTTTGGGTGCTGAGATCATCGGCACTTTTG

172 L V Y T V F S A T D P K R S A R D S H V

1081 TACTTGTCTACACTGTTTTCTCTGCAACTGACCCTAAGAGGAGTGCACGTGACTCTCACG

192 P

1141 TCCCTgtatgtctactggcttgatcttcttgttctcttcttagtgaaaatagtttagttt

193 V L A P L

1201 gcctttgaagttttgatgaatatttggttaattaattggattcagGTGTTGGCTCCTCTT

198 P I G F A V F M V H L A T I P I T G T G

1261 CCAATTGGGTTTGCTGTGTTCATGGTCCACTTGGCAACAATCCCCATCACTGGTACTGGT

218 I N P A R S F G A A V I Y N N D K A W D

1321 ATTAATCCTGCTAGGAGCTTTGGTGCTGCAGTTATCTACAACAATGACAAAGCCTGGGAT

238 D H

1381 GATCATgtatggccccttattaaatcttatacaatttaaaactttttatttttttaagcta

240 W I F W V

1441 ggtaattaattaagtgtgttatatataaatgatataattattacagTGGATTTTCTGGGT

245 G P F V G A L A A A A Y H Q Y I L R A A
1501 TGGTCCTTTCGTTGGAGCACTTGCAGCAGCAGCATACCATCAATACATACTGAGAGCAGC
265 A I K A L G S F R S N P T N *
1561 AGCCATCAAAGCTTTGGGATCTTTCCGCAGCAACCCCACCAAC**TAA**agaaagagaaatct
1621 gcacaaaaaaaaaagaaaaaaaaaagcctctttctctctactcttctttttctctttcaa
1681 attgtttgtttgtttgtgtgtattatgagaggattatgatgataaccctttttctttttc
1741 acttcaatctttaatctttctttgttaatttaattagtctctggctctgtatcattgtta
1801 tcaagtagtgctgtttttctttttcctcttttcacacattgccctctgctgcagtgaaaa
1861 gattcctctctatccttccacaaaaatcttaactaattttgattaattggcctgtaaatg
1921 acaaaaagaaaaagtacccttaaccaaattagcttaaatttaattataagtctgatttat
1981 tctg

13. ***HbPIP2;8***

1 cttccctctcttctcttacactagatgattagcttcacatgctcttatctgtcttttctt
61 ttcttcccaaaatgcccttctcttatgcattaaatcctcctttctctctccctctctctc
121 tctctcttctttaaaaccactgtcaaccgatctcttcttctatcagctactccaatacac
181 accaccaccttcaagctcacagtgttcttggcttttgggttttgagagagagagagagag
1 M A K E V S E E T Q P T H G K D Y V D P
241 c**ATG**GCTAAGGAGGTGAGTGAAGAAACGCAGCCTACCCATGGGAAGGACTATGTTGATCC
21 P P A P L I D V A E L K L W S F Y R A L
301 ACCACCAGCTCCTCTCATTGACGTGGCTGAGCTCAAGCTCTGGTCTTTCTACCGTGCTCT
41 I A E F I A T L L F L Y I T V A T V I G
361 TATAGCTGAGTTCATAGCCACTCTTCTTTTCCTCTACATCACTGTAGCTACTGTAATTGG
61 Y K K Q A D P C G G V G L L G I A W A F
421 CTACAAGAAACAAGCTGACCCTTGTGGCGGAGTTGGGCTTCTGGGTATTGCATGGGCCTT
81 G G M I F I L V Y C T A G I S G
481 TGGTGGCATGATTTTTATCCTTGTTTACTGCACTGCTGGTATCTCTGgtaattaagaacc
541 ctttcctctactccttaattatacatgcatccatataatggttacttttgtattgctttt
601 ttaaagaaacttatgcaaattagtgtttttctgttttctgtttatgtttatactaaaact
661 catctctctacttgaaaaatactgtgtaccccatttggttttgagaggtgtttgttaatg
97 G H I N P A V T F G L F
721 ggttttgcttgtttttgagtgcagGTGGTCATATTAACCCAGCGGTCACTTTTGGACTTT
109 L A R K V S L I R A V A Y M V A Q C L G
781 TCTTGGCGAGGAAGGTGTCACTGATTAGGGCAGTGGCTTACATGGTGGCTCAGTGCTTGG
129 A I C G V G L V K A F M K H P Y N A L G
841 GTGCAATCTGTGGTGTTGGGTTGGTGAAGGCATTTATGAAGCATCCATATAATGCTCTTG
149 G G A N S V A H G Y N K G T A L G A E I
901 GAGGCGGTGCTAACTCCGTGGCTCATGGTTACAACAAAGGCACCGCTTTGGGTGCTGAGA
169 I G T F V L V Y T V F S A T D P K R S A
961 TCATAGGCACTTTTGTGCTTGTCTACACTGTTTTCTCTGCCACTGACCCTAAGAGGAGTG
189 R D S H V P
1021 CACGTGACTCTCACGTCCCTgtatgtccactacctcgatctcttctcttctcatctcttc
195 V
1081 ttagcaagtataatgggtcttggttttgatagtatttggttaattaattgggttcagGTG
196 L A P L P I G F A V F V V H L A T I P V

1141 TTGGCTCCACTTCCAATTGGGTTTGCTGTGTTCGTGGTTCACTTAGCAACAATCCCCGTC
216 T G T G I N P A R S F G A A V I Y N N D
1201 ACTGGTACTGGTATTAATCCTGCTAGGAGCTTTGGTGCTGCAGTCATCTACAACAATGAC
236 K I W D D H
1261 AAAATCTGGGACGACCATgtatgacccattaaaaatcttttatatattggaaaaatttttt
1321 aatttttttcctattttttaagttagttgaatggtttataacatgtaaattattatttatt
242 G F S G L D L S L E H L Q L Q H I I N
1381 gcagtGGATTTTCTGGGTTGGACCTTTCATTGGAGCACTTGCAGCTGCAGCATATCATCA
261 T F * E Q Q P S R L W D L S A A T P P T
1441 ATACATTCTGAGAGCAGCAGCCATCAAGGCTTTGGGATCTTTCCGCAGCAACCCCACCAA
1501 C**TAA**aacaagaacgagaaatctccaccaagaaaagaaagcaaagaaaaaaaaaaaaaaaa
1561 aagcttgttcatctctagtctttctttatttttattctcaatttgtttgtttgtttgtgt
1621 gtattatgagaggattatgatgatgactcttttttttctcacttcaattcttaaatctttc
1681 ctttggataaaaaaaaaaaaaaaatcttaatctttctttgttaatttaattaatcctctgg
1741 ctgtgtatcattgttatggagtataatttaattaatgctctctttttttacacatggctcc
1801 gtgcagtgaaagaatctctgtagaaaggctaactaattttgattaattagccagtaaatg
1861 acaaaaaca

14. *HbPIP2;9*

1 gggaaaagaaaagttcacatcatttgccaactgatgtaatcctgatacgttttgttccat
61 tatgctaaagcatatgctccccttactcttggataaattcaatcttatataattctcact
1 M A K
121 ccactcactgtaagctctctctcatcttgtatgctaaataattaattttcaag**ATG**GCGA
4 E V T E E A G E A S Q Q E R D Y V E P P
181 AGGAAGTGACAGAAGAAGCAGGGGAGGCTTCGCAGCAAGAAAGAGACTATGTGGAACCGC
24 P A P L F D P E E L G L W S F Y R A V I
241 CACCAGCACCCCTTTTCGACCCGGAAGAGCTTGGTCTGTGGTCTTTTTACAGAGCTGTCA
44 A E F I A T L L F L Y V T V A T V I G Y
301 TCGCAGAGTTCATTGCTACCCTTCTCTTTCTCTATGTCACTGTTGCCACCGTGATTGGTT
64 K K Q T D P C A G V G F L G I A W S F G
361 ACAAGAAACAAACTGACCCCTGTGCAGGCGTTGGCTTTCTCGGCATTGCATGGTCTTTTG
84 G M I F I L V Y C T A G I S G
421 GTGGCATGATTTTCATCCTTGTATACTGTACTGCTGGAATATCAGgttcaaattcgataa
481 ccctgtacttatattttcttgacctttagtaacttcaggcagatcaagtgatgaatttct
99 G H I N P A V T F G L F L A
541 tggttatttacgcacagGTGGTCACATTAACCCGGCTGTGACCTTTGGTTTGTTCTTGGC
113 R K V S L V R A I A Y M V A Q C L G A I
601 AAGGAAGGTGTCACTGGTGCGAGCAATTGCTTACATGGTTGCTCAGTGCTTGGGAGCAAT
133 C G V G I V K G I M K D F Y N A Q G G G
661 ATGTGGAGTAGGTATAGTTAAGGGAATTATGAAGGACTTTTATAATGCACAAGGAGGTGG
153 A N T V A A T Y S K G T A L G A E I I G
721 TGCTAACACTGTGGCTGCGACATACTCCAAGGGAACTGCTCTAGGAGCTGAGATTATTGG
173 T F V L V Y I V L S A T D P K R N A R D
781 GACTTTTGTCCTCGTCTATATTGTGTTATCTGCCACTGACCCCAAGCGTAACGCGCGTGA
193 S H V P

841 TTCTCACGTTCCTgtaagtagtttaataaaatcgctaattactatagccttcgtctttca
197 V L A P L P I G F A
901 atttcccttctgatgagagggacatggctagGTTTTGGCTCCCTTGCCAATTGGGTTTGC
207 V F M V H L A T I P I T G T G I N P A R
961 AGTTTTTATGGTGCACCTAGCTACCATCCCCATAACAGGCACTGGAATCAACCCGGCTCG
227 S F G A A V I Y N N D K V W D D Q
1021 TAGCTTTGGGGCTGCTGTTATATATAACAACGACAAAGTCTGGGATGACCAGgcaggttc
1081 ctaactatataagcttgttgtaattcattcttacttaattatataattagagattgttta
244 W I F W V G P
1141 ttaatcttttgatcgtggctttatattgttgaggaacagTGGATCTTCTGGGTAGGGCCA
251 F V G A L A A A I Y H Q H I L R G T A I
1201 TTTGTGGGAGCACTTGCTGCAGCAATATATCACCAGCACATACTGAGAGGCACAGCCATA
271 K A L G S F T T N N *
1261 AAAGCACTGGGATCGTTCACCACCAACAAT**TAA**taacaactaagcgaatccctcctctct
1321 gcaaatctcctctatatggcatgcagtcagctagctgtataattgtatttgtatttcatt
1381 gttttcgttcgtagtctggaatctgcactttatggtgattggagacttgtgagcttgtgt
1441 ttgtggtctcttcaattattggttcatcaaacattttaatttgtaaatctgatttgtagt
1501 taaaagttggttttcttgttttaatttattgttgggatatatatatatatatattcgtct
1561 gttaaattattgccgagtgtagtctaataaacaactttatatatatgtatatatatattt
1621 gccagaatatcatggagcttaagtggac

15. *HbPIP2;10*

1 gcccatgcagccactcttatctccataaatctgaagtatctctcccttgagcagaagagc
1 M V K E M G E E G S F E H
61 aaggagtattgtgcagtaaaat**ATG**GTTAAGGAAATGGGTGAAGAGGGTTCTTTTGAGCA
14 E I H G Q H G K D Y V D P P P A P L L D
121 CGAGATCCACGGGCAGCATGGGAAGGACTACGTAGACCCACCACCAGCACCGCTGCTGGA
34 M E E L R R W S F Y R A L A E F V A T L
181 CATGGAAGAGCTGAGGCGTTGGTCTTTTTACAGAGCTCTTGCAGAGTTCGTGGCCACCCT
54 L F L H V S V A T V I G Y K S Q A D P C
241 TCTCTTCCTTCATGTCTCCGTTGCTACTGTTATTGGGTATAAGAGCCAAGCTGATCCTTG
74 A T V G F L G V A W A F G G M I F I L V
301 TGCCACTGTTGGCTTTCTTGGCGTTGCTTGGGCCTTTGGTGGCATGATTTTTATCCTTGT
94 Y C T A G I S G
361 TTATTGCACTGCTGGGATATCTGgtaagtatttatcacactatatacaagaaatccatta
421 atttaatttttcttctttctacacttctaataatactacttaatcattaatttttttaat
481 taattatgttaataaaaactcactaatctaaattaatcaatataagttaccaaatgaata
541 actattttcattgaaatctttcaaaaatagaacaaagaaaactccaatacaaaatttatt
601 ttataaagaacttagaaaaatagactagagaaataaattacaaaatttccatgaaaccac
102 G H I N P A V T F G L L L A R K L S L
661 agGAGGACATATTAACCCAGCGGTGACGTTCGGGCTGCTCCTGGCTAGGAAGTTGTCGCT
121 V R A V A Y M V S Q C L G A I A G A G L
721 GGTACGAGCAGTAGCGTACATGGTGTCACAATGCCTAGGAGCCATTGCCGGCGCTGGCTT
141 V K A V M K D D Y K S L G G G V N S V S
781 AGTGAAGGCAGTGATGAAGGATGACTACAAATCTCTCGGCGGTGGTGTCAACTCAGTTTC

161 S G Y S K G T A L G A E I I G T F V L V

841 TTCTGGTTACTCAAAAGGCACTGCTCTGGGAGCTGAGATCATCGGCACTTTCGTCCTTGT

181 Y T V F S A T D P K R K A R D S F V P

901 CTACACCGTCTTCTCCGCCACCGACCCTAAACGGAAAGCCCGCGATTCCTTCGTTCCAgt

961 aagtactataataacctaaagctgaggtttctcgatatcaatgaggtttttgatgtggtg

200 I L V P L P I G F A V F V V H

1021 tttgtgtgtggttcagATTTTGGTTCCCTTGCCCATTGGGTTTGCTGTGTTCGTAGTTCA

215 L A T I P I T G T G I N P A R S L G P A

1081 TTTGGCCACAATTCCCATCACTGGCACTGGCATCAACCCTGCTAGGAGCTTAGGTCCTGC

235 V I Y N K K T I W D D H

1141 TGTGATTTACAACAAGAAGACCATTTGGGATGATCACgtatgcccattttcttttctttt

1201 tcattttacttgcaattatacaccagcagattgacaattttatctttcacacttcattag

1261 gattttaaattttagaataaattacaaaattaacataaatttatttatttatttcttttg

247 W I F W A G

1321 tgcgtcataaatgatccgatttttttttggcaaaataaaacagTGGATTTTCTGGGCTGG

253 P F L G A L A A A L Y H Q Y V L R A G A

1381 GCCATTTCTGGGAGCTCTGGCTGCGGCACTATATCACCAGTACGTCCTGAGAGCAGGAGC

273 A K A L G S F R S N R S I *

1441 CGCTAAAGCTTTAGGATCGTTCCGTAGCAACCGCTCCATC**TAA**agaggctcaacaactga

1501 aaattcctacagaatttatccgcttagtgcactatgaaagcattctatgtattgtaatgg

1561 tttaataagctctgggattgaatacttggtatctaaattat

16. *HbTIP1;1*

1 gtctctagtctctagtgagctctacagagtgatcatcgtttggttttccaccgtttgatc

1 M P I R N I A V G H P Q E A

61 ttcatcgtacgcgaggaag**ATG**CCGATCAGAAACATCGCCGTAGGCCATCCCCAGGAGGC

15 T H P D A L K A A L A E F I S T L I F V

121 AACTCACCCAGACGCCTTGAAGGCGGCTCTGGCTGAGTTCATCTCTACTCTTATTTTCGT

35 F A G E G S G M A F S K L T N N G A T T

181 GTTCGCCGGAGAAGGTTCCGGTATGGCCTTTAGCAAGCTCACAAACAACGGTGCAACCAC

55 P A G L V A A S I A H A F A L F V A V S

241 CCCTGCTGGTCTCGTTGCTGCATCCATCGCGCACGCTTTTGCACTTTTTGTTGCTGTTTC

75 V G A N I S G G H V N P A V T F G A F V

301 CGTTGGCGCCAACATTTCCGGTGGTCATGTCAACCCTGCTGTTACATTTGGCGCCTTCGT

95 G G N I T L L R G I L Y W I A Q L L G S

361 CGGCGGCAACATCACTCTTCTTCGTGGAATCCTCTACTGGATCGCTCAGCTCCTCGGCTC

115 T V A C L L L K F S T G G L

421 CACAGTCGCTTGCTTGCTTCTCAAGTTCAGTACCGGTGGCCTGgtaaataagtaacgcta

481 taaaataattcataattaattccattcgttacagttcagttataactgagtgtgaatttt

129 T T A G F A L S S G V G V W N

541 gatatggtgctacgcagACCACGGCTGGTTTCGCACTCTCTTCCGGGGTTGGTGTATGGA

144 A F V F E I V M T F G L V Y T V Y A T A

601 ACGCGTTCGTTTTCGAGATCGTGATGACCTTTGGGCTAGTGTACACAGTATACGCCACAG

164 I D P K K G N L G I I A P I A I G F I V

661 CCATTGATCCAAAGAAGGGTAATTTGGGAATTATTGCACCCATCGCAATTGGTTTCATTG

184 G A N I L A G G A F D G A S M N P A V S
721 TAGGAGCAAATATTTGGCGGGAGGGGCATTCGACGGAGCATCGATGAACCCAGCGGTGT
204 F G P A L V S W S W D N H W V Y W A G P
781 CGTTTGGACCAGCCTTGGTGAGCTGGAGCTGGGACAACCACTGGGTGTACTGGGCTGGGC
224 L I G G G L A G V V Y E L L F I G H N T
841 CACTCATCGGTGGTGGACTTGCTGGGGTCGTTTATGAGTTGTTGTTCATCGGACACAACA
244 H E Q L P S T D Y *
901 CCCACGAGCAGCTCCCCTCCACTGACTAC**TAA**accagccgcggtggtggtggtgcatgga
961 tgatgggggtgactcggctttctttctttggtttttttttttttatctttcgagggtaat
1021 tatttatcccgtgtttaatctatgatcgttgatcttttctccaacactatcatccaaggg
1081 tgtgtgatttgtgtgttctgctcctgttgtattattcctttttttaactagtcatcttaac
1141 ctttttttttctctgagcggttcccaatttca

17. *HbTIP1;2*

1 tttaaatatttccctttttttggactttttttttttaatttctatatacactattggatct
61 ttccagagaccaaccatacagacaaaataatccacatcagccgaacgttacgtgctacgt
121 gtcaataattggaccggctattgcctcattgccccttgcctagctgtatcaacaccggt
181 gactggtagatggacctagactgaattttacaatttcaaaataaattccttaaatcgctt
241 tttacacgctgtccgccaatcggcttctaaaatattatttttcgtccccaatatttcaac
301 cggaaaccggttactgcgggttccccaggaccactctttcgatataagttgagaatgggg
361 agaccaggtactcgcactagcaagctctgcacccagtgtaagagagagatcatcgtttgg
1 M P I R N I A V
421 tttccaccgtttgatcttcatcatcacacgaggaag**ATG**CCGATCAGAAACATAGCCGTA
9 G H P Q E A T H P D A L R A A L A E F I
481 GGTCATCCCCAGGAGGCGACTCACCCAGACGCCTTGAGGGCAGCTCTGGCTGAGTTCATC
29 S T L I F V F A G E G S G M A F S K L T
541 TCCACTCTTATTTTCGTCTTCGCCGGAGAAGGTTCTGGTATGGCCTTTAGCAAGCTCACA
49 D N A A N T P A G L V A A S I A H A F A
601 GACAACGCTGCAAACACCCCTGCCGGTCTCGTTGCTGCATCCATCGCCCACGCTTTTGCC
69 L F V A V S V G A N I S G G H V N P A V
661 CTATTCGTTGCTGTTTCCGTTGGCGCTAACATCTCCGGTGGCCATGTCAACCCTGCTGTT
89 T F G A F V G G N I T L L R G I L Y W I
721 ACCTTCGGTGCTTTCGTTGGTGGGAACATCACTCTCCTCCGTGGTATCCTCTACTGGATC
109 A Q L L G S T V A C L L L K F S T G G L
781 GCTCAGCTCCTCGGATCCACCGTCGCTTGCTTGCTTCTCAAGTTCAGCACCGGTGGCCTG
841 gtaattaagcctataataattcatgcataattacagtaaccgttgtatatacagttctaa
129 T T S A F A L S S G
901 ctgattgtgagttttgatatggtgctacgcagACCACCTCAGCTTTCGCTCTTTCTTCTG
139 V G V W N A F V L E I V M T F G L V Y T
961 GTGTTGGTGTATGGAACGCTTTCGTTTTGGAGATCGTGATGACCTTCGGGCTTGTGTACA
159 V Y A T A I D P K K G N L G I I A P I A
1021 CAGTATACGCCACAGCCATTGATCCAAAGAAGGGCAATTTGGGAATTATTGCACCCATCG
179 I G F I V G A N I L A G G A F D G A S M
1081 CAATTGGTTTCATCGTAGGAGCTAACATTTTGGCGGGAGGGGCTTTCGATGGAGCCTCCA
199 N P A V S F G P A L V S W S W D N H W V

1141 TGAACCCAGCAGTGTCGTTTGGACCAGCTTTGGTGAGCTGGAGCTGGGACAATCACTGGG
219 Y W A G P L I G G G L A G L I Y E F F F
1201 TGTACTGGGCTGGGCCTCTGATCGGTGGTGGGCTTGCTGGGCTCATCTACGAGTTCTTCT
239 I G H N T H E Q L P T T D Y *
1261 TCATCGGCCACAACACCCACGAGCAGCTCCCCACCACCGACTAC**TAA**accagcgctgctg
1321 atggtggtggtgcatggatgatcgggtggctcggcttttcttttctttctctggtgtatt
1381 catttttatcttttgagggtaattatttatcctttgtttaaattgtgatcgttgatcttt
1441 ttttttttttgtcacaatcatccaagggtgtgatttgtgtattctgcttcggttgtatga
1501 ttccatttgtagtcatcttgcctttttttttttttttcctaatctcaattccaaatttc

18. *HbTIP1;3*

1 agggatctcatccacaggaattaagtactcctcactactagctagtgtagctcgttctct
1 M P I N R
61 atccgactatctaatctcagccgtttgatatttagagattaaccaaa**ATG**CCGATCAATC
6 V A I G L P R E D V V H P G A L K A A L
121 GGGTAGCAATCGGGCTGCCAAGAGAGGACGTTGTGCATCCCGGTGCACTTAAGGCCGCAT
26 A E F I S T A I F V F A G Q G S G M A F
181 TGGCAGAGTTCATTAGTACAGCAATTTTTGTTTTCGCCGGACAAGGTTCCGGTATGGCCT
46 S K L T D N A S N T P A G I I M A S L A
241 TTAGCAAACTTACGGATAATGCGTCCAACACACCTGCCGGAATTATCATGGCCTCATTGG
66 H A F G L F V G V S T A T N I S N G H V
301 CACATGCATTTGGCCTTTTCGTCGGTGTGTCTACAGCAACCAACATCTCTAACGGCCACG
86 N P A V T F G A F L G G N I S L L R G I
361 TCAATCCCGCCGTCACCTTTGGTGCCTTCCTTGGTGGCAATATCTCTCTCCTCCGAGGCA
106 L Y W I A Q L L G S T V A C L L L K F S
421 TTCTTTATTGGATCGCTCAGCTCCTTGGCTCCACCGTGGCCTGCCTACTACTCAAGTTTT
126 T H G M
481 CTACTCATGGGATGgtgagtttttgcatatctaaagttctttttttttttgcattattata
130 T T S A F A L
541 aatatttctaacaagttcataaacatgggaatttctgcagACAACATCGGCATTTGCTTT
137 S S G V N V W N A L V F E I V M T F G L
601 GTCGTCAGGGGTGAATGTGTGGAATGCACTTGTGTTCGAGATTGTAATGACGTTTGGCCT
157 V Y T V Y A T A F D R N K G D V G I I A
661 AGTTTACACAGTATATGCAACAGCCTTTGATCGTAATAAGGGTGACGTGGGGATTATTGC
177 P L A I G F V V G A N I L A G G A F E G
721 TCCTCTAGCAATCGGTTTCGTTGTTGGAGCCAACATTTTAGCTGGCGGAGCATTTGAAGG
197 A S M N P A V S F G P A L V S W D W T N
781 AGCATCTATGAACCCAGCCGTGTCTTTTGGACCTGCTTTGGTGAGCTGGGACTGGACCAA
217 H W V Y W V G P L I G G G L A G I I Y N
841 CCATTGGGTTTACTGGGTAGGTCCCTTGATCGGAGGTGGACTTGCTGGAATCATTTACAA
237 L F F I T R T H E P V P S T S E F *
901 TCTCTTCTTTATTACCCGCACTCATGAGCCGGTGCCTAGCACATCAGAGTTC**TGA**actaa
961 aagtactaagcatatccaccgcttttcatgatttctctttattctttcatattccaatgt
1021 gttggatttcattgttattgttgttgtaagattggagtctgtctgctttgatcatggtta
1081 attattagaggttgtagcttagtgaagttgtcttattttctcactattgtattgtctaat

1141 tcccatggccatcatcatttgtttatcgtttttggaatctgtctttttcctttagctct
1201 tctttcttgcccaaaaaattataatgcgcaaaccggagctaaaatgatctgc

19. *HbTIP1;4*

1 M P I N R I E V G L P R Q D V T H P S A
1 **ATG**CCAATCAATCGGATAGAAGTCGGGCTGCCGCGACAGGATGTAACGCATCCCAGTGCA
21 L K A A L A E F I S T L I F V F A E E G
61 CTTAAGGCGGCATTGGCAGAGTTCATTAGTACTTTGATTTTTGTCTTCGCCGAAGAAGGT
41 S G M A F S R L T D N A S N T P A G I I
121 TCCGGTATGGCCTTTAGCAGACTTACGGACAATGCATCCAACACACCTGCCGGCATTATC
61 M A S L A H A F G L F V G V S T A F N I
181 ATGGCCTCATTGGCACATGCATTTGGCCTTTTTGTCGGGGTTTCTACCGCTTTCAACATC
81 S G G H V N P A V T F G A F L G G S I S
241 TCCGGCGGCCATGTCAACCCCGCCGTTACCTTTGGTGCCTTCCTTGGTGGCAGTATCTCT
101 L I R G I L Y W I A Q L L G S T V A C L
301 CTCATTCGTGGCATTCTTTATTGGATCGCTCAGCTCCTTGGCTCCACTGTGGCCTGCCTG
121 L L K F S T H G M
361 CTACTTAAGTTCTCTACTCATGGCATGgtgagtactggaattttaagctacttaagtgtt
421 aaaaaacatgttaatataagaaaaaacatgttaatataagctttaatctatatttgaacg
130 T A S A F S
481 tgaatattggtaacaagttcagaaacatggcaaatttatgcagACAGCATCGGCATTTTC
136 L S V R G E C V E C T C I R D C T D V C
541 TTTGTCGGTCAGGGGTGAATGTGTGGAATGCACTTGTATTCGAGATTGTACTGACGTTTG
156 L V Y T V Y A T A L D P K K G E V G I I
601 CTTAGTTTACACAGTATATGCAACAGCTCTTGATCCTAAAAAGGGTGAGGTGGGGATTAT
176 A L L A I G F V V G A N I L A G G A F E
661 TGCACTTTTGGCAATTGGTTTCGTTGTTGGAGCTAACATTTTAGCTGGGGGAGCATTTGA
196 G A S M N P A V S F G P A L V S W D W A
721 AGGAGCATCCATGAATCCTGCAGTGTCTTTTGGACCTGCTTTGGTGAGCTGGGACTGGGC
216 N H W V Y G I F I K K V *
781 CAACCATTGGGTTTACGGGATTTTTATAAAAAAGGTG**TAA**

20. *HbTIP1;5*

1 cagttcatactgcgaatgctaaataaagaaaaagaaatagcaaaacagagcttcttctaa
1 M P I T R I A V G N P G E A S Q P
61 aattcaagaaa**ATG**CCCATCACCAGAATTGCAGTTGGGAATCCGGGAGAGGCCAGCCAAC
18 D A L R A A L A E F F S M I I F V F A G
121 CAGACGCCCTCAGGGCGGCTCTGGCCGAGTTCTTCTCTATGATTATTTTCGTATTCGCCG
38 E G S G M A F N
181 GCGAAGGATCCGGCATGGCTTTCAgtaagatatttaaatatagatagctattcatttttt
46 K L T D
241 tttttcacaattgaattggactaatatttagtggatgttttggcagATAAGTTAACGGAC
50 N G S T T P A G L I A A S L A H A F A L
301 AATGGGTCGACGACGCCGGCGGGTCTAATAGCTGCATCATTGGCTCATGCATTTGCACTT
70 F V A V S V G A N I S G G H V N P A V T

361 TTTGTAGCAGTTTCGGTTGGTGCTAACATCTCTGGAGGTCATGTAAATCCTGCTGTTACT

90 F G A F I G G N I T L L R G I L Y W I A

421 TTTGGAGCATTTATTGGAGGGAATATTACTCTGTTAAGAGGGATTTTGTACTGGATTGCA

110 Q L L G S V V A C L L L K F A T G G L

481 CAGTTGCTTGGATCTGTTGTTGCTTGCTTGCTTCTTAAATTCGCCACTGGTGGATTGgta

541 tggtcgaaaattgtccaagtttcttgaaaaattagacatgggtttgatgctaaattctga

129 E T S A F A L S S G V

601 tttctataccttgttcattcactgcagGAAACATCAGCATTTGCGTTATCATCTGGGGTG

140 S S W N A L V F E I V M T F G L V Y T V

661 TCTTCATGGAACGCACTTGTGTTTGAGATAGTGATGACCTTCGGCCTAGTCTACACTGTG

160 Y A T A V D P K K G N V G I V A P I A I

721 TATGCTACTGCAGTGGATCCCAAGAAAGGGAATGTGGGAATTGTTGCACCCATTGCAATT

180 G F I V G A N I L A G G A F D G A S M N

781 GGTTTCATTGTTGGTGCCAATATTTTGGCTGGTGGTGCTTTTGATGGTGCATCCATGAAC

200 P A V S F G P A V V S W T W T S H W V Y

841 CCAGCAGTTTCCTTTGGCCCTGCTGTTGTAAGCTGGACATGGACTAGTCACTGGGTCTAC

220 W V G P L I G A A I A A L V Y D N I Y I

901 TGGGTGGGTCCATTGATTGGTGCTGCCATTGCAGCCCTTGTCTATGATAACATCTACATT

240 G E N A H E P L S T S D F *

961 GGTGAAAATGCACACGAGCCACTTTCCACCAGTGATTTC**TAA**gaattgaatcccattttc

1021 atgttagaaattaaatgcctgtctcctttttttttttttacattaaggagcaataaaaggt

1081 ttagatttctgttgtagtttggttgtgttaggcgtttactgctgttgtttccacttattt

1141 tatagcagtggatatcaaattttcattagtttgtcttttccctgttgaatcaacgaaact

1201 gttttgtttatgagttattcttttctaaactcagtcacctaagcatataaacttggagaa

1261 tatgataaaaacatacaaacaaacttggaatctgtaaatgattttaggtattttagactt

1321 tgatgcgacatcaaagcaaggaattatcaggcaggaagccatacagccaagccatgtgaa

1381 ttaagggaaccataacggcctggtcatgtaagaggtcatcgtgctccaaaaagttacact

1441 ggccgcggccaacgcaggattaattgcatggtcatgtctctgtatgtgctccaattgtct

1501 gttgatccattagtttacgttataaagccgtgtaataccccactatcgcttgtagttcaa

1561 taggctgttgctggattagtttgaatattccagttttgtttaggattcttaatttaattt

1621 aaattctttttctttatttgtagaattatattcctatataattaaggatttataaaccca

1681 ttcctctttagttcaggctttagaaaattatataaaccc

21. *HbTIP1;6*

1 M P V A R I

1 cactccagacatagctaacagagcttcttctaaaatacaagaaa**ATG**CCGGTCGCCAGAA

7 A V G N P G E A S Q P D A I R A A L A E

61 TTGCAGTTGGGAATCCAGGAGAAGCCAGCCAGCCAGACGCTATCAGGGCGGCTCTGGCGG

27 F F S M I I F V F A G E G S G M A F S

121 AGTTCTTCTCTATGATTATTTTCGTATTCGCCGGTGAAGGATCCGGCATGGCTTTCAgta

181 agagatatctcaatttttttttctttttttttggtttagaaaagagggtggcgaggatt

241 tgaaggatttgctcttttctctcaattgaattggactaacattggtgtatgttttggcag

46 K L T N N G S T T P A G L I A A S L A H

301 GTAAGTTAACGAACAATGGGTCGACAACGCCGGCAGGTTTAATAGCTGCATCCCTGGCTC

66 A F A L F V A V S V G A N I S G G H V N

361 ATGCATTTGCACTTTTTGTGGCGGTTTCGGTTGGTGCTAATATCTCTGGGGGCCATGTAA
86 P A V T F G A F I G G N I T L L R G I L
421 ATCCTGCTGTCACTTTTGGTGCATTTATTGGAGGAAATATCACTCTTTTGAGAGGGATTT
106 Y W I A Q L L G S V V A C L L L K F A T
481 TGTACTGGATTGCTCAGTTGCTTGGATCTGTTGTTGCTTGCTTGCTCCTTAAATTCGCCA
126 G G L
541 CTGGTGGATTGgtatggaacttgtccacaaactgtttgaaaaattagacatgggtttgat
129 E T S A F A
601 gccaaatttcgatttctaattttctatgccttgttcgctacagGAAACATCAGCATTTGC
135 L S S G V S S W N A V V F E I V M T F G
661 GTTATCATCTGGGGTGTCTTCATGGAACGCGGTTGTGTTTGAGATAGTGATGACCTTCGG
155 L V Y T V Y A T A V D P K K G N V G T V
721 CCTAGTCTACACTGTGTATGCCACTGCAGTGGATCCCAAGAAAGGGAACGTGGGAACTGT
175 A P I A I G F I V G A N I L A G G A F D
781 TGCCCCTATTGCAATTGGTTTCATTGTTGGTGCCAACATTTTGGCTGGTGGTGCTTTTGA
195 G A S M N P A V S F G P A V V S W T W T
841 TGGTGCATCCATGAACCCAGCAGTTTCCTTTGGCCCTGCTGTTGTAAGCTGGACATGGAC
215 S H W V Y W V G P L I G A A I A A I V Y
901 TAGTCACTGGGTCTATTGGGTGGGTCCACTGATTGGTGCTGCCATTGCAGCCATTGTCTA
235 D N I F I G K N A H E P L S T N D F *
961 TGATAACATCTTCATTGGAAAAAATGCACATGAGCCACTTTCCACAAATGATTTT**TAA**ga
1021 ttttgaaattaaatgcctccccttttttcctttagatcactgaggagcaataaaaagtttg
1081 gatttttcttgtagcttggttgtattagcctattaggtatttactgttgttgttcctgct
1141 tattttatagcagtggatattagactttctttagttgtcttttacacgtcgaatcaatga
1201 agctgttctgtttatgaatggttacaatcccacattgttaagaaataatcttagcgagag
1261 attaataagtctttagactctctttcgataagatgattttcaagggtga

22. *HbTIP1;7*

1 ggaaaaaaaaattttttaatttgggctttggaagcagaagagccagctcaaagctaaatt
1 M P I T S I A I G S P A E A S Q P
61 aatccaaaaaa**ATG**CCGATTACTAGCATTGCAATTGGATCTCCAGCGGAGGCTAGCCAAC
18 D A L K A A L A E F I S M L I F V F A G
121 CAGATGCCCTCAAGGCAGCCCTTGCCGAGTTTATTTCTATGCTCATTTTCGTCTTTGCCG
38 E G S G M A F N
181 GTGAAGGATCCGGCATGGCATTTAgtaagaacctaacctaatttctctctctttatttcg
241 agatcatatacagttaaaaaattaagaaactcaattatgatttattcggatccatattgc
301 taataaaacgtatgtatatgatatataatttaatatgttttaataaaattattataattt
361 tagtaaaaaaatatagattataattaattagaattgattatgtaattttttttttatttaa
421 ttttatttaaaattaaaaaaatataaattttttgatattatttctctctgaaaagagcat
46 K L T D N G S
481 gttaatacaaaattcttatgaagcatatttttggcagACAAGCTGACCGATAATGGGTCA
53 T T P A G L V A A S L A H G F A L F V A
541 ACAACACCAGCCGGCCTAGTAGCAGCTTCACTGGCTCACGGATTTGCCCTGTTCGTGGCG
73 V S V G A N I S G G H V N P A V T F G A
601 GTTTCAGTAGGTGCTAATATTTCTGGTGGGCACGTAAACCCTGCTGTTACATTCGGTGCC

93 F V G G H I T F I R S V L Y W I A Q L L
661 TTTGTTGGTGGCCACATAACATTCATCAGAAGCGTTCTTTACTGGATTGCCCAATTGCTC
113 G S V V A C L L L K F A T G G L
721 GGTTCTGTGGTTGCTTGCTTGCTTCTTAAGTTTGCCACTGGTGGATTGgtaattacccct
781 tttatttattttgcatactgttttactgatactcgaactcgagactcacagctctggaga
129 E T S A F
841 cgactccactataatgctaactaaaataaatgtttttgtgattagGAAACATCTGCTTTT
134 A L S S G V G A W N A L V F E I V M T F
901 GCCCTATCATCTGGAGTTGGTGCATGGAATGCACTTGTTTTCGAGATCGTTATGACCTTT
154 G L V Y T V Y A T A V D P K K G N I G I
961 GGTTTGGTGTACACTGTATATGCAACAGCCGTGGACCCAAAGAAGGGTAACATCGGGATA
174 I A P I A I G F I V G A N I L A G G A F
1021 ATTGCACCCATTGCAATTGGTTTCATTGTGGGTGCCAATATCTTGGCTGGTGGTGCCTTT
194 D G A S M N P A V S F G P A V V S W T W
1081 GATGGTGCATCCATGAACCCAGCAGTCTCCTTCGGACCAGCAGTAGTCAGCTGGACATGG
214 D N H W V Y W L G P F L G A G I A A V V
1141 GACAACCACTGGGTCTACTGGCTCGGTCCATTCCTCGGGGCTGGCATCGCTGCTGTTGTT
234 Y E V F F I S P S T H E Q L P S T E F *
1201 TATGAAGTCTTCTTCATTAGCCCAAGTACACATGAACAGCTTCCCTCTACAGAATTT**TAA**
1261 gaattcagatttctttttggttttccctcttggtttatgtgatgagagaaggttgaaag
1321 ttgggtgcgtctctttggtttcaatgttgtattggtggtgctgttggcgtttgtatctgg
1381 ttcaagtacttctcgttaattgttgattcttgtatccttgtcttcttctaaaattcttcc
1441 atctttcaagtattttcttatttgttctcatttgattgattccaagcacacatcaccaca
1501 agttgagttatacttagaatagatttag

23. *HbTIP1;8*

1 M A
1 tttggctttaaaagcagaagagctcgatctcaaagctagactaaaatttggaaaca**ATG**G
3 I T S I A I G S P A E A S Q P D A L K A
61 CAATTACTAGCATTGCAATAGGATCTCCGGCGGAGGCTAGCCAACCAGATGCCCTGAAGG
23 A L A E F I S M L I F V F A G E G S G M
121 CAGCTCTTGCAGAGTTCATTTCAATGCTCATTTTTGTTTTTGCCGGTGAAGGCTCTGGCA
43 A F N
181 TGGCATTCAgtaagagcctagatagcttcatttatttttctaatctttcttagtttctta
46 K
241 atattgctctattttttaatccaattaataatgctgtcttcgtattttggcagACAAG
47 L T D N G S T T P A G L V A A S L A H G
301 CTGACCGATAATGGGTCAACAACACCGGCCGGCCTAGTAGCAGCCTCTCTGGCTCACGGA
67 F A L F V A V S V G A N I S G G H V N P
361 TTTGCACTGTTCGTGGCGGTTTCAGTAGGTGCTAATATTTCCGGTGGGCATGTAAACCCT
87 A V T F G A F V G G H I T L I R S V L Y
421 GCTGTTACTTTTGGTGCCTTTGTTGGTGGCCACATAACATTGATCAGAAGTGTTCTCTAC
107 W V A Q L L G S V V A C L L L K F A T G
481 TGGGTTGCTCAATTGCTTGGTTCTGTGGTTGCCTGCCTGCTTCTTAAGTTTGCTACTGGT
127 G W

541 GGATGGgtaataaaccccttcctttaacctaataatcatattaattatttgattctttaa
601 aagattttgtcaaaaaaaaagttttttttttaatagtggctgcaacattaatgtggttga
129 E T S A F A L S S G V G A G
661 ttttgcataaatgtttagGAAACATCTGCTTTTGCCCTATCATCTGGAGTTGGTGCAGGG
143 N A L V F E I V M T F G L V Y T V Y A T
721 AATGCACTTGTTTTCGAGATTGTCATGACCTTTGGTTTGGTGTACACTGTATATGCAACA
163 A V D P K K G D I G I I A P I A I G F I
781 GCCGTAGACCCAAAGAAGGGTGACATAGGGATCATTGCACCCATTGCAATTGGTTTCATC
183 V G A N I L A G G A F D G A S M N P A V
841 GTGGGTGCCAATATCTTGGCTGGTGGTGCCTTCGATGGTGCATCCATGAACCCAGCAGTC
203 S F G P A V V S W T W D N H W V Y W L G
901 TCATTTGGGCCAGCAGTGGTCAGCTGGACATGGGATAACCACTGGGTCTACTGGTTAGGT
223 P L L G A G I A A V V Y E V F F I S P S
961 CCATTACTCGGCGCTGGCATTGCTGCCGTTGTTTATGAGGTCTTCTTCATCAGCCCAAGT
243 T H E Q F P S A D F *
1021 ACGCATGAACAGTTTCCCTCTGCAGATTTT**TAA**gaattcttcagagagaaaaggttgaaa
1081 gttgggtggatctcttactttggtttgattgctgtgctgttggtgttgtttgtatgtggt
1141 tcaagtaattcttgtcaactgttgattcctgtaaccttgtttaatttctctttagattct
1201 ccaatcagttaaattttgttctctttcacatgtccatttcctaatcccaacaattcctct
1261 gctcctttcttttgctctcctatgatcaaccaagttactattgactatacaggccagttt
1321 tccaatatatatttcaacatgatcaat

24. *HbTIP2;1*

1 atggatgaatcattgatatgcatccagacatttctaataaagaaagttaatgacagctag
61 gatagcagaagaaatttgattggatgtgcatgtatgcattcccatattctttttatctat
121 cctcacatatatatcaaatacatgactaaaacaataataccccttaaacaatcattacttc
181 atttaataataaaaccttatattatactcatatatattttttatgtgtcaattcatagttg
241 tgtgtatatatatatacacatgacatgagaaaggcacatttgtgctatttctgaactctt
301 ttttacctacccttgcctggccttctcctccactgaagagttcacttccctctagctaag
1 M A R I A F G R F D D S F S L
361 ctaatcgtgtttcaaag**ATG**GCCAGGATTGCCTTTGGTCGCTTTGATGATTCTTTTAGTT
16 G S F K A Y L A E F I S T L L F V F A G
421 TAGGCTCTTTTAAAGCCTATCTTGCTGAATTTATCTCAACCTTGCTCTTTGTTTTTGCTG
36 V G S A I A Y N
481 GTGTTGGTTCAGCTATTGCTTACAgtcagttctatatctcgaactaatctacctatttgc
541 attatctaattaattacccatttttttagttactaattcttgatattattagtcttgcaac
44 K L T N D A A
601 gtataattatatagggtttttcttctctttgttacagATAAATTGACAAACGATGCAGCT
51 L D P A G L V A I A I C H G F A L F V A
661 CTTGATCCTGCTGGTCTAGTAGCCATTGCTATTTGCCATGGATTTGCTCTCTTTGTTGCA
71 V S V G A N I S G G H V N P A V T F G L
721 GTTTCCGTTGGAGCCAACATCTCCGGTGGCCATGTTAACCCTGCTGTTACCTTTGGCTTG
91 A L G G Q I T I L T G I F Y W I A Q L L
781 GCTCTTGGTGGCCAAATCACCATTCTCACTGGCATCTTCTACTGGATTGCCCAGCTTCTT
111 G S I V A C F L L K F V T G D L

841 GGCTCCATTGTTGCCTGCTTCCTTCTCAAATTTGTCACTGGAGACTTGgtaagttccatg

901 tccatgaacgccatcgtttatacaagtaatccctgacttctatattctcgtaatatcaag

961 attcttgtgcaagtaaaaccaagtgtagttttaagttttaacattttctttcctaattcg

1021 accgcagacatgataatacaataatttctgccatgggttgacaatgcttttaacatttta

1081 taatatgcttagcttaatgaggcgagtcagtctttttccattccccaatttttttaatca

1141 gagttttagtattaaagttggacataaaagacactaaatttttttaatcggatttttaata

1201 ttaaagggagaactcatgttccataaagtgggagagacaaccatgaatcctaaagagttt

1261 agtttagatcataaaatagacaaaaaaaaatacccgatcaataaaaaaaattaattttga

1321 catcatactttatctttaatttttttttttaaatatgaatgatgaatgcttaataaaata

1381 cataaaaattgccccatagtactattttccctaatacttagaaaaagaataaagttaata

1441 tatttttaaatcatatcactactttttttgtgcaatcaactaggggttaaacaagcttaa

1501 ttaattaccttaggatcatttgtacgtaattatcattaattgactatgtaaagttgtaaa

127 P I P T H S V A A G V G A

1561 cttttttttttcttcttttcagCCTATCCCCACCCACAGCGTTGCCGCCGGAGTTGGAGC

140 I E G V V M E I V I T F A L V Y T V Y A

1621 CATCGAAGGAGTGGTTATGGAGATTGTAATCACATTTGCTTTGGTATACACAGTGTATGC

160 T A A D P K K G S L G I I A P I A I G F

1681 TACTGCAGCCGACCCCAAGAAAGGATCCTTAGGCATCATTGCTCCCATTGCCATTGGTTT

180 I V G A N I L A A G P F S G G S M N P A

1741 CATCGTTGGTGCCAATATCCTGGCTGCAGGCCCATTCTCCGGTGGATCCATGAACCCAGC

200 R S F G P A V A S G D F H D N W I Y W V

1801 CCGGTCTTTTGGGCCTGCCGTCGCTAGCGGTGACTTTCATGACAATTGGATCTACTGGGT

220 G P L I G G G L A G L V Y G N L Y I P G

1861 TGGCCCCCTTATTGGAGGAGGGCTAGCTGGTCTCGTCTATGGAAACTTGTATATCCCTGG

240 D H A P L S N E F *

1921 TGATCATGCACCCTTATCCAATGAGTTC**TAA**gcttgtgcttcaaatccatttgcctatgt

1981 aataaaagaaaaaggagattattcccttctttcttttctttgcttttttttttttggggtc

2041 cttttcagttcatcatttgtctttgttgttgtggttatcttttgctttacgatgatgat

2101 gatgatgtgcagcaacttttctatcatggtcatggtttttttttttggcaatgtgattttt

2161 tttaatgacataaagaagatgattttttttccacgaccttgtaataattggaactaacaat

2221 atggccagtacaaaaa

25. *HbTIP2;2*

61 attggatatgtgcatgtagacatctccatattctttttatttatccctatatatgtataa

121 ataaaatatataattaattagaacaatagcccaagacttcgtttaataataaaactatta

181 tgctcctatatatgtgacatgtgaagagaaaggccattgcctggccctctcctccactga

1 M A

241 agagttcacttccctctaagctgagccaagttgagctaatcttctttcaaaaatt**ATG**GC

3 R I A F G R F D D S F S L G S F K A Y L

301 CAGAATTGCCTTTGGTCGCTTTGATGATTCTTTTAGTTTAGGCTCTTTTAAAGCCTATCT

23 A E F I S T L L F V F A G V G S A I A Y

361 TGCTGAATTTATCTCAACCTTGCTCTTTGTTTTTGCTGGTGTTGGTTCAGCTATTGCTTA

43 N

421 CAgttagttcattttctctctcttccactgatctacctatctggattatctaaactctat

481 taattattaaaccctttttttagttactaattcttgatattatttagtatcacaaatcgta

44
541 catagttatgttcttttttttttcccctgacttctttttcttcttgtctcttgtacagAT
44 K L T Y D A A L D P A G L V A I A I C H
601 AAACTGACATATGATGCAGCTCTTGATCCTGCTGGGCTAGTAGCTATTGCTATTTGCCAT
64 G F A L F V A V A V G A N I S G G H V N
661 GGATTTGCTCTCTTTGTTGCAGTAGCCGTGGGAGCCAACATCTCCGGTGGCCATGTTAAC
84 P A V T F G L A L G G Q I T I L T G V F
721 CCTGCTGTTACCTTTGGTTTGGCTCTTGGTGGCCAAATCACTATCCTCACTGGCGTCTTT
104 Y W I A Q L L G S I V A C F L L K F V T
781 TACTGGATCGCCCAGCTTCTTGGCTCCATCGTTGCCTGCTTCCTTCTCAAATTTGTCACA
124 G G L
841 GGAGGCTTGgtaagttcttcgtctatggacaccatcttttatatatatgtatatagaagt
901 agttcatgacttccatatcctatcctcagaatatcattatttcttcttatgcatgtaatc
961 aagggtagttttgacattttacttccttaattcgtccacaaggatgacagtagaaggttc
1021 tctgccaaaggttgaaaatgattttaacattttaattgatttgatatgatatttttgttg
127 A I P T H S V A A G V G A I E G V
1081 ttctctttcagGCAATCCCCACCCACAGCGTAGCAGCTGGAGTTGGAGCCATTGAAGGAG
144 V M E I V I T F A L V Y T V Y A T A A D
1141 TGGTTATGGAGATAGTCATCACATTTGCTTTGGTATACACGGTGTACGCAACTGCAGCTG
164 P K K G S L G I I A P I A I G F I V G A
1201 ACCCCAAGAAAGGTTCCCTTGGCATCATTGCGCCCATTGCCATTGGCTTTATTGTTGGTG
184 N I L A A G P F S G G S M N P A R S F G
1261 CCAATATCCTGGCTGCAGGCCCATTCTCCGGTGGATCAATGAACCCAGCCCGGTCCTTTG
204 P A V A S G D F H D N W V Y W V G P L I
1321 GGCCAGCCGTCGCTAGCGGTGACTTCCACGACAACTGGGTCTACTGGGTCGGGCCGCTTA
224 G G G V A G L I Y G N L Y I P S D H A P
1381 TTGGAGGTGGGGTAGCTGGTCTCATCTATGGAAACTTGTACATCCCTAGCGACCATGCGC
244 L S S E Y *
1441 CCTTGTCCAGTGAGTAT**TGA**agtttgtgcttctaatccatgaccttgtaataaaaagaaa
1501 gggggagattatgctcttccctcttttctttactgttccatttgtctttgttgttgtaa
1561 agccttgttgtgattaacttttgctttatgagaaaccaactatgcagaagatgtgcagca
1621 acttttctatcatgctcatctatttgcttttctggttttgttttgtttttcttctctgtt
1681 tttcaatagtgcatctgagtttatggacaaattcaaaatgttatgttcacattagggcat
1741 ttatattcatattaagaaattagaagttccattaaat

26. *HbTIP2;3*

1 tgtatataagcatgcttgcgggatatataactttcattctaatcgggtcttgagctatag
1 M P M I A V G S
61 ccaatagtgcaataacaactgaactggctagtgaag**ATG**CCGATGATAGCTGTTGGCAGT
9 I G D S F S I G S I K A Y L S E F I A T
121 ATCGGAGACTCTTTCAGTATTGGTTCCATCAAGGCCTATCTATCTGAGTTCATTGCTACT
29 L L F V F A G V G S A I A Y S
181 CTTCTTTTCGTTTTTGCTGGTGTTGGTTCTGCTATTGCTTACAgtaagtaaaataattat
241 gataataataattaaaaaaattatttagtcgtattgttaatgtaattaaattaataagaa
44 K L T A D A A L D P P G L V A V

```
301 ttttgtttttagGTAAGCTTACAGCAGACGCAGCTCTAGACCCACCTGGCCTGGTGGCTG
 60  A  V  A  H  A  F  A  L  F  V  G  V  A  I  A  A  N  I  S  G
361 TGGCCGTGGCTCATGCTTTTGCACTGTTTGTTGGGGTAGCCATTGCAGCCAACATCTCAG
 80  G  H  L  N  P  A  V  T  F  G  L  A  V  G  G  N  I  T  I  L
421 GTGGACACTTGAATCCAGCTGTCACCTTTGGATTGGCTGTTGGAGGCAACATCACCATCT
100  T  G  I  F  Y  W  I  A  Q  C  L  G  S  I  V  A  C  L  L  L
481 TAACTGGCATTTTCTATTGGATAGCCCAGTGCCTTGGCTCCATCGTGGCCTGTCTCCTCC
120  Q  F  V  T  N  G  K
541 TCCAATTCGTGACTAATGGCAAGgtatattagattagttattctcttattcaaacatatt
601 tactcagacaaatatttaaaataaaataaaaatttatatttccttcttttgattcggtgt
661 atatttaaagaacttatttccagtgacctttataaaaatgattaaatgaagaaaattctt
127              S  V  P  T  H  G  V  A  S  G  M  N  A  F  E  G  V
721 cgatttggcagAGTGTCCCAACCCATGGAGTTGCATCCGGCATGAATGCTTTTGAAGGAG
144  I  M  E  I  I  I  T  F  A  L  V  Y  T  V  Y  A  T  A  A  D
781 TAATAATGGAGATTATCATTACCTTTGCACTGGTGTACACGGTTTATGCCACAGCTGCTG
164  P  K  K  G  N  L  G  I  I  A  P  I  A  I  G  F  I  V  G  A
841 ACCCCAAGAAGGGCAATTTGGGAATTATAGCACCCATTGCAATTGGGTTCATAGTTGGTG
184  N  I  L  A  A  G  P  F  S  G  G  S  M  N  P  A  R  S  F  G
901 CAAACATCTTAGCTGCCGGTCCATTTAGCGGCGGATCGATGAACCCAGCCCGATCATTTG
204  P  A  V  V  S  G  D  F  S  E  N  W  I  Y  W  V  G  P  L  I
961 GCCCAGCTGTGGTAAGCGGAGACTTCTCAGAGAACTGGATCTACTGGGTTGGTCCATTAA
224  G  G  G  L  A  G  L  V  Y  G  Q  I  F  I  G  S  Y  V  P  A
1021 TTGGTGGAGGGCTGGCTGGGCTTGTTTATGGTCAAATTTTCATTGGGTCATACGTCCCAG
244  P  S  S  E  D  Y  A  *
1081 CCCCATCTTCTGAAGACTATGCCTAAattagctgttggcattttgcttgcttttgtgtct
1141 catctggtttcttacttttgggtgtgggcttgaagagagtgtgctttgtacttcataata
1201 atgaaaatgtaaataataaaatccaaattgagttttcaaatcttcttgttattat
```

27. *HbTIP2;4*

```
  1 M  A  R  M  A  F  G  S  F  G  D  F  F  S  I  G  S  I  K  A
  1 ATGGCGAGGATGGCTTTTGGCAGTTTTGGAGACTTTTTCAGTATTGGGTCCATCAAGGCT
 21 S  L  S  E  F  I  A  T  L  L  F  V  F  V  G  V  G  S  A  I
 61 TCTCTATCCGAGTTCATTGCCACTCTTCTTTTTGTGTTTGTTGGTGTTGGCTCAGCTATT
 41 A  Y  S
121 GCTTACAgtaagtggaattatttgtttgaaaattttatttagaagtattagtaatagaat
 44                                K  L  T  T  D  A  A  L  D
181 taaattaattaagtaaaataaaaaatttgtagGCAAGCTTACAACAGATGCAGCTCTAGA
 53  P  P  G  P  V  A  V  A  V  A  H  A  F  G  L  F  V  G  V  A
241 CCCACCAGGGCCGGTGGCTGTGGCGGTGGCCCATGCTTTTGGCCTGTTTGTTGGGGTAGC
 73  I  A  A  N  I  S  G  G  H  L  N  P  A  V  T  F  G  L  A  V
301 CATAGCAGCCAACATCTCAGGCGGACACTTAAATCCAGCTGTCACCTTTGGGTTGGCTGT
 93  G  G  N  I  T  I  L  T  G  I  F  Y  C  I  A  Q  C  L  G  S
361 CGGAGGCAACATCACCATCCTAACTGGCATTTTCTATTGCATTGCCCAGTGCCTTGGCTC
113  I  V  A  C  P  L  L  Y  F  V  A  N  G  K
421 CATTGTAGCCTGCCCCCTCCTATATTTCGTCGCTAATGGCAAGgtaattgagattaatta
```

481 ttctcttctttccggatacttaatttacacacacaatatttaacaataataaaataatta
541 tatttactttgatatatagttttatagaacttattgccaaagaacttgaaaggacaaaga
127 S V P T H G V A S
601 aaacgattaaatgaaaaatgattggatttggcagAGCGTCCCAACCCATGGAGTTGCTTC
136 G M N A F E G V V M E I V L T F G P L Y
661 AGGCATGAATGCTTTTGAAGGAGTAGTAATGGAGATTGTGTTAACTTTCGGACCATTGTA
156 T V Y A T A A E P E K G N L G I M A P L
721 CACAGTTTACGCCACAGCCGCTGAACCCGAGAAGGGTAATTTGGGAATTATGGCTCCCCT
176 A I G F V F G A N I L A T S P F S G G S
781 TGCAATTGGGTTCGTATTTGGAGCAAATATCTTAGCTACCAGCCCATTTAGTGGTGGCTC
196 M N P A S S F G P A V V S G D F S E N C
841 GATGAATCCGGCTAGTTCGTTCGGCCCAGCTGTGGTCAGTGGAGACTTCTCAGAGAACTG
216 I Y W L A H *
901 TATCTACTgGGTTGGCCCAT**TAA**

28. *HbTIP3;1*

1 acgcctacacttctcatccatgcaagaacccgatctctctcttcctttcctctctctttt
61 tctacgttttctttacctaaacaaagctctcaccaacttcgaaatccttccttgtagggc
121 ttggtgtgacatactgtagtagtctcagcgtgtgggattcaggttaatcaagaagaagag
1 M P R R Y A L G R A E E A T
181 ccaagtttatctagctaacc**ATG**CCTAGAAGATATGCACTTGGGAGGGCAGAAGAGGCCA
15 N P D S M R A A L A E F V S T L I F V F
241 CCAACCCTGACTCCATGAGAGCTGCATTAGCTGAATTCGTCTCCACTCTTATCTTCGTCT
35 A G E G S V L A L D
301 TCGCTGGTGAAGGCTCCGTCCTTGCTCTTGgtatatatttattccatctctttaatttg
361 aacagctcactttttctctctatataatgataaaggaaagggtcgatttgcttggtgatc
45 K L Y R E T E P P A S G L V M I
421 ttcctttgcagATAAATTGTATAGGGAAACTGAACCTCCGGCTTCAGGACTGGTGATGAT
61 A L A H A L A L F S A V S A S I N I S G
481 TGCGCTTGCACATGCATTGGCACTGTTTTCTGCTGTTTCAGCCAGCATCAACATATCAGG
81 G H V N P A V T F G A L V G G R I S V L
541 TGGTCATGTGAATCCTGCTGTTACCTTTGGAGCTCTCGTTGGAGGAAGGATCTCAGTCCT
101 Q A F Y Y W V A Q L L G A I V A S L L L
601 ACAAGCTTTCTATTACTGGGTTGCTCAGCTTCTTGGCGCTATTGTGGCTTCTCTCTTGTT
121 R L V T N G M
661 GAGGCTCGTCACTAATGGCATGgtatacaatacattcaactcaatacttttgaggatgtt
128 R P
721 acatataccttttcagaatttactggtaatgtaatattaattgatttttttgcagAGACCA
130 V G F Y I A S G V G E V H G L I M E M V
781 GTGGGATTCTATATAGCATCAGGGGTTGGAGAGGTGCATGGCCTTATAATGGAAATGGTA
150 M T F G L V Y T V Y A T A V D P K R G S
841 ATGACATTTGGACTGGTTTACACCGTGTATGCTACAGCCGTTGATCCCAAAAGGGGAAGC
170 L G I I A P L A I G F I V G A N I L V G
901 TTGGGAATCATTGCCCCTCTAGCCATCGGGTTCATTGTTGGAGCCAACATTTTAGTTGGG
190 G P F D G A S M N P A R A F G P A L V G

961 GGTCCTTTCGATGGAGCATCAATGAACCCAGCAAGGGCATTTGGGCCTGCCCTAGTTGGG
210 W R W R N H W I Y W L G P F V G G G L A
1021 TGGAGATGGAGGAACCACTGGATCTACTGGCTCGGTCCTTTCGTTGGAGGGGGCTTAGCG
230 A L I Y E Y T V I P T D P L P H H T H H
1081 GCACTCATATACGAGTACACGGTGATCCCAACAGATCCATTACCACATCATACTCATCAT
250 Q P L A P D D Y *
1141 CAACCTTTGGCTCCTGATGACTAC**TAG**tttattaatttacacgtccatgtgcttttgtgt
1201 tagccaacgattgtcttctccttggttgtctctatacttgtgtatctgtctgtgagaccg
1261 tcccttgaaccttgtgtgttgtgcgtcttatcatgtgctagcaactttgtgtatgatggt
1321 gcaaataaaaagtacgcctgac

29. *HbTIP3;2*

1 ctctctctctctcgctctctatttctttacctaatatcaaagctctcaccagtcaacaac
61 tttgaaatccttatttgtagatggtgtgacatactattctcagtgtgggaatcaagttaa
1 M S T R R Y A
121 ttaagaagatgtaccaagtttatcagatagctagctagct**ATG**TCTACTCGGAGGTATGC
8 F G N A E E A T H P D S M K A A L A E F
181 ATTTGGGAACGCAGAAGAGGCCACCCATCCTGACTCCATGAAAGCTGCCTTAGCTGAATT
28 V S T L I F V F A G E G S V L A L D
241 CGTCTCCACTCTCATCTTCGTCTTCGCTGGTGAAGGCTCCGTCCTTGCTCTTGgtatatt
301 tcattccatctctttaactatgaacaattctctttttctctctaaaaacggtaagtaaaa
46 K L Y R E T G P P
361 tggccgatttcttggtgatcttcctgtgcagACAAGTTGTATAGGGAAACGGGGCCTCCG
55 A S G L V M I A L A H A L A L F S A L S
421 GCTTCAGGTCTGGTGATGATTGCGCTTGCACATGCATTGGCACTTTTTTCTGCTCTTTCA
75 A S I N I S G G H V N P A V T F G A L V
481 GCCAGCATCAACATATCAGGTGGCCATGTGAATCCTGCTGTTACCTTCGGAGCTCTCGTT
95 G G R I S V L R A L Y Y W V A Q L L G S
541 GGAGGAAGGATCTCAGTCCTTCGCGCCCTTTACTACTGGGTTGCTCAGCTTCTTGGTTCT
115 I V A S L L L R L V T N R M
601 ATTGTCGCTTCTCTCTTGTTGAGGCTTGTCACTAACAGGATGgtatatcatacattcaac
661 tcttatagaggatgttatatatcttttcagaatttacaggtaatacaatatgaattgatt
129 R P V G F Y V A S G A A E V H G L I
721 tttgcagAGACCAGTGGGATTCTATGTAGCTTCGGGGGCTGCAGAGGTGCATGGCCTTAT
147 L E M V M T F G L A Y T V Y A T A V D T
781 ACTGGAAATGGTAATGACATTTGGACTGGCATACACTGTATATGCTACAGCCGTTGATAC
167 N R G S L G I I A P L A I G L I V G A N
841 CAACAGGGGAAGCTTGGGGATCATTGCCCCTCTAGCCATTGGGCTGATCGTTGGGGCCAA
187 I L V G G P F D G A S M N P A R A F G P
901 CATTTTAGTTGGTGGTCCTTTTGATGGAGCATCAATGAACCCAGCAAGAGCTTTTGGGCC
207 A L V G W R W R N H W I Y W L G P F I R
961 TGCCCTAGTTGGGTGGAGATGGAGGAACCACTGGATCTACTGGCTGGGTCCTTTCATTCG
227 G G L A A L I Y R I I S S A F G T *
1021 AGGGGGCTTGGCAGCGCTTATATACCGCATCATATCATCAGCCTTTGGCACC**TGA**agatt
1081 actag

30. *HbTIP4;1*

```
   1 aaaaatccttgaccttgttaggccaattgctcaattatgggtagacagacactgactgac
  61 acctaagtgcaagtgtaaaatgcaaagcagtagttgatttgtttttctccaagaaccata
 121 actttaatgatcatgctgtggaagctaaaggcttgcaagccagaagaagtcaacggccaa
 181 ggggtatccttgtaataatctctcgtatggcatgatgaccagctaaccccatatcctaat
 241 gaatccctcgtaatgcttatatatatcgttgtccttttgctctgcaaacccaagctttta
   1                                          M  A  K  I  A  L
 301 ctagtggaatttggtgtgattattccttaccaaaatcacccagaATGGCCAAAATTGCCT
   7  G  S  Q  R  E  A  T  Q  P  D  C  I  K  A  L  I  V  E  F  I
 361 TAGGATCTCAGCGAGAGGCCACCCAGCCCGACTGCATCAAGGCCCTTATCGTCGAGTTTA
  27  T  T  F  F  F  V  F  V  G  V  G  A  A  M  A  A  D
 421 TTACCACCTTTTTCTTCGTCTTCGTCGGAGTCGGAGCAGCCATGGCCGCTGgtgcatcat
 481 ttctttctttttaagcatttgtttgcaattatttatgtgaaattaaagctcgatcgtat
  44                                       K  L  V  G  G  S  L  A
 541 tatttaagagagattgatgttgtgcacatgaacagATAAACTAGTGGGAGGATCTCTAGC
  52 G  L  L  F  V  A  L  A  H  A  L  V  V  A  V  M  I  S  A  G
 601 GGGCTTGCTTTTCGTGGCTTTAGCACATGCACTTGTTGTGGCTGTAATGATATCCGCCGG
  72 H  I  S  G  G  H  L  N  P  A  V  T  L  G  L  L  A  G  G  H
 661 TCACATCTCCGGTGGTCATCTCAACCCAGCAGTCACTTTGGGCCTCCTCGCCGGCGGTCA
  92 I  T  V  F  R  S  I  L  Y  W  I  D  Q  L  V  A  S  S  A  A
 721 CATCACCGTCTTCCGATCCATCCTTTACTGGATTGACCAATTGGTTGCATCTTCTGCTGC
 112 C  L  L  L  S  Y  L  T  G  G  M
 781 TTGTCTCCTCCTAAGCTATCTCACAGGAGGAATGgtaagtctacttatttgttactctct
 841 ttgaccacagaatctcctattttaatcaaatcttttgatcttccctgcatttattttggt
 901 gtcttgttcttaattaaggtcatagtacatgtcattagctggtgggtgtctttgtattct
 123    A  T  P  V  I  T  L  A  S  G  V  G  Y  V  Q  G  V  V  W
 961 gcagGCCACTCCTGTGATTACGCTGGCAAGTGGGGTAGGCTACGTTCAAGGGGTAGTGTG
 142 E  I  L  L  T  F  S  L  L  F  T  V  Y  G  T  I  V  D  P  K
1021 GGAGATCTTACTGACCTTCTCCTTGCTGTTTACCGTCTATGGCACTATTGTGGACCCCAA
 162 K  G  S  I  D  G  L  G  P  L  L  T  G  L  V  V  G  A  N  I
1081 GAAGGGATCGATTGATGGGCTGGGCCCATTGCTCACTGGGCTCGTAGTTGGGGCAAACAT
 182 L  A  G  G  S  F  S  G  A  A  M  N  P  A  R  S  F  G  P  A
1141 CTTAGCTGGTGGATCATTTTCTGGTGCGGCTATGAACCCTGCACGATCCTTTGGGCCCGC
 202 L  V  S  W  D  W  T  H  H  W  V  Y  W  V  G  P  L  I  G  G
1201 TTTGGTGAGCTGGGACTGGACTCACCATTGGGTTTACTGGGTAGGACCCCTAATTGGTGG
 222 G  L  A  G  F  I  Y  E  N  F  F  I  T  R  S  H  L  P  L  P
1261 TGGGTTGGCTGGGTTCATCTATGAAAATTTCTTCATCACAAGATCTCATCTTCCTCTTCC
 242 N  D  E  E  T  Y  L  S  T  N  *
1321 CAATGATGAAGAAACTTACTTAAGTACTAATTAGcaattgcatcatgtaatgtagtcctt
1381 taatttagccactccttttcagtgtattaaattcctgcgtgtttccctgttattcaatat
1441 catgcatgtggtactttgattgatatgccactctttgttattagttttatctgaatcatt
1501 tttcaacaatttttcaccttttcatattagttgatttaaactccctccttcttattatca
1561 tcataaggtattagcatgaaaccccatggcaacaggtaacataagtgttgggattac
```

31. *HbTIP5;1*

1 M A P S S L N A R F K Q S V T P D A L R
1 **ATG**GCCCCATCATCTCTAAATGCCCGATTCAAACAATCTGTTACTCCCGACGCTTTAAGA
21 S Y L A E F I S T F F Y V F E V V G S A
61 TCATATCTCGCAGAGTTCATCTCCACTTTCTTTTATGTGTTTGAAGTTGTAGGATCTGCA
41 M A A R
121 ATGGCTGCACgtatgtcctaaataacaatttataagccaacccatttatttttatctttt
181 atgcaattggatattattgatttttcatgtttcttgtagcatgcatgagccgcttgactc
241 tcaacttctttatgtgaaactaattaacatgtagggtgggaatttcgtgacagtttgaaa
301 ttttgattttggtgtttgtttctttggtttgaggagttggcaaacatttctcttcttgtc
45 K L
361 attagagtaataaatattccaaagagttgatatttgattctgtcattacacagGGAAATT
47 M T G A D P S S L V I V A I A N S F A L
421 GATGACAGGAGCAGATCCATCCAGTTTAGTAATAGTTGCTATTGCGAATTCTTTTGCACT
67 S S A V Y I A A N I S G G H V N P A V T
481 TTCGTCGGCTGTGTACATCGCTGCCAACATCTCCGGTGGACATGTGAATCCTGCCGTCAC
87 F S L A V G G H I S V P T A L F Y W I S
541 ATTTAGTCTGGCCGTTGGAGGCCACATTAGCGTCCCTACTGCTCTATTCTACTGGATTTC
107 Q M L A S V M A C L L L R V V I V G Q
601 TCAAATGTTAGCCTCTGTCATGGCTTGTCTTCTCTTGAGAGTAGTCATTGTTGGACAGgt
661 aatgaaatctttccacatttctacacaagacattcatagtgttgatccttttatcctaat
721 tgcagttgtggactgattttgttgtggactgatttttatgcaatctactgtctcactttt
126 S L P T Y T I A E E M T G F G
781 gtatttgaattttgcagAGTCTTCCTACCTACACAATTGCAGAAGAAATGACAGGATTTG
141 A S V I E G V L T F G L V Y T V Y A A G
841 GAGCGTCGGTGATAGAAGGTGTGCTAACATTTGGTTTAGTATACACTGTTTATGCCGCAG
161 D P R R S L Q G V T G P L A I G L M A G
901 GGGACCCCAGGCGCAGCCTGCAGGGAGTCACCGGACCCTTGGCAATAGGACTGATGGCAG
181 A N V L A A G P F S G G S M N P A C A F
961 GAGCCAATGTGTTGGCTGCAGGACCCTTCTCAGGTGGTTCAATGAACCCTGCATGTGCAT
201 G S A V I A G R F K N Q A V Y W V G P L
1021 TTGGTTCTGCAGTCATTGCTGGACGGTTCAAGAATCAAGCAGTCTACTGGGTTGGACCCT
221 I G G T F A G L L Y D N A V F P N Q V P
1081 TGATTGGAGGCACATTTGCAGGGCTTCTATATGATAATGCTGTCTTCCCTAATCAAGTTC
241 D S I R G I S D G V G A *
1141 CTGATTCTATTAGGGGAATTTCAGACGGTGTTGGAGCG**TAA**

32. *HbTIP5;2*

1 M A P
1 gttctctttagccataaaatccatttatcattcttctgtttttcttggcaaaa**ATG**GCCC
4 T S L N D R F K Q S V T P D A L R S Y L
61 CGACATCACTAAATGATCGTTTCAAGCAATCTGTTACTCCCGACGCTTTAAGATCATATC
24 A E F I S T I F Y V F V V V G S A M A S
121 TTGCAGAGTTCATCTCCACTATTTTTTATGTGTTTGTAGTCGTTGGATCTGCCATGGCTT
44 D
181 CAGgtatgtctccggtaataatttcttctatttatgcaaatgggattattgatttttca

241 tctttcttgtagcataacacgagtcttagtgattctcgttgttgatttacaacttcacta
301 aatataaaactagcaagtagagtgggaatttggtagtggtttccttgttcgaggatttga
361 tatgctaattctattttctaagaatgcatttctcttcttgctaacatttccaatgagtat
45 T E K L M P G A D P S S L V
421 gcttgattcttccaagATACAGAGAAATTGATGCCAGGAGCAGATCCATCCAGTCTGGTA
59 I V A I A N A F A L S S A V Y I A A N V
481 ATAGTCGCCATTGCTAATGCTTTTGCGCTTTCATCGGCTGTGTACATTGCTGCCAACGTC
79 S S G H V N P A V T F S L A V G G H I N
541 TCCAGTGGGCATGTGAATCCTGCTGTCACATTTAGTCTGGCCGTTGGAGGCCACATTAAT
99 V P T A I F Y W I S Q M L A S V M A C L
601 GTCCCCACTGCTATATTCTACTGGATTTCTCAGATGTTGGCTTCTGTCATGGCTTGCCTT
119 L L R V A I V G Q
661 CTCTTGAGAGTAGCCATTGTTGGACAGgtaatgaaatctccctgcgtttctgcaaaaaat
721 atttataatttttatcctttctcccaattgtgattatggactaattttcttgcaatctgt
128 S L P T Y T I A A E M
781 tgtatgactccagtatttaaattttgcagTCTCTTCCTACCTACACTATTGCAGCAGAAA
139 T G F G A S V F E G V L T F G L V Y T V
841 TGACAGGATTTGGGGCGTCAGTGTTTGAAGGTGTGCTAACATTTGGTTTAGTCTACACAG
159 Y A A G D P R C S L L G A T G P L A I G
901 TTTACGCTGCCGGCGACCCTAGATGCAGTTTGCTGGGAGCCACTGGACCCTTGGCAATAG
179 L M A G A N V L A A G P F S G G S M N P
961 GGCTCATGGCAGGAGCCAATGTCTTGGCCGCAGGACCATTCTCAGGTGGCTCAATGAACC
199 A C A F G S A V I A G R F K N Q A V Y W
1021 CTGCCTGTGCATTTGGGTCTGCAGTCATTGCTGGAAGGTTCAAGAATCAAGCTGTTTACT
219 V G P L I G G T V A G L L Y D N V V F P
1081 GGGTTGGACCCTTAATTGGAGGCACAGTTGCAGGGCTTCTCTATGATAATGTTGTCTTCC
239 S Q V P D S I G V *
1141 CTAGTCAAGTTCCTGATTCTATTGGGGTG**TAA**ttgcaatttcgtaaaatgaaatgttact
1201 ttgttatttttggtatgtagttctttttccttcattttgcgacatattgcaattgta
1261 ttagccaacatttgtatgattgagtgaccaca

33. *HbNIP1;1*

1 cataacccttggtaggctacaaagttatctaactaagcaatacatatctttttctctctt
61 atttgctctctttcttattatcagatcgtgtgtggtctattttagtcacaaaagaactag
1 M A D E I S G T N G K H G V V L D V E G
121 tc**ATG**GCTGATGAGATCTCAGGAACTAATGGTAAGCATGGGGTTGTGTTGGATGTTGAGG
21 D N P C Y S P P P C N D R P P C A S K T
181 GTGATAATCCTTGTTATTCTCCTCCACCTTGTAATGACCGTCCACCTTGTGCTTCCAAAA
41 K E D S I L S I S V P F I Q K
241 CCAAGGAAGATTCAATCTTAAGCATCTCTGTACCTTTCATTCAAAAGgtgacatattgcc
301 ctatcaatttcagtgttacttcttttcttggttattgtatcagttcaattatgtatcctc
361 ttatgaaaacttgaatagtttcaaggctgtgtgtggccattaaagactgaaaaatatgtg
56 L I A E V V G T Y F L
421 attttgacgttgatgaatgatgtgcagTTGATAGCTGAGGTGGTAGGCACGTATTTCTTG
67 I F A G C T A V A V N L N F D K E V T L

481 ATATTCGCCGGATGTACAGCGGTGGCGGTGAATTTGAACTTCGACAAGGAGGTGACACTT
87 P G I S I V W G L A V M V L V Y S V G H
541 CCAGGAATATCAATAGTTTGGGGATTAGCTGTGATGGTCTTGGTTTACTCTGTTGGTCAC
107 I S G A H F N P A V T L A F A T C K R F
601 ATCTCTGGTGCCCATTTCAACCCTGCTGTCACTCTTGCCTTTGCCACCTGCAAGAGATTT
127 P W K E
661 CCTTGGAAAGAGgtaaaaccaaactcaaactacccagtttaaaagagttaaactaggcat
721 acacatatgatgagaaacctttaggtgtaagaaactgtatcaatcaagctgttgaactga
781 gctcaagcagaagatggatatatgcattgatattctttcccttttcttatttggtcatgt
131   V P A Y I A C Q V I G S T L A A G T I
841 cgtagGTGCCTGCTTATATAGCATGTCAAGTCATAGGATCAACACTAGCAGCTGGAACAA
150 R L I F T G K Q D Q F T G T M P A G S D
901 TTCGATTAATTTTTACAGGGAAGCAAGACCAATTCACAGGAACAATGCCAGCAGGATCAG
170 M Q S F V V E F I I T F Y L M F I I S G
961 ATATGCAATCCTTTGTAGTTGAGTTCATAATCACATTTTATCTCATGTTTATCATATCAG
190 V A T D N R A
1021 GCGTCGCTACCGATAACCGAGCTgtaagttattattcttcatctaatcattgattataga
1081 tttagaatccaaaagcttaatacaggatattgtggtaagccaattgagttagatatataa
1141 tgtctaattaagctaccaaattattgacctatgataacttcttagcatattcattggaat
1201 tttaattatttaattgtgttgaaagtgattgctgaattatttgattctgatgtttatgca
197 I G E L A G L A V G S T V L L N V L F A
1261 gATTGGTGAACTTGCTGGACTTGCTGTAGGTTCTACAGTTCTCCTAAATGTGCTTTTTGC
217 G
1321 AGGgtattaacctaacagagtttccttataatgcttaatctaatcaagcactaatcaata
1381 ttatccaaaatcattggctagtgatgaaatgggtaacataaattaatttatttaattgca
218   P I
1441 tttaaaaatagatacccttctgatggatttgccgatgtcattttgcaaaacagGCCAATT
220 S G A S M N P A R S L G P A I V S S Q Y
1501 TCAGGGGCATCAATGAACCCAGCAAGAAGCTTGGGGCCTGCAATTGTATCATCTCAATAC
240 K G I W V Y L I S P I L G A Q A G A W A
1561 AAGGGGATATGGGTTTATCTTATATCGCCGATTCTTGGTGCTCAAGCAGGTGCATGGGCG
260 Y N M I R Y T D K P L R E I T K S A S F
1621 TATAACATGATTAGGTATACAGATAAGCCTCTGAGAGAGATCACCAAGAGTGCTTCATTC
280 L K S T G R A *
1681 CTCAAGAGTACAGGACGTGCT**TGA**taagaaagtaatctttttcggcctaccatatttca
1741 tgtaaagctagaaagattttctgtaattttccca

34. ***HbNIP1;2***

1 tgcatattgtcgatctctgcccttttatttgtcttcttttggatctttctgcacttataa
61 atagtaccacaacatgtttttttccaagtttcttgcgtaacccctaggtgggcaacaaa
121 gctagccaactaagcaaccagaaccagcaaacatagggaaaaagaaaagaaggaatttct
181 ttcttctttattcacatctttgcctcccttatttactcgcttctcattctcagattgtct
1   M A Q A E I S V A N G R
241 tttactttgcaagaagaaactagt**ATG**GCTCAGGCTGAGATCTCAGTAGCTAATGGCAGG
13 H G V V L D V K D D N P C N P A P A G N

301 CATGGAGTTGTATTGGATGTTAAGGATGATAATCCTTGTAATCCTGCTCCAGCTGGTAAT
33 K P P P C A S K T K E D S I S S I S V P
361 AAGCCTCCTCCTTGTGCCTCCAAAACTAAAGAAGATTCAATCTCAAGCATCTCCGTACCT
53 F I Q K
421 TTCATTCAAAAGgtcagtgtttcttcttttcttggtcccggctttcttacccaaaaacaa
481 aaaaagccttttgaatcaaacccatttcataaaatttgatttcttgcttgccaccgattg
541 tatctggccaattgtatgtcctcttgatgaaaaattgaatagtttttttgaagggtatatg
601 aagctttatttagccattaaagactgaaagttttgcttttgaattgaattcaaatggaaa
57 L I A E M V G T Y F L I
661 ttttgtgttgatgaatgatgtgcagTTGATAGCTGAGATGGTTGGCACTTACTTCTTGAT
69 F A G C T S V A A N L N Y D K V V T L P
721 ATTTGCCGGATGTACATCGGTGGCGGCAAATTTGAACTACGACAAGGTGGTGACACTTCC
89 G I S I V W G L A V M V L V Y S V G H I
781 AGGAATATCAATAGTTTGGGGATTGGCGGTGATGGTCTTGGTTTACTCTGTTGGTCACAT
109 S G A H F N P A V T L A F A T C K R F P
841 CTCTGGTGCCCATTTCAACCCGGCTGTCACTCTTGCCTTTGCCACCTGCAAGAGATTTCC
129 W K Q
901 CTGGAAACAGgtaaaaccaaactaaatctactttgttgaaaattggttaactacacttgc
961 gggaaacctttaggggtaagaaattgttgcaataaaaaaattggtttatttttgtttatt
1021 gaaatttctcacttgtttgtaataagaattgtgttcacatgtacccctcagcattttctg
1081 aatgctaattaagtacatttgaccctccccagagctgacaatgcaggatgcaggaggcct
1141 ggtcaactggagcacttgaactcatttgtttcgaaatgtttatttgggattattatagaa
1201 tctactttgtgttacaaagtgtaaaagtaaacagcattatgaggttgaaatcaattcaat
1261 caagctgttgaactgagctccggtagaaggttatgtattgatattctttttccccccttt
132 V P A Y I A C Q V T G S
1321 cccctcttcttgtttggtcatgtagGTTCCAGCTTATATAGCATGCCAAGTCACTGGATC
144 T L A A G T I R L I F T G K Q D Q F T G
1381 AACACTAGCTGCTGGAACAATTCGATTAATTTTTACTGGGAAGCAAGATCAATTCACAGG
164 T L P A G S D M Q S F V V E F I I T F Y
1441 AACATTGCCAGCAGGATCAGATATGCAGTCCTTTGTAGTTGAGTTTATAATCACATTCTA
184 L M F I I S G V A T D N R A
1501 TCTCATGTTTATCATATCAGGTGTCGCTACCGATAATCGAGCTgtaagttattattcttc
1561 acctaatcattgattctagacttagaatacaaaaacttaataaagaataatgtggcttgc
1621 caatagacttggctaatccgtactcgctgattaagttaccaacttattcacctattataa
1681 ctttttagtgtattccttagaatttgaattattttgattgtgaaaagtgattgctgatg
198 I G E L A G L A V G A T V L L N V
1741 tttatgcagATTGGTGAACTTGCTGGACTTGCTGTTGGTGCTACGGTTCTCTTAAATGTG
215 M F A G
1801 ATGTTTGCAGGgtatccacctaatcaagtttgcttagaatacgtaatcaaattaagcaat
1861 aatcaatattatccaaaatctaaatcgttgactagtgatgaattgtttgcttgtatgaca
1921 tataagttaattatttactaatgaaaataaataccctttttgatggttttgctgatatcat
219 A I S G A S M N P A R S L G P A
1981 tttgcaaatcagCGCAATTTCAGGGGCATCAATGAACCCAGCAAGAAGCCTGGGGCCTGC
235 I V S S Q Y K G L W I Y I V S P I L G A

2041 AATTGTATCAAGTCAATACAAGGGGCTATGGATTTATATTGTGTCGCCAATACTTGGTGC
255 Q A G A W V Y N M I R Y T D K P L R E I
2101 TCAAGCAGGTGCATGGGTCTATAACATGATTAGATATACAGATAAGCCTCTGAGAGAGAT
275 T M S A S F L K N T G R A *
2161 CACCATGAGTGCTTCATTCCTCAAGAACACAGGACGTGCT**TAA**caagaaaatgatttctc
2221 ttactggctctttatttcatgtaatctaatacaagataagacctttaattcctgtatctg
2281 aaggatattctgtaatttttcctgctcatcaatgcaatagaaagatttccattagtagtc

35. *HbNIP2;1*

1 atttttcctcgttccacttgtatataaacaatcttttctagctcaataaacaggactatt
61 cattaattctcataatttcttctctgatatatatttctcttgctctgcttctatattct
121 ctgttcagctgaagggtatattagcaacttgtgttctcggcgttaacttggtatctttta
181 ttggttctctccctcctatgtatatttcacttttcccagtcctttttttacatttttccgt
1 M A T I D P N P N
241 caattttgcaggcttctgttcgatcaagaaaaa**ATG**GCGACAATCGATCCAAATCCCAAC
10 N S A S I G D G L V S V E N P K S Q L V
301 AACTCAGCTTCCATAGGAGATGGTCTGGTCTCTGTGGAGAACCCAAAGTCCCAACTTGTT
30 E S F G K N Y P P D F L K K
361 GAATCATTTGGAAAAAATTATCCTCCTGATTTTCTCAAAAAGgtaacatcattttgtttt
421 tcttgaacatgaacaggatgaggtagttaattaatttccaaggaaacaaacacatagaaa
481 agaaaaaagatgcatataaaagtgaatacagtaccaaataaagtttcaaaaacatactga
44 V V A E V I A T Y L L V F V T C G
541 gcaacgcagGTGGTGGCAGAGGTCATAGCTACCTATTTGTTAGTATTTGTGACATGTGGT
61 A A A I S T S D E R R I S K L G A S V A
601 GCCGCTGCAATTAGTACAAGTGATGAACGAAGAATCTCAAAACTAGGAGCCTCAGTAGCA
81 G G L I V T V M I Y A V G H V S G A H M
661 GGAGGGCTTATAGTGACAGTGATGATCTATGCAGTGGGACATGTCTCTGGTGCTCACATG
101 N P A V T T A F A A F R H F P W K Q
721 AACCCTGCAGTGACTACAGCTTTTGCAGCTTTTAGACATTTTCCATGGAAGCAGgtaaaa
781 aaacaaaaaccatgatctccatttgctatgtaagagttatttggtaattttgttcaattt
841 tcttgtttgctttggggctttgtggttatgtttcatcgatcagtagacaatataaagcca
901 cccacatatgatgacgggcttagagtatggatatgacatagtaataatctgaccccatag
961 aaatagttttcatcgcttcaggttttctggcagccacttgtggccacaaaatttaaataa
1021 atagcatatgtgagaaaattagtctattttggcatcggtagcccgtaataaatgtattta
1081 ttatgctataaacataagatgtgtaatagaatctttttattaaatgcataaattttatgt
1141 atttatgttttaaatttatatagattaaatttagacaatttttcttccctctaatctctt
1201 ttaatttcagagagatttaaaattaaaaaaaaaaaattagatcggaaaaaggatagtggg
1261 cacaatatgaggaaagagaatctttttggagggacatgcaagtaggcaaaattatattct
1321 catctgaccacatagtaggcatcaataggtcataaaaaaagagagccggccgtgaaaagt
1381 ctatttcttaagtgatttaattaatattttgtgtacataatattttattgttaaatgagt
1441 gtacatttcactatgtcacataataaagtgataaataaaacatcattgtcctgagattaa
1501 attagaagaaaaaccttcaataatttagattaatttagataatgggacactatagccaac
1561 tgagaaaaacccatttctaagtggtttaattaatattttgtgtacataatattttattg
1621 ttaaatgagtgtacatttcactatgtcacataataaagtgataaataaaacatcattgtc
1681 ctgagattaaattagaagaaaaacctttaataatttagattaatttagataatgggacac

1741 tatagccaactgagaaaaacccattttctaagtggtttaattaatattttgtgtacataa
1801 tattttattgttaaatgagtgtacatttcactatgtcacataataaagtgataaataaaa
1861 catcattgtcctgacattaaattagaagaaaaacctttaataatttagattaatttagat
1921 aatgggacactatagccaactgagaaaaacccattttctaagtggtttaattaatatttt
1981 gtgtacataatattttattgttaaatgagtgtacatttcactatgtcacataataaagtg
2041 ataaataaaacattattgtcctgagattaaattagaagaaaaacctttaataatttagat
2101 taatttagataatgggacactaccaaatcttagttcatcttctaaaagagaaaatgggtg
119 V P F Y A A A Q L T G A I S
2161 gatttatttttggttgcagGTACCATTTTATGCAGCAGCTCAACTAACAGGAGCAATTT
133 A S F T L R V L L H P I K Q V G T T S P
2221 CTGCTTCATTTACATTGAGAGTGCTCCTTCACCCTATAAAACAAGTTGGAACCACTTCAC
153 S G S D L Q A L I M E I V V T F S M M F
2281 CTTCTGGGTCAGATCTTCAAGCTCTCATCATGGAAATTGTAGTAACATTTTCTATGATGT
173 V T S A V A T D T K A
2341 TTGTAACTTCAGCTGTGGCAACTGATACAAAGGCTgtaaggaacatcctaaattttgtca
184 I G E L A
2401 tattttgccttttctttttttttttaattattttttttctgcagATAGGAGAGTTGGC
189 G V A V G S A V C I T S I L A G
2461 AGGTGTAGCAGTTGGTTCTGCAGTGTGTATAACATCCATCTTGGCCGGgtaagccatacc
2521 taacagaagaaattaatgggtctagatcttcaatttctaacaaacatttattaaataaat
2581 aaaataattaacttgggtgtataaatgaaaccaaaaatttcagtcaggaatgcctctacc
2641 aagaccagaacttgatgtagatctggtcaggaattggctcgaaaccgaaattagagtgga
2701 actggatcacttcaaattgaaattcaaaccagttaaaatcagatcgattaatattaatat
2761 tggatcaaaccgatttttttcttttaaaataaaaataaaaaattatttcttaaattaga
2821 acaaaatgggaatcctaaaattggaaaaaccccagagtcattctaagtttttaaccctga
2881 atcggcccttggccaccgtagtacgatacacacaatccacaattataaaactaaccatcc
205 P V S G G S M N P A R S L G P
2941 gtctagtgatcctcagCCCAGTATCAGGCGGATCTATGAACCCAGCAAGGTCGCTAGGGC
220 A I A S A Y Y K G I W V Y I I G P V V G
3001 CAGCAATTGCTAGCGCATACTACAAGGGGATATGGGTCTACATTATTGGACCAGTGGTGG
240 T L L G A C S Y N L I R V T D Q P I Q A
3061 GAACACTGCTTGGAGCATGTTCTTACAACCTGATTCGAGTGACTGATCAGCCAATCCAAG
260 I S Y S L K L R R I R S N D E Q A H N K
3121 CAATTTCTTATTCACTCAAACTTCGCAGAATCAGAAGCAATGACGAGCAGGCTCACAACA
280 D P F D A L *
3181 AAGACCCCTTTGATGCTCTT**TGA**agtttctaagggctaccattataatatcacagggaat
3241 agaaagcatgatgtaacaagtaagtatgcttcatttttgtggagctcacttgtagtatct
3301 ttacaaaaaccaagcttgtgtgcctgtaatatattatatgatgtgtagtagatgtaaaaa
3361 cagattgcctgtgaggcaccactatcatatctacctaatatcagtcacgcttgtttatga
3421 tatctatgtgtgtgcactagaacttgctgcatactactgggcattaaatatttcaaa

36. *HbNIP3;1*

1 ctagattccatattgccaaattgcttactggttcggtcagagttaaacgcatacatccat
61 ggcattttccttcacagctctttcacgttcaagctaatcatagtataagaacatgtgaag
1 M A S P

121 tgggtggctacaaagctattcccaatttggtttagttcctaagtgctgca**ATG**GCAAGCC
5 N S I T S V V S P K P Q L P T K Y S V A
181 CCAATTCTATCACTAGTGTAGTCTCCCCTAAACCTCAACTGCCTACTAAATACTCAGTGG
25 A E A K A S R S R E W F L T D D G S P S
241 CTGCAGAAGCAAAGGCAAGTCGTTCCCGCGAATGGTTCCTCACTGACGATGGTTCTCCAT
45 V L Q K
301 CTGTTCTTCAAAAGgtggccaaatttcatagttaaaagtctgatttttcatgacaaaaat
49 I I A E L
361 atggttatctttcttagaaaagatatttgtttctttggtttagcagATAATTGCCGAGCT
54 I G T Y I L I F V G C G A A L T D K V Q
421 GATAGGTACATATATTCTTATATTTGTGGGCTGTGGTGCTGCCCTGACCGACAAAGTTCA
74 K L T I V G I A I A W G V V L M A A I Y
481 AAAACTGACAATTGTGGGTATAGCAATTGCGTGGGGTGTAGTTCTGATGGCAGCAATTTA
94 A L G H V S G A H F N P A V S I A L A A
541 TGCACTTGGGCATGTCTCTGGTGCACACTTTAATCCTGCAGTTAGCATTGCCTTAGCTGC
114 A R K F S W K N
601 TGCACGGAAATTTTCTTGGAAAAATgtgagacattttcacttagatgtgtatcgtgtaaa
122 V P M Y I L
661 agaaacaaaacagaatatgattcacacggtattgtttgtgtagGTACCTATGTATATCCT
128 A Q V L G A T L A C L T L K V L F H D Q
721 GGCTCAGGTACTGGGAGCGACACTTGCTTGTCTTACCCTTAAAGTGCTGTTCCATGACCA
148 D D I Q A T M T Q Y K D S T S D L E A F
781 AGATGATATTCAAGCAACAATGACTCAGTACAAGGACTCAACCTCTGATCTTGAAGCTTT
168 I W E F I I T F N L M F N I C G V A T D
841 CATATGGGAATTTATAATCACGTTTAATTTAATGTTCAATATTTGTGGTGTTGCTACAGA
188 H R G
901 TCATAGAGGGgtatgatcacattttattcagttgattttattacttgcttaaaagaccat
191 S K
961 gcctagctctatctaatctgaagaatcctctacatgattgaccaaacaaaccagAGCAAA
193 D L S G V A I G G T L L F N V L L A G
1021 GACCTTTCTGGTGTAGCAATTGGCGGTACGCTGCTATTTAATGTCTTGCTTGCTGGgtaa
1081 ataaactcactccctcactaatcactgctttaaactgtgctctattccagcacttcatca
1141 ttacttaccaccacttgtaaatattcttaagcttggattcctttattcctcaaattctgc
212 P I T G A S M N P A R S L G P A I V S
1201 agACCGATCACTGGAGCTTCAATGAACCCTGCAAGAAGCTTAGGCCCTGCAATTGTCTCA
231 G V Y K N L W V F I V S P I L G A L A A
1261 GGTGTTTACAAGAATCTTTGGGTCTTTATCGTGTCCCCTATCCTTGGAGCTTTGGCCGCA
251 A L I Y S M L R V P N P E K P E E K N K
1321 GCTTTAATATACAGCATGCTTCGGGTGCCTAACCCTGAAAAACCTGAGGAGAAGAACAAA
271 I V L N Y L Y S P A E P *
1381 ATCGTACTTAATTATCTTTATTCACCAGCTGAGCCT**TAG**acaggtatgcttccacagctt
1441 cagatggtgaagtattttaggaccatggaaaatcactttgctcatgcctgaagcacaaga
1501 aattattcctaactgcatctatgctcaaaaagtttaggaggattgaagagagagagagag
1561 agagtcttctgccaatatgcatggcagaaaagataaaaatgaaaggaaagaaaacatgtg

1621 tcagcagtttgccccttcatgcataaaatatgactgcaataaaaagggggaaaattgtat
1681 aaatcaaaacggttcatctttttattattccctttatctgcaatttaagaaatgtgcggt
1741 ggtaaatgcatccttttttttttttcattctactaaaaaaagttaatttcca

37. *HbNIP4;1*

1 M S G A D D H E I R D V E E G Q Q E D S
1 **ATG**TCCGGAGCAGATGATCATGAGATCAGAGATGTAGAAGAAGGGCAACAAGAAGACAGT
21 S D S Q N N S K R S G F C S S N A T
61 AGTGATTCTCAAAATAACAGTAAAAGATCAGGGTTTTGTTCATCAAATGCGACAgttcaa
121 ctgctccagaaggttttacagagcctttttttttttccttttcttttggttggttgacaa
39 L I A
181 atatatcatagacattaagaccaagcgaatatgatctttctttcttgaacagCTGATTGC
42 E M V G T Y M L I F C G C G S V A V N Y
241 AGAGATGGTGGGGACATACATGTTGATATTTGTGGTTGTGGATCAGTTGCAGTCAATTA
62 I Y G S I T F P G V C V V W G L I V M V
301 CATATATGGTTCTATTACATTTCCGGGAGTATGTGTAGTTTGGGGTCTTATTGTGATGGT
82 M I Y S V G H I S G A H F N P A V S I T
361 TATGATTTACTCCGTTGGTCATATCTCTGGTGCTCATTTCAATCCTGCAGTTAGCATCAC
102 F A I F R Q F P I K Q
421 TTTTGCCATTTTTCGACAATTTCCGATCAAACAGgcaggtttccggctcgatccttttgt
481 tccgtcaggaatatttcagttctttttttcttgttctgatatgctattgctacatgaattt
113 L P L Y I L A Q F V G S L L A S G T
541 ttgcagCTACCTTTATACATTCTGGCACAGTTTGTTGGGTCACTTCTTGCTAGCGGCACA
131 L Y I L L D V E D E D F F G T K P V G P
601 TTGTACATCCTGCTTGATGTGGAAGATGAGGATTTCTTTGGGACAAAACCAGTAGGGCCT
151 H G R S F V I E L I T S F L L M F V I S
661 CATGGTCGTTCTTTTGTAATTGAATTAATCACCTCCTTCCTCCTAATGTTCGTCATCTCT
171 G V A T D N R A
721 GGCGTCGCAACGGATAATAGAGCAgtcagtaacccttaaacatcatttcatttttggtta
781 ttttctttgccattaaatgtgttttcttcttcaagatatctctctcatttggttccaact
179 I G E L A G I
841 atttctgttaaggaaaactgaaattgtcaacacttttgcagATTGGAGAATTAGCAGGAA
186 A I G M T I M L N V F I S G
901 TTGCTATTGGAATGACAATAATGTTAAATGTCTTCATTTCAGGgtacattaatgcgttct
961 tgaagtattttttttttttttttttgagtttcatcaattaacagatttttctaattttgt
200 P V S G G S M N P V R S L G P A I
1021 gggctgcagGCCAGTATCGGGGGGATCCATGAACCCTGTTCGGAGCTTAGGGCCTGCTAT
217 V M H I Y T G I W I Y I I G P V I G A I
1081 TGTTATGCATATATATACAGGAATCTGGATTTATATAATCGGGCCGGTCATCGGAGCCAT
237 L G G F C Y N L I R F T D K P L R E I S
1141 CCTGGGAGGCTTTTGTTACAATTTGATTAGATTTACAGATAAACCTCTCCGTGAAATAAG
257 K S S T L I N S F R S *
1201 CAAGAGTAGTACATTAATCAATAGTTTCAGGAGT**TAA**tcaagaccagttcattatcaacc
1261 ttttaattagcaataggcttcattccatcatttgttgccccatatgtacaaatatttcat
1321 tacgggtatcctaatactggatattgtcaaaagcacaggacacataacacgtgaaatgtt

1381 tgagaagaggccattttgctttgaggaattccacctgttccctgctttgcttcacaattg
1441 ttgcctattaatttggatattccttgaatggcccatgtg

38. ***HbNIP4;2***

1 M A T A H A D I I E E E E E E V S K I E
1 **ATG**GCCACAGCGCATGCAGATATAATTGAAGAGGAGGAGGAGGAGGTTTCCAAGATTGAA
21 Q G L P P S T T A D A S A N N T V G P C
61 CAAGGCCTACCCCCATCTACCACCGCTGACGCAAGTGCTAACAACACTGTTGGTCCTTGC
41 L S T S F V S I T Q K
121 CTATCAACTTCTTTTGTGAGCATCACACAAAAGgtcagttagacctatttaattatttgt
181 ttaattttcatatgtagttttgcatatgcagatagccaatgtgttcgttgggtattgctt
52 L I A E V I G T Y F V
241 gtgggtatttattatttatttttcagCTGATAGCTGAGGTTATTGGCACCTATTTTGTG
63 V F A G C G V V T V N K I Y G S V T F P
301 GTATTTGCTGGCTGTGGAGTCGTTACTGTGAACAAGATCTATGGCTCTGTCACCTTCCCA
83 G I S V T W G L I V M V M I Y T V G H I
361 GGCATAAGTGTGACTTGGGGTCTAATTGTAATGGTTATGATCTATACAGTTGGTCATATC
103 S G A H F N P A V T I T S A I F R R F P
421 TCTGGAGCACATTTCAATCCTGCCGTCACTATCACTTCTGCTATTTTTCGCCGATTTCCT
123 F R E
481 TTCCGGGAGgtgagtctcttgaaccattaaattccaaaggtaaaaagtttgtcatctata
541 agaggtagttcattttggagtaaatatttgattactcattggctaaatgcgtgtttgat
126 V P L Y I V A Q V L G S I L A S G T
601 ggttgtagGTACCCTTATACATAGTTGCTCAGGTGTTGGGATCAATTCTTGCTAGTGGCA
144 L A L V F D V T P N A Y F G T V P V G S
661 CACTAGCTTTAGTGTTTGATGTAACTCCAAATGCATACTTTGGAACAGTACCAGTTGGAT
164 N V Q P L V I E I I I T F L L M F V I S
721 CAAATGTGCAGCCATTAGTTATAGAAATCATCATCACCTTCCTCCTCATGTTTGTTATCT
184 G T T T D H R A
781 CTGGCACTACCACTGATCATAGAGCGgtttgtctttccaaccccatctcatttttttaag
841 ttactagtatgcttctttggcaaaatgagccataatatgcgactatactattgaatgtat
192 V G E L G G I G V G M T
901 tcgtcaaaattttactttttcacagGTGGGAGAGCTAGGAGGCATTGGTGTTGGCATGAC
204 I L L N V F V A G
961 TATACTATTGAATGTATTCGTCGCAGGgtaattttcatctatctttgtacatagtattga
213 P V S G A S M N
1021 atgtattcgtcaaattgatcaatatggtattttgcagGCCGGTTTCAGGAGCATCGATGA
221 P A R S I G P A I V K H V Y T G L W V Y
1081 ACCCAGCAAGGAGCATTGGGCCAGCAATAGTTAAGCACGTATACACAGGATTATGGGTGT
241 I V G P I V G A I A G A F A Y N L L R T
1141 ATATAGTGGGCCCAATTGTTGGAGCCATAGCAGGAGCTTTTGCTTATAACTTGCTCAGAA
261 P E K P L D E L A N K G I L K N S N S R
1201 CTCCAGAAAAGCCACTCGATGAGTTAGCTAACAAGGGAATCCTCAAAAACAGCAATTCAA
281 N *
1261 GGAAT**TAA**atattcatccattcttg

39. *HbNIP5;1*

```
   1 cacaagatgaaattgctaatgaagttgaaggcctcgtttttttgtatataaaccactgaaa
  61 cattaatggttttacgcgcctccttcttcttccttaacgctttactagctttctcttgct
 121 tctgctccagttccagtgtcttccttagattattttcttttcccatcaaaaagccaccca
 181 gatgttttctaatttctactttcgcatagtatattgatttggtttttttggaagctggatc
 241 aatccatcaccgctcttccttaaccataacattataaaaacatatcaattatgtaatttt
 301 catatttccttcacccaacaaaccccaaaaaaatttgaaaaaaaaaaatttctttcttta
 361 gtacttataatctggcttgcttcctttcttttcttccttagctgcacatctataaaagcc
 421 ctagaaaatatgtaagttcgtcttgtcttctccgtagcccaaatcttaaaaaaaaaaaaa
 481 aaaaaaaaaaaagaaagctcgtcattcgttcttaaaaattcttgtatcctaaccgttagc
 541 agtcctggataagaacaagatcatcgtgttggtaaaccgttaaaaaaaaaagagtcaaat
 601 catgtaaaatcgtcccaatcctagctatcatctaaaatttaaaaaatatatatatatata
   1                                                           M
 661 ccttccgttccattgatattcgatattgaagtgtatatatatatatatataaaagcaaAT
   2  P  E  S  E  A  G  T  P  T  V  S  A  P  A  T  P  G  T  P  G
 721 GCCGGAATCCGAGGCAGGGACACCAACAGTGTCAGCGCCGGCGACGCCTGGAACGCCGGG
  22  G  P  L  F  S  S  L  R  V  D  S  L  S  Y  D  R  K  S  M  P
 781 AGGGCCGCTGTTTTCGTCGCTGAGGGTAGACTCATTGTCTTATGATCGAAAGTCAATGCC
  42  R  C  K  C  L  P  V  N  A  P  T  W  G  Q  S  H  T  C  F  T
 841 AAGATGCAAGTGCTTGCCAGTTAATGCTCCAACCTGGGGTCAATCCCACACGTGCTTCAC
  62  D  F  P  S  P  D  V  S  L  T  R  K
 901 TGATTTCCCTTCCCCGGATGTCTCTCTTACCCGCAAGgtacctcctctcttccttccctc
 961 attttctttgaagtacgtcaaaacaattcatatatatattagactttttgttttattttttt
1021 attagtttcaaatctctcagcttttatattactagactaactccactactaattagtaat
  74                                                        L  G
1081 tactaatccgcaattaagaaaaacagtntatatgttgaaatggttggtattgaagCTTGG
  76  A  E  F  V  G  T  F  I  L  I  F  A  A  T  A  G  P  I  V  N
1141 AGCAGAATTCGTGGGAACCTTCATCCTCATATTTGCAGCAACAGCAGGACCAATAGTGAA
  96  Q  K  H  N  G  V  E  S  L  I  G  N  A  A  C  A  G  L  A  V
1201 TCAAAAGCACAATGGAGTGGAGTCATTGATTGGAAATGCAGCATGCGCAGGGTTAGCAGT
 116  M  I  I  I  L  S  T  G  H  I  S  G  A  H  L  N  P  S  L  T
1261 GATGATAATAATTCTATCAACTGGACACATATCAGGGGCTCACTTGAACCCATCCTTGAC
 136  I  A  F  A  A  L  R  H  F  P  W  M  Q  V  P  A  Y  I  A  A
1321 CATTGCCTTTGCAGCTCTCCGTCACTTTCCCTGGATGCAAGTCCCGGCCTACATAGCTGC
 156  Q  V  S  A  S  I  C  A  S  F  A  L  K  G  V  F  H  P  F  M
1381 CCAAGTATCTGCTTCCATATGTGCATCTTTCGCTCTCAAAGGAGTCTTCCATCCATTCAT
 176  S  G  G  V  T  V  P  S  V  S  T  G  Q  A  F  A  L  E  F  L
1441 GTCTGGTGGCGTTACTGTTCCCTCTGTAAGCACTGGCCAGGCTTTCGCACTTGAGTTTCT
 196  I  T  F  N  L  L  F  V  V  T  A  V  A  T  D  T  R  A
1501 CATTACTTTCAATCTCTTGTTCGTTGTAACTGCCGTTGCAACTGATACCAGAGCTgtaag
1561 ttttttttttgtcttattttcttcttaaattaccgttttgtttttttacctctatacgttt
 214                                               V  G  E  L  A  G
1621 ttatgtgtttatgttataaatactctctttctccctatgatcagGTAGGAGAGTTGGCTG
 220   I  A  V  G  A  T  V  M  L  N  I  L  V  A  G
```

1681 GAATAGCTGTTGGAGCAACTGTTATGCTCAACATTCTTGTTGCAGGgtaagtaataattc
1741 ttgccccgctccctccattttttttcccttcttttttttcatgtacccaacacttctttat
1801 tgcaagagtaatcgtgaatagaaaggaaatgtagaattaaaacagaaatcaatcactttc
1861 aactatttaggacttgttttgtttagttagaggaagtaaactatacatatatatacatac
1921 acatatatattacttttagaaaaagtataaaaatatttccttatctaatttacattatca
1981 aattttttcagaaataagttgtgttactctttttagggagggaaataacttgtttatggg
2041 agatttggtaatttatcattttctggttaaattcaattatctttaatttatattatgagt
2101 attattatttttataatttaaaagtaaattttttaaaaaaaaaaaaaaattagtgtatttt
2161 tttcaaaaagtatatttttcaataaatttgagtattttaactcaactaacctcaataatt
2221 ggagatgaccagttggaagaataccattggcttatctaatcaaatagtgtggttgagaga
2281 gctcaagaaaccaatatagtagtctagtcattgcagaatagctagtttggtaatgtatga
2341 aaacatgacaaggcggtcttgccagagtgattatgtctcctgaaaggtgcgaccaatgaa
235 P S
2401 gattccataagaaaggtactaataattgcaagttaccaacttacttcatggcagGCCATC
237 S G G S M N P V R T L G P A V A A G N Y
2461 AAGCGGTGGTTCAATGAATCCGGTGAGGACTCTGGGGCCGGCGGTGGCCGCAGGAAATTA
257 R D L W I Y L V A P T L G A L A G A G T
2521 CAGGGACTTGTGGATATACTTGGTGGCTCCCACACTTGGGGCTCTCGCTGGTGCAGGTAC
277 Y T L V K L R E D E A D P P R P V R S F
2581 TTACACTCTTGTGAAGCTCCGAGAGGACGAAGCGGATCCACCCCGCCCAGTCAGAAGCTT
297 R R *
2641 CCGTCGC**TAG**aagaatgcctctatatactatttaatgtgcaattatagtcattaatggtt
2701 tgcgttaaaaaaaacaaatggtgtgggacatgatatttccatcaaagattgtgaaaataa
2761 aggcgatgcgactataagtcgacgcctacagtgtggtttaataatgctgcagaaggatcg
2821 gtgcttatcatctctcaagtcaatctatgagagatgattgtttggctgtgtcttcttttg
2881 caatgctagctggctatctagctttctttgttgcttcttgtatgattattctactgactt
2941 tcgatataccgcgtggtctgtgcatatttggcttactctatttttaatttattattatta
3001 ttgtattttatttcattattatttaatacaagaaaaataaaaattagagatgc

40. *HbNIP6;1*

1 tctctctctctctctctctctctctctctctttcagagtatcataaaatacccttgat
1 M D N N N E E V P
61 tttgttttcttttgggtggtattagagtgatata**ATG**GACAATAATAATGAAGAGGTTCC
10 S A P S T P A T P G T P G A P L F G G F
121 ATCAGCTCCTTCAACACCAGCAACGCCAGGGACTCCAGGTGCTCCTCTCTTTGGTGGGTT
30 R A E R S G T N R K S L L K G C K C F S
181 TAGAGCGGAGAGATCAGGAACTAATAGAAAATCACTTCTCAAGGGCTGCAAATGCTTCAG
50 V E E W A L E E G R L P P V S C S I P P
241 TGTTGAAGAATGGGCTCTGGAGGAAGGCAGATTGCCTCCTGTCTCTTGCTCAATCCCTCC
70 P P V S L A R K
301 TCCTCCTGTCTCACTTGCAAGAAAGgttattatttatattatgtattttttttaatgtttt
78 V G
361 cttaattatattaattctaattacgatagatcatatttaattaattaattaccagGTGGG
80 A E F I G T L I L I F A G T A T A I V N
421 AGCTGAGTTCATAGGCACTCTTATACTGATATTTGCTGGAACAGCCACGGCCATTGTGAA

100 Q K T Q G T E T L I G L A A S T G L A V
481 CCAAAAAACACAAGGCACAGAAACACTAATAGGCCTCGCTGCTTCTACTGGTTTAGCTGT
120 M I V I L S T G H I S G A H L N P S V T
541 AATGATAGTAATATTATCAACAGGCCACATCTCTGGAGCACATCTCAACCCATCTGTCAC
140 I A F A A L K H F P W K H
601 TATTGCTTTTGCTGCTCTCAAGCACTTTCCATGGAAACATgtaagcacgtaaacaactta
661 cattcattaattgaagtacccttaatttaatatcatttgtattataattaagtaataata
153 V P V Y I G A Q V M A S V S
721 atgagttaactggacgcagGTGCCAGTGTATATTGGAGCACAGGTCATGGCCTCAGTGAG
167 A A F A L K G I F H P I M G G G V T V P
781 TGCAGCATTTGCTTTGAAAGGGATATTTCACCCAATAATGGGTGGAGGAGTAACAGTTCC
187 S G G Y G Q A F A L E F I I S F N L M F
841 TTCAGGAGGATACGGTCAAGCTTTTGCTTTGGAATTCATTATTAGCTTTAATCTCATGTT
207 V V T A V A T D T R A
901 TGTTGTCACTGCCGTGGCCACCGACACTAGAGCTgtaagcaccctcttaatttcttcatt
961 tcaatagtttacagctttcttctcttctcttttcttttcttttcttttctttttttcatta
1021 ttatataacattaatctaaagctagagaaaagttgaaaagaaattagaaaattctactct
1081 tatcacctttttctgattgaacccagttgttttaataagttaattacttaattattgtttc
1141 aagatggaattaaatttcaataatactaaattaagtagaaatttttcttcaattttgtca
1201 ttttattttctttttccttccctttttcaccacttgccagaagcagacaaaccttctgga
1261 ggaattttccagcagacaagagttttttttttttttaaatatttattttgcagagcccaa
1321 gagacaagagtcaatgaaaatggttggccaacctgcaacttgactttccccacctccctc
1381 ctcatcccccccttttttaggaataaatatatcaatcaatagatttcatgattcattttt
1441 ctaggatccagatcaagttataagttgattgctcactctttttaataaaataaagtgatg
1501 ggtaccgaccaaattaattgctcactcttttttaaaatacctgtaaacaacagtgaaatta
1561 taccatgtccttaaccacatcattagaaattaatagaattgtaccctaaattaataaaac
1621 tgccaaatataaaattgtcaaacggtgttaaaactgaggtagcaatcttgttaacatcta
1681 aaggtgttattaacgatagggtataattatattgttgttaataatttagagtgtaattat
1741 taaagaaaaaattagggtacaattttaaaaataagtaaaattctgtttttcaatattaaa
1801 gaaaaaaaaatggcatcaataaattgtttgaaaggaggggttttgagggtaaagttaagt
1861 aaagaaagatttaggagagttttagattatttgggagaagtttacacccccccctccaaa
1921 atcctttcgtttttcaacccaccaatttgatgggttgattactcatttcttatttttgtg
1981 tttttaattgttttataaaagacaatatttataatattcatatttaatcttactttttaa
2041 ttcttttcttttaaataaaataatattattaaatatttatttatcttactttactttatc
2101 tttttctctctcgaatataaaataaaatattatcttaccttaacatattttactttaacg
2161 taccttattttattaaagaaagccttagtgttttgctcaagtctctctcatggtaagagg
2221 aaaacttcaaacaatgatgaattattacacatatcccatccaataattaatagtcacaag
2281 aggaacagggaaaggagcaggaacaggagcagagcccctccttgtctcttgttctttcaa
2341 tttttttcccccaccttattctcctccattccaagtgcttttctccattccacacattc
2401 accatccagaacattttgtatttcatttttcaattgtagattctaggtgggcacatgcct
2461 atgtgagccctcttgttttgtttggtgaagtatctgagtcatcttggttagacttttgga
2521 atttctcccactttacggttcctttttttttttaataaacaaaaagcaacctggcttatct
2581 tttcaatcccccaactccctccttactaataatgatagaaaaaggtataataggctgggt
2641 cgattcaagtatggaactaacttaaaatcgctagtttctatttataaatccaaaattaaa

2701 tttttatttttatttaagatcaaataaattctcatctaataaaatattattattttt
2761 taattttaaagattaactgctgtaaaaaaatgagtgggctatgcatgtacatgttcaaac
2821 aataaaaaaaaactatgcatgtatactaataaataataattaatggcataaaaattgtca
218 V G E L A G I A
2881 aacttggcagctgtggctaaaattatgaacacatgcagGTGGGAGAGTTGGCGGGAATCG
226 V G A T V M L N I L I A G
2941 CGGTGGGGGCCACCGTCATGCTCAACATACTCATCGCCGGgtaggtcgtcgcccctcctg
3001 tcttccttttatatacatatctttcttgcaataattaaggattggtttatcctatactta
3061 agacacaaatatatgattttcatccattttatattgaatccaagcaatggtttctcatta
3121 atgaaataaaattttgcaaacgataggtattatgaaactgttattcgcacgtcaatttca
3181 tagtataatttttctggagattaatgattaatgcttatacatgcctcttgaacttgagga
239 Q S T G A S M N P V R T L
3241 tgtgacaccctctggtgcagCCAATCTACAGGTGCATCCATGAATCCAGTGAGAACTTTA
252 G P A I A A N N Y K G I W I Y L T A P I
3301 GGGCCTGCTATAGCTGCAAACAACTACAAGGGCATATGGATCTACCTCACTGCACCCATT
272 L G A L C G A G T Y S A V K L P E E D A
3361 CTTGGGGCACTGTGTGGAGCAGGAACCTACTCTGCTGTCAAATTGCCGGAGGAAGATGCT
292 D T R E K P S E A R S F R R *
3421 GACACCCGTGAAAAGCCTTCAGAAGCAAGGAGCTTCAGAAGG**TGA**ttatcaacaaatctg
3481 tattagttatccagaaactgtcagataaacaaatgcagatgattctaaatcaggagaaca
3541 aactcaaaagtctcaaactactgcttctgaagttccttctagtaggtacatacacatgca
3601 agagagataaaacatgaatagaatgactctgtatcacatatgaagagtactttactttg
3661 tgtgcagtatatattggaattctgtaacatggtggtctaagaaaagcaatttcagtgctc
3721 caatacacttgtcatttacttcttccgctgttcctgtctttgtctttccaccttctagtt
3781 tctactggtcccatatataatttttctcttagcattatacagcttaacccactaaaaat
3841 agttgacatgattcacggcatatatgctagtgccccaagcaactacgtgttttagtgata
3901 gacacactccaaaggttaataattatatgcctcttaaatttatgttacaacaactaccac
3961 tttcccattactgtcttatcaaatctaatttgataacatagtaaat

41. *HbNIP7;1*

1 M K M K Q L L E D Q L P Y P D I S N N S
1 **ATG**AAAATGAAGCAGCTACTGGAAGATCAGCTACCATATCCTGACATTTCTAATAATTCA
21 S N S G L S R D C P E M G S N A M S I D
61 TCCAACAGCGGATTATCTAGAGATTGTCCAGAGATGGGCTCAAATGCAATGTCAATAGAT
41 G D V F A K Y S V S R C L P E G M D L N
121 GGAGATGTATTTGCTAAATACTCTGTTTCACGCTGTTTACCTGAGGGAATGGATCTAAAT
61 P A R M
181 CCTGCACGCATGgtaagcaaaatgagaccagctagtgatatttcattattatggcctgtg
241 atgagttttgtattgtaatctcaaacgttacaagttacacttacaccaccaaaaatgtgc
65 V L A E M M G T F V L M F C V C G I I G
301 agGTGTTGGCAGAAATGATGGGGACCTTTGTATTAATGTTTTGTGTCTGTGGGATCATAG
85 T T Q I T R G Q V A L L E Y A S T A G L
361 GAACCACACAGATAACTCGAGGCCAAGTGGCTCTTTTGGAATATGCATCCACAGCAGGAT
105 S V I V L V F A L G P I S G A H V N P A
421 TATCAGTCATCGTCTTGGTTTTTGCCTTGGGCCCTATTTCTGGTGCACATGTCAATCCTG

125 V T I A F A A F G H F P W S R
481 CTGTCACAATTGCATTTGCAGCATTTGGTCATTTTCCATGGTCCAGGgtaagtttaccaa
541 attaaaaccagaactccattcatgaaaaccacacttttaggttatagttctgatgcagtt
140 V P F Y V L A Q I V G S I
601 gctttgttaattggttttgtagGTTCCCTTTTATGTACTGGCTCAAATAGTTGGATCAAT
153 L A T Y V G K C V Y D I K P E L M V T Q
661 ATTGGCAACATACGTTGGAAAGTGTGTCTATGATATAAAACCAGAACTTATGGTCACCCA
173 P L Q D C N S A F W V E F I A T F I I M
721 ACCACTTCAAGACTGCAATTCAGCCTTCTGGGTTGAGTTCATAGCTACTTTCATCATCAT
193 F L I A S L T Y Q T S
781 GTTCCTTATTGCTTCATTGACATACCAAACATCAgtaagaacaaaattctctcgtgatta
841 ataagttctatatatgttcctagagcaccaatttagtgatgtttctattccatgacgaat
901 caatgtaaatccatttatgctcgcacttgtatccatcagtattcatataacttaaattta
204 V G H L S G F I V G L S I G L A
961 caaatcttttgcagGTAGGGCATTTGTCTGGTTTTATTGTTGGGCTTTCCATTGGACTTG
220 V L I T G
1021 CAGTCCTTATTACTGGgtatgagtcctttgcatgtttttaatttggatcaataaatgaca
225 P L
1081 ctcagtccaaaccaatcttaattatatgattggcttttatatatcaagtgtagGCCTCTT
227 S G G S L N P A R S L G P A I I S W N F
1141 TCAGGAGGATCATTGAATCCTGCAAGGTCATTAGGGCCCGCAATCATTTCCTGGAATTTT
247 K D I W V Y I T A P V I G S L A G A L M
1201 AAGGACATATGGGTATATATTACTGCACCAGTCATCGGATCTTTAGCCGGAGCTCTCATG
267 F H A L R I Q S R P C T S T D S S T N A
1261 TTTCATGCTCTGCGCATTCAAAGCCGGCCATGCACTTCCACTGATTCCTCAACTAATGCT
287 G L L G H S I A I R R S *
1321 GGTCTACTTGGTCACTCCATAGCCATTAGAAGGAGC**TAG**

42. *HbXIP1;1*

1 M D L V A T Q G D N N H Q P F S K S V E
1 **ATG**GATTTGGTAGCCACACAAGGTGATAATAATCACCAACCATTCTCAAAATCAGTTGAA
21 N C D A I N D F K G T K S P K T S F L V
61 AATTGCGACGCAATCAATGATTTCAAGGGAACAAAATCCCCAAAGACAAGTTTTCTTGTT
41 F I G A H E F F S R E
121 TTTATTGGTGCCCATGAATTCTTTTCACGAGAGgtaatgctacgttatgcatatataaaa
181 tttcaatgttttagagaacttttaattattagggttcttcatgtttgtgtgttctttaat
52 M W R A A L V E L V A T A C L
241 ttttttttttttccagATGTGGAGGGCAGCACTCGTAGAGCTAGTAGCAACAGCTTGCCT
67 L F T L T I S I I S C L E S H V P E P K
301 TTTGTTCACACTAACCATTTCCATAATCTCATGCTTGGAATCACATGTGCCTGAACCCAA
87 L L V P F A V F I I A F F F L L T T V P
361 GCTTCTAGTCCCATTCGCCGTCTTCATTATCGCTTTCTTCTTTCTCTTAACAACTGTTCC
107 L S G G H M S P V F T F I A A L K G V I
421 TTTATCTGGGGGTCATATGAGCCCAGTTTTCACCTTCATTGCAGCTCTCAAAGGCGTTAT
127 T L V R A L L Y V L A Q C I G S I M A Y

481 AACTCTTGTTCGTGCTTTACTCTACGTCTTGGCACAATGTATAGGTTCAATAATGGCTTA
147 M V I K N V M N N S A V E K Y S L G G C
541 TATGGTAATCAAGAATGTGATGAACAATAGTGCAGTAGAGAAGTATTCATTGGGTGGCTG
167 M I D G N E G G I A S G T A L V L E F S
601 CATGATTGATGGAAATGAGGGAGGAATAGCTTCAGGAACTGCACTGGTGCTGGAATTTTC
187 C T F V V L F V G V T V A F D K R R F K
661 TTGCACTTTTGTGGTGTTGTTTGTTGGTGTAACAGTGGCATTTGACAAGAGAAGATTCAA
207 E L G L V M V C V I L A A T M G L A I F
721 AGAGCTAGGCTTAGTGATGGTATGCGTGATATTGGCAGCAACCATGGGGCTTGCAATTTT
227 V S I T V T G R G G Y A G V G L N P A R
781 TGTGTCAATTACCGTAACTGGGAGGGGTGGGTATGCTGGTGTGGGGTTGAATCCTGCAAG
247 C L G P A L L H G G P L W H G H W V F W
841 ATGCTTAGGTCCAGCATTATTGCATGGAGGTCCATTGTGGCATGGGCATTGGGTTTTTG
267 V G P F L A C I I Y Y C Y T L T F G S V
901 GGTTGGGCCTTTCCTGGCTTGCATTATTTATTATTGTTACACTTTGACCTTCGGAAGTGT
287 D E D *
961 AGATGAAGAT**TAG**aaaacgcagtatatttttttgttgttggttgaga

43. *HbXIP1;2*

1 M D S H N T K G S Q Y P M M T F L Y R I
1 **ATG**GATTCTCACAATACTAAAGGCAGCCAATATCCAATGATGACATTTCTTTATCGCATT
21 G A Y E F F S P E
61 GGTGCCTATGAATTCTTTTCCCCAGAGgtaaatatgcttcatttttatatatttcttta
121 ttgaactctagttgagatttaaacttgtaaacttagcataaatgcgattccattacaact
30 L C R A V V T E
181 gatgtttggtgatctcttgtttaattttgttctttcagTTGTGCAGGGCAGTAGTAACCG
38 M A A T T C L L F M L T T T I I A R L E
241 AGATGGCAGCAACTACTTGTCTTCTGTTCATGCTTACCACTACCATTATTGCACGCCTGG
58 S H E T E P K L L I P I A V I V I A F L
301 AATCACACGAGACTGAACCTAAACTTCTAATCCCAATTGCTGTCATCGTTATTGCCTTTC
78 L L V V T V P L S G G H M S P I F T F I
361 TTTTGCTTGTGGTGACAGTTCCTTTATCTGGGGGACATATGAGTCCCATTTTCACATTTA
98 S A L R G L I T L V R A L F N V L A Q C
421 TCTCCGCCCTAAGGGGTCTCATAACTCTCGTTCGAGCTCTCTTCAATGTCCTGGCACAAT
118 V G S I M A Y L V I K S V M N E N T A E
481 GTGTTGGCTCAATAATGGCTTACCTTGTGATCAAGAGCGTAATGAACGAAAATACAGCAG
138 K Y S L G G C M V N G N R S G V N A G T
541 AAAAGTATTCCTTGGGAGGCTGCATGGTGAATGGAAATAGGTCAGGAGTAAACGCAGGAA
158 A L I L E F T C S F L V L Y V A I T I A
601 CTGCCTTGATACTAGAGTTTACATGCTCATTTCTGGTGCTGTATGTTGCCATAACTATTG
178 F N K K M C Q E L G F T M V C V I V A G
661 CATTTAACAAGAAAATGTGCCAAGAATTGGGCTTTACAATGGTTTGTGTTATAGTAGCAG
198 V Y A L A V F A S I T V T G Q A G Y R G
721 GGGTTTATGCACTAGCAGTTTTTGCATCCATCACAGTAACTGGGCAGGCTGGCTATAGAG
218 V G L N P A R F L G P A L L L G G S L W

781 GTGTGGGTCTGAATCCTGCAAGGTTCTTAGGCCCAGCACTGTTGCTAGGAGGGTCATTAT
238 D G H W V F W V G P F L A C I V Y Y G F
841 GGGATGGGCATTGGGTTTTCTGGGTGGGACCATTTCTTGCCTGCATTGTCTATTATGGTT
258 T L T L P K Q G L V R A E K E H H I T Q
901 TTACTTTGACGTTGCCAAAGCAGGGCTTGGTGAGGGCAGAGAAAGAGCATCACATCACAC
278 L V L G S C Y G A G F P S H V E E K V *
961 AGCTGGTTCTGGGTTCTTGTTATGGAGCTGGCTTTCCTTCACATGTTGAGGAGAAAGTT**T**
1021 **AG**

44. *HbXIP1;3*

1 M D L D L V L S H D A G N Q A F P N Q L
1 **ATG**GATTTAGATTTGGTTCTCTCCCATGATGCTGGGAATCAAGCATTCCCAAATCAACTT
21 D G Q N K N I N D S L E S P K K T F L S
61 GACGGCCAGAACAAAAATATTAATGACAGCTTGGAATCTCCAAAGAAAACATTTCTTTCT
41 C I G V H E L L S P E
121 TGCATTGGCGTCCATGAACTCCTTTCCCCAGAGgtaattaaattaattgatagattaatc
181 aatcattagtttcttcataataaacatagatttacattaaggtttagttgtacgcttgct
52 T W K A A I T
241 tgcacgcatatataactctccttgcttttattttcttatagACGTGGAAAGCAGCAATTA
59 E L V S T A C Q L F T L I T M V T A C L
301 CAGAGCTAGTATCAACAGCTTGTCAGTTGTTCACACTAATCACAATGGTAACAGCATGCT
79 E S H V A E A K L L V P V V V F S T I F
361 TGGAATCACATGTAGCAGAGGCGAAACTCCTAGTTCCAGTGGTAGTCTTCTCTACAATCT
99 L L L V V T I P V S G V H M N P T F T F
421 TCCTTTTGCTTGTGGTGACAATTCCTGTATCTGGGGTACATATGAACCCAACTTTCACAT
119 I F A L K G V I T F V R A L V Y I L A Q
481 TTATCTTTGCCTTGAAGGGTGTAATAACTTTTGTTCGAGCTTTGGTCTACATCTTGGCTC
139 C L G S T M A Y L I V K R A M N P K I A
541 AATGTTTAGGGTCAACAATGGCATATCTTATAGTGAAGAGAGCAATGAACCCTAAAATAG
159 E K Y S L G G C S M G G N G E G I S A G
601 CAGAAAAGTATTCCTTAGGTGGCTGCAGTATGGGTGGAAATGGAGAAGGAATAAGTGCAG
179 T A L A I E F A C T F L V L Y F S V T V
661 GAACTGCATTGGCAATAGAATTTGCATGCACATTTCTGGTGCTGTATTTTTCTGTTACTG
199 A F D K K R C K Q L G L T M F C V M V S
721 TTGCATTTGACAAGAAAAGGTGCAAGCAACTAGGCTTGACAATGTTTTGTGTTATGGTGT
219 G I L A V A Y F I S L T I T G Q V G Y G
781 CAGGGATACTTGCGGTAGCATATTTTATCTCACTCACAATAACTGGGCAGGTTGGCTACG
239 G P R L N P A R C I G P A V L V G G S L
841 GGGGTCCACGGCTGAACCCAGCAAGGTGCATAGGCCCAGCAGTATTGGTTGGTGGATCAT
259 W E S L W V F W V G P F C A S I V L *
901 TGTGGGAGAGCCTTTGGGTTTTCTGGGTAGGGCCCTTTTGTGCCTCTATTGTTTTA**TGA**

45. *HbXIP1;4*

1 M D L D L V L S H D A G N Q A F P N Q L
1 **ATG**GATTTAGATTTGGTTCTCTCCCATGATGCTGGGAATCAAGCATTTCCAAATCAACTT

21 D G Q D K N I K G S L E S P K K T F L S
61 GACGGCCAGGACAAAAATATTAAAGGCAGCTTGGAATCTCCAAAGAAAACATTTCTTTCT
41 C I V V H E L L S P E
121 TGCATTGTTGTCCACGAACTCCTTTCACCAGAGgtaattaaattatttgatagattaatc
181 aatcattagtttcttcataataaacatagatttacattaaggtttagttgtacgcatgct
52 K W K A A I T
241 tgcacgcatatataactctccttgcttttatttcttatagAAGTGGAAAGCAGCAATTA
59 Q L V S T A C L L F T L I T M V T A C L
301 CACAGCTAGTATCAACAGCTTGTCTGTTGTTCACACTAATCACAATGGTAACAGCATGCT
79 E S H V A E P K L L V P V V V F S T I F
361 TGGAATCACATGTAGCAGAGCCGAAACTCCTAGTTCCAGTGGTAGTCTTCTCTACAATCT
99 L L L V L T I P V S G G H M N P T F T F
421 TCCTCTTGCTTGTGTTGACAATTCCTGTATCTGGGGGTCATATGAACCCAACTTTCACAT
119 I F A L K G A I T F V R A L V Y I L A Q
481 TTATCTTTGCCTTGAAGGGTGCAATAACTTTTGTTCGAGCTTTGGTCTACATCTTGGCTC
139 C L G S T M A N L I V K R A M N P K I A
541 AATGTTTAGGGTCAACAATGGCAAATCTTATAGTGAAGAGAGCAATGAACCCTAAAATAG
159 E K Y S L G S C S V G G N G E G I S A G
601 CAGAAAAGTATTCCTTAGGTAGCTGCAGTGTAGGTGGAAATGGAGAAGGAATAAGTGCAG
179 T A L A I E F A C T F L V L Y F S I S V
661 GAACTGCACTGGCAATAGAATTTGCATGCACATTTCTGGTGCTGTATTTTTCTATTAGTG
199 A F D K E R C K Q L G L T M F C V I V S
721 TGGCATTTGACAAGGAAAGGTGCAAGCAACTAGGCTTGACAATGTTTTGTGTTATAGTGT
219 G I F A V A Y F I S L T I T G Q V G Y G
781 CAGGGATATTTGCGGTAGCATATTTTATCTCACTCACAATAACTGGGCAGGTTGGCTACG
239 G A R L N P A R C I G P A V L V G G S L
841 GGGGTGCACGGCTGAACCCAGCAAGGTGCATAGGCCCAGCAGTATTGGTTGGTGGATCAT
259 W Q S L W V V W V G P F C A S I V L *
901 TGTGGCAGAGCCTTTGGGTTGTCTGGGTAGGGCCCTTTTGTGCCTCTATTGTTTTA**TGA**

46. *HbXIP2;1*

1 M A D N A S R V V E D E E
1 aagatacatccacactaaaaaaag**ATG**GCAGATAATGCTAGTAGGGTTGTTGAAGATGAAG
14 N G Y G G R K V Q P F A S T P R
61 AAAATGGCTATGGAGGAAGAAAAGTCCAACCATTTGCCTCTACACCACGgtaagtttcaa
121 attctttttcttttttttctccttctagtttttgatagttgctcctcagttcttatcatc
30 P D M D K T E G K K H
181 tggggataaacccttttgtttttgcagGCCAGATATGGACAAAACTGAAGGGAAGAAGCA
41 H P T T L S R I L G F E D L S S L H
241 TCATCCCACTACATTAAGCAGAATTTTGGGCTTCGAAGACCTTTCTTCTTTACATgttag
301 tatctttctatatatatttttcaaataaggccatagatactttgatcctttcaatgatcg
361 tattactgttgcaattggcacatttgtgaactgtaattctattactgttactgataata
59 V W R A S L A E A L G T A
421 gcaagaccaatggtgctatcagGTATGGAGGGCATCTTTAGCAGAGGCTCTTGGCACAGC
72 S L V F A M D T I V I S S Y E T E T K T

481 ATCCCTTGTGTTTGCAATGGATACCATAGTCATCTCCTCCTACGAAACCGAAACCAAAAC
92 P N L I M S A L I A I T V T I L L N A T
541 ACCAAACCTTATAATGTCAGCTTTAATCGCTATAACTGTTACAATTCTCCTCAATGCAAC
112 F P I S G G H I N P V I T L S A A F T G
601 ATTTCCCATCTCGGGTGGACATATCAATCCTGTAATCACCTTGTCAGCTGCCTTCACAGG
132 L V S L S R A A I Y I L A Q C L G G I L
661 CCTTGTCTCTCTTTCACGGGCTGCCATATACATCTTAGCACAGTGTCTCGGAGGCATACT
152 G A L A L K A V V N S T I E K T F S L G
721 GGGTGCACTAGCACTAAAAGCTGTGGTAAACAGCACCATAGAGAAAACATTTTCACTTGG
172 G C T L S I V A P G P H G P I V I G L G
781 AGGTTGTACTCTAAGCATTGTTGCACCAGGGCCACATGGGCCTATTGTCATCGGCCTCGG
192 T A Q A L W L E I I C T F V F L F S S I
841 AACAGCCCAGGCTCTTTGGCTGGAGATAATTTGTACATTTGTTTTCCTTTTTTCTTCAAT
212 W V A F D K R Q A K P L G R V I V C S I
901 CTGGGTGGCATTTGACAAACGCCAAGCCAAACCCCTGGGAAGAGTCATTGTTTGCAGTAT
232 I G L V V G L L V F I S T T V T A T R G
961 AATTGGACTGGTTGTTGGTCTTCTTGTGTTCATTTCTACAACAGTGACTGCAACCAGAGG
252 Y A G V G M N P A R C F G P A I I R G G
1021 TTATGCAGGGGTTGGGATGAACCCAGCAAGGTGTTTCGGTCCAGCAATTATTAGAGGTGG
272 H L W N G H W V F W V G P I I A S I A F
1081 TCACCTGTGGAATGGGCACTGGGTGTTTTGGGTTGGGCCTATCATTGCTAGTATAGCATT
292 A V Y T K I V P S A E V H A *
1141 TGCTGTGTACACCAAGATAGTTCCAAGCGCTGAGGTCCATGCA**TAA**ggcgaaaaaagctg
1201 tcttcgtcaataaaatgaagaggctttaaatgatgtaagatggtatctttaaaatggcct
1261 caggattaggaaggaatgcttgtttatgatgttacagaaataatgcccaattacgcttgc
1321 tgttaggctttcatttctggtcttaaaaagtgctgatattttgcaccagaaagtgtcaac
1381 ttctttataatttaaaataatttaggcttggtagtggaaaataaattgtacatgaagaat
1441 actttttaaaaaatattaattaacagcttacggattaacttaaaaaa

47. *HbXIP3;1*

1 M V L T F Q V W R A S F S E F L G T A V
1 **ATG**GTGCTTACGTTTCAGGTGTGGAGAGCATCATTTTCAGAGTTCTTGGGCACAGCGGTT
21 L V F V I D T V V I S T I E S E T K I P
61 CTCGTTTTCGTAATAGACACCGTGGTCATTTCCACCATTGAAAGTGAGACAAAAATACCA
41 N L I L S C L V A I T V T I I L L A T Y
121 AACCTTATACTATCATGCCTAGTTGCCATCACTGTCACAATTATCCTCCTGGCAACCTAT
61 P I S G G H I N P L V T F S A A L T G L
181 CCCATTTCCGGTGGCCACATTAACCCTCTTGTCACCTTCTCAGCTGCACTCACTGGCCTC
81 I S M T K A F I Y I L A Q C A G G V V G
241 ATTTCCATGACAAAAGCCTTCATATACATCTTGGCTCAATGTGCTGGTGGCGTTGTCGGT
101 A L A L K A V V N S K I E S T F S L G G
301 GCACTAGCACTAAAAGCTGTAGTGAACAGCAAAATTGAGAGTACATTTTCGCTTGGAGGC
121 C T L H I V A P G P D G R P T V I G L E
361 TGCACCCTGCATATTGTTGCACCAGGGCCGGATGGTCGTCCAACGGTGATTGGGTTAGAA
141 T G Q A L W L E I I C G F V F L F A S V

421 ACTGGGCAGGCCCTTTGGCTAGAGATAATTTGTGGGTTTGTGTTTCTCTTTGCATCGGTG
161 W M A F D H R Q A K A L G H V K I F M I
481 TGGATGGCCTTTGATCATCGCCAGGCCAAGGCCTTGGGTCATGTCAAAATTTTCATGATC
181 V G I V L G L L V Y V S T S V T T A K G
541 GTGGGTATAGTGCTGGGTCTTCTTGTGTATGTTTCAACTTCGGTGACAACAGCTAAAGGC
201 Y A G A G L N P A R C L G P A I V R G G
601 TATGCTGGAGCTGGGCTGAACCCAGCTAGGTGTTTGGGTCCAGCTATAGTTCGAGGAGGT
221 R L W D G H W V F W V G P A V S S V A F
661 CGTCTTTGGGATGGGCATTGGGTTTTTTGGGTGGGGCCTGCTGTTTCTTCTGTTGCATTT
241 S L Y T K L I P P Q L S H T I F *
721 TCTTTGTACACAAAACTTATTCCACCTCAGCTTTCTCACACTATTTTT**TAA**

48. *HbSIP1;1*

1 aattcccaaagccggccagtgtcttttggttttgtcttgtacagcgaacctttttgtttc
61 ttttctacctcaggactgcacaaacaaacacccaagagataaaacagtttaatattgcag
1 M G V I K A A I G D A
121 tccaagagaaatagagagatacaccacc**ATG**GGTGTGATCAAAGCAGCCATAGGAGATGC
12 I L T S M W V F S M P L L G I F A S A V
181 AATTTTGACTTCCATGTGGGTATTTAGCATGCCACTTCTGGGAATTTTCGCCTCCGCTGT
32 A A Y I G V E A M S V A G L F I T I N V
241 AGCAGCATATATAGGTGTAGAAGCCATGTCAGTAGCAGGTCTTTTCATTACTATAAATGT
52 A T C F V L T F S L I G A A L G G A S F
301 AGCCACTTGTTTTGTGCTAACTTTCAGCTTGATAGGAGCTGCCTTGGGTGGTGCCAGCTT
72 N P T A T I S F Y A A G I K P D A S L M
361 CAATCCCACCGCAACTATATCCTTTTATGCTGCTGGGATTAAACCTGATGCGTCTCTCAT
92 S M A V R F P A Q A A G G V G G A K A I
421 GTCCATGGCCGTACGATTTCCTGCTCAGGCGGCTGGAGGGGTTGGTGGAGCCAAGGCAAT
112 L Q A M P S K Y K H L L K G P S L K V D
481 CTTACAAGCTATGCCTAGCAAATACAAGCATCTGCTCAAGGGTCCTTCTTTGAAGGTGGA
132 L H T G A V A E G T L T F V F C L A M L
541 TTTGCATACAGGAGCAGTTGCAGAAGGGACGTTGACTTTTGTTTTTTGCCTTGCTATGCT
152 L V M V K G P K N L L L K V W M V A V V
601 TCTTGTTATGGTTAAGGGTCCCAAGAACTTGTTATTGAAGGTTTGGATGGTGGCTGTGGT
172 T V G L V V S G R Q Y T G P S L N P A N
661 TACTGTGGGATTGGTTGTTTCTGGTAGGCAATATACAGGGCCTTCCTTGAACCCTGCCAA
192 A Y G W A Y I N N W H N T W E L F Y V Y
721 TGCCTATGGATGGGCATATATAAACAATTGGCATAACACCTGGGAGTTGTTTTATGTTTA
212 W I C P F V G A T L A A W V F R Y L F K
781 TTGGATCTGCCCTTTTGTTGGAGCAACTTTGGCTGCTTGGGTTTTCCGTTACCTGTTCAA
232 A P I K D K Q A *
841 GGCCCCCATCAAGGATAAGCAAGCT**TGA**atagaagaacagttatatgctttagaggtatt
901 ttatctcctctgtatttgcttgtactcttctctttgatttggaaacaagaaattggatta
961 ttaagcaagtgaaagaaattcatgaatcccaaatgaaaaatgaagctaattatcaatgga
1021 tgcctgcaaagacctgagcttgaaaattgagttttccaccatgacagatttgtggggaat
1081 attgaaggtactaaagatttttttttttttcctttcttaaaaaataaaaaagaaagattc

1141 tagcttagtggatagtcagtggacactagttaataatgcttattattgaaaacttccata
1201 accccctaaggtgtcatccctttgcacaagaattttctaaagtatggttcaaatgtttga
1261 tttattcagaacataatcaaaccgtttggtttttgtcga

49. ***HbSIP1;2***

1 gagaaacagggtccaaatagaagacaaactccacaggaaatttggaaacagaagaagctt
61 atcccttctctgattgggtaaatcacaatcacaaacactacatttccatctttccctgta
121 atttttagagctttaatccaaacaaaacccaaaaggaaaagacgtccactttcccagtt
181 actgccaaaactcgcgcaaaattccaggccaactaaacgccaccattctctcttcactag
241 atacccagctgtagcttcattgacttgaacataccttgcatcgctatcaaacccaaagag
1 M G A I K A A I G D A V L T
301 gaaaactttaggaatttgcg**ATG**GGGGCTATAAAGGCGGCGATTGGTGATGCAGTGCTGA
15 F M W V F C S S M F G L F T S L I A T A
361 CTTTTATGTGGGTCTTCTGCTCTTCAATGTTTGGTTTGTTTACCAGCCTCATAGCCACCG
35 L G V Q H L F W A S M F I T T V L F F I
421 CTCTCGGCGTTCAGCACCTGTTCTGGGCATCTATGTTTATCACTACCGTTCTATTTTTTA
55 F F F L F G L I A E F F G G A S F N P T
481 TCTTTTTTTTCTTGTTTGGTTTGATCGCTGAGTTCTTTGGTGGAGCTAGTTTTAATCCCA
75 G T A S F Y A A G F G G D N L F S M A L
541 CTGGTACTGCTTCTTTCTATGCTGCTGGCTTTGGTGGGGATAATCTCTTTTCCATGGCTC
95 R F P A Q
601 TCAGATTCCCTGCTCAGgtaatatatatgcgtctctgtgtgtgagaatccatttttttat
661 ctgtctgcatgcatgtttagtagatacaggaacaaaagaattgaattacccccggttat
721 tgtttattgcatggatttattgatcgtttcattcgtttgttgccaattcagtggtatttt
781 gttcgttctgtatatttatggatttaggtttttatcatttgtgttatttctgaaagagag
841 aagaaaaatcgactggattactttcttttagtcttttttttttttggttttcagctttag
901 catttttgtggactttttgaattcttcctagaaatatcttgtaattattgtctatgaacc
961 tttattttctagagaaaatgaatctagaaatagaataatacccatatgccaaaaaaggag
1021 taatatagttttgatagaaagaaagtagctttataatgagccatcttccagaaaagagaa
1081 aaatggattccaaaagaataattcatacgtgcaataacctgtatctggaaatctctggaa
1141 aaaagagaagaattttaaagggacaacaaagatttataatgttacattaatgcatttacc
1201 ttacttcttcagaaatagtattcattaacatgcttaagctgttagtgaataatattaagc
100 A A G A V G G A L A I L
1261 gtggaatgtgattggcttcttggtagGCAGCAGGTGCCGTGGGTGGTGCATTGGCGATTT
112 E V M P P Q Y K H M L G G P T L K V D L
1321 TGGAGGTGATGCCACCACAGTATAAGCACATGCTTGGAGGCCCTACTTTGAAAGTTGACT
132 H T G A I A E G L L T F L I S F A V L I
1381 TGCATACAGGAGCCATTGCCGAGGGGTTGTTGACATTTTTAATTAGCTTTGCTGTTCTTA
152 I F L R G P C N S I V Q N W L L A V V T
1441 TAATTTTCCTTAGGGGCCCTTGTAACTCGATAGTGCAAAATTGGTTGCTTGCTGTTGTGA
172 V T L V V T G S K Y T G P S M N P A N
1501 CTGTAACATTGGTTGTTACAGGTTCCAAATACACTGGGCCTTCCATGAATCCTGCTAATg
1561 tgagtagcccagcttctttacattttcagagtaatttattgcttttatttttttgtcttaa
1621 taatgtggagtgcttttgaatctttctaaattattagcgatatctaaatgacgcaatgac
1681 cattgatttttttgcacttatcgcttgtattactaagtagaaactactttaaattgcatgg

1741 ttgtcaaatgattttaattgtgcaactatgcctaaaggtactattttatttcgtgtcgca
1801 tcttattttattttgtgtttagtgttgctcccacttgctccaatcttttcttgatcattt
1861 tttgtcattattctatttgaaatttagccaactcaagttttgaacattttaaatgcatat
1921 tgtagttacttgcttgaagatgtgccatacaatgctttaaaatttatatgaaactatgtg
1981 tttacagaatacaaactggtagtgtgttttgccttgtcttatttcatgtgtttaatgtta
2041 cttctgcctgcttcaatcttcttgcgattgcttttggttttcattctatccaagattttg
2101 gcttacatgagttttacacatttagattttcattatttgtttgaagatgtgtcatacatt
2161 gctttaacatttatatgaaactatatgttaaatagaaaataaatgtggtttttgcattgc
2221 atcttgttttattttgtgcttatggtattggtacttccaattgccttaatcttcttgcaa
2281 tcactttttttgtcattattctatctgaaagtatttttgccatcacttgttcgaagatgtgc
2341 cattcattgctatagtatttatataacactaaaatttaacagaacacaactgtggagtat
2401 taacctgtgctatctcttcaatttgatttccttttgtgctcatacctatcccccatttta
2461 cttgagcattttattttaagattgttttggatatatgattggcactgccttgctgattaa
2521 actgtgttggagacattttagcacaaggttggaacttactcggttagtctattctttttc
2581 catttttatgggcatgtagccaggttgctttcttcatactatgtcaatattttactgact
2641 tgcctattttaaattcattgaaatccaaaaaagataaaaagataaatcaagaaatatatt
2701 gggtataagaaggaaaaaggcaagttatctgggagcttaatttcaagaagaggcaaattt
2761 gtggtatttatgagatctctatcttactggacagaggggtatgactttggtttttatgct
2821 gtatattataatagctatatttttaactaagaattgccactattttttttgtataccttat
2881 tgaatgacaagctcaaattctcccttccttgcacacctaggattataataatatagatac
2941 gaaatgacagaagaataactttatgtgataaagaatgtggtcaaggaaccttttgtgaaa
3001 ttgagatgaacttctatattttttttgctagaactggaagtagtggaagaagagatagaca
3061 aaaagatgatatttaagtgggaagggaggccagatcctgctaatactctagttttctacc
3121 atactttagagtattctgataaacttatgtcctttttccttcttctgccttgtttagtat
3181 aaattaattcagttacactgaaaatttttaatgggtctagggaaatattctgacttatcc
3241 ttccctcttgaattacctaggctaaatcattcattccctcatttaaaggatgaactggat
3301 caataaataaatgatgaggaggaaaggaaaggaatgatcatttaatcttcttttccttac
3361 agtctaggcattctatgtctgtcatatttactcagtttgccaagagtgggaagaaatgta
3421 gagcaattgtggacatttttttgtcctgattaagatctgtttgatatatatgcaaagcaga
3481 tatgatggtttaggacgtttggttctgttgtcataatggtggtgctaatgagtataatgg
3541 tgagccctggtgttgatcttggtggctgcccatgctggtatgtcatgttttttgaaagtt
3601 ttgaggctagtattcttcccctgagatgtttggttgtgtggtcataatggtggtgctaat
3661 gagtataatggtgagacctgctgttgatcttggtggttgcccatgctggtatgtcatgtt
3721 ttttgaaagttttgaggctagtactcttcccctgagatgacaattctggatcatggcaaa
3781 catgtttctgtatgaagataagtgcagaaaagaaatgaaagcacagggatttagtttaca
3841 aagttgcctttgacaaatctttgaagggtaaactggtaactatatgttttcatctccaac
3901 agctttttttggttgatttcaaaggtttttgtgacttgttctttgatggtataatatagg
3961 atctgttgctgccattttgctccagaatttcttctgtggaatagaaaggaattgtgcaca
4021 tggaaactgagttttgcaaattgatcttgcatccatcattttgcaatttgtagaactttc
4081 tacttctctctcatattgacattatctattctaatattaggctaacttatgtttaggccg
4141 attgtgcagctcaggctctgaactctgatgtactaaagaaattatgaggtgtctcgtagt
4201 atggatttcatgtttacactatggttgacaatgttggttgtcctgtttctgtctatccat
4261 catgtggcgatagagtaatgaatcaaaatgcgtcttttcacattatcttgaggcacaatc
4321 cttgttcgggtgcaagttgagtcataggcaataaaagttagattcttctcttgtacaggg

4381 atattattggtctttccaggaaacattgcctacattaggaaaagtaatagcctttacaga

4441 actaattgctgctaagattgttgttagcaggccttttacataatatattctttgcatgat

4501 tcctgttttgggtcttagttataggaatttaaatatgccagtaaatattgcaacgtaaac

4561 atacatgtagatgtatatgtgtgtcagtatgtgtgttcaagttatttgttccttccttta

4621 atttgcatttttttgttccttcagaggatcattacctttggttgcaaattcgcacatttag

4681 acgactactataacaatcatatataagtctaactaaatgtagatgagttattgtttctac

191 A F G W A Y V N K W H D T W E Q F Y V Y

4741 agGCTTTTGGGTGGGCATATGTAAACAAATGGCATGATACATGGGAACAATTCTACGTTT

211 W I C P F I G A I L A A W V F R L V F P

4801 ATTGGATTTGCCCCTTCATAGGAGCAATATTGGCTGCTTGGGTCTTTCGCCTGGTCTTCC

231 P P A P K Q K E A *

4861 CCCCACCAGCACCAAAACAGAAGGAAGCC**TGA**aaatgtacggttagctcattttaattgc

4921 ataatagagaagttaggtgtcaagtgtaatgaaatattttactcttccctttgactctta

4981 atgtttggatcatcttcagttaagttgcttgtactgatggcaattggcaaatctttccca

5041 tctttctcccctctcatcagtgctttctttcatgccattattattttcaatatgacatgc

5101 ttctcaaggaaataggcaactgattgactcacgcttatcatgac

50. *HbSIP1;3*

1 gaaaaaaaagtcaaaacttgtaaataaagagaaagagagtcctaatgaaacatggaaaga

61 acagaagaagagcttcttgtggttaggtaaatcacaaacaaaacccaaactaaaaaatcc

121 catctttaaacgttcactttcccagttcctgccaaaactcgcacaaatatcccaggccaa

181 ctaagcaccaccaagtttgctcttttcatctgatacccagctgattccttctacttaacc

1 M G A I K

241 agactatagctatcaaacccagaaacgaaaagctttaggaagtacca**ATG**GGTGCGATAA

6 S A I G D A V L T F M W V F C S S M F G

301 AGTCGGCTATTGGTGATGCTGTGCTCACGTTCATGTGGGTCTTCTGCTCGTCAATGTTCG

26 F F T S L I A T A L G V H H Q F W A S L

361 GCTTCTTTACCAGCCTCATAGCCACTGCGCTCGGCGTTCATCACCAGTTTTGGGCCTCTC

46 F I T T V I V F V F V F L F G L I A E F

421 TTTTTATTACCACTGTTATTGTTTTTGTATTTGTTTTCTTGTTTGGCCTGATCGCTGAAT

66 L G G A S F N P T G T A S F Y A A G F G

481 TCTTGGGTGGAGCCAGTTTTAATCCCACTGGTACTGCTTCTTTCTATGCTGCTGGGTTTG

86 G D N L F S M A L R F P A Q

541 GTGGGGATAATCTCTTCTCCATGGCCCTGAGATTCCCTGCTCAGgtaacgtatacaagaa

601 tcgatttttatatctgtctgcatgcttttgaattgtccctttaaatgtttattgcatgga

661 tttatcgattagttgttgccaattcagtggtattttgtttattctgtgtatgtatggatt

721 tagattcttatcgtctgtgttaataattcgactggatcttactttcttttagtcccccc

781 tccgtcttttttttttttttattctttttcgttttcagctttagcttttctggggacttt

841 ttgaagtctaattgtttttcctagaaacattttgtaattgttgcttatgtgcctttatat

901 tttctacagaaaatggatctagaataacccgtgtaccacaaaaggagaaatgtagttttg

961 aagtaaagaaaatagctttatgataagccatctttcggaaaagagaaaagcagattccaa

1021 aagaataatttaaatgtgtaatagcccttttctgaaaattcgtgggaaaagaggagaatt

1081 ttaaaggaacaacaaagattttatattgttgcagtcatgcttttaccttagactgcttca

1141 gaaatagtatttcataacatgcttaagctgccagtgaatctattaagaatggaatgttaa

100 A A G A V G G A L A I L E V M P

1201 tgtcttctcggcagGCAGCAGGTGCTGTGGGTGGTGCGTTGGCCATTTTGGAGGTGATGC
116 P Q Y K H M L G G P T L K V D L H T G A
1261 CACCACAGTATAAGCACATGCTTGGAGGCCCTACTTTGAAAGTTGACTTGCATACTGGAG
136 I A E G V L T F L I S F A V L V I I L R
1321 CCATTGCTGAGGGGGTGTTGACATTTTTAATTAGCTTTGCTGTTCTTGTAATTATACTTA
156 G P R N S L V Q N W L L A V V T V T L V
1381 GGGGTCCTCGTAACTCACTGGTGCAGAATTGGTTGCTTGCCGTTGTAACTGTAACATTGG
176 V S G S K Y T G P S M N P A N
1441 TTGTCTCAGGTTCCAAATACACTGGGCCTTCCATGAATCCTGCTAATgtgagtagccctg
1501 ttctcttcacattttctgatttactgctttaatttcctgtcttaataatgtgtattgct
1561 tttgaatcctttctgaattatgcaatgtgatgtaaataatgcatggacaggacaattgcc
1621 ttttgaccttgtgttttctattacgaagtagaaactactgtaaaatgtatgcttgttaaa
1681 tgatttaaattgtgaatatgctgaaaggtacttttttttttggtgtcacatcttgtttta
1741 ttttatgtgtttaatgttactctcattcgctccattctttttgccatcaccttttgtcat
1801 tattctatttcaactttaggctaacttgagttttactcattttaaatgtatattttcact
1861 acttgtttgaagaagtgtcgcacaccactctaacattcacatgaaaattatgtgctaacg
1921 gaagtcaaatgtggttgtgttttgcatctcgttttatttcatgtatttgatgttacaccc
1981 tccagctcgaatcttctcgtaaatgcgtttttttgtttttttattatgtcattatatgttgg
2041 aagatgtgtcatacagtgctttagcatttatatgatacttatctcaacagaaaatacatg
2101 tggttttgtgttgcatcttgttcttcttgcaatcacttttttgtagttattacatccaaaa
2161 ttctaactggcttgagttctgcacatttttaatgtatattgctgttatttgttcgaagat
2221 gcggcatatattgctccaacatttacataattgtgtcttgtttgattcatggaatgaaat
2281 aaagtaggtatggaggataaatttttgacaaccgtccctgaacttatttagttataacatt
2341 acagtctctcaatttaaaaatataacataaaacccatcaattttcaaattttccacaata
2401 aaattcctctaaaccctaattatcggtttttcagttagatgctgacctggacagttttag
2461 catggagtttagtcagtatttctcttttctctctcaagccatgtgtaaattgaatccttc
2521 tcctctttgtagacagaataatttttcacttgtagagagaataatttatactttgcata
2581 agagaggagagagaaatattgactaaacatcatatgggagctgttcacgtcagtttttaa
2641 ctggaaaattaataattgaaagtcagagggattttacagtgcaaaatttgaaagtttatg
2701 gggttttatgttatattttaaagttgaaggattgtaatgttacaaccatataagttcaat
2761 aacagttgttgaaatttatcctaggtatggaataattattcatggtatatatatatatat
2821 atatatatatatatatatatatatatatatatatatatatatataatgattatttgacat
2881 tttaaagtgtcattaaaaaattaaaatgataaaatagttattccatagaatagtattctg
2941 catttaaatttgaaccaaagctaggaataactattctatacctattgtattctaggaacc
3001 aagctaggccaaaatgttaagagaagacaactgtggcttattggcctgtcatatgggatt
3061 cgttatattagtttgaaatgtactttatctcttcagtttgatttctttttgcgcttatcc
3121 ctatccctacatgattggcattaccttagtgatgaaattcgttatattagtttgaaatgt
3181 actgtatctcttgagtttgattaccgtttgtgcttatccctatccccctttttaacattca
3241 tgtttaagcattttctttaagattgttttggatatatgattggcattaccttattgatga
3301 aattatgtgttggagacattttagcacaaggttggaacttggaacttgcttgctttttttt
3361 ccattttttcggggatgtagccaggttgctttcttcgtaccacgtcaatgtttttctgga
3421 cttgcctatttttattcattgaaaacaaaaaagataaaaggtaaatcaagaaacatgttg
3481 tacataagatggggaaaagaaaggcaacttatctcagaacatacttccaagaagaggcaa
3541 attgatgatatttacgagatctctgactattctgtctatgaacagaatatgggaatgact

3601 ttggcttatctatatattataatagctatgtttttagatgagaaattgccactatatttt
3661 gtaaaccttattgaatggaaactcttcctacttgcccacctttaattataatgatatagt
3721 tctgaaaaatgacattatggataactttatttgataaaaaatgtggtcaaggaaccttttt
3781 gtgaaattgagatggacatctgtgtatggttgctagaattagtggaagaagacccagaca
3841 aaaagttgaaatttgagtgagtaaggaggttggatcccactaatgctttgtttgtctttt
3901 ttccacctgcctttttttggaataaatttatctgcttaaaagtaaaaaatttcagtggtct
3961 gaagtaatattctgacttctccttccattttgaattacctaaaccactcatttacctcat
4021 ttaaaggatgaattagattacctcacttgttctttttttgttgactggatgggcacaaaa
4081 ggttttgcccttctatcatttggatgaataaataagtgatagggagggaaaggaatggaa
4141 agattatttaatcttcttttcctcatatgttactggataagaagtgtaaattttatacat
4201 tgtagtgtgtcacataattactcaattcgccaagaatgggaaggaatggaaagcaactga
4261 agtttttctcctatttaaaatctcctctttgatttgtatgcaacgtagatgtgttggtt
4321 taggatgtttagttgtatggtcgtaatggtggtgctgaccaatataatggtaagacatgg
4381 tgttgatctgggtctgtgccctgtggttaccaatgttgtttttttttaaaaagttttggg
4441 gctagtacttttcctctgagatatcaggtgtttaatgcaaccatgattttgttatgactg
4501 gtagccaaagtagtttgataacaattcaggatcatgacaagcatatttcaatgtgaagat
4561 aagtgcagaaaagaaatgaaagcgcgggtgtttagtttgctaagtttcctttgacaggag
4621 tctttgaagagtaaactgaaaactatctgttatcataattcataaacaatatttttttgtt
4681 ggttagtatagtttttgttggttatcttcaagcgtttttggaacctgccctttgatggta
4741 aaaatgtaggatctgttacctgtatttttgccccaggatttgacttttgtttgattggat
4801 taagctcatcagttgaagggctgcttctgtggaatagaaaagatttgtttcacaggaagc
4861 tgagttttgcatatagatcttgcatctatcatcattattctaatattaggctaattgatg
4921 tattgtgcagctctagctctgaattctgtatgtactagagaaattatgagacatctcatg
4981 atgtggatttcatgtttacagtctggttgagaatgttgatctcttgcacctactgacctc
5041 tgttgtcctgtttcttttttttcatcatgtagctgtataatgtaaccaatcaaattgcat
5101 cttttcacattatcttgaggcatatgtggtattggggtcagggtaagggagctttgacac
5161 agtccttgtttttgtaggagttagtcatcaacaataaaaattggattgctctcttgtact
5221 ggtatattactggactttccagaaaacattgcacactttaggataggtcatagtagtctt
5281 tgcacaactaaatggtgctgagattgttgttagcaggctctcagaatttggcgttgctac
5341 ataatctgatctttgcgcgtaactcctgtcgcaggatttagttattagtaaattatgata
5401 tgcctgtaaacattgcatcataaacactaaaaagtcatttgcatgtgtctctggttttat
5461 tgttcttgttcctccaaatgatcattaccttcgattgcaaatttagcacttctagactac
191 A F
5521 tactataataatcacatataagactaactaaatgtgcaagttattgtttctacagGCATT
193 G W A Y I N K R H D T W E Q F Y V Y W I
5581 TGGGTGGGCATATATAAACAAAAGGCACGATACATGGGAACAATTCTATGTTTATTGGAT
213 C P F V G A I L A A W V F R L V F P P P
5641 TTGCCCCTTCGTAGGAGCAATATTGGCTGCTTGGGTCTTCCGCCTCGTCTTCCCTCCGCC
233 A P K Q K K A *
5701 AGCACCAAAACAGAAGAAAGCC**TGA**aagagcacaattagctcatggtaattgcataatag
5761 agaagctgggtgtcaagtgtaataaagtcctttactctttcctcttgacccttaagacaa
5821 tcttcagttaatttgcttgtattgattgcaatttgcatatcttttccatctggcccttgc
5881 tttccttcgtaccattttta

51. ***HbSIP2;1***

1 ttcgtcactgcgtatgtactattcagtgcactaataaactaaaccaatcgtaaacccaag
61 tctttaaaaaataaaaaaaccatgggaaagggctagcaatattttattctgtggtgaata
121 aaatacaagtttattaaatagaaaatgaattaacgactctacattttgacccggctgcta
181 tcatagaacacgaaggtggtgctgggaaaaagcggagagcctcaccaacgagaaatagga
241 aatagagcaggaagccgtgagttcaccatcaccacctctcactttccttaatataaacaa
301 cgtccgtttacatgcattacccagtttaagttacagagaaaacaaaagttcttggtggaa
1 M S S A V T L R L I I S D F V I S F M W
361 **ATG**AGTTCTGCAGTTACTCTTCGTTTGATCATATCAGACTTTGTCATTTCTTTCATGTGG
21 V W S G A L I K M F V N R V L G V G H H
421 GTATGGTCAGGAGCTTTGATAAAGATGTTTGTCAATCGTGTTTTGGGAGTGGGGCATCAC
41 E P R G E A I K A T L S I I N M I F F A
481 GAACCCAGAGGCGAGGCCATCAAAGCTACTTTGTCTATCATTAATATGATCTTCTTTGCT
61 F L G K I T K G G A Y N P L T V F S P A
541 TTCTTGGGCAAGATCACCAAAGGAGGGGCTTACAATCCTCTTACTGTTTTCTCTCCTGCA
81 I S G D F S R F L L T V G A R I P A Q
601 ATCTCTGGGGATTTTAGTCGTTTTCTCCTCACTGTTGGGGCTAGAATCCCTGCTCAGgtt
661 tttgttcattttgatttcgtttttgcttgatcattattttattctgatttgctttctgtg
721 tttgtttcggtagctgaataactctgattgagtaatttggatttgagtttcaaattattt
781 ttatgttttggggttgcgtgaaattgcaagttctaatgtgcaaaatttaaccttttcttt
841 ttctgaaaaaaaaaaaatcactatgttattgactttaatgtcagcatttttggagtccag
901 atgggtttggctttgaatcatctctaatccagtcatttttacctattgtttctaacttta
961 tctcacaataattggatgtgggtgcagctttccattgttatgcaatttatttttttttt
1021 gtagatcttaaatttttggaaagagatgaagaattcattattattagcaaatcttataag
1081 tgcatttttttttttgctttatactactgtccttacccaccctttcaataagagaaaaaaa
1141 aaaaaaaaaaaaagtaatttgatgcaattaatttgattgtccttaataaagcagaagggt
1201 catgtagccaacctcaactagttagtagtgtttgatgaaattgcaaattgagtgcagaaa
1261 atttgagatttgcatattttagtattcccaaatttaaatttgagattcaactgaattata
1321 agtttttaactaaagactgctttgttaccaaaatacagattgtgaatttgattatattgt
1381 taaaatatcacaactaaattttacaattatatgaatttagcaagctaaagataggggtga
1441 tgatggtggtattatggatttcaaatagtggcagttatgttgtctaacagctgtaaatgc
1501 tgaagtaaaggtaatggctattatcaatacaaaatggttctttctttgggtgtttttgta
1561 ggttttgatgcacaaaatccctacttctctaggtgccatttcatgtcctttttagccttc
1621 aacctaaatcgtacaaaactcttgctacaagatttttttctttaaaaagtttcactttg
1681 tagcaaagtaaaagcaaagtcgaagggagtatgtttaggcctttctgcattttcttttag
1741 atggacgagtcaattttgactttgttgttgtgcttttgagcatttgttttggttttccta
1801 taattatggctccttttgctgatgatgatgttagatctccttgttgtttgttgctagtta
1861 ctgatgattgacgttaatgaatataaatcgtggaaccatgtttttatttgatgtatatat
1921 tatacttgtttgttagttgcattctgggtattattcaagtccttgaatatctatttatat
1981 aagaattttgcaatttgtatcaaaaacaaaattgtattgacaagctagtactgttagctt
2041 atgagtgcgatggccctgattccttcacaaccaactctgattttaatatgcgggtggcta
2101 gtaatatatgtggtgatataatgacatggtagtggcattgatgatgataatgattattgt
2161 taatgtattgatggcaatcatagtgttcctggttatggtggtggtgagggtagatgacaa
2221 caatagtggtaatgacggtgctagcttagaacattatgtaaaaatttctttttgatatt
2281 cttccctttccctcccccatcttctctccatatctctaatttcttgttattctttctctc

2341 tgatctctgaaggttccacatcaatttttttcccctcagagcaacccttgatattgacat
2401 tgccatcatactcgttactacatcttttgcaaatgaaataccaaattaacctcttaaccc
2461 ctaattttgaactaccatttggacattgtcttttgttgttcttaaatgaatttgcaaag
2521 aatatatcggttgctattttgataccctgcctaccacaaattcaaattgctatgactgaa
2581 tttaagtgttgtgtgaatagaacatctcaactaccaagacgagaaactgataatgctcga
2641 attgttctgggatgtctaccatttctgaaacctcatcaacaattatgcctttctcaaaaa
2701 ccccaccctcaaaattctgtcatctttgatcattgatattgtcatcattgagttggtatt
2761 tgctatatcttatttattgattattgaggctgctctctgtctggtttgcattcctgtctt
2821 cactagttatttttgccaccaaggctgtttactggttgatggggccgattggtgcagata
2881 tttatgttggtgtttaactgactggtttctgaggtgtattgttagacggcaagcagttta
2941 atttgagtttgatattaagttgggttggttgctgacagattttggtttactatgcattct
3001 ataggcatggcaaagcttgtggataaaaatactatagtaagcactccaattatggatata
3061 gggattgtgagttgaccagaattttgtaacacaatattttcaatccagttgaagtctgtt
3121 gttatagcttagtggaggatagtggaggagatattcattatgcgaccttttattaaataa
3181 tatgttgtaaacaatgccattttggttgtgacattactctcaggattcttaatttatgat
3241 gatcactaacatctggacattcagtatttgttggtgaatatacttctcattatgaaactc
3301 tctcttaagggaaagagactattctaaagaaaactgaccgcatatttctgaatattggac
3361 aaggttggagaagattatagctacaattcatgggaagaattttcttcatgtagatgctaa
3421 gcaacaatcatgtcatgaatggcacaagaaaaatgtgatcatcatcttgattagttctaa
3481 gtttccaaccattgtgaaaagagatatttcaaaggttagtgataaacctcttgtacacat
3541 ttgtttgaaacaaacaatttaagaagagctattgccacttggggaaataacccaaactaa
3601 ggtatcataatgttatgaatgttaacaagggttgtgattgtatggtgaaaggagaagaac
3661 aagaagatgtaattgagatgatgttacgtactatagaaagaaatataagagaatatgatt
3721 agatattctagaagttgggtaagtaaggaattggagggaaatgttgatgagttgttaggg
3781 agccagctgttctgtagttggcaagtgagctgttctacagttgacaaaatgagacagttg
3841 aggcagttatgagatagttgttgtataaataactcagctatatctttcttgtattcattc
3901 cagaatttacaagaaatacaataactactctctattctcttctctctctctctctctttt
3961 ctctctactcttctattcttttctgttagagttctcaaaccctaacattggtatcagagc
4021 tttgcatccaagaaactagtgggtaattctttcttaaaggttgcagccatgactaggtct
4081 gttgtggttggcccagaaatggtacggattgcagaattggaggagcaaatgagaaatttg
4141 caggaagtaactcagaggaatgctgaggaactgaatgatttcaagaaggagatgaaagag
4201 gaattaaacaagacttctttaaccaataatgccattttggaggacttgcgacaaatgatg
4261 atcactcatgtggtggaaaggaataagggcagcagtagttcaaatgagggtggaagcaga
4321 gtcgaaaacaggggaatgcaatctaaccctgtagtgtagccagttcctgtagtacattca
4381 gtccctataatccaatcaattcctgcagataatagggggattattacctattcctaacatg
4441 ggtgctatgtctccattattgcctaaaattgaattagttacttttgaaggtaaagaacct
4501 agggcttggttgagaaaatgtgttaagtattttgaggtgtatatggtccctcaagatcaa
4561 agagtgcaattagcaagtcttttcttgcttgatagggctgatgcttggtttcacaattgg
4621 gaaaaaggagagaaacattcttgggaggaatttgagaaggaaatttgtagtaggtttggg
4681 gaggatggattagaagatatagtggaggaatttatgaaactgagacagtaagggatagtg
4741 ggggaatatcaagatgagtttgaggatttaagaataaggatggagaggctattacctaat
4801 ttgggggaatcttactttttgtcaggatttataggaggtttaaaagatgaaatcagatta
4861 atggtaaagatgatgaaacctgtcactctttcccaagctgtggaaatagctagattgcaa
4921 gaacaattgttagaaaacaccaagaaacctggatctgcaactaattcttccaaattcaaa

4981 gccatgactaatagtccgacaacttatttcaattcttcttttcagtattctagaccttat
5041 caaacctatcaataccctcctagaccaccaaattttgattctcaaaataaacccaacccc
5101 cttgttaccaagcaacaatcagtaggtagtactgctacagttactaatatttccacagca
5161 gcctcaaaacctactaccaatcagactccaccaacaagaaacttacctaaaccatgtttt
5221 agatgtggtgaaaagtattttccgggccatcaatgtaagtcaaagacattgaatgctttg
5281 tgtatggatgggaaagatgaagaattgaatgaggagtggcatgatgtgggggctgatata
5341 gaagaattgggagaggctggttccaccttatctgttcatgctttagagggtagtcatggg
5401 gctgacactataagaatgttagggattcacaaaaataggcaattggtgatcctcattgat
5461 agtggcagtaccactagttttatggacaggaggatagaacaggaactgaaattagacttg
5521 ctacagattccatttaaagcagttactgttgctgatggaagaaaattgggttgtaaccat
5581 ctatgtcaacaatttaaatggaaaatgcaacataatgactttacgtttgacttcaaaata
5641 ttggagttaggtggatttgacatgattttgggagttgattggttaaaagatcataatcct
5701 gttctttttgattttgctacatcttgtagtacaattgcaaaggatggcgaacgcattcaa
5761 ttgcagggaattggtgaggaaaatttgcaatctggtttgtggcattctgataatttgttg
5821 agcaaaggagaggtggattttcaaactcctttattctgtgttgtgaattccagtctgtca
5881 atagattctttggctactgcaactgctgtttctgttccaaatgagagcctgccggattta
5941 attcagaagtacaagggtgtttttgcagaacctaaggggttgcctccatttaggagtcac
6001 aatcatgctattcccttacaatcaaatgctcagcccatcaatattagaccctacaggtat
6061 ccttattatcaaaaggctgaaatagaaaaacttgtagctgatatgatagaatcttcaata
6121 attcagcctagcactagcccatattcctctccatttcttttggtgaagaaaaaagatggc
6181 acatggaggttttgcattgattatagaaggttgaatgatcaaacaataaaagataagttt
6241 cccatccctattattgaagatctattggatgaattgaatggggctaaagtgttttctaag
6301 atagatttgagggccggatatcactaaattaggatgttgcccgaggatattcctaagaca
6361 gcctttagaacacaccatgggcattttgaattcaaagtgatgccttttgggctcactaat
6421 gctcctgcaacatttcaagccttaatgaatcacatcttccaaccatttttgaggaagttt
6481 gtgttagtatttttttgatgacattttggtgtttagcaaggatatggagtctcatttgctg
6541 catttggaggaagtttttaaagtactacaggcccaacaattatttgcaaagcagtctaaa
6601 tgttttttttgggcaaactgagattgaatacttggggcatataatttcaggggcaggggta
6661 tctaccgaccctaagaaggtggctgccatggttgattggcctgtgccttcgtctgttaaa
6721 gaactcagaagtttttttaggccttactggatattataggaaatttgtaaggcactatggc
6781 attatctgcaggcctcttactgaattattgaagaaagattcttttcattggaataaggta
6841 gcccaaacagcatttgatactttgaggagattgatgtctgaagcaccagtccttgctctt
6901 cctaatttttctaagccatttattgtggaaactgatgcaagcaattggggaatgggtgca
6961 gttttatagcagcaagggcatccaattgcctatatttctaaggcatttggtcctaggagt
7021 caggctatgtcagtatatgaaaaagaattattggctattacttttgcagtcagcaaatgg
7081 agacattatttggagcaagggcagttctttattaagactgatcatgaaagcattaagtat
7141 cttttggagcaaaggttacataacaatttgcagcagaagggtatttcaaagttgcttggt
7201 ctggattacaaaattttgtatagaaagggcattgaaaataaggtggcagatgctttatcc
7261 agaaggtgtgttgatgtggattcttattcttatgctatgtattcagtggtggttccagtt
7321 tggatgcaaaagttaattgctagttatgaaaatgatgggaaagcctctgacttactccag
7381 cagttgtttttggataaggatgcagtttctgggtatcaattgaagaatggtattttgtat
7441 tataaagatagaatgtatgtggggactactaccaatttgaggcagttgttgttggaatct
7501 tatcatagttcagcagtgggaggccattcaggtgttcatgctacttatttgaaattaaag
7561 aaaattttcttttggcctgctatgcttaatgatgtcatgcagtgggttcatacctgtgat

7621 acttgtgctagatgtaagggagagcactgtgcttatccagggttattacaacctctacca
7681 attcctactcaagcctggcagcaggtgtctatggattttattgaaagattgcccaagtca
7741 aaggggaaagacactattttggtagttgtttgcagatttactaaatttggccattttatt
7801 tcactagcacatccattttctgctgtttctgtagctaaacttttcattgatcacattttt
7861 aagttgcatggagctcctcaagttattgtttctgacagagataaggtgtttactagccta
7921 ttttggcaagaattgtttagaagtatgggaattaaacttaattttagttctgcctatcac
7981 cctcagtctgatggacagactgaaagagttaatcagtgtctgcagaattatttgaggtgc
8041 atggttcatttgaggcctaccaattggagctcttggttacctatggcagaatggtggtac
8101 aattcctcttatcaaattgctattcagattactccatttgaagcactgtatgggtatagt
8161 ccacctctgtttcatgaaaattttactcttgattcctcagttggggttgtagggagattg
8221 ttacaagagagacagcatcttaataatctcttaaaggaaaatttacaagtggcccaacat
8281 aggatgaagcagcaagctgacaagaggagaactgagagggaatttgttgttggggattgg
8341 gtgtatttgaaacttcagccttatatgcagacttcagtatctgtcagacaaacccttaaa
8401 ctttctgctaagttttatgggccattcagaattcttgctaagattgggaaagttgcttat
8461 caattagacttgcctccaacagctactattcaccctgttttccatgtatccatgcttaag
8521 aggaaggttggggatggtgtggtggttgctactgatttacctgttatgcaagatgaccag
8581 attaaagttgttcctgagcaggttttgcagaccagaataattgaaagaggacaccagaga
8641 gtagaacagggacttattaaatggcttaatctgtcaacagaggatgccacttgggaagat
8701 agaagctttattgaagggcaatttcctgaagtctccctttcttggggacaagaaagtgct
8761 aaaggagggagtattgttacgtactatagaaagaaatataagagaatacgattagatatt
8821 ctagaagttgggtaagtaaggaattggagggaaatgtggatgagttgttagggagccagc
8881 tgttctgtagttggcaagtgagctgttttgtagttggcaagtgagctgttctacagttgg
8941 caagatgagacagctgaggcagttatgagatagttgttgtataaataactcagctatatc
9001 tttcttgtattcattccagaatttacaagaaatacaataactactctctattctctttc
9061 tctctctctctctttctctctactcttctattcttttctgttagagttctcaaaccc
9121 taacacaatttccccactttacattgggagacacctacataggcttttgaagaactacaa
9181 gagttggtggactcgtgcgcacaaggatgggcctagccaagtgtaacgggtggacgtgcg
9241 tataattcgcgaggacttgatccagtttcctttttcatagccttttattttcatttatat
9301 ttttccaaagaaatatccttacctaaaaacatgtttttgacctagttaaaactagattcc
9361 tatttttctctacctagccaaaactgaactccatttttttggtacttagctattttatt
9421 aatgttacataatgtctcttttatttttcctctcttgaacgttactaacattctattttt
9481 tgcttatatagggacatgtacattgagcctaagacactacaaaactctttttcttctacc
9541 tttctacgtctaggtacataattaaaattacgtttgttgttcctatgtcctaattattga
9601 ttgtacaataattacaaggtctattgatgcctggaggatatagttcctttgcacttagtg
9661 atgtagcattttctagagattttaggactacctattttagataaaaaatgggaagtagag
9721 ttttattttatttaggaataaataaaggtcatgatacacgataaaggtcatattacttct
9781 caatgcccacatcattcattagctttagagcaagcatgcaaacatgagcatgagggtgaa
9841 cataatgattcctcagactataaggaagaagttatatgacctagagagtattttgggaag
9901 ggtgagctagatttaggtgatgtgcattgcaatgttgttcgatgtgttttgtcaactatt
9961 gtggattgttatggaaagtcaatcactatattatttctctgtatattctatattctgtat
10021 ttctatttaggatttcttatttatgatttcttcctaattaatagaacacaattataggaa
10081 tcaattgtatatatatacccatgtacagattaattgaaatcaaggagaattatctctttc
10141 tacatggtaccagagcaggtcatctatctagggtgactatttgttctcatagttacatag
10201 tcttcctcattataggatgaatcgtaaggagcaagctaagtagaataggcaagttgaata

10261 aaggggtttgtgagacttagccttagtccatgtgttgtgcttgcaatgttgacacctaag
10321 aaagatggctcttggagaatgtgtgtggatagtagggccattaacatgatcactgttaag
10381 tatagatttccaattcctagacttaataggttagatttccttataggttcttgttggctc
10441 tctaaagttgacttgcatagtaggtatcattaaatacgcattaagctaggtgatgaatag
10501 aaaattgcttttaaaacctaggaatgattgtatgacaggttagtgatgccctttggattg
10561 tctaatgcacttagcacttttatctgttagtttttgctaacactaactacagtaaggaaa
10621 caatgaatacaatctatagagtgaaaaatggtctgctgatgattgctcttttagagaggt
10681 taagtttttgaacttttcaatcggtgttgagtgttgacttgatataataattgagaaatg
10741 gaggtgatagaacaatgcaatcataacaacaactaagtcttaatcacaaactagtcggca
10801 tcgactatatagatcatttttcaccattaagctctattaaacattaggtccgcattaata
10861 tccaagacctataaattctttgatacggagatgatagaacaatatgataaataaattggc
10921 ttaatcaatatacatattaaatggcccggtacaagacaaagaattaatatattaattttt
10981 tttttggagtaaatggtatcaaatctaaggttggaagtttaggatccagatggtggcact
11041 acacttagaactcaaggctaagcttcatcaatcttacagagtttgatgcatctataagaa
11101 gataaaatccatgaaatggtggtttgttatttgaatagcctttttttagcgccaatgtcaa
11161 gcttgaggtgcattttgttttgactcagaatttccatgttttgaaggttatttgatgttg
11221 ttaggaacattcgatattagttaggatctttggtagttattagggaatttattcagtgtg
11281 gtttttttaaaaggtatttggcaagtttacaaagtaacagtactttttaacaatccttaaa
11341 tagagtccttttgcaaatcaccaccttaatggctgtaagcattattatataatcactact
11401 atggctattacacttacccaatttttaaaaaaaataaatggccaacattgtagatgtaatt
11461 aattacatttataacttctgcatttactgagcatatatcacattccttgcatacatatgg
11521 ttttaaatggtacgcaacaatcaaattaaagctccatttgttccatggataataacttgt
11581 tcgtgaaaatatttttttaagaaaataaattattttctattgttttcttacaattctgaaa
11641 atcgcttagcaactatttatgtcatgtcattcatcatacattaaaaaatattgattttc
11701 tttaaacatataagattatgaaattgttttgatttgtaaaaatttcaatttttaatttat
11761 cattttcttagatatatattttttttaaatctatctttgaatatgagaaaaaaattatataa
11821 aaatggtaaattacagctgtcataaatgatcaatgttttcctatgatccatttctatgtc
11881 cttcctttctaacaacattaatggttatgttttctccctcgttccttctattattttctt
11941 cttgtgttcatctctactattattactcttcttcatctccatctccactagttcattcgt
12001 ggtgatatctgtaaaaccataataaatttgcttaatgcatgtgatagcggaatgaaacat
12061 gcccaaattgaaacacgaggaggtaatagctcttgtaattttatgatggccttttatgta
12121 ctaagttaatgtttatttggactttagagggagggtaagagttagtgagggtgatattaa
12181 ccccctttaattgtattttgtttacctatcccttatcagggaaggagggggggagagggcgg
12241 ctatagagaagaaaaaaaaatattttttttaatttcttaacatttgtcactagctaaatt
12301 gcgataatatataataaataaatacctttttgtatttggaaaaattactatttagatctctgt
12361 attttagagactatttagtctttgtattttgaaaaatatattatttactctccatatttg
12421 cttccgttaaactctttagtcctttcgtcaaattttctgttagtcaatggattttaatgc
12481 tagtcaaattactatttcattattttattttagtgaaactaatgagttgatctctctatt
12541 tttaaaaaaaatatatattatatagtcattttattttagtgaaactaattagatgttccct
12601 atattttaaaaaatacaatattctcaaaaacatatttttaaatgcagaattttgttttgg
12661 agacttgaatttgctttttttttgaattgtacaagtattttcattcaattctgtgaacatc
12721 atttttgtgttttctttattgaaaaataatttttttctgaattatcgtcttgattacttgg
12781 atatttaggggaactgattaacctttttgtacatacaacttactattctctatcaaaaga
12841 tgaaaaatagagagagaatgaggggggagtaggttgtggatgtagaaaagaaataacact

12901 gggagagagataaagaagagtaggagagaaataagtgggagaggatcagagtagtttgag
12961 tgtaaaaaataactggagggaatagagttaacagaaccaaattataagattaattaattt
13021 gtttttaaaatacatggaataattaattagttttattgaaataaatgaactaaatagtag
13081 tttgactagtattgactaacggaaaaattgatggatgactaaatagtttaacgaactaaa
13141 aataaaggaattaaatggtatacttttcaaatatagggactaagtagttagttttcttaa
13201 catatagggactatatagtaattttccctttgtttttttaatattgtaagaaacaaagtt
13261 agttgttactcataacttctgtcatttttttgctcccttaattttaacactctttaacttt
13321 tacttttgtaacttttaactttcaattcttaaacttcaagacaaatgcaccctaacagct
13381 aataatagggcactagaaatggtacttttacaatttggagagagattgattaaaactctc
13441 atgttaatgacaaatgatctaggcatttttttgcaatgttctttggaaacaatttcgatta
13501 ttatgctgagacaattaaacaatttttatgaatgttctggaaataattgatcgtatcatc
13561 tgagtatctacaataagcaatataactgagtctgaactataacacaaaggcaatttactt
100 V I G S I T G V
13621 ggtaaatcatgtgtcattcggcttttacacttcccagGTAATCGGATCTATCACTGGAGT
108 R Y I I E Y F P E I G F G P R L N V D I
13681 TAGGTACATTATTGAGTACTTTCCTGAAATAGGATTTGGGCCACGCCTCAATGTCGACAT
128 H H G A L T E G L L T F A I V T I S L G
13741 CCATCATGGCGCACTCACTGAAGGATTGCTTACATTTGCAATTGTTACCATCTCTCTTGG
148 L S R K I P G S F F M K T W I S S V S K
13801 GCTTTCAAGAAAGATCCCTGGAAGTTTCTTCATGAAGACATGGATATCAAGTGTCTCTAA
168 L A L H I L G S D L T G G C M N P A S
13861 ATTAGCTCTTCACATACTTGGCTCCGATCTGACTGGCGGTTGTATGAACCCAGCCTCTgt
13921 aagtgatatcaatatcattagcatttgatgcacatatcatacatctgtttttgttgccaa
187 V M G W
13981 aactttggaaaacagagatgaactactagagctttttaaaatgtgtagGTGATGGGATGG
191 A Y A R G D H I T K E H I L V Y W L A P
14041 GCTTATGCCCGTGGAGATCATATAACTAAGGAGCATATACTTGTATATTGGCTTGCTCCA
211 I E A T L L A V W T F K L L V R P Q K Q
14101 ATAGAGGCAACTCTTCTGGCAGTATGGACATTTAAGCTACTAGTTCGACCCCAAAAGCAA
231 E K E E S K S K S D *
14161 GAGAAGGAAGAGTCGAAGAGTAAATCAGAT**TGA**tgtttgatgataatcaaactgaagcaa
14221 tccactgtgttttctccttgtaaagttgcttggatcagtgtctatttattgttaatttat
14281 ctccagcatcttcttgttggtcagaactctctatcagatgatggccaaccgtgttagcta
14341 gcttagcagttctgcttgattattttaataaatgtaaatacttttgttagctaatgcaaa
14401 attactatggaataaggttcaaatttcaaattgcccattgtacttgtcggggatattcaa
14461 tttagttaatttttaatttgtg
