# Supplementary material for: Genome-wide identification of rubber tree (Hevea brasiliensis Muell. Arg.) aquaporin genes and their response to ethephon stimulation in the laticifer, a rubber-producing tissue
Source: BMC Genomics. 2015 Nov 25;16:1001. doi: 10.1186/s12864-015-2152-6 (PMC4658816; doi:10.1186/s12864-015-2152-6)
Supplement: Additional file 5: — Alignment of predicted amino acid sequences of rubber tree aquaporins with structure determined Spinach SoPIP2;1. (PDF 275 kb) [file 12864_2015_2152_MOESM5_ESM.pdf]

Additional file 5: Alignment of predicted amino acid sequences of rubber tree aquaporins with structure determined Spinach SoPIP2;1.

```
SoPIP2;1        ----------------MSKEVSEEA-----QAHQHGKDYVDPPPAPFFDLGE-------- 31(60)
HbPIP1;1        ------MEGKEEDVRLGANKYRETQPIGTSAQSQDDKDYTEPPAAPLFEPTE-------- 46(60)
HbPIP1;2        ------MEGKEEDVRLGANKYRETQPIGTAAQSLDDKDYTEPPPAPLFEPTE-------- 46(60)
HbPIP1;3        ------MEGKEEDVRLGANKFTERQPIGTSAQS--DKDYKEPPPAPLFEPGE-------- 44(60)
HbPIP1;4        ------MEGKEEDVRLGANKFTERQPIGTSAQT--DKDYKEPPPAPLFEPGE-------- 44(60)
HbPIP1;5        -----------------------MQPLGTSAQT--DKDYRDPLPAPLFEPVE-------- 27(60)
HbPIP2;1        ----------------MAKDVEVGG----QGGEFQAKDYNDPPPAPLIDAEE-------- 32(60)
HbPIP2;2        ----------------MAKDIEVGG----DGSEFRAKDYHDPAPSPLIDAEE-------- 32(60)
HbPIP2;3        ----------------MAKDVEVAE----NPGEFSAKDYHDPPPAPLIDVEE-------- 32(60)
HbPIP2;4        ----------------MAKDVEVAE----NPGEFSAKDYHDPPPAPLIDVEE-------- 32(60)
HbPIP2;5        ----------------MVKDVTEQ-------GSFPAKDYHDPPPAPLIDAVE-------- 29(60)
HbPIP2;6        ----------------MVKDITEQ-------GSFSAKDYHDPPPAPLIDAVE-------- 29(60)
HbPIP2;7        ----------------MAKEVSEE----RQP----GKDYVDPPPAPLIDMAE-------- 28(60)
HbPIP2;8        ----------------MAKEVSEE----TQPT--HGKDYVDPPPAPLIDVAE-------- 30(60)
HbPIP2;9        ----------------MAKEVTEE----AGEASQQERDYVEPPPAPLFDPEE-------- 32(60)
HbPIP2;10       ----------------MVKEMGEEGSFEHEIHGQHGKDYVDPPPAPLLDMEE-------- 36(60)
HbTIP1;1        ---------------------------------------MPIRNIAVGHPQE-------- 13(60)
HbTIP1;2        ---------------------------------------MPIRNIAVGHPQE-------- 13(60)
HbTIP1;3        ---------------------------------------MPINRVAIGLPRED------- 14(60)
HbTIP1;4        ---------------------------------------MPINRIEVGLPRQD------- 14(60)
HbTIP1;5        ---------------------------------------MPITRIAVGNPGE-------- 13(60)
HbTIP1;6        ---------------------------------------MPVARIAVGNPGE-------- 13(60)
HbTIP1;7        ---------------------------------------MPITSIAIGSPAE-------- 13(60)
HbTIP1;8        ---------------------------------------MAITSIAIGSPAE-------- 13(60)
HbTIP2;1        -----------------------------------------MARIAFGRFDD-------- 11(60)
HbTIP2;2        -----------------------------------------MARIAFGRFDD-------- 11(60)
HbTIP2;3        -----------------------------------------MPMIAVGSIGD-------- 11(60)
HbTIP2;4        -----------------------------------------MARMAFGSFGD-------- 11(60)
HbTIP3;1        ----------------------------------------MPRRYALGRAEE-------- 12(60)
HbTIP3;2        ---------------------------------------MSTRRYAFGNAEE-------- 13(60)
HbTIP4;1        -----------------------------------------MAKIALGSQRE-------- 11(60)
HbTIP5;1        -----------------------------------------MAPSSLNARFKQ------- 12(60)
HbTIP5;2        -----------------------------------------MAPTSLNDRFKQ------- 12(60)
HbNIP1;1        --------MADEISGTNGKHGVVLDVEGDNPCYSPPPCNDRPPCASKTKEDSI------- 45(60)
HbNIP1;2        -------MAQAEISVANGRHGVVLDVKDDNPCNPAPAGNKPPPCASKTKEDSI------- 46(60)
HbNIP2;1        ------------MATIDPNPNNSASIGDG-----LVSVENPKSQLVESFG---------- 33(60)
HbNIP3;1        ------------MASPNSITSVVSPKPQLP---TKYSVAAEAKASRSREWFLT------- 38(60)
HbNIP4;1        ------------MSGADDHE--IRDVEEG-----QQEDSSD--SQNNSK----------- 28(60)
HbNIP4;2        -----MATAHADIIEEEEEE--VSKIEQG-----LPPSTTADASANNTVGPCL------- 41(60)
HbNIP5;1        ----------MPESEAGTPTV-SAPATPGTPGGPLFSSLRVDSLSYDRKSMPR-CKCLPV 48(60)
HbNIP6;1        ----------MDNNNEEVPSAPSTPATPGTPGAPLFGGFRAERSGTNRKSLLKGCKCFSV 50(60)
HbNIP7;1        -------MKMKQLLEDQLPYPDISNNSSNSGLSRDCPEMGSNAMSIDGDVFAKYSVSRCL 53(60)
HbXIP1;1        -------MDLVATQGDNNHQPFSKSVENCDAINDFKGTKSPKTSFLVFIGAHE------- 46(60)
HbXIP1;2        -------MDSHNT----------------------KGSQYPMMTFLYRIGAYE------- 24(60)
HbXIP1;3        -------MDLDLVLSHDAGNQAFPNQLDGQNKNINDSLESPKKTFLSCIGVHE------- 46(60)
HbXIP1;4        -------MDLDLVLSHDAGNQAFPNQLDGQDKNIKGSLESPKKTFLSCIVVHE------- 46(60)
HbXIP2;1        MADNASRVVEDEENGYGGRKVQPFASTPRPDMDKTEGKKHHPTTLSRILGFED------- 53(60)
HbXIP3;1        ----------------------------------------------------M-------  1(60)
HbSIP1;1        ------------------------------------------------------------
HbSIP1;2        ------------------------------------------------------------
HbSIP1;3        ------------------------------------------------------------
HbSIP2;1        ------------------------------------------------------------
```

TM1

```
SoPIP2_1     ------------------LKLWSFWRAAIAEFIATLLFLYITVATVIGHSKETVV-----  68(120)
HbPIP1;1     ------------------LTSWSFYRAGIAEFIATFLFLYISVLTVMGVVKAPTK-----  83(120)
HbPIP1;2     ------------------LTSWSFYRAGIAEFIATFLFLYISILTVMGVVKAPTK-----  83(120)
HbPIP1;3     ------------------LCSWSFYRAGIAEFIATFLFLYITVLTVMGFSKSTNK-----  81(120)
HbPIP1;4     ------------------LSSWSFYRAGIAEFIATFLFLYITVLTVMGFSKPNNK-----  81(120)
HbPIP1;5     ------------------LKSWSFWRAGIPEFFATFFFLYVTVLTVMCFSRSPNK-----  64(120)
HbPIP2;1     ------------------FTQWSFYRAIIAEFIATLLFLYITVLTVIGYKSQTDPAKNAD  74(120)
HbPIP2;2     ------------------LTKWSFYRAIIAEFIATLLFLYITVLTVIGYKSQTDPAKNAD  74(120)
HbPIP2;3     ------------------LGKWSFYRALIAEFIATLLFLYITVLTVIGYKSQTDPAKNSD  74(120)
HbPIP2;4     ------------------LGKWSLYRALIAEFIATLLFLYITVLTVIGYKSQTDPLKNAD  74(120)
HbPIP2;5     ------------------LTKWSFYRALIAEFIATLLFLYITVLTVIGYKSQTDPAKNAD  71(120)
HbPIP2;6     ------------------LTKWSFYRALIAEFIATLLFLYITVLTVIGYKSQTDPAKTHD  71(120)
HbPIP2;7     ------------------IKLWSFYRALIAEFIATLLFLYITVATVIGYKKQADP-----  65(120)
HbPIP2;8     ------------------LKLWSFYRALIAEFIATLLFLYITVATVIGYKKQADP-----  67(120)
HbPIP2;9     ------------------LGLWSFYRAVIAEFIATLLFLYVTVATVIGYKKQTDP-----  69(120)
HbPIP2;10    ------------------LRRWSFYRAL-AEFVATLLFLHVSVATVIGYKSQADP-----  73(120)
HbTIP1;1     ------------------ATHPDALKAALAEFISTLIFVFAGEGSGMAFSKLTNNG----  51(120)
HbTIP1;2     ------------------ATHPDALRAALAEFISTLIFVFAGEGSGMAFSKLTDNA----  51(120)
HbTIP1;3     ------------------VVHPGALKAALAEFISTAIFVFAGQGSGMAFSKLTDNA----  52(120)
HbTIP1;4     ------------------VTHPSALKAALAEFISTLIFVFAEEGSGMAFSRLTDNA----  52(120)
HbTIP1;5     ------------------ASQPDALRAALAEFFSMIIFVFAGEGSGMAFNKLTDNG----  51(120)
HbTIP1;6     ------------------ASQPDAIRAALAEFFSMIIFVFAGEGSGMAFSKLTNNG----  51(120)
HbTIP1;7     ------------------ASQPDALKAALAEFISMLIFVFAGEGSGMAFNKLTDNG----  51(120)
HbTIP1;8     ------------------ASQPDALKAALAEFISMLIFVFAGEGSGMAFNKLTDNG----  51(120)
HbTIP2;1     ------------------SFSLGSFKAYLAEFISTLLFVFAGVGSAIAYNKLTNDA----  49(120)
HbTIP2;2     ------------------SFSLGSFKAYLAEFISTLLFVFAGVGSAIAYNKLTYDA----  49(120)
HbTIP2;3     ------------------SFSIGSIKAYLSEFIATLLFVFAGVGSAIAYSKLTADA----  49(120)
HbTIP2;4     ------------------FFSIGSIKASLSEFIATLLFVFVGVGSAIAYSKLTTDA----  49(120)
HbTIP3;1     ------------------ATNPDSMRAALAEFVSTLIFVFAGEGSVLALDKLYRET----  50(120)
HbTIP3;2     ------------------ATHPDSMKAALAEFVSTLIFVFAGEGSVLALDKLYRET----  51(120)
HbTIP4;1     ------------------ATQPDCIKALIVEFITTFFFVFVGVGAAMAADKLVGG-----  48(120)
HbTIP5;1     ------------------SVTPDALRSYLAEFISTFFYVFEVVGSAMAARKL--MT----  48(120)
HbTIP5;2     ------------------SVTPDALRSYLAEFISTIFYVFVVVGSAMASDTEKLMP----  50(120)
HbNIP1;1     -----------------LSISVPFIQKLIAEVVGTYFLIFAGCTAVAVNLNFDKE-----  83(120)
HbNIP1;2     -----------------SSISVPFIQKLIAEMVGTYFLIFAGCTSVAANLNYDKV-----  84(120)
HbNIP2;1     -----------------KNYPPDFLKKVVAEVIATYLLVFVTCGAAAISTSDERR-----  71(120)
HbNIP3;1     -----------------DDGSPSVLQKIIAELIGTYILIFVGCGAALTDKVQK-------  74(120)
HbNIP4;1     -----------------RSGFCSSNATLIAEMVGTYMLIFCGCGSVAVNYIYG-S-----  65(120)
HbNIP4;2     -----------------STSFVSITQKLIAEVIGTYFVVFAGCGVVTVNKIYG-S-----  78(120)
HbNIP5;1     N--APTWGQSHTCFTDFPSPDVSLTRKLGAEFVGTFILIFAATAGPIVNQKHNGV----- 101(120)
HbNIP6;1     EEWALEEGRLPPVSCSIPPPPVSLARKVGAEFIGTLILIFAGTATAIVNQKTQGT----- 105(120)
HbNIP7;1     P----------------EGMDLNPARMVLAEMMGTFVLMFCVCGIIGTTQITRGQ-----  92(120)
HbXIP1;1     ------------------FFSREMWRAALVELVATACLLFTLTISIISCLESHVPE----  84(120)
HbXIP1;2     ------------------FFSPELCRAVVTEMAATTCLLFMLTTTIIARLESHETE----  62(120)
HbXIP1;3     ------------------LLSPETWKAAITELVSTACQLFTLITMVTACLESHVAE----  84(120)
HbXIP1;4     ------------------LLSPEKWKAAITQLVSTACLLFTLITMVTACLESHVAE----  84(120)
HbXIP2;1     ------------------LSSLHVWRASLAEALGTASLVFAMDTIVISSYETETKT----  91(120)
HbXIP3;1     ------------------VLTFQVWRASFSEFLGTAVLVFVIDTVVISTIESETKI----  39(120)
HbSIP1;1     ---------------------MGVIKAAIGDAILTSMWVFSMPLLGILASAVAAYIG-VE  38(120)
HbSIP1;2     ---------------------MGAIKAAIGDAVLTFMWVFCSSMFGLFTSLIATALG-VQ  38(120)
HbSIP1;3     ---------------------MGAIKSAIGDAVLTFMWVFCSSMFGFFTSLIATALG-VH  38(120)
HbSIP2;1     ------------------MSSAVTLRLIISDFVISFMWVWSGALIKMFVNRVLGVG----  38(120)
                                           :       :
```

```
                            TM2                    HB           TM3
SoPIP2_1      -CGSVGLLGIAWAFGGMIFVLVYCTAGISGGHINPAVTFGLFLA---RKVSLLRALVYMI 124(180)
HbPIP1;1      -CSTVGIQGIAWSFGGMIFALVYCTAGISGGHINPAVTFGLFLA---RKLSLTRALYYMV 139(180)
HbPIP1;2      -CSTVGIQGIAWAFGGMIFALVYCTAGISGGHINPAVTFGLFLA---RKLSLTRALYYMV 139(180)
HbPIP1;3      -CATVGTQGIAWAFGGMIFALVYCTAGISGGHINPAVTFGLFLA---RKLSLTRALFYII 137(180)
HbPIP1;4      -CTTVGTQGIAWAFGGMIFALVYCTAGISGGHINPAVTFGLFLA---RKLSLTRALFYMI 137(180)
HbPIP1;5      -CASVGVQGIAWAFGGMIFVLVYCTAGISGGHINPAVTFGLTLA---RKVSLTRAIFYMV 120(180)
HbPIP2;1      PCGGVGILGIAWAFGGMIFILVYCTAGISGGHINPAVTLGLFLA---RKVSLVRAILYMA 131(180)
HbPIP2;2      ACGGVGILGIAWAFGGMIFILVYCTAGISGGHINPAVTFGLFLA---RKVSLVRAVLYMA 131(180)
HbPIP2;3      ACGGVGILGIAWAFGGMIFILVYCTAGISGGHINPAVTFGLFLG---RKVSLIRALLYMV 131(180)
HbPIP2;4      ACGGVGILGIAWAFGGMIFILVYCTAGISGGHINPAVTFGLFLG---RKVSLIRALLYMV 131(180)
HbPIP2;5      SCGGVGILGIAWAFGGMIFILVYCTAGISGGHINPAVTFGLFLA---RKVSLVRAVMYMV 128(180)
HbPIP2;6      ACGGVGILGIAWAFGGMIFILVYCTAGISGGHINPAVTFGLFLA---RKISLVRAVMYMV 128(180)
HbPIP2;7      -CAGVGLLGIAWAFGGMIFILVYCTAGISGGHINPAVSFGLFLA---RKVSLIRAVAYMV 121(180)
HbPIP2;8      -CGGVGLLGIAWAFGGMIFILVYCTAGISGGHINPAVTFGLFLA---RKVSLIRAVAYMV 123(180)
HbPIP2;9      -CAGVGFLGIAWSFGGMIFILVYCTAGISGGHINPAVTFGLFLA---RKVSLVRAIAYMV 125(180)
HbPIP2;10     -CATVGFLGVAWAFGGMIFILVYCTAGISGGHINPAVTFGLLLA---RKLSLVRAVAYMV 129(180)
HbTIP1;1      ATTPAGLVAASIAHAFALFVAVSVGANISGGHVNPAVTFGAFVG---GNITLLRGILYWI 108(180)
HbTIP1;2      ANTPAGLVAASIAHAFALFVAVSVGANISGGHVNPAVTFGAFVG---GNITLLRGILYWI 108(180)
HbTIP1;3      SNTPAGIIMASLAHAFGLFVGVSTATNISNGHVNPAVTFGAFLG---GNISLLRGILYWI 109(180)
HbTIP1;4      SNTPAGIIMASLAHAFGLFVGVSTAFNISGGHVNPAVTFGAFLG---GSISLIRGILYWI 109(180)
HbTIP1;5      STTPAGLIAASLAHAFALFVAVSVGANISGGHVNPAVTFGAFIG---GNITLLRGILYWI 108(180)
HbTIP1;6      STTPAGLIAASLAHAFALFVAVSVGANISGGHVNPAVTFGAFIG---GNITLLRGILYWI 108(180)
HbTIP1;7      STTPAGLVAASLAHGFALFVAVSVGANISGGHVNPAVTFGAFVG---GHITFIRSVLYWI 108(180)
HbTIP1;8      STTPAGLVAASLAHGFALFVAVSVGANISGGHVNPAVTFGAFVG---GHITLIRSVLYWV 108(180)
HbTIP2;1      ALDPAGLVAIAICHGFALFVAVSVGANISGGHVNPAVTFGLALG---GQITILTGIFYWI 106(180)
HbTIP2;2      ALDPAGLVAIAICHGFALFVAVAVGANISGGHVNPAVTFGLALG---GQITILTGVFYWI 106(180)
HbTIP2;3      ALDPPGLVAVAVAHAFALFVGVAIAANISGGHLNPAVTFGLAVG---GNITILTGIFYWI 106(180)
HbTIP2;4      ALDPPGPVAVAVAHAFGLFVGVAIAANISGGHLNPAVTFGLAVG---GNITILTGIFYCI 106(180)
HbTIP3;1      EPPASGLVMIALAHALALFSAVSASINISGGHVNPAVTFGALVG---GRISVLQAFYYWV 107(180)
HbTIP3;2      GPPASGLVMIALAHALALFSALSASINISGGHVNPAVTFGALVG---GRISVLRALYYWV 108(180)
HbTIP4;1      --SLAGLLFVALAHALVVAVMISAG-HISGGHLNPAVTLGLLAG---GHITVFRSILYWI 103(180)
HbTIP5;1      GADPSSLVIVAIANSFALSSAVYIAANISGGHVNPAVTFSLAVG---GHISVPTALFYWI 105(180)
HbTIP5;2      GADPSSLVIVAIANAFALSSAVYIAANVSSGHVNPAVTFSLAVG---GHINVPTAIFYWI 107(180)
HbNIP1;1      ----VTLPGISIVWGLAVMVLVYSVGHISGAHFNPAVTLAFATC---KRFPWKEVPAYIA 136(180)
HbNIP1;2      ----VTLPGISIVWGLAVMVLVYSVGHISGAHFNPAVTLAFATC---KRFPWKQVPAYIA 137(180)
HbNIP2;1      ----ISKLGASVAGGLIVTVMIYAVGHVSGAHMNPAVTTAFAAF---RHFPWKQVPFYAA 124(180)
HbNIP3;1      ----LTIVGIAIAWGVVLMAAIYALGHVSGAHFNPAVSIALAAA---RKFSWKNVPMYIL 127(180)
HbNIP4;1      ----ITFPGVCVVWGLIVMVMIYSVGHISGAHFNPAVSITFAIF---RQFPIKQLPLYIL 118(180)
HbNIP4;2      ----VTFPGISVTWGLIVMVMIYTVGHISGAHFNPAVTITSAIF---RRFPFREVPLYIV 131(180)
HbNIP5;1      ----ESLIGNAACAGLAVMIIILSTGHISGAHLNPSLTIAFAAL---RHFPWMQVPAYIA 154(180)
HbNIP6;1      ----ETLIGLAASTGLAVMIVILSTGHISGAHLNPSVTIAFAAL---KHFPWKHVPVYIG 158(180)
HbNIP7;1      ----VALLEYASTAGLSVIVLVFALGPISGAHVNPAVTIAFAAF---GHFPWSRVPFYVL 145(180)
HbXIP1;1      -----PKLLVPFAVFIIAFFLLTTVPLSGGHMSPVFTFIAALK---GVITLVRALLYVL 136(180)
HbXIP1;2      -----PKLLIPIAVIVIAFLLLVVTVPLSGGHMSPIFTFISALR---GLITLVRALFNVL 114(180)
HbXIP1;3      -----AKLLVPVVVFSTIFLLLVVTIPVSGVHMNPTFTFIFALK---GVITFVRALVYIL 136(180)
HbXIP1;4      -----PKLLVPVVVFSTIFLLLVLTIPVSGGHMNPTFTFIFALK---GAITFVRALVYIL 136(180)
HbXIP2;1      -----PNLIMSALIAITVTILLNATFPISGGHINPVITLSAAFT---GLVSLSRAAIYIL 143(180)
HbXIP3;1      -----PNLILSCLVAITVTIILLATYPISGGHINPLVTFSAALT---GLISMTKAFIYIL  91(180)
HbSIP1;1      AMSVAGLFITINVATCFVLTFSLIGAALGGASFNPTATISFYAAGIKPDASLMSMAVRFP  98(180)
HbSIP1;2      HLFWASMFITTVLFFIFFFLFGLIAEFFGGASFNPTGTASFYAAGFGGDN-LFSMALRFP  97(180)
HbSIP1;3      HQFWASLFITTVIVFVFVFLFGLIAEFLGGASFNPTGTASFYAAGFGGDN-LFSMALRFP  97(180)
HbSIP2;1      HHEPRGEAIKATLSIINMIFFAFLGKITKGGAYNPLTVFSPAISG-DFSRFLLTVGARIP  97(180)
                                         .   .*
```

TM3 **TM4**

```
SoPIP2_1     AQCLGAICGVGLVKAFMK-GPYNQFGGGANSVAL------------GYNKGTALGAEIIG 171(240)
HbPIP1;1     MQCLGAICGAGVVKGFEGRHQYTLLGGGANSVNP------------GYTKGDGLGAEIVG 187(240)
HbPIP1;2     MQCLGAICGAGVVKGFEGRHQYTLLGGGANSVNP------------GYTKGDGLGAEIVG 187(240)
HbPIP1;3     MQCLGAICGAGVVKGFEGDRVYETLGGGANVVAH------------GYTKGDGLGAEIVG 185(240)
HbPIP1;4     MQCLGAICGAGVVKGFEGNRAYETLGGGANVVAH------------GYTKGDGLGAEIVG 185(240)
HbPIP1;5     MQCLGAIFGAGIVKGFQP-TPFETLGGGANVVNP------------GYSKGDGLGAEIVE 167(240)
HbPIP2;1     AQCLGAICGCGLVKAFQK-AYYNRYGGGANELAD------------GYSKGTGLGAEIIG 178(240)
HbPIP2;2     AQCLGAICGCGLVKAFQK-AYYNRYGGGANELAD------------GYSKGTGLGAEIIG 178(240)
HbPIP2;3     AQCLGAICGCGLVKAFQK-AYYTRYGGGANELSS------------GYSKGTGLGAEIIG 178(240)
HbPIP2;4     AQCLGAICGCGLVKAFQK-AYYNRYGGGANELSD------------GYNKGTGLGAEIIG 178(240)
HbPIP2;5     AQCLGAIAGVGLVKAFQS-SFYKRYGGGANSLAA------------GYSKGVGLGAEIIG 175(240)
HbPIP2;6     AQCLGAIAGVGLVKAFQS-SHYKRYGGGANSLAN------------GYSTGVGLGAEIIG 175(240)
HbPIP2;7     AQCLGAICGVGLVKAFMK-HPYNGLGGGANTVAP------------GYNKGTALGAEIIG 168(240)
HbPIP2;8     AQCLGAICGVGLVKAFMK-HPYNALGGGANSVAH------------GYNKGTALGAEIIG 170(240)
HbPIP2;9     AQCLGAICGVGIVKGIMK-DFYNAQGGGANTVAA------------TYSKGTALGAEIIG 172(240)
HbPIP2;10    SQCLGAIAGAGLVKAVMK-DDYKSLGGGVNSVSS------------GYSKGTALGAEIIG 176(240)
HbTIP1;1     AQLLGSTVACLLLKFSTG-----GLTTAGFALSS------------GVGVWNAFVFEIVM 151(240)
HbTIP1;2     AQLLGSTVACLLLKFSTG-----GLTTSAFALSS------------GVGVWNAFVLEIVM 151(240)
HbTIP1;3     AQLLGSTVACLLLKFSTH-----GMTTSAFALSS------------GVNVWNALVFEIVM 152(240)
HbTIP1;4     AQLLGSTVACLLLKFSTH-----GMTASAFSLSV------------RGECVECTCIRDCT 152(240)
HbTIP1;5     AQLLGSVVACLLLKFATG-----GLETSAFALSS------------GVSSWNALVFEIVM 151(240)
HbTIP1;6     AQLLGSVVACLLLKFATG-----GLETSAFALSS------------GVSSWNAVVFEIVM 151(240)
HbTIP1;7     AQLLGSVVACLLLKFATG-----GLETSAFALSS------------GVGAWNALVFEIVM 151(240)
HbTIP1;8     AQLLGSVVACLLLKFATG-----GWETSAFALSS------------GVGAGNALVFEIVM 151(240)
HbTIP2;1     AQLLGSIVACFLLKFVTG-----DLPIPTHSVAA------------GVGAIEGVVMEIVI 149(240)
HbTIP2;2     AQLLGSIVACFLLKFVTG-----GLAIPTHSVAA------------GVGAIEGVVMEIVI 149(240)
HbTIP2;3     AQCLGSIVACLLLQFVTN-----GKSVPTHGVAS------------GMNAFEGVIMEIII 149(240)
HbTIP2;4     AQCLGSIVACPLLYFVAN-----GKSVPTHGVAS------------GMNAFEGVVMEIVL 149(240)
HbTIP3;1     AQLLGAIVASLLLRLVTN-----GMRPVGFYIAS------------GVGEVHGLIMEMVM 150(240)
HbTIP3;2     AQLLGSIVASLLLRLVTN-----RMRPVGFYVAS------------GAAEVHGLILEMVM 151(240)
HbTIP4;1     DQLVASSAACLLLSYLTG-----GMATPVITLAS------------GVGYVQGVVWEILL 146(240)
HbTIP5;1     SQMLASVMACLLLRVVIV-----GQSLPTYTIAE------------EMTGFGASVIEGVL 148(240)
HbTIP5;2     SQMLASVMACLLLRVAIV-----GQSLPTYTIAA------------EMTGFGASVFEGVL 150(240)
HbNIP1;1     CQVIGSTLAAGTIRLIFTG---KQDQFTGTMPAG--------------SDMQSFVVEFII 179(240)
HbNIP1;2     CQVTGSTLAAGTIRLIFTG---KQDQFTGTLPAG--------------SDMQSFVVEFII 180(240)
HbNIP2;1     AQLTGAISASFTLRVLLHP----IKQVGTTSPSG--------------SDLQALIMEIVV 166(240)
HbNIP3;1     AQVLGATLACLTLKVLFHDQDDIQATMTQYKDST--------------SDLEAFIWEFII 173(240)
HbNIP4;1     AQFVGSLLASGTLYILLDV---EDEDFFGTKPVG--------------PHGRSFVIELIT 161(240)
HbNIP4;2     AQVLGSILASGTLALVFDV---TPNAYFGTVPVG--------------SNVQPLVIEIII 174(240)
HbNIP5;1     AQVSASICASFALKGVFHP----FMSGGVTVPSV--------------STGQAFALEFLI 196(240)
HbNIP6;1     AQVMASVSAAFALKGIFHP----IMGGGVTVPSG--------------GYGQAFALEFII 200(240)
HbNIP7;1     AQIVGSILATYVGKCVYDI----KPELMVTQPLQ--------------DCNSAFWVEFIA 187(240)
HbXIP1;1     AQCIGSIMAYMVIKNVMNNSAVEKYSLGGCMID----GNEG-----GIASGTALVLEFSC 187(240)
HbXIP1;2     AQCVGSIMAYLVIKSVMNENTAEKYSLGGCMVN----GNRS-----GVNAGTALILEFTC 165(240)
HbXIP1;3     AQCLGSTMAYLIVKRAMNPKIAEKYSLGGCSMG----GNGE-----GISAGTALAIEFAC 187(240)
HbXIP1;4     AQCLGSTMANLIVKRAMNPKIAEKYSLGSCSVG----GNGE-----GISAGTALAIEFAC 187(240)
HbXIP2;1     AQCLGGILGALALKAVVNSTIEKTFSLGGCTLSIVAPGPHG-PIVIGLGTAQALWLEIIC 202(240)
HbXIP3;1     AQCAGGVVGALALKAVVNSKIESTFSLGGCTLHIVAPGPDGRPTVIGLETGQALWLEIIC 151(240)
HbSIP1;1     AQAAGGVGGAKAILQAMPSKYKHLLKGPSLKVDL----------------HTGAVAEGTL 142(240)
HbSIP1;2     AQAAGAVGGALAILEVMPPQYKHMLGGPTLKVDL----------------HTGAIAEGLL 141(240)
HbSIP1;3     AQAAGAVGGALAILEVMPPQYKHMLGGPTLKVDL----------------HTGAIAEGVL 141(240)
HbSIP2;1     AQVIGSITGVRYIIEYFP-----EIGFGPRLNVD----------------IHHGALTEGLL 137(240)
              *   ..   .                                          .
```

```
                    TM4                              TM5                  HE
SoPIP2_1        TFVLVYTVFSATDPKRSARDS-----HVPILAPLPIGFAVFMVHLATIPITGTGINPARS 226(300)
HbPIP1;1        TFVLVYTVFSATDAKRNARDS-----HVPILAPLPIGFAVFLVHLATIPITGTGINPARS 242(300)
HbPIP1;2        TFVLVYTVFSATDAKRNARDS-----HVPILAPLPIGFAVFLVHLATIPITGTGINPARS 242(300)
HbPIP1;3        TFVLVYTVFSATDAKRNARDS-----HVPILAPLPIGFAVFLVHLATIPITGTGINPARS 240(300)
HbPIP1;4        TFVLVYTVFSATDAKRNARDS-----HVPILAPLPIGFAVFLVHLATIPITGTGINPARS 240(300)
HbPIP1;5        TFALVYTVLSATDAKRSARDS-----HVPILASLPIGFAVFLVHLATIPITGTGINPARS 222(300)
HbPIP2;1        TFVLVYTVFSATDPKRNARDS-----HVPVLAPLPIGFAVFMVHLATIPVTGTGINPARS 233(300)
HbPIP2;2        TFVLVYTVFSATDPKRNARDS-----HVPVLAPLPIGFAVFMVHLATIPVTGTGINPARS 233(300)
HbPIP2;3        TFVLVYTVFSATDPKRNARDS-----HVPVLAPLPIGFAVFMVHLATIPITGTGINPARS 233(300)
HbPIP2;4        TFVLVYTVFSATDPKRNARDS-----HVPVLAPLPIGFAVFMVHLATIPITGTGINPARS 233(300)
HbPIP2;5        TFVLVYTVFSATDPKRNARDS-----HVPVLAPLPIGFAVFMVHLATIPITGTGINPARS 230(300)
HbPIP2;6        TFVLVYTVFSATDPKRSARDS-----HVPVLAPLPIGFAVFMVHLATIPITGTGINPARS 230(300)
HbPIP2;7        TFVLVYTVFSATDPKRSARDS-----HVPVLAPLPIGFAVFMVHLATIPITGTGINPARS 223(300)
HbPIP2;8        TFVLVYTVFSATDPKRSARDS-----HVPVLAPLPIGFAVFVVHLATIPVTGTGINPARS 225(300)
HbPIP2;9        TFVLVYIVLSATDPKRNARDS-----HVPVLAPLPIGFAVFMVHLATIPITGTGINPARS 227(300)
HbPIP2;10       TFVLVYTVFSATDPKRKARDS-----FVPILVPLPIGFAVFVVHLATIPITGTGINPARS 231(300)
HbTIP1;1        TFGLVYTVYATAIDPKKG--------NLGIIAPIAIGFIVGANILAGGAFDGASMNPAVS 203(300)
HbTIP1;2        TFGLVYTVYATAIDPKKG--------NLGIIAPIAIGFIVGANILAGGAFDGASMNPAVS 203(300)
HbTIP1;3        TFGLVYTVYATAFDRNKG--------DVGIIAPLAIGFVVGANILAGGAFEGASMNPAVS 204(300)
HbTIP1;4        DVCLVYTVYATALDPKKG--------EVGIIALLAIGFVVGANILAGGAFEGASMNPAVS 204(300)
HbTIP1;5        TFGLVYTVYATAVDPKKG--------NVGIVAPIAIGFIVGANILAGGAFDGASMNPAVS 203(300)
HbTIP1;6        TFGLVYTVYATAVDPKKG--------NVGTVAPIAIGFIVGANILAGGAFDGASMNPAVS 203(300)
HbTIP1;7        TFGLVYTVYATAVDPKKG--------NIGIIAPIAIGFIVGANILAGGAFDGASMNPAVS 203(300)
HbTIP1;8        TFGLVYTVYATAVDPKKG--------DIGIIAPIAIGFIVGANILAGGAFDGASMNPAVS 203(300)
HbTIP2;1        TFALVYTVYATAADPKKG--------SLGIIAPIAIGFIVGANILAAGPFSGGSMNPARS 201(300)
HbTIP2;2        TFALVYTVYATAADPKKG--------SLGIIAPIAIGFIVGANILAAGPFSGGSMNPARS 201(300)
HbTIP2;3        TFALVYTVYATAADPKKG--------NLGIIAPIAIGFIVGANILAAGPFSGGSMNPARS 201(300)
HbTIP2;4        TFGPLYTVYATAAEPEKG--------NLGIMAPLAIGFVFGANILATSPFSGGSMNPASS 201(300)
HbTIP3;1        TFGLVYTVYATAVDPKRG--------SLGIIAPLAIGFIVGANILVGGPFDGASMNPARA 202(300)
HbTIP3;2        TFGLAYTVYATAVDTNRG--------SLGIIAPLAIGLIVGANILVGGPFDGASMNPARA 203(300)
HbTIP4;1        TFSLLFTVYGTIVDPKKG--------SIDGLGPLLTGLVVGANILAGGSFSGAAMNPARS 198(300)
HbTIP5;1        TFGLVYTVYAAG-DPRRS--------LQGVTGPLAIGLMAGANVLAAGPFSGGSMNPACA 199(300)
HbTIP5;2        TFGLVYTVYAAG-DPRCS--------LLGATGPLAIGLMAGANVLAAGPFSGGSMNPACA 201(300)
HbNIP1;1        TFYLMFIISGVATDNRAIGE----------LAGLAVGSTVLLNVLFAGPISGASMNPARS 229(300)
HbNIP1;2        TFYLMFIISGVATDNRAIGE----------LAGLAVGATVLLNVMFAGAISGASMNPARS 230(300)
HbNIP2;1        TFSMMFVTSAVATDTKAIGE----------LAGVAVGSAVCITSILAGPVSGGSMNPARS 216(300)
HbNIP3;1        TFNLMFNICGVATDHRGSKD----------LSGVAIGGTLLFNVLLAGPITGASMNPARS 223(300)
HbNIP4;1        SFLLMFVISGVATDNRAIGE----------LAGIAIGMTIMLNVFISGPVSGGSMNPVRS 211(300)
HbNIP4;2        TFLLMFVISGTTTDHRAVGE----------LGGIGVGMTILLNVFVAGPVSGASMNPARS 224(300)
HbNIP5;1        TFNLLFVVTAVATDTRAVGE----------LAGIAVGATVMLNILVAGPSSGGSMNPVRT 246(300)
HbNIP6;1        SFNLMFVVTAVATDTRAVGE----------LAGIAVGATVMLNILIAGQSTGASMNPVRT 250(300)
HbNIP7;1        TFIIMFLIASLTYQTS-VGH----------LSGFIVGLSIGLAVLITGPLSGGSLNPARS 236(300)
HbXIP1;1        TFVVLFVGVTVAFDKRRFKELGLVMVCVILAATMGLAIFVSITVTGRGGYAGVGLNPARC 247(300)
HbXIP1;2        SFLVLYVAITIAFNKKMCQELGFTMVCVIVAGVYALAVFASITVTGQAGYRGVGLNPARF 225(300)
HbXIP1;3        TFLVLYFSVTVAFDKKRCKQLGLTMFCVMVSGILAVAYFISLTITGQVGYGGPRLNPARC 247(300)
HbXIP1;4        TFLVLYFSISVAFDKERCKQLGLTMFCVIVSGIFAVAYFISLTITGQVGYGGARLNPARC 247(300)
HbXIP2;1        TFVFLFSSIWVAFDKRQAKPLGRVIVCSIIGLVVGLLVFISTTVTATRGYAGVGMNPARC 262(300)
HbXIP3;1        GFVFLFASVWMAFDHRQAKALGHVKIFMIVGIVLGLLVYVSTSVTTAKGYAGAGLNPARC 211(300)
HbSIP1;1        TFVFCLAMLLVMVKGPKN----------LLLKVWMVAVVTVGLVVSGRQYTGPSLNPANA 192(300)
HbSIP1;2        TFLISFAVLIIFLRGPCN----------SIVQNWLLAVVTVTLVVTGSKYTGPSMNPANA 191(300)
HbSIP1;3        TFLISFAVLVIILRGPRN----------SLVQNWLLAVVTVTLVVSGSKYTGPSMNPANA 191(300)
HbSIP2;1        TFAIVTISLGLSRKIPGS----------FFMKTWISSVSKLALHILGSDLTGGCMNPASV 187(300)
                 .                                                 *  :**.
```

```
                           HE                    TM6
SoPIP2_1        FGAAVIFNSNKVWDDQWIFWVGPFIGAAVAAAYHQYVLRAAAIKALGSFRSNPTN----- 281(360)
HbPIP1;1        LGAAIIFNKDKGWDDHWIFWVGPFIGAALAALYHQVVIRAIPFKK--------------- 287(360)
HbPIP1;2        LGAAIIFNKDKGWDDHWIFWVGPFIGAALAALYHQVVIRAIPFKK--------------- 287(360)
HbPIP1;3        LGAAIIFNKDHAWDDHWVFWVGPFIGAALAAVYHQIVIRAIPFKARA------------- 287(360)
HbPIP1;4        LGAAIIFNKDHAWDDHWIFWVGPFIGAALAAVYHQIVIRAIPFKARA------------- 287(360)
HbPIP1;5        LGAAIVYNEDCAWYDHWIFWVRPFIGAALAALSNQIVIRAIPFMSKA------------- 269(360)
HbPIP2;1        FGAAVIYNQDKAWDDQWIFWVGPFIGAAIAAFYHQYILRAAAVKALGSFRSTSNI----- 288(360)
HbPIP2;2        FGAAVIYNQDKAWDDQWIFWVGPLIGAAIAAFYHQYILRASAAKALGSFRSNSNI----- 288(360)
HbPIP2;3        FGAAVIYNKDKAWDDQWIFWVGPFIGAAIAAFYHQYILRAAAIKALGSFRSNA------- 286(360)
HbPIP2;4        FGAAVIYNKDKAWDDQWIFWVGPFIGAAIAALYHQYILRAAAIKALGSFRSNA------- 286(360)
HbPIP2;5        LGAAVIYNQDKPWDDHWIFWVGPFIGAAIAAFYHQFILRAGAVKALGSFRSNPTV----- 285(360)
HbPIP2;6        LGAAVIYNQDKAWDDQWIFWVGPFAGAAIAAFYHQFILRAGAVKALGSFRSNPSTV---- 286(360)
HbPIP2;7        FGAAVIYNNDKAWDDHWIFWVGPFVGALAAAAYHQYILRAAAIKALGSFRSNPTN----- 278(360)
HbPIP2;8        FGAAVIYNNDKIWDDHWIFWVGPFIGALAAAAYHQYILRAAAIKALGSFRSNPTN----- 280(360)
HbPIP2;9        FGAAVIYNNDKVWDDQWIFWVGPFVGALAAAIYHQHILRGTAIKALGSFTTNN------- 280(360)
HbPIP2;10       LGPAVIYNKKTIWDDHWIFWAGPFLGALAAALYHQYVLRAGAAKALGSFRSNRSI----- 285(360)
HbTIP1;1        FGPALVSWS---WDNHWVYWAGPLIGGGLAGVVYELLFIGHNTHEQLPSTDY-------- 252(360)
HbTIP1;2        FGPALVSWS---WDNHWVYWAGPLIGGGLAGLIYEFFFIGHNTHEQLPTTDY-------- 252(360)
HbTIP1;3        FGPALVSWD---WTNHWVYWVGPLIGGGLAGIIYNLFFITRTHEPVPSTSEF-------- 253(360)
HbTIP1;4        FGPALVSWD---WANHWVYGI----------------FIKKV------------------ 227(360)
HbTIP1;5        FGPAVVSWT---WTSHWVYWVGPLIGAAIAALVYDNIYIGENAHEPLSTSDF-------- 252(360)
HbTIP1;6        FGPAVVSWT---WTSHWVYWVGPLIGAAIAAIVYDNIFIGKNAHEPLSTNDF-------- 252(360)
HbTIP1;7        FGPAVVSWT---WDNHWVYWLGPFLGAGIAAVVYEVFFISPSTHEQLPSTEF-------- 252(360)
HbTIP1;8        FGPAVVSWT---WDNHWVYWLGPLLGAGIAAVVYEVFFISPSTHEQFPSADF-------- 252(360)
HbTIP2;1        FGPAVASGD---FHDNWIYWVGPLIGGGLAGLVYGNLYIPG-DHAPLSNEF--------- 248(360)
HbTIP2;2        FGPAVASGD---FHDNWVYWVGPLIGGGVAGLIYGNLYIPS-DHAPLSSEY--------- 248(360)
HbTIP2;3        FGPAVVSGD---FSENWIYWVGPLIGGGLAGLVYGQIFIGSYVPAPSSEDYA-------- 250(360)
HbTIP2;4        FGPAVVSGD---FSENCIYWVGPLIGGGLSGLQYGQVFIGSYSPVPSSEDYA-------- 250(360)
HbTIP3;1        FGPALVGWR---WRNHWIYWLGPFVGGGLAALIYEYTVIPTDPLPHHTHHQPLAPDDY-- 257(360)
HbTIP3;2        FGPALVGWR---WRNHWIYWLGPFIRGGLAALIYR---IISSAFGT-------------- 243(360)
HbTIP4;1        FGPALVSWD---WTHHWVYWVGPLIGGGLAGFIYENFFITRSHLPLPNDEETYLSTN--- 251(360)
HbTIP5;1        FGSAVIAGR---FKNQAVYWVGPLIGGTFAGLLYDNAVFPNQVPDSIRGISDGVGA---- 252(360)
HbTIP5;2        FGSAVIAGR---FKNQAVYWVGPLIGGTVAGLLYDNVVFPSQVPDSI-----GV------ 247(360)
HbNIP1;1        LGPAIVSSQ---YKGIWVYLISPILGAQAGAWAYNMIRYTDKPLREITKSASFLKSTGRA 286(360)
HbNIP1;2        LGPAIVSSQ---YKGLWIYIVSPILGAQAGAWVYNMIRYTDKPLREITMSASFLKNTGRA 287(360)
HbNIP2;1        LGPAIASAY---YKGIWVYIIGPVVGTLLGACSYNLIRVTDQPIQAISYSLKLRRIRSND 273(360)
HbNIP3;1        LGPAIVSGV---YKNLWVFIVSPILGALAAALIYSMLRVPNPEKPEEKNKIVLNYLYSPA 280(360)
HbNIP4;1        LGPAIVMHI---YTGIWIYIIGPVIGAILGGFCYNLIRFTDKPLREISKSSTLINSFRS- 267(360)
HbNIP4;2        IGPAIVKHV---YTGLWVYIVGPIVGAIAGAFAYNLLRTPEKPLDELANKGILKNSNSRN 281(360)
HbNIP5;1        LGPAVAAGN---YRDLWIYLVAPTLGALAGAGTYTLVKLREDEADP---PRPVRSFRR-- 298(360)
HbNIP6;1        LGPAIAANN---YKGIWIYLTAPILGALCGAGTYSAVKLPEEDADTREKPSEARSFRR-- 305(360)
HbNIP7;1        LGPAIISWN---FKDIWVYITAPVIGSLAGALMFHALRIQSRPCTSTDSSTNAGLLGHSI 293(360)
HbXIP1;1        LGPALLHGG-PLWHGHWVFWVGPFLACIIYYCYTLTFG---------------------- 284(360)
HbXIP1;2        LGPALLLGG-SLWDGHWVFWVGPFLACIVYYGFTLTLPKQGLVRAEKEHHITQLVLGSCY 284(360)
HbXIP1;3        IGPAVLVGG-SLWESLWVFWVGPFCASIVL------------------------------ 276(360)
HbXIP1;4        IGPAVLVGG-SLWQSLWVVWVGPFCASIVL------------------------------ 276(360)
HbXIP2;1        FGPAIIRGG-HLWNGHWVFWVGPIIASIAFAVYTKIVPSAEVHA---------------- 305(360)
HbXIP3;1        LGPAIVRGG-RLWDGHWVFWVGPAVSSVAFSLYTKLIPPQLSHTIF-------------- 256(360)
HbSIP1;1        YGWAYINNWHNTWELFYVYWICPFAGATLAAWVFRYLFKAPI-KDKQA------------ 239(360)
HbSIP1;2        FGWAYVNKWHDTWEQFYVYWICPFIGAILAAWVFRLVFPPPAPKQKEA------------ 239(360)
HbSIP1;3        FGWAYINKRHDTWEQFYVYWICPFVGAILAAWVFRLVFPPPAPKQKKA------------ 239(360)
HbSIP2;1        MGWAYARGDHITKEHILVYWLAPIEATLLAVWTFKLLVRPQKQEKEESKSKSD------- 240(360)
                 * *             :

SoPIP2_1        ------------
HbPIP1;1        ------------
HbPIP1;2        ------------
HbPIP1;3        ------------
HbPIP1;4        ------------
HbPIP1;5        ------------
```

```
HbPIP2;1        ------------
HbPIP2;2        ------------
HbPIP2;3        ------------
HbPIP2;4        ------------
HbPIP2;5        ------------
HbPIP2;6        ------------
HbPIP2;7        ------------
HbPIP2;8        ------------
HbPIP2;9        ------------
HbPIP2;10       ------------
HbTIP1;1        ------------
HbTIP1;2        ------------
HbTIP1;3        ------------
HbTIP1;4        ------------
HbTIP1;5        ------------
HbTIP1;6        ------------
HbTIP1;7        ------------
HbTIP1;8        ------------
HbTIP2;1        ------------
HbTIP2;2        ------------
HbTIP2;3        ------------
HbTIP2;4        ------------
HbTIP3;1        ------------
HbTIP3;2        ------------
HbTIP4;1        ------------
HbTIP5;1        ------------
HbTIP5;2        ------------
HbNIP1;1        ------------
HbNIP1;2        ------------
HbNIP2;1        EQAHNKDPFDAL 285(372)
HbNIP3;1        EP---------- 282(372)
HbNIP4;1        ------------
HbNIP4;2        ------------
HbNIP5;1        ------------
HbNIP6;1        ------------
HbNIP7;1        AIRRS------- 298(372)
HbXIP1;1        ------SVDED- 289(372)
HbXIP1;2        GAGFPSHVEEKV 296(372)
HbXIP1;3        ------------
HbXIP1;4        ------------
HbXIP2;1        ------------
HbXIP3;1        ------------
HbSIP1;1        ------------
HbSIP1;2        ------------
HbSIP1;3        ------------
HbSIP2;1        ------------
```

Additional file 5: Alignment of predicted amino acid sequences of rubber tree aquaporins with structure determined Spinach SoPIP2;1.

Multiple alignments were performed using ClustalW2. Trans-membrane helices (TM1–TM6) and the two short helices forming the two NPAs (HB and HE) (shaded), P_1–P_5 residues (shown in blue), NPA motifs (shown in red), and ar/R selectivity filter residues (shown in green) are indicated. The highly conserved cysteine residues in XIPs are shown in **bold**. The positions corresponding to S115 and S274 in SoPIP2;1 (GenBank accession number 1Z98) are highlighted in bright green. The residues corresponding to S262 in GmNOD26 (GenBank accession number P08995) are shown in sky blue. The residues at the position corresponding to L197 from SoPIP2;1, determined to be the key residue involved in gating (occurring just before TM5) are underlined. The numbers at the end of each line

indicate amino acid position excluding the introduced gaps (number without parentheses) or including the introduced gaps (number in parentheses).
