# Supplementary material for: Genome-wide identification of rubber tree (Hevea brasiliensis Muell. Arg.) aquaporin genes and their response to ethephon stimulation in the laticifer, a rubber-producing tissue
Source: BMC Genomics. 2015 Nov 25;16:1001. doi: 10.1186/s12864-015-2152-6 (PMC4658816; doi:10.1186/s12864-015-2152-6)
Supplement: Additional file 6: — SDP analysis of the HbAQPs based on the sequence alignment with AQPs transporting non-aqua substrates. (PDF 165 kb) [file 12864_2015_2152_MOESM6_ESM.pdf]

Additional file 6: SDP analysis of rubber tree aquaporins from alignments with putative amino acid sequences of AQPs transporting non-aqua substrates.

Ammonia (NH$_3$) Transporters

```
HbTIP2;2        VFYWIAQLLGSIVACFLLKFVTGGLAIPTHSVAAGVGAIEGVVMEIVITFALVYTVYATA
TaTIP2;1        IFYWVAQLLGAIVGAFLVQFCT-GVATPTHGLS-GVGAFEGVVMEIIVTFGLVYTVYATA
TaTIP2;2        IFYWVAQLLGAIVGAFLVQFCT-GVATPTHGLS-GVGAFEGVVMEIIVTFGLVYTVYATA
AtTIP2;1        VFYWIAQLLGSTAACFLLKYVTGGLAVPTHSVAAGLGSIEGVVMEIIITFALVYTVYATA
AtTIP2;3        FFYWIAQCLGSIVACLLLVFVTNGKSVPTHGVSAGLGAVEGVVMEIVVTFALVYTVYATA
GmNOD26         PAYVVAQLLGSILASGTLRLLFMGNHDQFSGTVPNGTNLQAFVFEFIMTFFLMFVICGVA
                  * :** **:  ..  :     *      .   .   .:..*:*:::** *::.: ..*

HbTIP2;2        ADPKKGSLGIIAPIAIGFIVGANILAAGPFSGGSMNPARSFGPAVASGDFHDNWVYWVGP
TaTIP2;1        ADPKKGSLGTIAPIAIGFIVGANILVAGPFSGGSMNPARSFGPAVASGDFTNIWVYWAGP
TaTIP2;2        ADPKKGSLGTIAPIAIGFIVGANILVAGPFSGGSMNPARSFGPAVASGDFTNIWIYWAGP
AtTIP2;1        ADPKKGSLGTIAPLAIGLIVGANILAAGPFSGGSMNPARSFGPAVAAGDFSGHWVYWVGP
AtTIP2;3        ADPKKGSLGTIAPIAIGFIVGANILAAGPFSGGSMNPARSFGPAVVSGDLSQIWIYWVGP
GmNOD26         TDNR--AVGEFAGIAIGSTLLLNVIIGGPVTGASMNPARSLGPAFVHGEYEGIWIYLLAP
                :* :  ::* :* :***  :  *:: .**.:*.*******:***.. *:    *:*  .*

HbTIP2;2        LIGGGVAGLIYGNLYIPS-DHAPLSSEY----------
TaTIP2;1        LIGGGLAGVVYRYLYMCD-DHSSVAGNDY----------
TaTIP2;2        LIGGGLAGVVYRYVYMCD-DHSSVAGNDY----------
AtTIP2;1        LIGGGLAGLIYGNVFMGSSEHVPLASADF---------
AtTIP2;3        LVGGALAGLIYGDVFIGSYEAVETREIRV---------
GmNOD26         VVGAIAGAWVYNIVRYTDKPLSETTKSASFLKGRAASK
                ::*.  .. :*  :           .
```

Boric Acid Transporters

```
HbPIP1;1        STVGIQGIAWSFGGMIFALVYCTAGISGGHINPAVTFGLFLARKLSLTRALYYMVMQCLG
HbPIP1;2        STVGIQGIAWAFGGMIFALVYCTAGISGGHINPAVTFGLFLARKLSLTRALYYMVMQCLG
HbPIP1;3        ATVGTQGIAWAFGGMIFALVYCTAGISGGHINPAVTFGLFLARKLSLTRALFYIIMQCLG
HbPIP1;4        TTVGTQGIAWAFGGMIFALVYCTAGISGGHINPAVTFGLFLARKLSLTRALFYMIMQCLG
HbPIP1;5        ASVGVQGIAWAFGGMIFVLVYCTAGISGGHINPAVTFGLTLARKVSLTRAIFYMVMQCLG
HbNIP2;1        RRISKLGASVAGGLIVTVMIYAVGHVSGAHMNPAVTTAFAAFRHFPWKQVPFYAAAQLTG
HbNIP5;1        GVESLIGNAACAGLAVMIIILSTGHISGAHLNPSLTIAFAALRHFPWMQVPAYIAAQVSA
HbNIP6;1        GTETLIGLAASTGLAVMIVILSTGHISGAHLNPSVTIAFAALKHFPWKHVPVYIGAQVMA
HbXIP1;3        VAEAKLLVPVVVFSTIFLLLVVTIPVSGVHMNPTFTFIFALKGVITFVRALVYILAQCLG
HbXIP1;4        VAEPKLLVPVVVFSTIFLLLVLTIPVSGGHMNPTFTFIFALKGAITFVRALVYILAQCLG
HbXIP2;1        TKTPNLIMSALIAITVTILLNATFPISGGHINPVITLSAAFTGLVSLSRAAIYILAQCLG
HvPIP1;3        GTVGIQGIAWSFGGMIFVLVYCTAGISGGHINPAVTFGLFLARKLSLTRAVFYIVMQCLG
HvPIP1;4        GTVGIQGIAWSFGGMIFVLVYCTAGISGGHINPAVTFGLFLARKLSLTRAVFYIVMQCLG
ZmPIP1;1        ATVGIQGIAWSFGGMILALVYCTAGISG-HINPAVTFGLFLARKLSLTRAVFYIIMQCLG
AtNIP5;1        GAETLIGNAACAGLAVMIIILSTGHISGAHLNPSLTIAFAALRHFPWAHVPAYIAAQVSA
AtNIP6;1        GAETLIGCAASAGLAVMIVILSTGHISGAHLNPAVTIAFAALKHFPWKHVPVYIGAQVMA
OsNIP2;1        SRISQLGQSIAGGLIVTVMIYAVGHISGAHMNPAVTLAFAVFRHFPWIQVPFYWAAQFTG
NtXIP1;1α       VKMPNLIMSILIAIVITILLLAVVPVSGGHINPVISFSAALVGIISMSRAIIYMVAQCVG
NtXIP1;1β       VKMPNLIMSILIAIVITILLLAVVPVSGGHINPVISFSAALVGIISMSRAIIYMVAQCVG
StXIP1;1α       TKMPNLIMSILIAVVITILLLAVVPVSGGHINPVISFSAALVGIISMSRAIIYIVAQCLG
StXIP1;1β       TKMPNLIMSILIAVVITILLLAVVPVSGGHINPVISFSAALVGIISMSRAIIYIVAQCLG
SlXIP1;1α       TKMPNLIMSILIAVVITILLLAVVPVSGGHINPVISFSAALVGIISMSRAIIYIVAQCVG
SlXIP1;1β       TKMPNLIMSILIAVVITILLLAVVPVSGGHINPVISFSAALVGIISMSRAIIYIVAQCVG
                        .      :  ::  .  :** *:** .:        ..  :.  *   *  .
```

```
HbPIP1;1       AICGAGVVKGFEGRHQYTLL------------GGGANSVNPGYTKGDGLGAEIVGTFVLV
HbPIP1;2       AICGAGVVKGFEGRHQYTLL------------GGGANSVNPGYTKGDGLGAEIVGTFVLV
HbPIP1;3       AICGAGVVKGFEGDRVYETL------------GGGANVVAHGYTKGDGLGAEIVGTFVLV
HbPIP1;4       AICGAGVVKGFEGNRAYETL------------GGGANVVAHGYTKGDGLGAEIVGTFVLV
HbPIP1;5       AIFGAGIVKGFQPT-PFETL------------GGGANVVNPGYSKGDGLGAEIVETFALV
HbNIP2;1       AISASFTLRVLLHPIKQVGTTS------------------PSGSDLQALIMEIVVTFSMM
HbNIP5;1       SICASFALKGVFHPFMSGGVTV------------------PSVSTGQAFALEFLITFNLL
HbNIP6;1       SVSAAFALKGIFHPIMGGGVTV------------------PSGGYGQAFALEFIISFNLM
HbXIP1;3       STMAYLIVKRAMNPKIAEKYSLGGCSM-----GGNG----EGISAGTALAIEFACTFLVL
HbXIP1;4       STMANLIVKRAMNPKIAEKYSLGGSCSV-----GGNG----EGISAGTALAIEFACTFLVL
HbXIP2;1       GILGALALKAVVNSTIEKTFSLGGCTLSIVAPGPHG-PIVIGLGTAQALWLEIICTFVFL
HvPIP1;3       AICGAGVVKGFQTT-LYQGN------------GGGANSVAAGYTKGDGLGAEIVGTFVLV
HvPIP1;4       AICGAGVVKGFQTT-LYQGN------------GGGANSVAAGYTKGDGLGAEIVGTFVLV
ZmPIP1;1       AICGRGVVKGFQQG-LYMGN------------GGRRNVVAPGYTKGDGLGAEIVGTFILV
AtNIP5;1       SICASFALKGVFHPFMSGGVTI------------------PSVSLGQAFALEFIITFILL
AtNIP6;1       SVSAAFALKAVFEPTMSGGVTV------------------PTVGLSQAFALEFIISFNLM
OsNIP2;1       AICASFVLKAVIHPVDVIGTTT------------------PVGPHWHSLVVEVIVTFNMM
NtXIP1;1α      AILGALALKAVVSSTIAQTFSLGGCTITVIAPGPNG-PITVGLEMAQALWLEIFCTFVFL
NtXIP1;1β      AILGALALKAVVSSTIAQTFSLGGCTITVIAPGPNG-PITVGLEMAQALWLEIFCTFVFL
StXIP1;1α      AVLGALALRAVVSSSIEDTFSLGGCTVTIIAPGPNG-PVTVGLETAQALWLEIFCTFVFL
StXIP1;1β      AVLGALALRAVVSSSIEDTFSLGGCTVTIIAPGPNG-PVTVGLETAQALWLEIFCTFVFL
SlXIP1;1α      AILGALALRAVVSSSIEDTFSLGGCTVTIIAPGPNG-PVIVGLETAQALWLEIFCTFVFL
SlXIP1;1β      AILGALALRAVVSSSIEDTFSLGGCTVTIIAPGPNG-PVIVGLETAQALWLEIFCTFVFL
               .  .   ::                                      .:  *.   * .:
```

```
HbPIP1;1       YTVFSATDAKRNARDSHVPILAPLP---IGFAVFLVHLATIP--ITGTGINPARSLGAAI
HbPIP1;2       YTVFSATDAKRNARDSHVPILAPLP---IGFAVFLVHLATIP--ITGTGINPARSLGAAI
HbPIP1;3       YTVFSATDAKRNARDSHVPILAPLP---IGFAVFLVHLATIP--ITGTGINPARSLGAAI
HbPIP1;4       YTVFSATDAKRNARDSHVPILAPLP---IGFAVFLVHLATIP--ITGTGINPARSLGAAI
HbPIP1;5       YTVLSATDAKRSARDSHVPILASLP---IGFAVFLVHLATIP--ITGTGINPARSLGAAI
HbNIP2;1       FVTSAVATDTK--------AIGELAGVAVGSAVCITSILAGP--VSGGSMNPARSLGPAI
HbNIP5;1       FVVTAVATDTR--------AVGELAGIAVGATVMLNILVAGP--SSGGSMNPVRTLGPAV
HbNIP6;1       FVVTAVATDTR--------AVGELAGIAVGATVMLNILIAGQ--STGASMNPVRTLGPAI
HbXIP1;3       YFSVTVAFDKKRCKQLGLTMFCVMVSGILAVAYFISLTITGQVGYGGPRLNPARCIGPAV
HbXIP1;4       YFSISVAFDKERCKQLGLTMFCVIVSGIFAVAYFISLTITGQVGYGGARLNPARCIGPAV
HbXIP2;1       FSSIWVAFDKRQAKPLGRVIVCSIIGLVVGLLVFISTTVTATRGYAGVGMNPARCFGPAI
HvPIP1;3       YTVFSATDAKRSARDSHVPILAPLP---IGFAVFLVHLATIP--ITGTGINPARSLGAAI
HvPIP1;4       YTVFSATDAKRSARDSHVPILAPLP---IGFAVFLVHLATIP--ITGTGINPARSLGAAI
ZmPIP1;1       YTVFSATDAKRRARDSHVPILAPLP---IGFAVFLVHLATMG--ITGTGINPARSLGAAV
AtNIP5;1       FVVTAVATDTR--------AVGELAGIAVGATVMLNILVAGP--STGGSMNPVRTLGPAV
AtNIP6;1       FVVTAVATDTR--------AVGELAGIAVGATVMLNILIAGP--ATSASMNPVRTLGPAI
OsNIP2;1       FVTLAVATDTR--------AVGELAGLAVGSAVCITSIFAGA--ISGGSMNPARTLGPAL
NtXIP1;1α      FASIWMAYDHRQAKALGLVTVLSIVGIVLGLLVFISTTVTMKKGYAGAGMNPARCFGAAV
NtXIP1;1β      FASIWMAYDHRQAKALGLVTVLSIVGIVLGLLVFISTTVTMKKGYAGAGMNPARCFGAAV
StXIP1;1α      FASIWMAYDHRQAKALGHVTVLSIVGLVLGLLVFISTTVTAKKGYGGAGINPARCLGPAI
StXIP1;1β      FASIWMAYDHRQAKALGHVTVLSIVGLVLGLLVFISTTVTAKKGYGGAGINPARCLGPAI
SlXIP1;1α      FASIWMAYDHRQAKALGHVTVLSIVGLVLGLLVFISTTVTAKKGYGGAGINPARCLGPAI
SlXIP1;1β      FASIWMAYDHRQAKALGHVTVLSIVGLVLGLLVFISTTVTAKKGYGGAGINPARCLGPAI
               :     :   .         .  :    ..    :    :      .  :**.* :*.*:
```

Carbon dioxide (CO_2) Transporters

```
HbPIP1;3       HINPAVTFGLFLARKLSLTRALFYIIMQCLGAICGAGVVKGFEGDRVYETLGGGANVVAH
AtPIP1;2       HINPAVTFGLFLARKLSLTRAVYYIVMQCLGAICGAGVVKGFQ-PKQYQALGGGANTIAH
NtAQP1         HINPAVTFGLFLARKLSLTRAIFYIVMQCLGAICGAGVVKGFM-VGPYQRLGGGANVVNH
HvPIP2;1       HINPAVTFGLFLARKVSLIRALLYIIAQCLGAICGVGLVKGFQ-SSYYVRYGGGANELSA
               ***************:** **: **: ********.*:****     *   ***** :
```

```
HbPIP1;3      GYTKGDGLGAEIVGTFVLVYTVFSATDAKRNARDSHVPILAPLPIGFAVFLVHLATIPIT
AtPIP1;2      GYTKGSGLGAEIIGTFVLVYTVFSATDAKRNARDSHVPILAPLPIGFAVFLVHLATIPIT
NtAQP1        GYTKGDGLGAEIIGTFVLVYTVFSATDAKRNARDSYVPILAPLPIGFAVFLVHLATIPIT
HvPIP2;1      GYSKGTGLAAEIIGTFVLVYTVFSATDPKRNARDSHIPVLAPLPIGFAVFMVHLATIPIT
              **:** **.***:**************.*******::*:***********:*********

HbPIP1;3      GTGINPARSLGAAIIFNKDHAWDDHWVFWVGPFIGAALAAVYHQIVIRAIPFKARA----
AtPIP1;2      GTGINPARSLGAAIIFNKDNAWDDHWVFWVGPFIGAALAALYHVIVIRAIPFKSRS----
NtAQP1        GTGINPARSLGAAIIYNTDQAWDDHWIFWVGPFIGAALAAVYHQIIIRAIPFHKSS----
HvPIP2;1      GTGINPARSLGAAVIYNTDKAWDDQWIFWVGPLIGAAIAAAYHQYVLRASAAKLGSYRSN
              ************:*:*.*:****:*:*****:****:**  **  ::** . :  :
```

H_2O_2 Transporters

```
HbPIP1;1      AICGAGVVKGFEGR-----HQYTLLGGGANSVNPGY--------TKGDGLGAEIVGTFVL
HbPIP1;2      AICGAGVVKGFEGR-----HQYTLLGGGANSVNPGY--------TKGDGLGAEIVGTFVL
HbPIP1;3      AICGAGVVKGFEGD-----RVYETLGGGANVVAHGY--------TKGDGLGAEIVGTFVL
HbPIP1;4      AICGAGVVKGFEGN-----RAYETLGGGANVVAHGY--------TKGDGLGAEIVGTFVL
HbPIP1;5      AIFGAGIVKGFQP------TPFETLGGGANVVNPGY--------SKGDGLGAEIVETFAL
HbPIP2;1      AICGCGLVKAFQK------AYYNRYGGGANELADGY--------SKGTGLGAEIIGTFVL
HbPIP2;2      AICGCGLVKAFQK------AYYNRYGGGANELADGY--------SKGTGLGAEIIGTFVL
HbPIP2;3      AICGCGLVKAFQK------AYYTRYGGGANELSSGY--------SKGTGLGAEIIGTFVL
HbPIP2;4      AICGCGLVKAFQK------AYYNRYGGGANELSDGY--------NKGTGLGAEIIGTFVL
HbPIP2;5      AIAGVGLVKAFQS------SFYKRYGGGANSLAAGY--------SKGVGLGAEIIGTFVL
HbPIP2;6      AIAGVGLVKAFQS------SHYKRYGGGANSLANGY--------STGVGLGAEIIGTFVL
HbPIP2;7      AICGVGLVKAFMK------HPYNGLGGGANTVAPGY--------NKGTALGAEIIGTFVL
HbPIP2;8      AICGVGLVKAFMK------HPYNALGGGANSVAHGY--------NKGTALGAEIIGTFVL
HbPIP2;10     AIAGAGLVKAVMK------DDYKSLGGGVNSVSSGY--------SKGTALGAEIIGTFVL
HbTIP1;5      SVVACLLLKFATGG----------LETSAFALSSGV--------SSWNALVFEIVMTFGL
HbTIP1;6      SVVACLLLKFATGG----------LETSAFALSSGV--------SSWNAVVFEIVMTFGL
HbTIP4;1      SSAACLLLSYLTGG----------MATPVITLASGV--------GYVQGVVWEILLTFSL
HbTIP5;1      SVMACLLLRVVIVG----------QSLPTYTIAEEM--------TGFGASVIEGVLTFGL
HbTIP5;2      SVMACLLLRVAIVG----------QSLPTYTIAAEM--------TGFGASVFEGVLTFGL
HbNIP3;1      ATLACLTLKVLFHD-------QDDIQATMTQYKDST--------SDLEAFIWEFIITFNL
HbNIP4;2      SILASGTLALVF----DVTP---NAYFG---TVPVG--------SNVQPLVIEIIITFLL
HbNIP5;1      SICASFALKGVF-----------HPFMSGGVTVPSV--------STGQAFALEFLITFNL
HbXIP1;3      STMAYLIVKRAMNP--KIAEKYSLGGCSMGGNGEG--------ISAGTALAIEFACTFLV
HbXIP1;4      STMANLIVKRAMNP--KIAEKYSLGSCSVGGNGEG--------ISAGTALAIEFACTFLV
ZmPIP2;5      AICGVGLVKGFQS------AFYVRYGGGANELSAGY--------SKGTGLAAEIIGTFVL
AtPIP2;1      AICGVGFVKAFQS------SYYTRYGGGANSLADGY--------STGTGLAAEIIGTFVL
AtPIP2;2      AICGVGFVKAFQS------SYYDRYGGGANSLADGY--------NTGTGLAAEIIGTFVL
AtPIP2;4      AICGCGFVKAFQS------SYYTRYGGGANELADGY--------NKGTGLGAEIIGTFVL
AtPIP2;5      AICGVALVKAFQS------AYFTRYGGGANGLSDGY--------SIGTGVAAEIIGTFVL
AtPIP2;7      AICGVGFVKAFMK------TPYNTLGGGANTVADGY--------SKGTALGAEIIGTFVL
AtTIP1;1      SVVACLILKFATGG----------LAVPAFGLSAGV--------GVLNAFVFEIVMTFGL
AtTIP1;2      SVAACFLLSFATGG----------EPIPAFGLSAGV--------GSLNALVFEIVMTFGL
AtTIP2;3      SIVACLLLVFVTNG----------KSVPTHGVSAGL--------GAVEGVVMEIVVTFAL
AtNIP1;2      STLAAATLRLLFGLDQDVCSGKHDVFVG---TLPSG--------SNLQSFVIEFIITFYL
TgTIP1;1      SSVACLLLRFTTG-----------LGTGTFGLVAGV--------SVWSGLVMEIVMTFGL
TgTIP1;2      SSVACLLLRFTTG-----------LGTGTFGLVAGV--------SVWSGLVMEIVMTFGL
NtXIP1;1α     AILGALALKAVVSS--TIAQTFSLGGCTITVIAPGNGPITVGLEMAQALWLEIFCTFVF
NtXIP1;1β     AILGALALKAVVSS--TIAQTFSLGGCTITVIAPGNGPITVGLEMAQALWLEIFCTFVF
StXIP1;1α     AVLGALALRAVVSS--SIEDTFSLGGCTVTIIAPGNGPVTVGLETAQALWLEIFCTFVF
StXIP1;1β     AVLGALALRAVVSS--SIEDTFSLGGCTVTIIAPGNGPVTVGLETAQALWLEIFCTFVF
SlXIP1;1α     AILGALALRAVVSS--SIEDTFSLGGCTVTIIAPGNGPVIVGLETAQALWLEIFCTFVF
SlXIP1;1β     AILGALALRAVVSS--SIEDTFSLGGCTVTIIAPGNGPVIVGLETAQALWLEIFCTFVF
              :  .   :                                            *   ** .
```

```
HbPIP1;1      VYTVFSATDAKRNARDS---HVPILAPLPIGFAVFLVHLATIPI—TGTGINPARSLGAA
HbPIP1;2      VYTVFSATDAKRNARDS---HVPILAPLPIGFAVFLVHLATIPI--TGTGINPARSLGAA
HbPIP1;3      VYTVFSATDAKRNARDS---HVPILAPLPIGFAVFLVHLATIPI--TGTGINPARSLGAA
HbPIP1;4      VYTVFSATDAKRNARDS---HVPILAPLPIGFAVFLVHLATIPI--TGTGINPARSLGAA
HbPIP1;5      VYTVLSATDAKRSARDS---HVPILASLPIGFAVFLVHLATIPI--TGTGINPARSLGAA
HbPIP2;1      VYTVFSATDPKRNARDS---HVPVLAPLPIGFAVFMVHLATIPV--TGTGINPARSFGAA
HbPIP2;2      VYTVFSATDPKRNARDS---HVPVLAPLPIGFAVFMVHLATIPV--TGTGINPARSFGAA
HbPIP2;3      VYTVFSATDPKRNARDS---HVPVLAPLPIGFAVFMVHLATIPI--TGTGINPARSFGAA
HbPIP2;4      VYTVFSATDPKRNARDS---HVPVLAPLPIGFAVFMVHLATIPI--TGTGINPARSFGAA
HbPIP2;5      VYTVFSATDPKRNARDS---HVPVLAPLPIGFAVFMVHLATIPI--TGTGINPARSLGAA
HbPIP2;6      VYTVFSATDPKRSARDS---HVPVLAPLPIGFAVFMVHLATIPI--TGTGINPARSLGAA
HbPIP2;7      VYTVFSATDPKRSARDS---HVPVLAPLPIGFAVFMVHLATIPI--TGTGINPARSFGAA
HbPIP2;8      VYTVFSATDPKRSARDS---HVPVLAPLPIGFAVFVVHLATIPV--TGTGINPARSFGAA
HbPIP2;10     VYTVFSATDPKRKARDS---FVPILVPLPIGFAVFVVHLATIPI--TGTGINPARSLGPA
HbTIP1;5      VYTVYATAVDPKKG------NVGIVAPIAIGFIVGANILAGGAF--DGASMNPAVSFGPA
HbTIP1;6      VYTVYATAVDPKKG------NVGTVAPIAIGFIVGANILAGGAF--DGASMNPAVSFGPA
HbTIP4;1      LFTVYGTIVDPKKG------SIDGLGPLLTGLVVGANILAGGSF--SGAAMNPARSFGPA
HbTIP5;1      VYTVYA-AGDPRRS------LQGVTGPLAIGLMAGANVLAAGPF--SGGSMNPACAFGSA
HbTIP5;2      VYTVYA-AGDPRCS------LLGATGPLAIGLMAGANVLAAGPF--SGGSMNPACAFGSA
HbNIP3;1      MFNICGVATDHR--------GSKDLSGVAIGGTLLFNVLLAGPI--TGASMNPARSLGPA
HbNIP4;2      MFVISGTTTDHR--------AVGELGGIGVGMTILLNVFVAGPV--SGASMNPARSIGPA
HbNIP5;1      LFVVTAVATDTR--------AVGELAGIAVGATVMLNILVAGPS--SGGSMNPVRTLGPA
HbXIP1;3      LYFSVTVAFDKKRCKQLGLTMFCVMVSGILAVAYFISLTITGQVGYGGPRLNPARCIGPA
HbXIP1;4      LYFSISVAFDKERCKQLGLTMFCVIVSGIFAVAYFISLTITGQVGYGGARLNPARCIGPA
ZmPIP2;5      VYTVFSATDPKRNARDS---HVPVLAPLPIGFAVFMVHLATIPI--TGTGINPARSLGAA
AtPIP2;1      VYTVFSATDPKRSARDS---HVPVLAPLPIGFAVFMVHLATIPI--TGTGINPARSFGAA
AtPIP2;2      VYTVFSATDPKRNARDS---HVPVLAPLPIGFAVFMVHLATIPI--TGTGINPARSFGAA
AtPIP2;4      VYTVFSATDPKRNARDS---HVPVLAPLPIGFAVFMVHLATIPI--TGTGINPARSFGAA
AtPIP2;5      VYTVFSATDPKRSARDS---HVPVLAPLPIGFAVFIVHLATIPI--TGTGINPARSLGAA
AtPIP2;7      VYTVFSATDPKRSARDS---HIPVLAPLPIGFAVFMVHLATIPI--TGTGINPARSFGAA
AtTIP1;1      VYTVYATAIDPKNG------SLGTIAPIAIGFIVGANILAGGAF--SGASMNPAVAFGPA
AtTIP1;2      VYTVYATAVDPKNG------SLGTIAPIAIGFIVGANILAGGAF--SGASMNPAVAFGPA
AtTIP2;3      VYTVYATAADPKKG------SLGTIAPIAIGFIVGANILAAGPF--SGGSMNPARSFGPA
AtNIP1;2      MFVISGVATDNR--------AIGELAGLAVGSTVLLNVIIAGPV--SGASMNPGRSLGPA
TgTIP1;1      VYTVYATAVDPKKG------DIGTIAPIAIGFIVGANILVGGAF--TGASMNPAIAFGPA
TgTIP1;2      VYTVYATAVDPKKG------DIGTIAPIAIGFIVGANILVGGAF--TGASMNPAIAFGPA
NtXIP1;1α     LFASIWMAYDHRQAKALGLVTVLSIVGIVLGLLVFISTTVTMKKGYAGAGMNPARCFGAA
NtXIP1;1β     LFASIWMAYDHRQAKALGLVTVLSIVGIVLGLLVFISTTVTMKKGYAGAGMNPARCFGAA
StXIP1;1α     LFASIWMAYDHRQAKALGHVTVLSIVGLVLGLLVFISTTVTAKKGYGGAGINPARCLGPA
StXIP1;1β     LFASIWMAYDHRQAKALGHVTVLSIVGLVLGLLVFISTTVTAKKGYGGAGINPARCLGPA
SlXIP1;1α     LFASIWMAYDHRQAKALGHVTVLSIVGLVLGLLVFISTTVTAKKGYGGAGINPARCLGPA
SlXIP1;1β     LFASIWMAYDHRQAKALGHVTVLSIVGLVLGLLVFISTTVTAKKGYGGAGINPARCLGPA
              ::         .                  .                *  :**   :*.*
```

```
HbPIP1;1      IIFNKDKGWDDHWIFWVGPFIGAALAALYHQVVIRAIPFKK-------------------
HbPIP1;2      IIFNKDKGWDDHWIFWVGPFIGAALAALYHQVVIRAIPFKK-------------------
HbPIP1;3      IIFNKDHAWDDHWVFWVGPFIGAALAAVYHQIVIRAIPFKARA-----------------
HbPIP1;4      IIFNKDHAWDDHWIFWVGPFIGAALAAVYHQIVIRAIPFKARA-----------------
HbPIP1;5      IVYNEDCAWYDHWIFWVRPFIGAALAALSNQIVIRAIPFMSKA-----------------
HbPIP2;1      VIYNQDKAWDDQWIFWVGPFIGAAIAAFYHQYILRAAAVKALGSFRSTSNI---------
HbPIP2;2      VIYNQDKAWDDQWIFWVGPLIGAAIAAFYHQYILRASAAKALGSFRSNSNI---------
HbPIP2;3      VIYNKDKAWDDQWIFWVGPFIGAAIAAFYHQYILRAAAIKALGSFRSNA-----------
HbPIP2;4      VIYNKDKAWDDQWIFWVGPFIGAAIAALYHQYILRAAAIKALGSFRSNA-----------
HbPIP2;5      VIYNQDKPWDDHWIFWVGPFIGAAIAAFYHQFILRAGAVKALGSFRSNPTV---------
HbPIP2;6      VIYNQDKAWDDQWIFWVGPFAGAAIAAFYHQFILRAGAVKALGSFRSNPSTV--------
HbPIP2;7      VIYNNDKAWDDHWIFWVGPFVGALAAAAYHQYILRAAAIKALGSFRSNPTN---------
HbPIP2;8      VIYNNDKIWDDHWIFWVGPFIGALAAAAYHQYILRAAAIKALGSFRSNPTN---------
HbPIP2;10     VIYNKKTIWDDHWIFWAGPFLGALAAALYHQYVLRAGAAKALGSFRSNRSI---------
HbTIP1;5      VVSWT---WTSHWVYWVGPLIGAAIAALVYDNIYIGENAHEPLSTSDF------------
HbTIP1;6      VVSWT---WTSHWVYWVGPLIGAAIAAIVYDNIFIGKNAHEPLSTNDF------------
HbTIP4;1      LVSWD---WTHHWVYWVGPLIGGGLAGFIYENFFITR-SHLPLPNDEETYLSTN------
HbTIP5;1      VIAGR---FKNQAVYWVGPLIGGTFAGLLYDNAVFPNQVPDSIRGISDGVGA--------
HbTIP5;2      VIAGR---FKNQAVYWVGPLIGGTVAGLLYDNVVFPSQVPDSI-----GV----------
HbNIP3;1      IVSGV---YKNLWVFIVSPILGALAAALIYSMLRVPNPEKPEEKNKIVLNYLYSPAEP--
HbNIP4;2      IVKHV---YTGLWVYIVGPIVGAIAGAFAYNLLRTPEKPLDELANKG----ILKNSNSRN
HbNIP5;1      VAAGN---YRDLWIYLVAPTLGALAGAGTYTLVKLRE---DEADPPR----PVRSFRR--
HbXIP1;3      VLVGG-SLWESLWVFWVGPFCASIVL----------------------------------
HbXIP1;4      VLVGG-SLWQSLWVVWVGPFCASIVL----------------------------------
ZmPIP2;5      VIYNNDKAWDDHWIFWVGPFIGAAIAAAYHQYVLRASAAK-LGSSASFSR----------
AtPIP2;1      VIYNKSKPWDDHWIFWVGPFIGAAIAAFYHQFVLRASGSKSLGSFRSAANV---------
AtPIP2;2      VIYNKSKPWDDHWIFWVGPFIGAAIAAFYHQFVLRASGSKSLGSFRSAANV---------
AtPIP2;4      VIYNNEKAWDDQWIFWVGPMIGAAAAAFYHQFILRAAAIKALGSFGSFGSFRSFA-----
AtPIP2;5      IIYNKDKAWDHHWIFWVGPFAGAAIAAFYHQFVLRAGAIKALGSFRSQPHV---------
AtPIP2;7      VIYNNEKAWDDQWIFWVGPFLGALAAAAYHQYILRASAIKALGSFRSNATN---------
AtTIP1;1      VVSWT---WTNHWVYWAGPLVGGGIAGLIYEVFFINT-THEQLPTTDY------------
AtTIP1;2      VVSWT---WTNHWVYWAGPLIGGGLAGIIYDFVFIDENAHEQLPTTDY------------
AtTIP2;3      VVSGD---LSQIWIYWVGPLVGGALAGLIYGDVFIGS--YEAVETREIRV----------
AtNIP1;2      MVYSC---YRGLWIYIVSPIVGAVSGAWVYNMVRYTDKPLREITKSGSFLKTVRNGSSR-
TgTIP1;1      LVSWS---WTSHWVYWVGPLIGGGIAGIVYDLFFINS-THEQLPSTDY------------
TgTIP1;2      LVSWS---WASHWVYWVGPLIGGGIAAIVYDLFFISG-THEQLPTTDY------------
NtXIP1;1α     VVRGG-HLWDGHWIFWVGPTIACVAFYVYTKIIPPKHFHAD-GYKYDFIGVVKASFGLHE
NtXIP1;1β     VVRGG-HLWDGHWIFWVGPTIACVAFYVYTKIIPPKHFHAD-GYKYDFIGVVKASFGLHE
StXIP1;1α     IIRGG-HLWDGHWIFWVGPTIGCVAFYVYTKIIPPKHFLAEYGFKHDFVGVVKALSNV--
StXIP1;1β     IIRGG-HLWDGHWIFWVGPTIGCVAFYVYTKIIPPKHFLAEYGFKHDFVGVVKALSNV--
SlXIP1;1α     IIRGG-HLWDGHWIFWVGPTIGCVAFYVYTKIIPTKHFLAEYGFKHDFVGVVKALSNV--
SlXIP1;1β     IIRGG-HLWDGHWIFWVGPTIGCVAFYVYTKIIPTKHFLAEYGFKHDFVGVVKALSNV--
              :            :  . *  .
```

Silicic Acid Transporters

```
HbNIP2;1      FRHFPWKQVPFYAAAQLTGAISASFTLRVLLHPIKQVGTTSPSGSDLQALIMEIVVTFSM
HvNIP2;1      FRHFPWIQVPFYWAAQFTGAICASFVLKAVLHPITVIGTTEPVGPHWHALVIEVVVTFNM
TaLsi1        FRHFPWIQVPFYWAAQFTGAICASFVLKAVLHPITVIGTTEPVGPHWHALVIEVVVTFNM
ZmNIP2;1      FRHFPWIQVPFYWAAQFTGSICASFVLKAVLHPIAVLGTTTPTGPHWHSLVIEIIVTFNM
OsNIP2;1      FRHFPWIQVPFYWAAQFTGAICASFVLKAVIHPVDVIGTTTPVGPHWHSLVVEVIVTFNM
ZmNIP2;2      FRHFPWIQVPFYWAAQFTGAMCAAFVLKAVLHPIAVIGTTTPSGPHWHALLIEIVVTFNM
OsNIP2;2      FRHFPWIQVPFYWAAQFTGAMCAAFVLRAVLYPIEVLGTTTPTGPHWHALVIEIVVTFNM
CmNIP2;1      TRHFPWKQVPLYAAAQLSGATCAAFTLRLLLHPIKHLGTTTPSGSDLQALVMEIVVTFSM
GmNIP2;1      VRHLPWPQLPFYIAAQLTGAISASYTLRELLRPSNEIGGTSPAGSHIQALIMEMVTTYTM
GmNIP2;2      VRHLPWPQLPFYVAAQLTGAISASYTLRELLRPSDEIGGTSPAGSHIQALIMEMVSTYTM
               **:** ::* * ***::*: .*::.*: :: *   :* * * *.. ::*::*:: *: *
```

```
HbNIP2;1      MFVTSAVATDTKAIGELAGVAVGSAVCITSILAGPVSGGSMNPARSLGPAIASAYYKGIW
HvNIP2;1      MFVTLAVATDTRAVGELAGLAVGSSVCITSIFAGAVSGGSMNPARTLGPALASNRYPGLW
TaLsi1        MFVTLAVATDTRAVGELAGLAVGSSVCITSIFAGAVSGGSMNPARTLGPALASNRYPGLW
ZmNIP2;1      MFVTLAVATDTRAVGELAGLAVGSAVCITSIFAGAVSGGSMNPARTLGPALASNLYTGLW
OsNIP2;1      MFVTLAVATDTRAVGELAGLAVGSAVCITSIFAGAISGGSMNPARTLGPALASNKFDGLW
ZmNIP2;2      MFVTCAVATDSRAVGELAGLAVGSAVCITSIFAGPVSGGSMNPARTLAPAVASNVFTGLW
OsNIP2;2      MFVTCAVATDSRAVGELAGLAVGSAVCITSIFAGPVSGGSMNPARTLAPAVASNVYTGLW
CmNIP2;1      MFVTCAVATDTKAVGELAGLAVGSAVCITSILAGPVSGGSMNPVRTLGPAMASDNYKGLW
GmNIP2;1      VFISMAVATDSNATGQLSGVAVGSSVCIASIVAGPISGGSMNPARTLGPAIATSYYKGLW
GmNIP2;2      VFISMAVATDSNATGQLSGVAVGSSVCIASIVAGPISGGSMNPARTLGPAIATSYYKGLW
              :*:: *****:.* *:*:*:****:***:**.**.:*******.*:*.**:*:  : *:*

HbNIP2;1      VYIIGPVVGTLLGACSYNLIRVTDQP---IQAIS---YSLKLRRIRSN-DEQAHNKDPFD
HvNIP2;1      LYFLGPVLGTLSGAWTYTYIRFEDPP--KDA--PQKLSSFKLRRLQSQ-SVAADD-DELD
TaLsi1        LYFLGPVLGTLSGAWTYTYIRFEDPP--KDG--PQKLSSFKLRRLQSQ-SVAADD-DELD
ZmNIP2;1      IYFLGPVLGTLSGAWTYTYIRFEEAPSHKDM--SQKLSSFKLRRLQSQ-SVAVDD-DELD
OsNIP2;1      IYFLGPVMGTLSGAWTYTFIRFEDTP-KEGS—SQKLSSFKLRRLRSQQSIAADDVDEME
ZmNIP2;2      IYFLGPVIGTLSGAWVYTYIRFEEAPAAKD---TQRLSSFKLRRMQSQ--LAADEFDTV-
OsNIP2;2      IYFLGPVVGTLSGAWVYTYIRFEEAPAAAGGAAPQKLSSFKLRRLQSQ-SMAADEFDNV-
CmNIP2;1      VYFVGPVTGTLLGAWSYKFIRASDKP---VHLISPHSFSLKLRRMSRS-DVGEGER----
GmNIP2;1      VYFVGPITGAVLAAWSYNVIRDTEHP---GFPISLSSISSKVRQSIGGTEQKSDQRCLV-
GmNIP2;2      VYFVGPITGAVLAAWSYNVIRDTEHP---GFPISLSSISSKVRQSIGGTEQKSDQRCLV-
              :*::* : *:: .:  *. **  : *       .    * *:*:          :
```

Urea Transporters

```
HbPIP1;1      VGIQGIAWSFGGMIFALVYCTAGISGGHINPAVTFGLFLARKLSLTRALYYMVMQCLGAI
HbPIP1;2      VGIQGIAWAFGGMIFALVYCTAGISGGHINPAVTFGLFLARKLSLTRALYYMVMQCLGAI
HbPIP1;3      VGTQGIAWAFGGMIFALVYCTAGISGGHINPAVTFGLFLARKLSLTRALFYIIMQCLGAI
HbPIP1;4      VGTQGIAWAFGGMIFALVYCTAGISGGHINPAVTFGLFLARKLSLTRALFYMIMQCLGAI
HbPIP1;5      VGVQGIAWAFGGMIFVLVYCTAGISGGHINPAVTFGLTLARKVSLTRAIFYMVMQCLGAI
HbPIP2;1      VGILGIAWAFGGMIFILVYCTAGISGGHINPAVTLGLFLARKVSLVRAILYMAAQCLGAI
HbPIP2;2      VGILGIAWAFGGMIFILVYCTAGISGGHINPAVTFGLFLARKVSLVRAVLYMAAQCLGAI
HbPIP2;3      VGILGIAWAFGGMIFILVYCTAGISGGHINPAVTFGLFLGRKVSLIRALLYMVAQCLGAI
HbPIP2;4      VGILGIAWAFGGMIFILVYCTAGISGGHINPAVTFGLFLGRKVSLIRALLYMVAQCLGAI
HbPIP2;5      VGILGIAWAFGGMIFILVYCTAGISGGHINPAVTFGLFLARKVSLVRAVMYMVAQCLGAI
HbPIP2;6      VGILGIAWAFGGMIFILVYCTAGISGGHINPAVTFGLFLARKISLVRAVMYMVAQCLGAI
HbPIP2;7      VGLLGIAWAFGGMIFILVYCTAGISGGHINPAVSFGLFLARKVSLIRAVAYMVAQCLGAI
HbPIP2;8      VGLLGIAWAFGGMIFILVYCTAGISGGHINPAVTFGLFLARKVSLIRAVAYMVAQCLGAI
HbPIP2;9      VGFLGIAWSFGGMIFILVYCTAGISGGHINPAVTFGLFLARKVSLVRAIAYMVAQCLGAI
HbPIP2;10     VGFLGVAWAFGGMIFILVYCTAGISGGHINPAVTFGLLLARKLSLVRAVAYMVSQCLGAI
HbTIP1;1      AGLVAASIAHAFALFVAVSVGANISGGHVNPAVTFGAFVGGNITLLRGILYWIAQLLGST
HbTIP1;2      AGLVAASIAHAFALFVAVSVGANISGGHVNPAVTFGAFVGGNITLLRGILYWIAQLLGST
HbTIP1;3      AGIIMASLAHAFGLFVGVSTATNISNGHVNPAVTFGAFLGGNISLLRGILYWIAQLLGST
HbTIP1;4      AGIIMASLAHAFGLFVGVSTAFNISGGHVNPAVTFGAFLGGSISLIRGILYWIAQLLGST
HbTIP1;5      AGLIAASLAHAFALFVAVSVGANISGGHVNPAVTFGAFIGGNITLLRGILYWIAQLLGSV
HbTIP1;6      AGLIAASLAHAFALFVAVSVGANISGGHVNPAVTFGAFIGGNITLLRGILYWIAQLLGSV
HbTIP1;7      AGLVAASLAHGFALFVAVSVGANISGGHVNPAVTFGAFVGGHITFIRSVLYWIAQLLGSV
HbTIP1;8      AGLVAASLAHGFALFVAVSVGANISGGHVNPAVTFGAFVGGHITLIRSVLYWVAQLLGSV
HbTIP2;1      AGLVAIAICHGFALFVAVSVGANISGGHVNPAVTFGLALGGQITILTGIFYWIAQLLGSI
HbTIP2;2      AGLVAIAICHGFALFVAVAVGANISGGHVNPAVTFGLALGGQITILTGVFYWIAQLLGSI
HbTIP2;3      PGLVAVAVAHAFALFVGVAIAANISGGHLNPAVTFGLAVGGNITILTGIFYWIAQCLGSI
HbTIP3;1      SGLVMIALAHALALFSAVSASINISGGHVNPAVTFGALVGGRISVLQAFYYWVAQLLGAI
HbTIP3;2      SGLVMIALAHALALFSALSASINISGGHVNPAVTFGALVGGRISVLRALYYWVAQLLGSI
HbTIP5;1      SSLVIVAIANSFALSSAVYIAANISGGHVNPAVTFSLAVGGHISVPTALFYWISQMLASV
HbTIP5;2      SSLVIVAIANAFALSSAVYIAANVSSGHVNPAVTFSLAVGGHINVPTAIFYWISQMLASV
HbNIP2;1      ISKLGASVAGGLIVTVMIYAVGHVSGAHMNPAVTTAFAAFRHFPWKQVPFYAAAQLTGAI
HbNIP5;1      ESLIGNAACAGLAVMIIILSTGHISGAHLNPSLTIAFAALRHFPWMQVPAYIAAQVSASI
HbNIP6;1      ETLIGLAASTGLAVMIVILSTGHISGAHLNPSVTIAFAALKHFPWKHVPVYIGAQVMASV
HbXIP1;1      EPKLLVPFAVFIIAFFFLLTTVPLSGGHMSPVFTFIAALKGVITLVRALLYVLAQCIGSI
HbXIP1;2      EPKLLIPIAVIVIAFLLLVVTVPLSGGHMSPIFTFISALRGLITLVRALFNVLAQCVGSI
HbXIP3;1      IPNLILSCLVAITVTIILLATYPISGGHINPLVTFSAALTGLISMTKAFIYILAQCAGGV
HbXIP2;1      TPNLIMSALIAITVTILLNATFPISGGHINPVITLSAAFTGLVSLSRAAIYILAQCLGGI
NtAQP1        VGIQGVAWAFGGMIFALVYCTAGISGGHINPAVTFGLFLARKLSLTRAIFYIVMQCLGAI
ZmPIP1;5      VGIQGIAWSFGGMIFALVYCTAGISGGHINPAVTFGLFLARKLSLTRALFYMVMQCLGAI
NtTIPa        VSLFFVAMAHALVVAVTISAGFRISGGHLNPAVTLGLCMGGHITVFRSILYWIDQLLASV
AtTIP1;1      SGLVAAAVAHAFGLFVAVSVGANISGGHVNPAVTFGAFIGGNITLLRGILYWIAQLLGSV
AtTIP1;2      SGLVAAALAHAFGLFVAVSVGANISGGHVNPAVTFGVLLGGNITLLRGILYWIAQLLGSV
AtTIP1;3      AGLVAASLSHAFALFVAVSVGANVSGGHVNPAVTFGAFIGGNITLLRAILYWIAQLLGAV
AtTIP2;1      PGLVAIAVCHGFALFVAVAIGANISGGHVNPAVTFGLAVGGQITVITGVFYWIAQLLGST
AtTIP4;1      VGLFAVAVAHAFVVAVMISAG-HISGGHLNPAVTLGLLLGGHISVFRAFLYWIDQLLASS
AtTIP5;1      FGVLIPAIANALALSSSVYISWNVSGGHVNPAVTFAMAVAGRISVPTAMFYWTSQMIASV
CpNIP1        VSQLGASVAGGLIVTVMIYAVGHISGAHMNPAVTTAFAATRHFPWKQVPLYAAAQLSGAT
OsNIP2;1      ISQLGQSIAGGLIVTVMIYAVGHISGAHMNPAVTLAFAVFRHFPWIQVPFYWAAQFTGAI
AtNIP6;1      ETLIGCAASAGLAVMIVILSTGHISGAHLNPAVTIAFAALKHFPWKHVPVYIGAQVMASV
NtXIP1;1α     MPNLIMSILIAIVITILLLAVVPVSGGHINPVISFSAALVGIISMSRAIIYMVAQCVGAI
NtXIP1;1β     MPNLIMSILIAIVITILLLAVVPVSGGHINPVISFSAALVGIISMSRAIIYMVAQCVGAI
StXIP1;1α     MPNLIMSILIAVVITILLLAVVPVSGGHINPVISFSAALVGIISMSRAIIYIVAQCLGAV
StXIP1;1β     MPNLIMSILIAVVITILLLAVVPVSGGHINPVISFSAALVGIISMSRAIIYIVAQCLGAV
                    .          :     :*..*:.* .:        .           *  ..
```

```
HbPIP1;1      CGAGVVKGFEGRHQYTLLGGGANSVNPGYTKGDG------------LGAEIVGTFVLVYT
HbPIP1;2      CGAGVVKGFEGRHQYTLLGGGANSVNPGYTKGDG------------LGAEIVGTFVLVYT
HbPIP1;3      CGAGVVKGFEGDRVYETLGGGANVVAHGYTKGDG------------LGAEIVGTFVLVYT
HbPIP1;4      CGAGVVKGFEGNRAYETLGGGANVVAHGYTKGDG------------LGAEIVGTFVLVYT
HbPIP1;5      FGAGIVKGFQPT-PFETLGGGANVVNPGYSKGDG------------LGAEIVETFALVYT
HbPIP2;1      CGCGLVKAFQKA-YYNRYGGGANELADGYSKGTG------------LGAEIIGTFVLVYT
HbPIP2;2      CGCGLVKAFQKA-YYNRYGGGANELADGYSKGTG------------LGAEIIGTFVLVYT
HbPIP2;3      CGCGLVKAFQKA-YYTRYGGGANELSSGYSKGTG------------LGAEIIGTFVLVYT
HbPIP2;4      CGCGLVKAFQKA-YYNRYGGGANELSDGYNKGTG------------LGAEIIGTFVLVYT
HbPIP2;5      AGVGLVKAFQSS-FYKRYGGGANSLAAGYSKGVG------------LGAEIIGTFVLVYT
HbPIP2;6      AGVGLVKAFQSS-HYKRYGGGANSLANGYSTGVG------------LGAEIIGTFVLVYT
HbPIP2;7      CGVGLVKAFMKH-PYNGLGGGANTVAPGYNKGTA------------LGAEIIGTFVLVYT
HbPIP2;8      CGVGLVKAFMKH-PYNALGGGANSVAHGYNKGTA------------LGAEIIGTFVLVYT
HbPIP2;9      CGVGIVKGIMKD-FYNAQGGGANTVAATYSKGTA------------LGAEIIGTFVLVYI
HbPIP2;10     AGAGLVKAVMKD-DYKSLGGGVNSVSSGYSKGTA------------LGAEIIGTFVLVYT
HbTIP1;1      VACLLLKFSTGG-----LTTAGFALSSGVGVWNA------------FVFEIVMTFGLVYT
HbTIP1;2      VACLLLKFSTGG-----LTTSAFALSSGVGVWNA------------FVLEIVMTFGLVYT
HbTIP1;3      VACLLLKFSTHG-----MTTSAFALSSGVNVWNA------------LVFEIVMTFGLVYT
HbTIP1;4      VACLLLKFSTHG-----MTASAFSLSVRGECVEC------------TCIRDCTDVCLVYT
HbTIP1;5      VACLLLKFATGG-----LETSAFALSSGVSSWNA------------LVFEIVMTFGLVYT
HbTIP1;6      VACLLLKFATGG-----LETSAFALSSGVSSWNA------------VVFEIVMTFGLVYT
HbTIP1;7      VACLLLKFATGG-----LETSAFALSSGVGAWNA------------LVFEIVMTFGLVYT
HbTIP1;8      VACLLLKFATGG-----WETSAFALSSGVGAGNA------------LVFEIVMTFGLVYT
HbTIP2;1      VACFLLKFVTGD-----LPIPTHSVAAGVGAIEG------------VVMEIVITFALVYT
HbTIP2;2      VACFLLKFVTGG-----LAIPTHSVAAGVGAIEG------------VVMEIVITFALVYT
HbTIP2;3      VACLLLQFVTNG-----KSVPTHGVASGMNAFEG------------VIMEIIITFALVYT
HbTIP3;1      VASLLLRLVTNG-----MRPVGFYIASGVGEVHG------------LIMEMVMTFGLVYT
HbTIP3;2      VASLLLRLVTNR-----MRPVGFYVASGAAEVHG------------LILEMVMTFGLAYT
HbTIP5;1      MACLLLRVVIVG-----QSLPTYTIAEEMTGFGA------------SVIEGVLTFGLVYT
HbTIP5;2      MACLLLRVAIVG-----QSLPTYTIAAEMTGFGA------------SVFEGVLTFGLVYT
HbNIP2;1      SASFTLRVLLHP-----IKQVGTTSPS-GSDLQA------------LIMEIVVTFSMMFV
HbNIP5;1      CASFALKGVFHP-----FMSGGVTVPS-VSTGQA------------FALEFLITFNLLFV
HbNIP6;1      SAAFALKGIFHP-----IMGGGVTVPS-GGYGQA------------FALEFIISFNLMFV
HbXIP1;1      MAYMVIKNVMNNSAVEKYSLGGCMID----GNEG-----GIASGTALVLEFSCTFVVLFV
HbXIP1;2      MAYLVIKSVMNENTAEKYSLGGCMVN----GNRS-----GVNAGTALILEFTCSFLVLYV
HbXIP2;1      LGALALKAVVNSTIEKTFSLGGCTLSIVAPGPHG-PIVIGLGTAQALWLEIICTFVFLFS
HbXIP3;1      VGALALKAVVNSKIESTFSLGGCTLHIVAPGPDGRPTVIGLETGQALWLEIICGFVFLFA
NtAQP1        CGAGVVKGFMVG-PYQRLGGGANVVNHGYTKGDG------------LGAEIIGTFVLVYT
ZmPIP1;5      CGAGVVKGFQEG-LYMGAGGGANAVNPGYTKGDG------------LGAEIVGTFVLVYT
NtTIPa        AACALLNYLTAG-----LETPVHTLANGVSYGQG------------IIMEVILTFSLLFT
AtTIP1;1      VACLILKFATGG-----LAVPAFGLSAGVGVLNA------------FVFEIVMTFGLVYT
AtTIP1;2      AACFLLSFATGG-----EPIPAFGLSAGVGSLNA------------LVFEIVMTFGLVYT
AtTIP1;3      VACLLLKVSTGG-----METAAFSLSYGVTPWNA------------VVFEIVMTFGLVYT
AtTIP2;1      AACFLLKYVTGG-----LAVPTHSVAAGLGSIEG------------VVMEIIITFALVYT
AtTIP4;1      AACFLLSYLTGG-----MGTPVHTLASGVSYTQG------------IIWEIILTFSLLFT
AtTIP5;1      MACLVLKVTVME-----QHVPIYKIAGEMTGFGA------------SVLEGVLAFVLVYT
CpNIP1        CAAFTLRLLLHP-----IKHLGTTTPS-GSDLQA------------LVMEIVVTFSMMFV
OsNIP2;1      CASFVLKAVIHP-----VDVIGTTTPV-GPHWHS------------LVVEVIVTFNMMFV
AtNIP6;1      SAAFALKAVFEP-----TMSGGVTVPT-VGLSQA------------FALEFIISFNLMFV
NtXIP1;1α     LGALALKAVVSSTIAQTFSLGGCTITVIAPGPNG-PITVGLEMAQALWLEIFCTFVFLFA
NtXIP1;1β     LGALALKAVVSSTIAQTFSLGGCTITVIAPGPNG-PITVGLEMAQALWLEIFCTFVFLFA
StXIP1;1α     LGALALRAVVSSSIEDTFSLGGCTVTIIAPGPNG-PVTVGLETAQALWLEIFCTFVFLFA
StXIP1;1β     LGALALRAVVSSSIEDTFSLGGCTVTIIAPGPNG-PVTVGLETAQALWLEIFCTFVFLFA
               .   :                                           .    . . :
```

```
HbPIP1;1      VFSATDAKRNARDS---HVPILAPLPIGFAVFLVHLATI--PITGTGINPARSLGAAIIF
HbPIP1;2      VFSATDAKRNARDS---HVPILAPLPIGFAVFLVHLATI--PITGTGINPARSLGAAIIF
HbPIP1;3      VFSATDAKRNARDS---HVPILAPLPIGFAVFLVHLATI--PITGTGINPARSLGAAIIF
HbPIP1;4      VFSATDAKRNARDS---HVPILAPLPIGFAVFLVHLATI--PITGTGINPARSLGAAIIF
HbPIP1;5      VLSATDAKRSARDS---HVPILASLPIGFAVFLVHLATI--PITGTGINPARSLGAAIVY
HbPIP2;1      VFSATDPKRNARDS---HVPVLAPLPIGFAVFMVHLATI--PVTGTGINPARSFGAAVIY
HbPIP2;2      VFSATDPKRNARDS---HVPVLAPLPIGFAVFMVHLATI--PVTGTGINPARSFGAAVIY
HbPIP2;3      VFSATDPKRNARDS---HVPVLAPLPIGFAVFMVHLATI--PITGTGINPARSFGAAVIY
HbPIP2;4      VFSATDPKRNARDS---HVPVLAPLPIGFAVFMVHLATI--PITGTGINPARSFGAAVIY
HbPIP2;5      VFSATDPKRNARDS---HVPVLAPLPIGFAVFMVHLATI--PITGTGINPARSLGAAVIY
HbPIP2;6      VFSATDPKRSARDS---HVPVLAPLPIGFAVFMVHLATI--PITGTGINPARSLGAAVIY
HbPIP2;7      VFSATDPKRSARDS---HVPVLAPLPIGFAVFMVHLATI--PITGTGINPARSFGAAVIY
HbPIP2;8      VFSATDPKRSARDS---HVPVLAPLPIGFAVFVVHLATI--PVTGTGINPARSFGAAVIY
HbPIP2;9      VLSATDPKRNARDS---HVPVLAPLPIGFAVFMVHLATI--PITGTGINPARSFGAAVIY
HbPIP2;10     VFSATDPKRKARDS---FVPILVPLPIGFAVFVVHLATI--PITGTGINPARSLGPAVIY
HbTIP1;1      VYATAIDPKKG------NLGIIAPIAIGFIVGANILAGG--AFDGASMNPAVSFGPALVS
HbTIP1;2      VYATAIDPKKG------NLGIIAPIAIGFIVGANILAGG--AFDGASMNPAVSFGPALVS
HbTIP1;3      VYATAFDRNKG------DVGIIAPLAIGFVVGANILAGG--AFEGASMNPAVSFGPALVS
HbTIP1;4      VYATALDPKKG------EVGIIALLAIGFVVGANILAGG--AFEGASMNPAVSFGPALVS
HbTIP1;5      VYATAVDPKKG------NVGIVAPIAIGFIVGANILAGG--AFDGASMNPAVSFGPAVVS
HbTIP1;6      VYATAVDPKKG------NVGTVAPIAIGFIVGANILAGG--AFDGASMNPAVSFGPAVVS
HbTIP1;7      VYATAVDPKKG------NIGIIAPIAIGFIVGANILAGG--AFDGASMNPAVSFGPAVVS
HbTIP1;8      VYATAVDPKKG------DIGIIAPIAIGFIVGANILAGG--AFDGASMNPAVSFGPAVVS
HbTIP2;1      VYATAADPKKG------SLGIIAPIAIGFIVGANILAAG--PFSGGSMNPARSFGPAVAS
HbTIP2;2      VYATAADPKKG------SLGIIAPIAIGFIVGANILAAG--PFSGGSMNPARSFGPAVAS
HbTIP3;1      VYATAVDPKRG------SLGIIAPLAIGFIVGANILVGG--PFDGASMNPARAFGPALVG
HbTIP3;2      VYATAVDTNRG------SLGIIAPLAIGLIVGANILVGG--PFDGASMNPARAFGPALVG
HbTIP2;3      VYATAADPKKG------NLGIIAPIAIGFIVGANILAAG--PFSGGSMNPARSFGPAVVS
HbTIP5;1      VYA-AGDPRRS------LQGVTGPLAIGLMAGANVLAAG--PFSGGSMNPACAFGSAVIA
HbTIP5;2      VYA-AGDPRCS------LLGATGPLAIGLMAGANVLAAG--PFSGGSMNPACAFGSAVIA
HbNIP2;1      TSAVATDTK--------AIGELAGVAVGSAVCITSILAG--PVSGGSMNPARSLGPAIAS
HbNIP5;1      VTAVATDTR--------AVGELAGIAVGATVMLNILVAG--PSSGGSMNPVRTLGPAVAA
HbNIP6;1      VTAVATDTR--------AVGELAGIAVGATVMLNILIAG--QSTGASMNPVRTLGPAIAA
HbXIP1;1      GVTVAFDKRRFKELGLVMVCVILAATMGLAIFVSITVTGRGGYAGVGLNPARCLGPALLH
HbXIP1;2      AITIAFNKKMCQELGFTMVCVIVAGVYALAVFASITVTGQAGYRGVGLNPARFLGPALLL
HbXIP2;1      SIWVAFDKRQAKPLGRVIVCSIIGLVVGLLVFISTTVTATRGYAGVGMNPARCFGPAIIR
HbXIP3;1      SVWMAFDHRQAKALGHVKIFMIVGIVLGLLVYVSTSVTTAKGYAGAGLNPARCLGPAIVR
NtAQP1        VFSATDAKRNARDS---YVPILAPLPIGFAVFLVHLATI—PITGTGINPARSLGAAIIY
ZmPIP1;5      VFSATDAKRSARDS---HVPILAPLPIGFAVFLVHLATI--PITGTGINPARSLGAAIVY
NtTIPa        VYTTIVDPKKG------ILEGMGPLLTGLVVGANIMAGG--PFSGASMNPARSFGPAFVS
AtTIP1;1      VYATAIDPKNG------SLGTIAPIAIGFIVGANILAGG--AFSGASMNPAVAFGPAVVS
AtTIP1;2      VYATAVDPKNG------SLGTIAPIAIGFIVGANILAGG--AFSGASMNPAVAFGPAVVS
AtTIP1;3      VYATAVDPKKG------DIGIIAPLAIGLIVGANILVGG--AFDGASMNPAVSFGPAVVS
AtTIP2;1      VYATAADPKKG------SLGTIAPLAIGLIVGANILAAG--PFSGGSMNPARSFGPAVAA
AtTIP4;1      VYATIVDPKKG------SLDGFGPLLTGFVVGANILAGG--AFSGASMNPARSFGPALVS
AtTIP5;1      VFT-ASDPRRG------LPLAVGPIFIGFVAGANVLAAG--PFSGGSMNPACAFGSAMVY
CpNIP1        TCAVATDTK--------AVGELAGLAVGSAVCITSILAG--PVSGGSMNPVRTLGPAMAS
OsNIP2;1      TLAVATDTR--------AVGELAGLAVGSAVCITSIFAG--AISGGSMNPARTLGPALAS
AtNIP6;1      VTAVATDTR--------AVGELAGIAVGATVMLNILIAG--PATSASMNPVRTLGPAIAA
NtXIP1;1α     SIWMAYDHRQAKALGLVTVLSIVGIVLGLLVFISTTVTMKKGYAGAGMNPARCFGAAVVR
NtXIP1;1β     SIWMAYDHRQAKALGLVTVLSIVGIVLGLLVFISTTVTMKKGYAGAGMNPARCFGAAVVR
StXIP1;1α     SIWMAYDHRQAKALGHVTVLSIVGLVLGLLVFISTTVTAKKGYGGAGINPARCLGPAIIR
StXIP1;1β     SIWMAYDHRQAKALGHVTVLSIVGLVLGLLVFISTTVTAKKGYGGAGINPARCLGPAIIR
                    .                  .                . .:**.  :*.*.
```

Additional file 6: SDP analysis of rubber tree aquaporins from alignments with putative amino acid sequences of AQPs transporting non-aqua substrates.

Multiple alignments were performed using ClustalW. Shown is the sequences containing the nine SD positions which are highlighted in yellow and the representative sequences are marked in blue. The numbers at the end of each line indicate amino acid position excluding the introduced gaps (number without parentheses) or including the introduced gaps (number in parentheses). GenBank/Phytozome accession numbers: AtPIP1;2 (AT2G45960), AtPIP2;1 (AT3G53420), AtPIP2;4 (AT5G60660), AtTIP1;1 (AT2G36830), AtTIP1;2 (AT3G26520), AtTIP1;3 (AT4G01470), AtTIP2;1 (AT3G16240), AtTIP2;3 (AT5G47450), AtTIP4;1 (AT2G25810), AtTIP5;1 (AT3G47440), AtNIP1;1 (AT4G19030), AtNIP1;2 (AT4G18910), AtNIP5;1 (AT4G10380), AtNIP6;1 (AT1G80760), CmNIP2;1 (BAK09176), CpNIP1 (CAD67694), GmNOD26 (P08995), GmNIP2;1 (XP_003534451), GmNIP2;2 (NP_001240190), HvPIP1;3 (BAA23745), HvPIP1;4 (BAF33068), HvPIP2;1 (BAA23744), HvNIP2;1 (BAH24163), NtAQP1 (O24662), NtTIPa (Q9XG70), NtXIP1-1α (ADO66667), NtXIP1-1β (ADO66666), OsNIP2;1 (Q6Z2T3), OsNIP2;2 (Q67WJ8), OsNIP3;2 (Q7EYH7), SlXIP1-1α (ADO66672), SlXIP1-1β (ADO66671), StXIP1-1α (ADO66669), StXIP1-1β (ADO66670), TaLsi1 (ADM47602), TaTIP2;1 (AAS19468), TaTIP2;2 (AAS19469), TgTIP1-1 (BAL41683), TgTIP1-2 (BAL41684), ZmPIP1;1 (Q41870), ZmPIP1;5 (Q9AR14), ZmNIP2;1 (Q19KC1), ZmNIP2;2 (Q9ATN2).
