# Supplementary material for: Genome-wide identification of rubber tree (Hevea brasiliensis Muell. Arg.) aquaporin genes and their response to ethephon stimulation in the laticifer, a rubber-producing tissue
Source: BMC Genomics. 2015 Nov 25;16:1001. doi: 10.1186/s12864-015-2152-6 (PMC4658816; doi:10.1186/s12864-015-2152-6)

Additional file 7: Expression profiles of the 51 HbAQP genes in the laticifer of rubber tree clone RRIM928. Shown is 22 expressed HbAQP genes, which includes five extremely low expressed genes (*HbPIP2;2*, *HbPIP2;6*, *HbPIP2;8*, *HbPIP2;9* and *HbTIP2;1*) but without *HbTIP1;2* and *HbNIP6;1* that identified in the laticifer of clone PR107. The result is based on the Illumina transcriptome data of SRX278513.

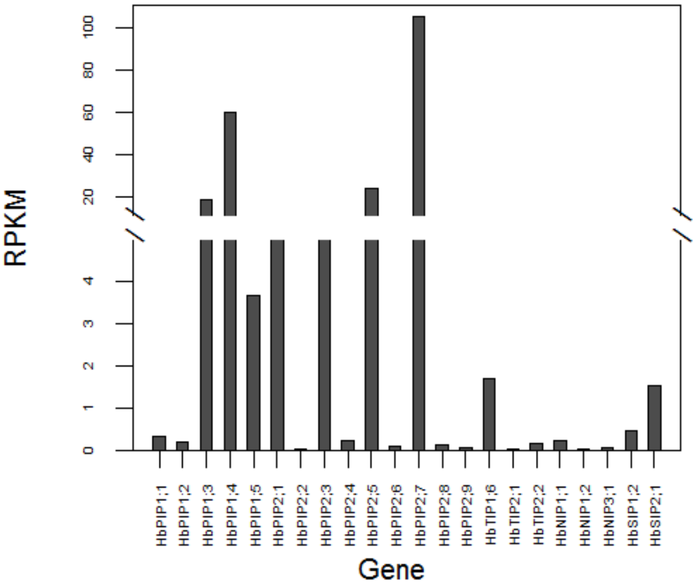

Supplement: Additional file 7: — Expression profiles of the 51 HbAQP genes in the laticifer of rubber tree clone RRIM928. (PDF 36 kb) [file 12864_2015_2152_MOESM7_ESM.pdf]
